# DFT/NMR Approach for the Configuration Assignment of Groups of Stereoisomers by the Combination and Comparison of Experimental and Predicted Sets of Data

**DOI:** 10.1021/acs.joc.9b03129

**Published:** 2020-01-21

**Authors:** Gianluigi Lauro, Pronay Das, Raffaele Riccio, D. Srinivasa Reddy, Giuseppe Bifulco

**Affiliations:** † Department of Pharmacy, University of Salerno, Via Giovanni Paolo II 132, Fisciano 84084, Italy; ‡ Organic Chemistry Division, CSIR-National Chemical Laboratory, Dr. Homi Bhabha Road, Pune 411008, India; § Academy of Scientific and Innovative Research (AcSIR), New Delhi 110025, India

## Abstract

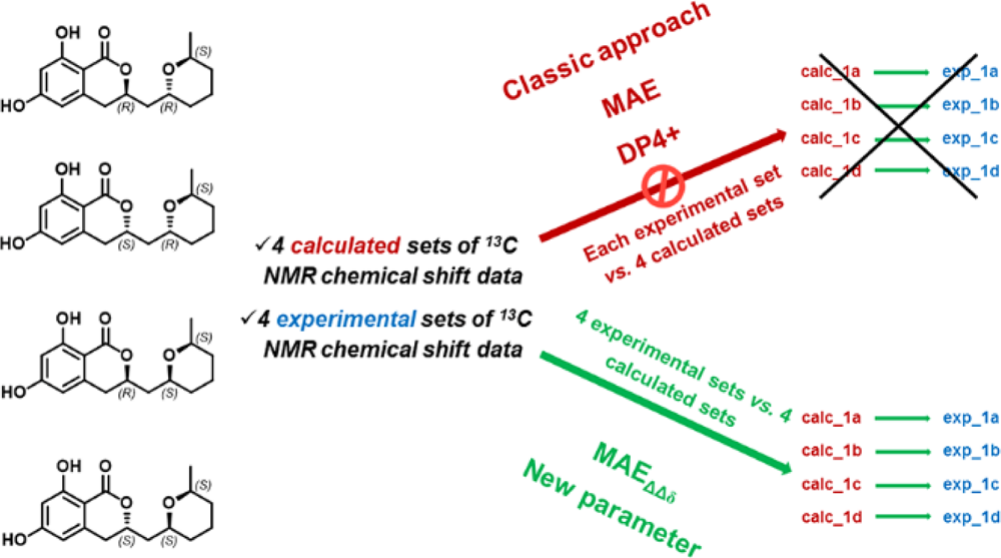

Quantum mechanical/nuclear magnetic resonance (NMR) approaches are widely used for the configuration assignment of organic compounds generally comparing one cluster of experimentally determined data (e.g., ^13^C NMR chemical shifts) with those predicted for all possible theoretical stereoisomers. More than one set of experimental data, each related to a specific stereoisomer, may occur in some cases, and the accurate stereoassignments can be obtained by combining the experimental and computed data. We introduce here a straightforward methodology based on the simultaneous analysis, combination, and comparison of all sets of experimental/calculated ^13^C chemical shifts for aiding the correct configuration assignment of groups of stereoisomers. The comparison of the differences between the calculated/experimental chemical shifts instead of the shifts themselves led to the advantage of avoiding errors arising from calibration procedures, reducing systematic errors, and highlighting the most diagnostic differences between calculated and experimental data. This methodology was applied on a tetrad of synthesized cladosporin stereoisomers (cladologs) and further corroborated on a tetrad of pochonicine stereoisomers, obtaining the correct correspondences between experimental and calculated sets of data. The new MAE_ΔΔδ_ parameter, useful for indicating the best fit between sets of experimental and calculated data, is here introduced for facilitating the stereochemical assignment of groups of stereoisomers.

## Introduction

Nuclear magnetic resonance (NMR) spectroscopy is one of the pivotal analytical tools used to determine key chemical properties of organic compounds, for example, relative/absolute configurations,^1,2^ and to provide further structural information, for example, representative conformational patterns of the investigated molecules.^3^ In this context, the spectroscopic properties of organic compounds can be proficiently predicted by accurate quantum chemical methods.^1,4−7^ Indeed, the integration of the information from experimental and computational data can then be of fundamental importance to solve different structural issues of organic compounds. In the last decade, different studies were performed with the combination of the information from NMR spectroscopy (experimental part) and quantum mechanical (QM) calculations (predicted part) (QM/NMR integrated approach) for the successful elucidation of the configurational patterns of organic compounds.^1,4^ Also, this approach is helpful for the stereostructural assignment of natural compounds, thus representing a reliable alternative, faster and cheaper, to total synthesis.^8^ Also, the notable advances in computer science nowadays allows the performance of accurate conformational sampling and QM calculations even on desktop computers, thus facilitating the structural elucidation process.

The QM/NMR integrated approach, successfully applied by different research groups and us,^9−14^ is based on the assumption that the possible theoretical stereoisomers show different NMR features (e.g., ^1^H/^13^C chemical shifts and *J* coupling constants). Once both the experimental and predicted data are collected, their comparison may be quantified using different factors, such as by the mean absolute error (MAE),^1^ the corrected MAE,^1^ the root-mean-square deviation (RMSD), and the correlation coefficient *R* and, as reported in recent studies, by more challenging statistical parameters, such as the DP4 parameter by Goodman^15^ and the optimized DP4+ by Sarotti.^16^

Specifically, the general workflow for determining the relative/absolute configurational pattern of an organic compound relies on the following two main phases:^1,4,17^

- generation of the ensembles of conformers to be accounted for the subsequent prediction of the chemical properties (e.g., ^13^C/^1^H NMR chemical shift and *J* coupling constants). Generally, this step foresees extensive conformational searches for all possible theoretical stereoisomers by molecular mechanics (MM) methods; subsequently, the sets of conformers are geometry-optimized by QM methods, and the contribution of each conformer to the final Boltzmann population, according to the related computed energy, is then computed;
- extraction of the values, computation of the Boltzmann-weighted final set of data, and comparison with experimental values. The computed sets of data for all possible stereoisomers are generally compared with each single set of experimental values using specific quantitative parameters (e.g. MAE, DP4, and DP4+)^1,15,16^ useful for predicting the correct relative configurations (and in some cases, the absolute configurations, when coupled to other methods) of the case-study compound.

In particular, focusing on the last point, it is important to note that this methodology allows to predict the most probable stereoisomer as that featuring the best parameter value after generating a ranking (e.g., the lowest MAE value or the highest DP4+ probability). Using this approach, the results are dramatically affected by the set of stereoisomers accounted and the related sets of data: this means that if one of the theoretical isomers is excluded from the investigation, the application of this workflow anyway leads to a solution, identifying the most probable isomer among the set of accounted items. For the same reasons, if two (or more) sets of experimental data related to different isomers are available, this protocol could lead in principle to the identification of the same most probable solution, namely, the isomer whose computed data lead to the best ranked parameter values related to both the sets of experimental values. Accordingly, the assignment of the configurations of groups of stereoisomers (e.g., pairs, triads etc.) performed by comparing one-by-one each single set of experimental data to all sets of computed data could likely lead to errors. Contrarily, we speculated that accounting and comparing all sets of experimental/predicted data at the same time might be convenient for a more robust assignment.

Starting from these premises, in this study, we propose a method for the configuration assignment of groups of stereoisomers by accounting, combining, and comparing all possible sets of experimental and predicted chemical shift values in order to find the best match between the available data. As a proof of concept, we report the application of this methodology considering four synthesized cladosporin steroisomers (cladologs), whose related sets of ^13^C NMR experimental chemical shift values are available, and demonstrating how this approach led to the identification of the correct correspondences between experimental and calculated sets of data.

## Results and Discussion

### Comparing Experimental and Calculated Chemical Shift Data for Cladologs

**Chart 1:**
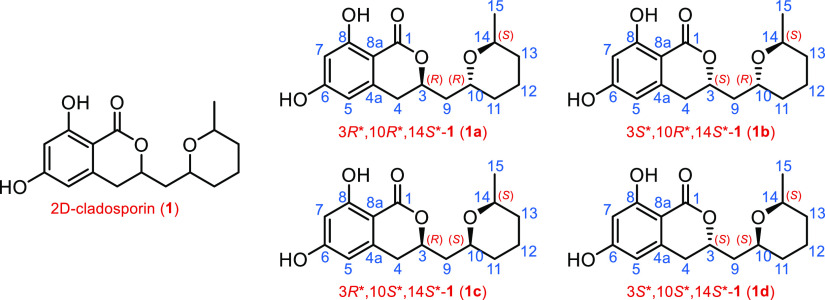
Chemical Structures of 2D-Cladosporin (**1**) Reference Compound and of Cladologs **1a–1d**

Cladosporin is a secondary metabolite isolated from fungal sources^18^ bearing three stereocenters and featuring 2*R*,9*R*,13*S* absolute configuration. In 2018, Reddy et al. reported a divergent synthesis of all eight possible stereoisomers based on the cladosporin 2D structure (**1**, Chart 1) (cladologs); also, all ^13^C NMR chemical shift data were assigned to each specific isomer.^19^ In the present study, the four cladologs featuring different relative configurations (**1a–1d**, Chart 1) were accounted, and then four sets of experimental data were considered for the subsequent comparison with the four calculated ones. Specifically, we named the sets of calculated data for **1a–1d** as calc_**1a**, calc_**1b**, calc_**1c**, and calc_**1d**, respectively. For simplicity, the sets of experimental data, assigned in the reference study^19^ (corroborated by comparison with already reported studies on cladosporin and related analogues^20,21^), were named for **1a–1d** as exp_**1a**, exp_**1b**, exp_**1c**, and exp_**1d**, respectively.

Concerning the computation of the ^13^C chemical shift data, after performing an extensive conformational search (see computational details, Experimental Section), the ensembles of sampled conformers were then submitted to a geometry and energy optimization step at the density functional theory (DFT) using the MPW1PW91/6-31g(d) functional/basis set.^22^ Then, for each isomer, ^13^C NMR chemical shifts were computed on the MPW1PW91/6-31g(d,p) level,^22^ considering the influence of each conformer on the total Boltzmann distribution taking into account the relative energies.

Once all experimental/calculated values were available, we started employing a classic QM/NMR approach in order to confirm the assignments for **1a–1d** and test this methodology when different experimental sets of data are available. Each experimental set of data was separately compared in detail with the four calculated ones; specifically, for each accounted atom, the experimental and calculated chemical shits (δ) were compared using the Δδ parameter
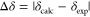
where δ_calc_ and δ_exp_ are the calculated and experimental chemical shift values, respectively.

After calculating all Δδ values, the MAE values and DP4+ probabilities were computed for determining which calculated set of data fits better with the experimental one. The MAE is defined as the following
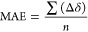
Namely, it is the summation (∑) of the n computed absolute δ error values (Δδ) normalized to the number of Δδ errors considered (*n*)

The obtained data, and precisely the MAE values, highlighted uncertain results that questioned the reliability of this procedure when multiple sets of experimental data are accessible. Specifically, exp_**1a** set of experimental data, assigned to compound **1a**, showed the best fit with calc_**1b**, featuring the lowest MAE values and highest DP4+ probability among the obtained rankings, thus not in accordance with the assignment reported in the reference study (Tables 1 and S1, Supporting Information). Moving to the exp_**1b** pattern of experimental data, the lowest MAE value and highest DP4+ probability were found against calc_**1b** among the ranking, in accordance with the starting assignment (Tables 1 and S2, Supporting Information). The third set of experimental data exp_**1c** fit with calc_**1c**, thus in agreement with the known assignments (Tables 1 and S3, Supporting Information). Finally, exp_**1d** set of experimental data, assigned to compound **1d**, showed the best DP4+ value with calc_**1c**, again not in accordance with the assignment reported in the reference study (Tables 1 and S4, Supporting Information). Summarizing, the same calculated set of data calc_**1b**, related to compound **1b**, showed both the best MAE values and DP4+ probabilities among the related rankings when compared to two different experimental sets of data (exp_**1a** and exp_**1b**) originally assigned to two different compounds (specifically, **1a** and **1b**). The same behavior was found with the calculated set of data calc_**1c**, related to compound **1c**, showing the best MAE values and/or DP4+ probabilities among the related rankings compared to exp_**1c** and exp_**1d** experimental sets of data. The obtained results prompted us to perform further calculations employing different levels of theory by the combination of various DFT functional/basis sets in order to obtain additional sets of computed ^13^C chemical shift data to be compared with the experimental ones. However, both expanding the basis set from 6-31g(d,p) to 6-311+g(d,p) on the same MPW1PW91 level and considering the B97-2/cc-pVTZ functional/basis set combination,^23^ the results did not show a remarkable improvement and basically confirmed what was obtained on the initial MPW1PW91/6-31g(d,p) level (Tables 1, and S5–S12, Supporting Information). The analysis of all these data highlighted that by comparing each experimental set of data against the four calculated ones separately, through the MAE and DP4+ rankings, the same theoretical stereoisomer can be predicted as the most probable one, and it is also the case if compared with different experimental sets of data. In summary, following this approach, we obtained ambiguous results that prompted us to find an alternative method to solve this issue.

**Table 1:**
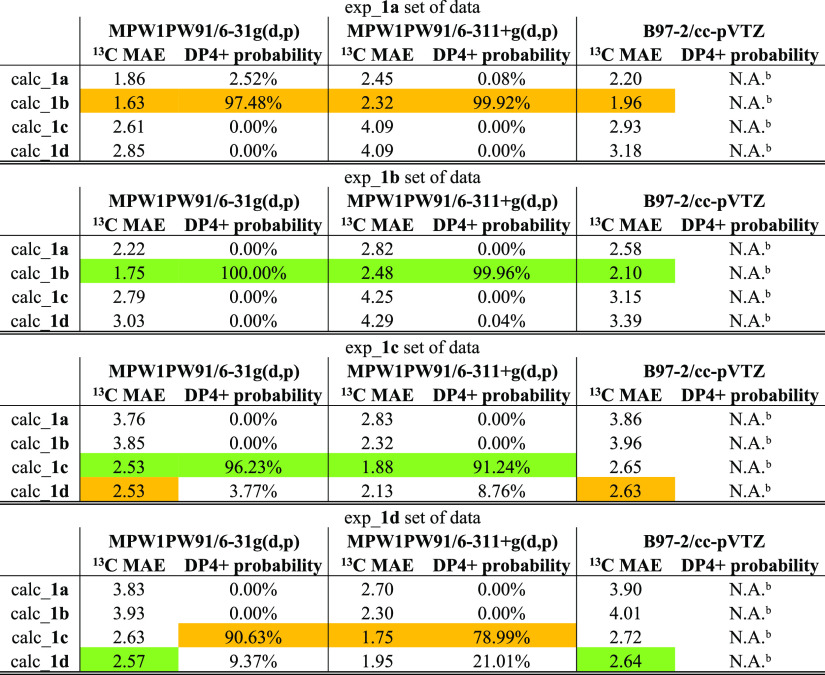
^13^C NMR MAE Values and DP4+ Probabilities Computed for the Three Functional/Basis Set Combinations Accounted in This Study Related to Compounds **1a–1d**^a^

a The correct/incorrect correspondence between experimental and calculated data are highlighted in green and orange, respectively.

b B97-2/cc-pVTZ functional/basis set combination cannot be set in the calculation of DP4+ probability.

**Figure 1:**
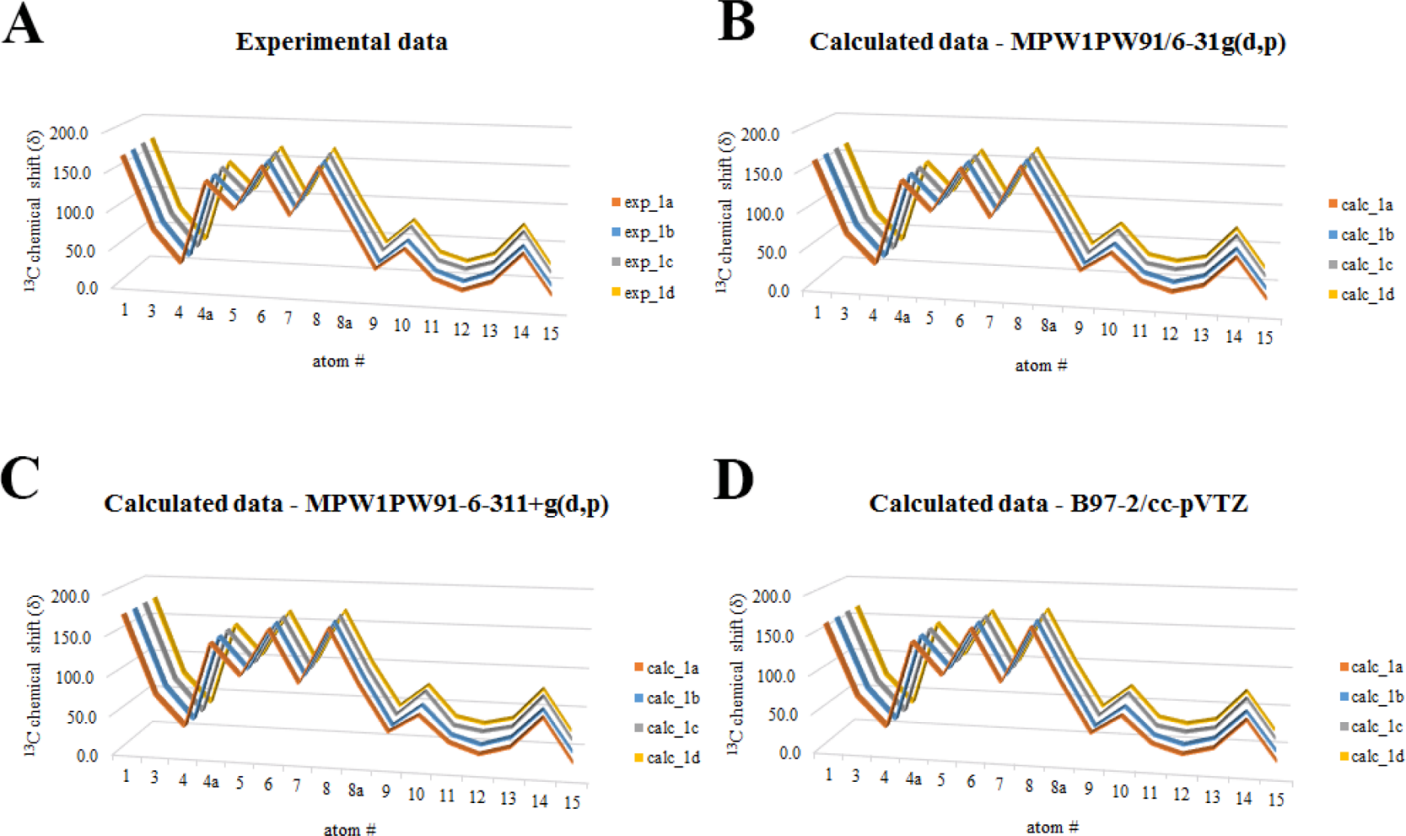
Line graphs related to the (a) experimental and (b–d) calculated ^13^C chemical shift values belonging to compounds **1a–1d**. In particular, concerning the computed data, those related to all three functional/basis set combinations were reported: (a) MPW1PW91/6-31g(d,p); (b) MPW1PW91/6-311+g(d,p); (c) B97-2/cc-pVTZ.

In particular, an accurate analysis of the experimental sets of data and the corresponding sets of calculated ones accounting the three employed functional/basis set combinations was performed; the deep investigation and comparison of all data highlighted the high similarity of the values (Figure 1), as clearly indicated by the computed averaged RMSD considering all investigated atoms for both the experimental and computed data sets, prompting us to find an alternative methodology for solving this stereochemical issue [averaged RMSD for experimental data set = 1.542 ppm, min. RMSD = 0.112 ppm, max RMSD = 4.058 ppm; averaged RMSD for calculated data set MPW1PW91/6-31g(d,p) = 0.932 ppm, min. RMSD = 0.011 ppm, max RMSD = 3.430 ppm; averaged RMSD for calculated data set MPW1PW91/6-311+g(d,p) = 1.103 ppm, min. RMSD = 0.062 ppm, max RMSD = 3.873 ppm; averaged RMSD for calculated data set B97-2/cc-pVTZ = 0.938 ppm, min. RMSD = 0.004 ppm, max RMSD = 3.430 ppm].

### Comparing all Calculated/Experimental Data: the MAE_ΔΔδ_ Parameter

Accordingly, we focused on managing all available clusters of data in a different manner, specifically combining all experimental and calculated sets of data at the same time in order to achieve a more robust comparison between all values. In this scenario, different methodologies were proposed based on this concept, such as the computation of the CP3 probability as proposed by Goodman.^24^ Specifically, this method is only applicable to pairs of stereoisomers, making its use poorly suitable when more stereoisomers must be considered. However, the CP3 approach highlighted the benefit in simultaneously accounting and comparing all experimental/calculated chemical shifts: specifically, aligned values drive the results toward the correct assignment and, accordingly, disarranged data aid in excluding incorrect stereoisomers.

In this study, we took advantage of the above concept, introducing an approach applicable on all of the available experimental/calculated sets of data. This methodology is based on building all possible combination alignment schemes between the experimental and calculated groups of values; afterward, all accounted combination alignment schemes are ranked accounting a specific parameter in order to propose the best fit between experimental and calculated patterns. It is inferable that increasing the number of accounted isomers (e.g., moving from two to three to four isomers and so on), the comparison of experimental/computed data becomes more arduous because the number of possible combinations increases. On the other hand, the availability of a large set of comparable data should lead to a more confident and robust assignment.

In more detail, the proposed methodology can be summarized in the following steps:

(a) generate all possible experimental/calculated comparison alignments between the sets of data. Specifically, a starting fixed sequence is defined for the calculated sets used as a reference since the stereochemistry related to each of them is known a priori. Then, all possible sequences related to the experimental set counterparts, for which the related stereochemistry must be determined, are built.

For the most simple case, that is, two stereoisomers, we can assume that two sets of calculated data, named A and B, and two sets of experimental data, named **1** and **2**, are available.

**Figure 2:**
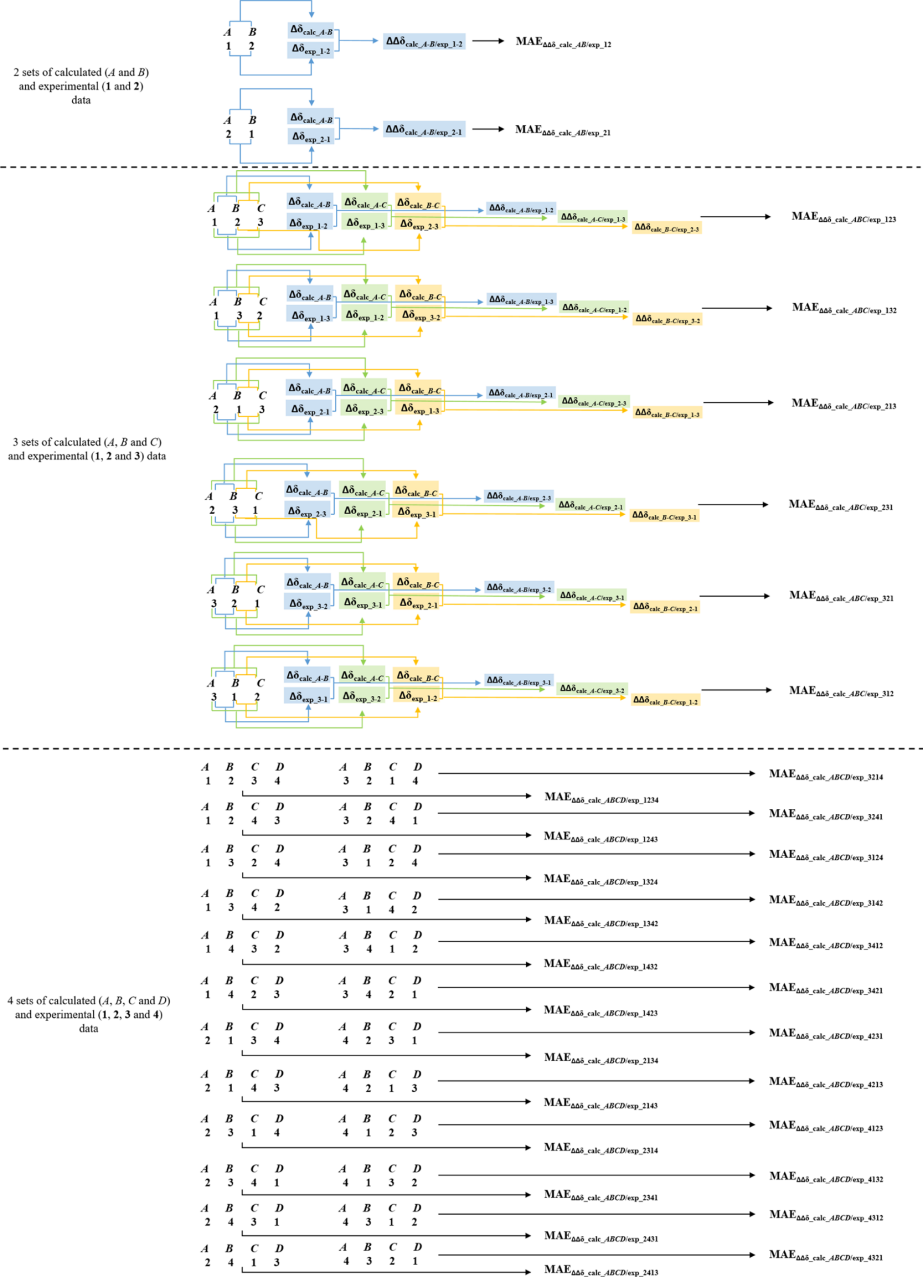
Possible combination alignments when two, three, and four sets of experimental/calculated data are available.

The possible comparison alignments are (Figure 2) as follows:

1) AB/**12**: calculated sets A and B corresponding to experimental **1** and **2**, respectively;
2) AB/**21**: calculated sets A and B corresponding to experimental **2** and **1**, respectively.

Thus, as the number of experimental/calculated sets increases, the number of comparison sequences increases. Indeed, with three stereoisomers, three sets of calculated, named A, B, and C, and three sets of experimental, named **1**, **2**, and **3**, will be accounted (Figure 1). The possible comparison sequences then will be

1) ABC/**123**: calculated A, B, and C corresponding to experimental **1**, **2**, and **3**, respectively;
2) ABC/**132**: calculated A, B, and C corresponding to experimental **1**, **3**, and **2**, respectively;
3) ABC/**213**: calculated A, B, and C corresponding to experimental **2**, **1**, and **3**, respectively;
4) ABC/**231**: calculated A, B, and C corresponding to experimental **2**, **3**, and **1**, respectively;
5) ABC/**321**: calculated A, B, and C corresponding to experimental **3**, **2**, and **1**, respectively;
6) ABC/**312**: calculated A, B, and C corresponding to experimental **3**, **1**, and **2**, respectively;

Starting from the number of experimental/calculated sets of data (*n*), the final number of comparison alignments (*c*) is then computed with the following relation
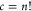
1

Then, for 2, 3, and 4 sets of experimental/calculated data 2, 6, and 24 possible combinations will be taken into account (Figure 2), respectively (eq 1).

(b) The differences between the chemical shift values belonging to each possible pair of calculated sets of data following the defined sequence are computed; then, the same procedure is applied to the experimental sets of data following the possible sequences (Figure 2). The obtained values will be subsequently compared, as described in the following (c) point (vide infra).

Specifically, for the above-reported case featuring two sets of calculated data (A and B) and two sets of experimental data (**1** and **2**),

1) AB/**12**: for each accounted atom, the difference (Δ) between each chemical shift (δ) belonging to the calculated set *A* and the corresponding value belonging to *B* is computed through the Δδ_calc_ parameter:

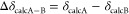
where δ_calcA_ and δ_calcB_ are the chemical shift values belonging to A and B sets of calculated data, respectively.

In the same way, the procedure is applied to the experimental sets, specifically computing Δδ between the chemical shifts belonging to **1** and **2** sets of values
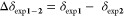
where δ_exp**1**_ and δ_exp**2**_ are the chemical shift values belonging to **1** and **2** sets of experimental data, respectively. Afterward, Δδ_calc_A–B_ and Δδ_exp_**1–2**_ values will be compared (vide infra, (c) point).

It is important to note that, in this step, the differences between the calculated (Δδ_calc_) and experimental (Δδ_exp_) chemical shifts of corresponding carbons are computed for the subsequent comparison (vide infra), following the idea by Belostotskii,^25^ Rodríguez,^26^ and Goodman,^24^ which highlighted the higher accuracy in comparing the differences between the chemical shifts than the shifts themselves because of the elimination of systematic errors.

(2) AB/**21**: again, Δδ_calc_A–B_ group of values are computed as reported above; contrarily, for the experimental sets of data, the chemical shift differences are computed following the new sequence, namely, between **2** and **1**, and leading to Δδ_calc_**2–1**_ group of values. In this case, Δδ_calc_A–B_ values will be then compared with those from Δδ_exp_**2–1**_ (vide infra, (c) point).

Moving to three calculated/experimental sets of data, for each defined comparison alignment, three possible Δδ_calc_ and Δδ_exp_ sets of values can be computed after defining the combination pairs (Figure 2). For instance, considering the ABC/**123** calculated/experimental comparison alignments, the following Δδ_calc_ and Δδ_exp_ sets of values can be defined for the subsequent comparison
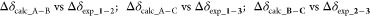


In general, starting from the number of calculated/experimental sets of data (*n*), for each defined comparison alignment, the related number of calculated/experimental Δδ sets (*N*_Δδ_) to be accounted considering all possible pairs can be computed with the following relation (eq 2)
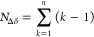
2

Thus, for each of the 24 comparison alignments arising from 4 sets of experimental/calculated data (eq 1), 6 possible Δδ_calc_ and Δδ_exp_ sets of values can be computed after defining the related combination pairs (eq 2).

It is important to note that, following this procedure, the calculation of the differences between calculated chemical shift data (Δδ_calc_) allows to avoid all systematic errors arising from calibration procedures required for computing the chemical shift data from shielding the tensor values [using trimethylsilane (TMS) as the reference].

(c) Following the comparison alignments, the specific Δδ_calc_ and corresponding Δδ_exp_ group of values are then compared atom by atom using the ΔΔδ parameter
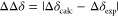
defined as the absolute difference between the Δδ_calc_ and Δδ_exp_ for each accounted atom.

In this way, the obtained ΔΔδ differences are employed for detecting the similarities between calculated and experimental sets of data and then for identifying the most promising comparison alignment among all possibilities. Indeed, all computed ΔΔδ values can be easily converted into a parameter that quickly indicates the best comparison alignment among all possibilities. In this study, we have defined the MAE_ΔΔδ_ parameter
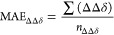
defined as the summation (∑) of the *n* computed Δδ absolute error values (ΔΔδ) normalized to the number of ΔΔδ errors considered (*n*_ΔΔδ_).

Summarizing, for each possible experimental/calculated comparison alignment, the related MAE_ΔΔδ_ value can be computed; finally, the lowest MAE_ΔΔδ_ value among the ranking indicated the best fit between each experimental and calculated set of data.

### Applying the Methodology on Cladosporine and Pochonicine Stereoisomers

The reported workflow was then applied to the four investigated cladologs (**1a–1d**). In this case, with 4 available sets of experimental/calculated data, 24 possible comparison alignments were taken into account (eq 1) for generating the related MAE_ΔΔδ_ values (Table 2). The calculated sets of data arising from the different combinations of functional/basis sets above reported were accounted (Table 2) in order to evaluate the applicability of the proposed procedure and to compare the results with those previously obtained. For each employed level of theory, the analysis of the data indicated that the lowest MAE_ΔΔδ_ value obtained among the ranking of 24 possibilities was that related to the calc_**1a** calc_**1b** calc_**1c** calc_**1d**/exp_**1a** exp_**1b** exp_**1c** exp_**1d** comparison alignment (Table 2). On the other hand, we also computed, for each of the 24 comparison alignments, the average of the 4 possible MAE values obtained from the comparison of the calculated and experimental chemical shifts (see Table S13) instead of comparing the differences of the shifts, as proposed by us.

**Table 2:**
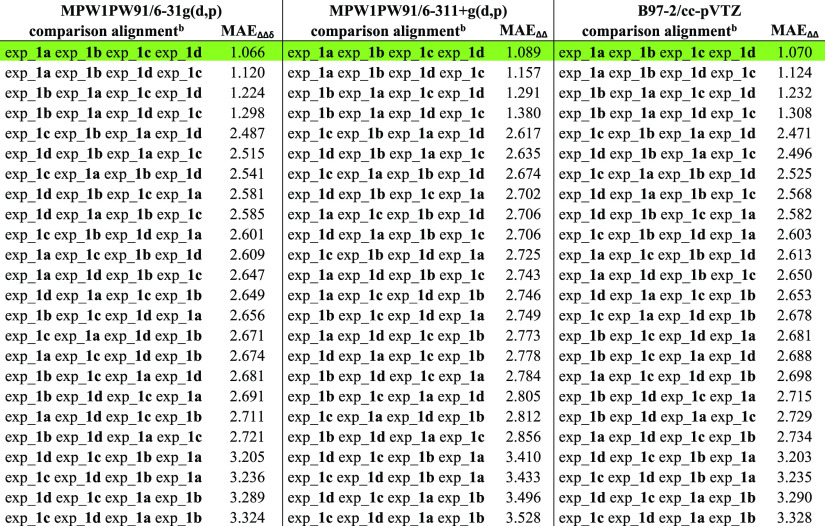
^13^C NMR MAE_ΔΔδ_ Values Related to the Accounted Comparison Alignments Considering calc_**1a** calc_**1b** calc_**1c** calc_**1d** Fixed Sequence and All Possible 24 Combinations Considering exp_**1a**, exp_**1b**, exp_**1c**, and exp_**1d** Sets of Data^a^

a The correct comparison alignments are highlighted in green, showing their top-ranked positions also accounting different functional/basis set combinations.

b considering calc_**1a** calc_**1b** calc_**1c** and calc_**1d** starting fixed sequence related to the calculated sets of data.

In this case, we again obtained the correct comparison alignment, but the comparison with the MAE_ΔΔδ_ data pointed out for the latter more discrete values and better discriminating power in identifying the correct correspondences between the data sets (see Table S13). These results strongly confirmed the applicability of the proposed methodology, highlighting with a high level of confidence the correct stereochemical assignment of groups of stereoisomers.

**Chart 2:**
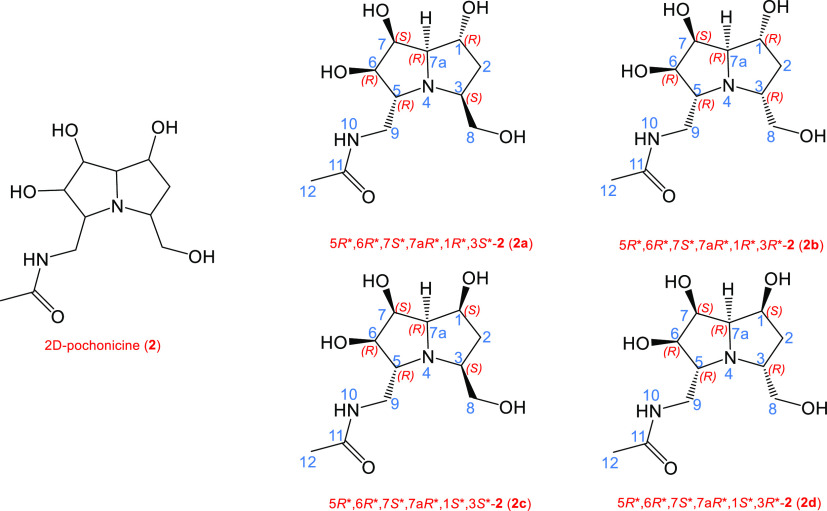
Chemical Structures of the Four Accounted Pochonicine-Related Stereoisomers (**2a–2d**)

In order to further corroborate the proposed approach, we investigated another tetrad of stereoisomers related to pochonicine, a naturally occurring polyhydroxylated pyrrolizidine from *Pochonia suchlasporia* var. *suchlasporia* TAMA 87. In 2013, Yu et al. reported the synthesis of eight stereoisomers of pochonicine^27^ and, in this study, we accounted the four stereoisomers with different relative configurations at C-1 and C-3 while maintaining the 5R*,6R*,7S*,7aR* configurations. In Chart 2, the four accounted stereoisomers related to pochonicine (**2a–2d**) are depicted.

Following the same scheme above reported for cladologs, we named the sets of calculated data for **2a–2d** as calc_**2a**, calc_**2b**, calc_**2c**, and calc_**2d**, respectively, and the sets of experimental data, reported in the reference study^27^ (corroborated by comparison with further studies on pochonicine^28,29^), were named for **2a–2d** as exp_**2a**, exp_**2b**, exp_**2c**, and exp_**2d**, respectively.

Again, employing the “classic” QM/NMR approach, the correct correspondences between the four calculated and experimental sets of data were not found considering the three functional/basis set combinations (Tables 3 and S14–S25). Conversely, the computation of the 24 MAE_ΔΔδ_ values related to the comparison alignments considering calc_**2a** calc_**2b** calc_**2c** calc_**2d** sequence highlighted exp_**2a**, exp_**2b**, exp_**2c**, and exp_**2d** as the solution showing the lowest MAE_ΔΔδ_ values for all three functional/basis sets employed (Table 4), thus confirming the applicability of the proposed approach.

**Table 3:**
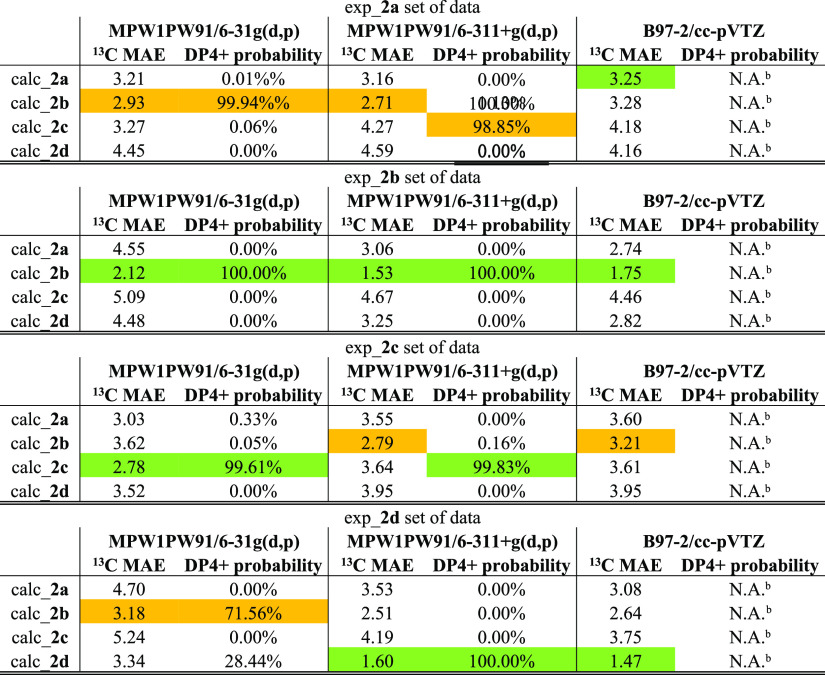
^13^C NMR MAE Values and DP4+ Probabilities Computed for the Three Functional/Basis Set Combinations Accounted in This Study Related to Compounds **2a–2d**^a^

a The correct/incorrect correspondence between experimental and calculated data are highlighted in green and orange, respectively.

b B97-2/cc-pVTZ functional/basis set combination cannot be set in the calculation of DP4+ probability.

**Table 4:**
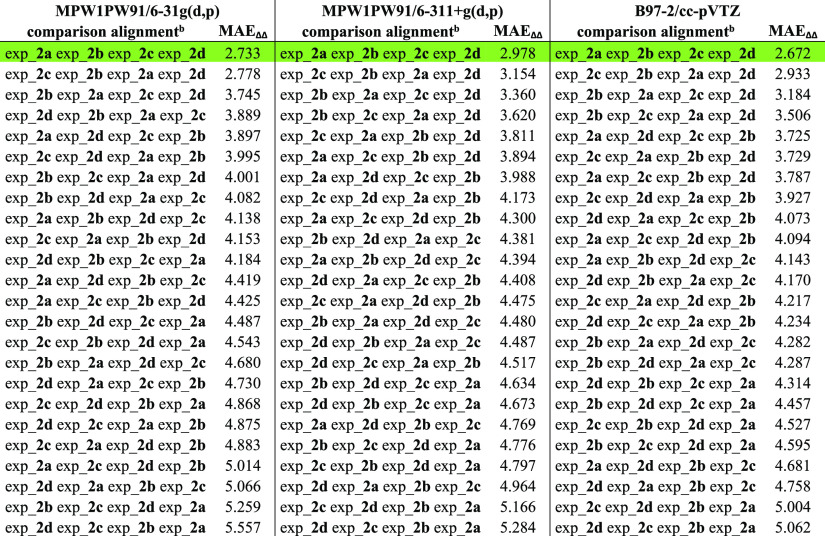
^13^C NMR MAE_ΔΔδ_ Values Related to the Accounted Comparison Alignments Considering calc_**2a** calc_**2b** calc_**2c** calc_**2d** Fixed Sequence and all the Possible 24 Combinations Considering exp_**2a**, exp_**2b**, exp_**2c**, and exp_**2d** Sets of Data^a^

a The correct comparison alignments are highlighted in green, showing their top-ranked positions also accounting different functional/basis set combinations.

b Considering calc_**2a** calc_**2b** calc_**2c** and calc_**2d** starting fixed sequence related to the calculated sets of data.

## Conclusions

In this study, we introduced an approach guiding the correct assignment of groups of stereoisomers. This methodology is based on building all possible comparison alignments between a fixed sequence from the QM/NMR calculated sets of data and all possible sequences arising from the combinations of the experimental sets of data. For each comparison alignment, the MAE_ΔΔδ_ value is computed, generating a final ranking from the lowest to the highest value. Accordingly, the comparison alignment featuring the lowest MAE_ΔΔδ_ value indicates the best fit between each calculated and experimental set of value, facilitating the assignment of groups of stereoisomers. We validated this approach accounting four stereoisomers of cladosporin (cladologs) and pochonicine, showing the correct assignment of each set of experimental data to the specific stereoisomer. The present approach is not limited by the number of stereoisomers to be accounted, thus representing a valuable tool for solving specific stereochemical issues. Moreover, we inserted a dedicated tab on the website of our research group (https://computorgchem.unisa.it) containing a tool for the straightforward MAE_ΔΔδ_ computation starting from calculated and experimental data sets as input files.

## Experimental Section

### Experimental ^13^C NMR Data

All experimental ^13^C NMR chemical shift data related to compounds **1a–1d** and **2a–2d** were retrieved from the related reference papers,^19−21,27−29^ as reported above.

### Computation of NMR Parameters

Three-dimensional starting models of compounds **1a–1d** and **2a–2d** were built by Maestro 10.2^30^ and optimized by MacroModel 10.2^31^ with the OPLS force field^32^ and the Polak-Ribier conjugate gradient algorithm (maximum derivative less than 0.001 kcal/mol). Conformational search rounds for the above-mentioned compounds were performed using MacroModel 10.2^30,31^ on the empirical MM level. Specifically, Monte Carlo multiple minimum and low mode conformational search methods were first employed in order to explore the conformational space. Furthermore, rounds of molecular dynamics simulations were performed at 450, 600, 700, and 750 K, with a time step of 2.0 fs, an equilibration time of 0.1 ns, and a simulation time of 10 ns. All produced conformers were then collected and analyzed in order to discard the redundant ones. Specifically, the nonredundant conformers were selected by using the “redundant conformer elimination” module of Macromodel 10.2^30^ excluding those differing more than 12.5 kJ/mol (3.0 kcal/mol) from the most energetically favored conformation and setting a 0.1 Å RMSD minimum cutoff for saving structures. The following reported QM calculations were performed using Gaussian 09 software.^33^

The obtained conformers were geometry optimized on the QM level by using the MPW1PW91 functional and the 6-31G(d) basis set. After this step, the new geometries were visually inspected in order to filter out further possible redundant conformers. Finally, the obtained conformers were accounted for the subsequent computation of the ^13^C NMR chemical shifts using the MPW1PW91/6-31G(d,p), MPW1PW91/6-311+G(d,p), B97-2/cc-pVTZ functionals/basis set combinations (see Results and Discussion and Tables S1–S12, S14–S25, Supporting Information). The final ^13^C NMR chemical shift data were computed considering the influence of each conformer on the total Boltzmann distribution and taking into account the relative energies. Calibrations of calculated ^13^C chemical shifts were performed following the multistandard approach.^34,35^ Benzene was used as the reference compound for computing sp^2^^13^C NMR chemical shifts (excluding carbonyl carbons) in detail,^34,35^ whereas TMS was used for computing sp^3^^13^C chemical shift data.

The comparison of calculated and experimental data^19^ was performed accounting Δδ, Δδ_calc_, Δδ_exp_, ΔΔδ, MAE, and MAE_ΔΔδ_ parameters, as reported in the Results and Discussion section.

## Supporting Information Available

Calculated NMR chemical shifts with cartesian coordinates of the optimized geometries and related energies for the three employed functional/basis set combinations for the conformers of **1a–1d** and **2a–2d** (PDF)
