# Supplementary material for: DFT/NMR Approach for the Configuration Assignment of Groups of Stereoisomers by the Combination and Comparison of Experimental and Predicted Sets of Data
Source: J Org Chem. 2020 Jan 21;85(5):3297–306. doi: 10.1021/acs.joc.9b03129 (PMC7997581; doi:10.1021/acs.joc.9b03129)
Supplement: Supplementary file 1 — jo9b03129_si_001.pdf [file jo9b03129_si_001.pdf]

Supporting Information

DFT/NMR Approach for the Configuration Assignment of Groups of Stereoisomers by the Combination and Comparison of Experimental and Predicted Sets of Data

Gianluigi Lauro,[a] Pronay Das,[b,c] Raffaele Riccio,[a] D. Srinivasa Reddy,[b,c] and Giuseppe Bifulco*,[a]

[a]Department of Pharmacy, University of Salerno, Via Giovanni Paolo II 132, Fisciano 84084, Italy

[b]Organic Chemistry Division, CSIR-National Chemical Laboratory, Dr. Homi Bhabha Road, Pune, 411008, India.

[c]Academy of Scientific and Innovative Research (AcSIR), New Delhi, 110025, India

*To whom correspondence should be addressed: bifulco@unisa.it, Telephone: +39 (0)89969741, Fax: +39 (0)89969602

Table of contents

Table S1. ^{13}C experimental related to exp_**1a** set of data and calculated NMR chemical shifts (MPW1PW91/6-31g(d,p) level of theory) related to calc_**1a**, calc_**1b**, calc_**1c**, calc_**1d** sets of data, with |Δδ|(^{13}C) and MAE values S6

Table S2. ^{13}C experimental related to exp_**1b** set of data and calculated NMR chemical shifts (MPW1PW91/6-31g(d,p) level of theory) related to calc_**1a**, calc_**1b**, calc_**1c**, calc_**1d** sets of data, with |Δδ|(^{13}C) and MAE values........ S**Error! Bookmark not defined.**

Table S3. ^{13}C experimental related to exp_**1c** set of data and calculated NMR chemical shifts (MPW1PW91/6-31g(d,p) level of theory) related to calc_**1a**, calc_**1b**, calc_**1c**, calc_**1d** sets of data, with |Δδ|(^{13}C) and MAE values........ S8

Table S4. ^{13}C experimental related to exp_**1d** set of data and calculated NMR chemical shifts (MPW1PW91/6-31g(d,p) level of theory) related to calc_**1a**, calc_**1b**, calc_**1c**, calc_**1d** sets of data, with |Δδ|(^{13}C) and MAE values........ S9

Table S5. ^{13}C experimental related to exp_**1a** set of data and calculated NMR chemical shifts (MPW1PW91/6-311+g(d,p) level of theory) related to calc_**1a**, calc_**1b**, calc_**1c**, calc_**1d** sets of data, with |Δδ|(^{13}C) and MAE values S10

Table S6. ^{13}C experimental related to exp_**1b** set of data and calculated NMR chemical shifts (MPW1PW91/6-311+g(d,p) level of theory) related to calc_**1a**, calc_**1b**, calc_**1c**, calc_**1d** sets of data, with |Δδ|(^{13}C) and MAE values........ S11

Table S7. ^{13}C experimental related to exp_**1c** set of data and calculated NMR chemical shifts (MPW1PW91/6-311+g(d,p) level of theory) related to calc_**1a**, calc_**1b**, calc_**1c**, calc_**1d** sets of data, with |Δδ|(^{13}C) and MAE values........ S12

Table S8. ^{13}C experimental related to exp_**1d** set of data and calculated NMR chemical shifts (MPW1PW91/6-311+g(d,p) level of theory) related to calc_**1a**, calc_**1b**, calc_**1c**, calc_**1d** sets of data, with |Δδ|(^{13}C) and MAE values........ S13

Table S9. ^{13}C experimental related to exp_**1a** set of data and calculated NMR chemical shifts (B97-2/cc-pVTZ level of theory) related to calc_**1a**, calc_**1b**, calc_**1c**, calc_**1d** sets of data, with |Δδ|(^{13}C) and MAE values S14

Table S10. ^{13}C experimental related to exp_**1b** set of data and calculated NMR chemical shifts (B97-2/cc-pVTZ level of theory) related to calc_**1a**, calc_**1b**, calc_**1c**, calc_**1d** sets of data, with |Δδ|(^{13}C) and MAE values........ S15

Table S11. ^{13}C experimental related to exp_**1c** set of data and calculated NMR chemical shifts (B97-2/cc-pVTZ level of theory) related to calc_**1a**, calc_**1b**, calc_**1c**, calc_**1d** sets of data, with |Δδ|(^{13}C) and MAE values S16
Table S12. ^{13}C experimental related to exp_**1d** set of data and calculated NMR chemical shifts (B97-2/cc-pVTZ level of theory) related to calc_**1a**, calc_**1b**, calc_**1c**, calc_**1d** sets of data, with |Δδ|(^{13}C) and MAE values S17
Table S13. Comparison of the results obtained by computing the $MAE_{\Delta\Delta\delta}$ values (bottom) and the AVER_MAE values (top), the latter defined as the average of the four possible MAE values obtained from the comparison of the calculated and experimental chemical shifts, for each of the 24 comparison alignments. S18
Table S14. ^{13}C experimental related to exp_**2a** set of data and calculated NMR chemical shifts (MPW1PW91/6-31g(d,p) level of theory) related to calc_**2a**, calc_**2b**, calc_**2c**, calc_**2d** sets of data, with |Δδ|(^{13}C) and MAE values S20
Table S15. ^{13}C experimental related to exp_**2b** set of data and calculated NMR chemical shifts (MPW1PW91/6-31g(d,p) level of theory) related to calc_**2a**, calc_**2b**, calc_**2c**, calc_**2d** sets of data, with |Δδ|(^{13}C) and MAE values S21
Table S16. ^{13}C experimental related to exp_**2c** set of data and calculated NMR chemical shifts (MPW1PW91/6-31g(d,p) level of theory) related to calc_**2a**, calc_**2b**, calc_**2c**, calc_**2d** sets of data, with |Δδ|(^{13}C) and MAE values S22
Table S17. ^{13}C experimental related to exp_**2d** set of data and calculated NMR chemical shifts (MPW1PW91/6-31g(d,p) level of theory) related to calc_**2a**, calc_**2b**, calc_**2c**, calc_**2d** sets of data, with |Δδ|(^{13}C) and MAE values S23
Table S18. ^{13}C experimental related to exp_**2a** set of data and calculated NMR chemical shifts (MPW1PW91/6-311+g(d,p) level of theory) related to calc_**2a**, calc_**2b**, calc_**2c**, calc_**2d** sets of data, with |Δδ|(^{13}C) and MAE values S24
Table S19. ^{13}C experimental related to exp_**2b** set of data and calculated NMR chemical shifts (MPW1PW91/6-311+g(d,p) level of theory) related to calc_**2a**, calc_**2b**, calc_**2c**, calc_**2d** sets of data, with |Δδ|(^{13}C) and MAE values S25
Table S20. ^{13}C experimental related to exp_**2c** set of data and calculated NMR chemical shifts (MPW1PW91/6-311+g(d,p) level of theory) related to calc_**2a**, calc_**2b**, calc_**2c**, calc_**2d** sets of data, with |Δδ|(^{13}C) and MAE values S26
Table S21. ^{13}C experimental related to exp_**2d** set of data and calculated NMR chemical shifts (MPW1PW91/6-311+g(d,p) level of theory) related to calc_**2a**, calc_**2b**, calc_**2c**, calc_**2d** sets of data, with |Δδ|(^{13}C) and MAE values S27

Table S22. ^{13}C experimental related to exp_**2a** set of data and calculated NMR chemical shifts (B97-2/cc-pVTZ level of theory) related to calc_**2a**, calc_**2b**, calc_**2c**, calc_**2d** sets of data, with |Δδ|(^{13}C) and MAE values S28
Table S23. ^{13}C experimental related to exp_**2b** set of data and calculated NMR chemical shifts (B97-2/cc-pVTZ level of theory) related to calc_**2a**, calc_**2b**, calc_**2c**, calc_**2d** sets of data, with |Δδ|(^{13}C) and MAE values.. S29
Table S24. ^{13}C experimental related to exp_**2c** set of data and calculated NMR chemical shifts (B97-2/cc-pVTZ level of theory) related to calc_**2a**, calc_**2b**, calc_**2c**, calc_**2d** sets of data, with |Δδ|(^{13}C) and MAE values.. S30
Table S25. ^{13}C experimental related to exp_**2d** set of data and calculated NMR chemical shifts (B97-2/cc-pVTZ level of theory) related to calc_**2a**, calc_**2b**, calc_**2c**, calc_**2d** sets of data, with |Δδ|(^{13}C) and MAE values.. S31
Table S26. Sampled and DFT geometry optimized conformers related to calc_**1a**, with energy values (Hartree) and related % contribution on the final Boltzmann distribution for the three employed functional/basis set combinations S32
Table S27. Cartesian coordinates of the optimized geometries for the conformers related to calc_**1a**. The related energies and % contribution on the final Boltzmann distribution for the three employed functional/basis set combinations are reported in Table S26.. S33
Table S28. Sampled and DFT geometry optimized conformers related to calc_**1b**, with energy values (Hartree) and related % contribution on the final Boltzmann distribution for the three employed functional/basis set combinations S54
Table S29. Cartesian coordinates of the optimized geometries for the conformers related to calc_**1b**. The related energies and % contribution on the final Boltzmann distribution for the three employed functional/basis set combinations are reported in Table S28.. S56
Table S30. Sampled and DFT geometry optimized conformers related to calc_**1c**, with energy values (Hartree) and related % contribution on the final Boltzmann distribution for the three employed functional/basis set combinations S81
Table S31. Cartesian coordinates of the optimized geometries for the conformers related to calc_**1c**. The related energies and % contribution on the final Boltzmann distribution for the three employed functional/basis set combinations are reported in Table S30.. S82
Table S32. Sampled and DFT geometry optimized conformers related to calc_**1d**, with energy values (Hartree) and related % contribution on the final Boltzmann distribution for the three employed functional/basis set combinations S97

Table S33. Cartesian coordinates of the optimized geometries for the conformers related to calc_**1d**. The related energies and % contribution on the final Boltzmann distribution for the three employed functional/basis set combinations are reported in Table S32 .... S98
Table S34. Sampled and DFT geometry optimized conformers related to calc_**2a**, with energy values (Hartree) and related % contribution on the final Boltzmann distribution for the three employed functional/basis set combinations S115
Table S35. Cartesian coordinates of the optimized geometries for the conformers related to calc_**2a**. The related energies and % contribution on the final Boltzmann distribution for the three employed functional/basis set combinations are reported in Table S34 .... S116
Table S36. Sampled and DFT geometry optimized conformers related to calc_**2b**, with energy values (Hartree) and related % contribution on the final Boltzmann distribution for the three employed functional/basis set combinations S127
Table S37. Cartesian coordinates of the optimized geometries for the conformers related to calc_**2b**. The related energies and % contribution on the final Boltzmann distribution for the three employed functional/basis set combinations are reported in Table S36 .... S128
Table S38. Sampled and DFT geometry optimized conformers related to calc_**2c**, with energy values (Hartree) and related % contribution on the final Boltzmann distribution for the three employed functional/basis set combinations S145
Table S39. Cartesian coordinates of the optimized geometries for the conformers related to calc_**2c**. The related energies and % contribution on the final Boltzmann distribution for the three employed functional/basis set combinations are reported in Table S38 .... S147
Table S40. Sampled and DFT geometry optimized conformers related to calc_**2d**, with energy values (Hartree) and related % contribution on the final Boltzmann distribution for the three employed functional/basis set combinations S184
Table S41. Cartesian coordinates of the optimized geometries for the conformers related to calc_**2d**. The related energies and % contribution on the final Boltzmann distribution for the three employed functional/basis set combinations are reported in Table S40 .... S186

Table S1. ^{13}C experimental related to exp_**1a** set of data and calculated NMR chemical shifts (MPW1PW91/6-31g(d,p) level of theory) related to calc_**1a**, calc_**1b**, calc_**1c**, calc_**1d** sets of data, with $^{a}|\Delta\delta|(^{13}C)$ and ^{b}MAE values. Chemical shift data here reported were produced using benzene as reference compound for sp^2 carbons (highlighted in yellow), and tetramethylsilane (TMS) for sp^3 carbons.

	$\delta_{exp}(^{13}C)$, ppm	$\delta_{calc}(^{13}C)$, ppm				$\lvert\Delta\delta\rvert$ (^{13}C), ppm^a			
**Position**	exp_**1a**	calc_**1a**	calc_**1b**	calc_**1c**	calc_**1d**	calc_**1a**	calc_**1b**	calc_**1c**	calc_**1d**
1	169.9	163.65	163.79	163.82	163.62	6.25	6.11	6.08	6.29
3	76.3	72.81	73.56	73.37	72.48	3.49	2.74	2.93	3.82
4	33.6	34.93	33.52	33.73	35.02	1.33	0.08	0.13	1.42
4a	141.8	143.10	143.24	143.31	143.05	1.30	1.44	1.51	1.25
5	106.7	105.75	105.80	105.76	105.75	0.95	0.90	0.94	0.95
6	163.1	160.89	160.91	160.89	160.92	2.21	2.19	2.21	2.18
7	102.0	101.15	101.10	101.10	101.15	0.85	0.90	0.90	0.85
8	164.3	165.73	165.74	165.75	165.76	1.43	1.44	1.45	1.46
8a	101.5	104.34	104.27	104.25	104.32	2.84	2.77	2.75	2.82
9	39.3	40.76	38.21	40.74	42.54	1.46	1.09	1.44	3.24
10	66.6	64.23	65.24	71.87	71.21	2.37	1.36	5.27	4.61
11	30.9	31.53	30.46	30.94	31.66	0.63	0.44	0.04	0.76
12	18.1	20.24	20.19	24.80	24.74	2.14	2.09	6.70	6.64
13	30.9	30.21	30.54	32.33	32.55	0.69	0.36	1.43	1.65
14	68.0	66.65	66.18	71.98	71.73	1.35	1.82	3.98	3.73
15	18.9	18.45	19.27	22.82	22.76	0.45	0.37	3.92	3.86
MAE^b						1.86	1.63	2.61	2.85

^{a} $\mathbf{\lvert\Delta\delta\rvert(^{13}C)}$ = $\lvert\delta_{exp} - \delta_{calc}\rvert$ (^{13}C), ppm: absolute differences for experimental versus calculated ^{13}C NMR chemical shifts
^{b} **MAE** = $\Sigma[\lvert(\delta_{exp} - \delta_{calcd})\rvert]/n$, summation through n of the absolute error values (difference of the absolute values between corresponding experimental and ^{13}C chemical shifts), normalized to the number of the chemical shifts

Table S2. ^{13}C experimental related to exp_**1b** set of data and calculated NMR chemical shifts (MPW1PW91/6-31g(d,p) level of theory) related to calc_**1a**, calc_**1b**, calc_**1c**, calc_**1d** sets of data, with [a]|Δδ|(^{13}C) and [b]MAE values. Chemical shift data here reported were produced using benzene as reference compound for sp^2 carbons (highlighted in yellow), and tetramethylsilane (TMS) for sp^3 carbons.

	δ_{exp}(^{13}C), ppm	δ_{calc}(^{13}C), ppm				|Δδ| (^{13}C), ppm[a]			
**Position**	exp_**1b**	calc_**1a**	calc_**1b**	calc_**1c**	calc_**1d**	calc_**1a**	calc_**1b**	calc_**1c**	calc_**1d**
1	170.0	163.65	163.79	163.82	163.62	6.35	6.21	6.18	6.38
3	76.6	72.81	73.56	73.37	72.48	3.79	3.04	3.23	4.12
4	32.6	34.93	33.52	33.73	35.02	2.33	0.92	1.13	2.42
4a	141.6	143.10	143.24	143.31	143.05	1.50	1.64	1.71	1.45
5	106.7	105.75	105.80	105.76	105.75	0.95	0.90	0.94	0.95
6	163.0	160.89	160.91	160.89	160.92	2.11	2.09	2.11	2.08
7	102.0	101.15	101.10	101.10	101.15	0.85	0.90	0.90	0.85
8	164.4	165.73	165.74	165.75	165.76	1.33	1.34	1.35	1.36
8a	101.6	104.34	104.27	104.25	104.32	2.74	2.67	2.65	2.72
9	37.3	40.76	38.21	40.74	42.54	3.46	0.91	3.44	5.24
10	67.2	64.23	65.24	71.87	71.21	2.97	1.96	4.67	4.01
11	29.8	31.53	30.46	30.94	31.66	1.73	0.66	1.14	1.86
12	18.1	20.24	20.19	24.80	24.74	2.14	2.09	6.70	6.64
13	31.5	30.21	30.54	32.33	32.55	1.29	0.96	0.83	1.05
14	67.4	66.65	66.18	71.98	71.73	0.75	1.22	4.58	4.33
15	19.7	18.45	19.27	22.82	22.76	1.25	0.43	3.12	3.06
MAE[b]						2.22	1.75	2.79	3.03

[a] **|Δδ|(^{13}C)** = $|\delta_{exp} - \delta_{calc}|$ (^{13}C), ppm: absolute differences for experimental versus calculated ^{13}C NMR chemical shifts
[b] **MAE** = $\Sigma[|(\delta_{exp} - \delta_{calcd})|]/n$, summation through n of the absolute error values (difference of the absolute values between corresponding experimental and ^{13}C chemical shifts), normalized to the number of the chemical shifts

Table S3. ^{13}C experimental related to exp_**1c** set of data and calculated NMR chemical shifts (MPW1PW91/6-31g(d,p) level of theory) related to calc_**1a**, calc_**1b**, calc_**1c**, calc_**1d** sets of data, with [a]|Δδ|(^{13}C) and [b]MAE values. Chemical shift data here reported were produced using benzene as reference compound for sp^2 carbons (highlighted in yellow), and tetramethylsilane (TMS) for sp^3 carbons.

	δ_{exp}(^{13}C), ppm	δ_{calc}(^{13}C), ppm				|Δδ| (^{13}C), ppm[a]			
**Position**	exp_**1c**	calc_**1a**	calc_**1b**	calc_**1c**	calc_**1d**	calc_**1a**	calc_**1b**	calc_**1c**	calc_**1d**
1	171.7	163.65	163.79	163.82	163.62	8.05	7.91	7.88	8.08
3	78.0	72.81	73.56	73.37	72.48	5.19	4.44	4.63	5.52
4	34.5	34.93	33.52	33.73	35.02	0.43	0.98	0.77	0.52
4a	143.6	143.10	143.24	143.31	143.05	0.50	0.36	0.29	0.55
5	108.1	105.75	105.80	105.76	105.75	2.35	2.30	2.34	2.35
6	165.8	160.89	160.91	160.89	160.92	4.91	4.89	4.91	4.88
7	102.3	101.15	101.10	101.10	101.15	1.15	1.20	1.20	1.15
8	166.4	165.73	165.74	165.75	165.76	0.67	0.66	0.65	0.64
8a	101.8	104.34	104.27	104.25	104.32	2.54	2.47	2.45	2.52
9	42.2	40.76	38.21	40.74	42.54	1.44	3.99	1.46	0.34
10	75.2	64.23	65.24	71.87	71.21	10.97	9.96	3.33	3.99
11	32.5	31.53	30.46	30.94	31.66	0.97	2.04	1.56	0.84
12	22.6	20.24	20.19	24.80	24.74	2.36	2.41	2.20	2.14
13	33.8	30.21	30.54	32.33	32.55	3.59	3.26	1.47	1.25
14	75.5	66.65	66.18	71.98	71.73	8.85	9.32	3.52	3.77
15	24.7	18.45	19.27	22.82	22.76	6.25	5.43	1.88	1.94
MAE[b]						3.76	3.85	2.53	2.53

[a] **|Δδ|(^{13}C)** = $|\delta_{exp} - \delta_{calc}|$ (^{13}C), ppm: absolute differences for experimental versus calculated ^{13}C NMR chemical shifts
[b] **MAE** = $\Sigma[|(\delta_{exp} - \delta_{calcd})|]/n$, summation through n of the absolute error values (difference of the absolute values between corresponding experimental and ^{13}C chemical shifts), normalized to the number of the chemical shifts

Table S4. ^{13}C experimental related to exp_**1d** set of data and calculated NMR chemical shifts (MPW1PW91/6-31g(d,p) level of theory) related to calc_**1a**, calc_**1b**, calc_**1c**, calc_**1d** sets of data, with $^{a}|\Delta\delta|(^{13}C)$ and ^{b}MAE values. Chemical shift data here reported were produced using benzene as reference compound for sp^2 carbons (highlighted in yellow), and tetramethylsilane (TMS) for sp^3 carbons.

	$\delta_{exp}(^{13}C)$, ppm	$\delta_{calc}(^{13}C)$, ppm				$\lvert\Delta\delta\rvert$ (^{13}C), ppm^a			
**Position**	exp_**1d**	calc_**1a**	calc_**1b**	calc_**1c**	calc_**1d**	calc_**1a**	calc_**1b**	calc_**1c**	calc_**1d**
1	171.7	163.65	163.79	163.82	163.62	8.05	7.91	7.88	8.08
3	77.6	72.81	73.56	73.37	72.48	4.79	4.04	4.23	5.12
4	34.6	34.93	33.52	33.73	35.02	0.33	1.08	0.87	0.42
4a	143.7	143.10	143.24	143.31	143.05	0.60	0.46	0.39	0.65
5	108.0	105.75	105.80	105.76	105.75	2.25	2.20	2.24	2.25
6	165.8	160.89	160.91	160.89	160.92	4.91	4.89	4.91	4.88
7	102.3	101.15	101.10	101.10	101.15	1.15	1.20	1.20	1.15
8	166.4	165.73	165.74	165.75	165.76	0.67	0.66	0.65	0.64
8a	101.7	104.34	104.27	104.25	104.32	2.64	2.57	2.55	2.62
9	43.0	40.76	38.21	40.74	42.54	2.24	4.79	2.26	0.46
10	74.8	64.23	65.24	71.87	71.21	10.57	9.56	2.93	3.59
11	33.1	31.53	30.46	30.94	31.66	1.57	2.64	2.16	1.44
12	22.5	20.24	20.19	24.80	24.74	2.26	2.31	2.30	2.24
13	34.5	30.21	30.54	32.33	32.55	4.29	3.96	2.17	1.95
14	75.3	66.65	66.18	71.98	71.73	8.65	9.12	3.32	3.57
15	24.8	18.45	19.27	22.82	22.76	6.35	5.53	1.98	2.04
MAE^b						3.83	3.93	2.63	2.57

^{a} **$\lvert\Delta\delta\rvert(^{13}C)$** = $\lvert\delta_{exp} - \delta_{calc}\rvert$ (^{13}C), ppm: absolute differences for experimental versus calculated ^{13}C NMR chemical shifts
^{b} **MAE** = $\Sigma[\lvert(\delta_{exp} - \delta_{calcd})\rvert]/n$, summation through n of the absolute error values (difference of the absolute values between corresponding experimental and ^{13}C chemical shifts), normalized to the number of the chemical shifts

Table S5. ^{13}C experimental related to exp_**1a** set of data and calculated NMR chemical shifts (MPW1PW91/6-311+g(d,p) level of theory) related to calc_**1a**, calc_**1b**, calc_**1c**, calc_**1d** sets of data, with $^{a}|\Delta\delta|(^{13}C)$ and ^{b}MAE values. Chemical shift data here reported were produced using benzene as reference compound for sp^2 carbons (highlighted in yellow), and tetramethylsilane (TMS) for sp^3 carbons.

	$\delta_{exp}(^{13}C)$, ppm	$\delta_{calc}(^{13}C)$, ppm				$|\Delta\delta|$ (^{13}C), ppm[a]			
**Position**	exp_**1a**	calc_**1a**	calc_**1b**	calc_**1c**	calc_**1d**	calc_**1a**	calc_**1b**	calc_**1c**	calc_**1d**
1	169.9	174.92	174.85	174.99	174.84	5.02	4.95	5.09	4.94
3	76.3	76.11	77.30	77.23	75.96	0.19	1.00	0.93	0.34
4	33.6	37.32	35.68	35.96	37.41	3.72	2.08	2.36	3.81
4a	141.8	143.90	144.02	144.05	143.74	2.10	2.22	2.25	1.94
5	106.7	104.22	103.73	103.70	104.59	2.48	2.97	3.00	2.11
6	163.1	163.20	163.66	163.57	163.48	0.10	0.56	0.47	0.38
7	102.0	98.44	98.67	98.64	98.83	3.56	3.33	3.36	3.17
8	164.3	167.21	167.47	167.47	167.13	2.91	3.17	3.17	2.83
8a	101.5	100.45	100.42	100.43	100.19	1.05	1.08	1.07	1.31
9	39.3	43.97	40.97	43.72	45.32	4.67	1.67	4.42	6.02
10	66.6	66.34	68.35	75.57	74.23	0.26	1.75	8.97	7.63
11	30.9	34.67	33.04	33.68	34.59	3.77	2.14	2.78	3.69
12	18.1	22.22	22.10	27.49	27.31	4.12	4.00	9.39	9.21
13	30.9	33.13	33.71	35.67	35.97	2.23	2.81	4.77	5.07
14	68.0	70.78	70.14	76.32	75.88	2.78	2.14	8.32	7.88
15	18.9	18.69	20.13	23.93	23.97	0.21	1.23	5.03	5.07
MAE[b]						2.45	2.32	4.09	4.09

[a] $|\Delta\delta|(^{13}C) = |\delta_{exp} - \delta_{calc}|$ (^{13}C), ppm: absolute differences for experimental versus calculated ^{13}C NMR chemical shifts
[b] **MAE** = $\Sigma[|(\delta_{exp} - \delta_{calcd})|]/n$, summation through n of the absolute error values (difference of the absolute values between corresponding experimental and ^{13}C chemical shifts), normalized to the number of the chemical shifts

Table S6. ^{13}C experimental related to exp_**1b** set of data and calculated NMR chemical shifts (MPW1PW91/6-311+g(d,p) level of theory) related to calc_**1a**, calc_**1b**, calc_**1c**, calc_**1d** sets of data, with [a]$|\Delta\delta|(^{13}C)$ and [b]MAE values. Chemical shift data here reported were produced using benzene as reference compound for sp^2 carbons (highlighted in yellow), and tetramethylsilane (TMS) for sp^3 carbons.

	$\delta_{exp}(^{13}C)$, ppm	$\delta_{calc}(^{13}C)$, ppm				$\lvert\Delta\delta\rvert$ (^{13}C), ppm[a]			
**Position**	exp_**1b**	calc_**1a**	calc_**1b**	calc_**1c**	calc_**1d**	calc_**1a**	calc_**1b**	calc_**1c**	calc_**1d**
1	170.0	174.92	174.85	174.99	174.84	4.92	4.85	4.99	4.84
3	76.6	76.11	77.30	77.23	75.96	0.49	0.70	0.63	0.64
4	32.6	37.32	35.68	35.96	37.41	4.72	3.08	3.36	4.81
4a	141.6	143.90	144.02	144.05	143.74	2.30	2.42	2.45	2.14
5	106.7	104.22	103.73	103.70	104.59	2.48	2.97	3.00	2.11
6	163.0	163.20	163.66	163.57	163.48	0.20	0.66	0.57	0.48
7	102.0	98.44	98.67	98.64	98.83	3.56	3.33	3.36	3.17
8	164.4	167.21	167.47	167.47	167.13	2.81	3.07	3.07	2.73
8a	101.6	100.45	100.42	100.43	100.19	1.15	1.18	1.17	1.41
9	37.3	43.97	40.97	43.72	45.32	6.67	3.67	6.42	8.02
10	67.2	66.34	68.35	75.57	74.23	0.86	1.15	8.37	7.03
11	29.8	34.67	33.04	33.68	34.59	4.87	3.24	3.88	4.79
12	18.1	22.22	22.10	27.49	27.31	4.12	4.00	9.39	9.21
13	31.5	33.13	33.71	35.67	35.97	1.63	2.21	4.17	4.47
14	67.4	70.78	70.14	76.32	75.88	3.38	2.74	8.92	8.48
15	19.7	18.69	20.13	23.93	23.97	1.01	0.43	4.23	4.27
MAE[b]						2.82	2.48	4.25	4.29

[a] $\mathbf{|\Delta\delta|(^{13}C)} = |\delta_{exp} - \delta_{calc}|$ (^{13}C), ppm: absolute differences for experimental versus calculated ^{13}C NMR chemical shifts
[b] **MAE** = $\Sigma[|(\delta_{exp} - \delta_{calcd})|]/n$, summation through n of the absolute error values (difference of the absolute values between corresponding experimental and ^{13}C chemical shifts), normalized to the number of the chemical shifts

Table S7. ^{13}C experimental related to exp_**1c** set of data and calculated NMR chemical shifts (MPW1PW91/6-311+g(d,p) level of theory) related to calc_**1a**, calc_**1b**, calc_**1c**, calc_**1d** sets of data, with [a]$|\Delta\delta|(^{13}C)$ and [b]MAE values. Chemical shift data here reported were produced using benzene as reference compound for sp^2 carbons (highlighted in yellow), and tetramethylsilane (TMS) for sp^3 carbons.

	$\delta_{exp}(^{13}C)$, ppm	$\delta_{calc}(^{13}C)$, ppm				$|\Delta\delta|$ (^{13}C), ppm[a]			
**Position**	exp_**1c**	calc_**1a**	calc_**1b**	calc_**1c**	calc_**1d**	calc_**1a**	calc_**1b**	calc_**1c**	calc_**1d**
1	171.7	174.92	174.85	174.99	174.84	3.22	3.15	3.29	3.14
3	78.0	76.11	77.30	77.23	75.96	1.89	0.70	0.77	2.04
4	34.5	37.32	35.68	35.96	37.41	2.82	1.18	1.46	2.91
4a	143.6	143.90	144.02	144.05	143.74	0.30	0.42	0.45	0.14
5	108.1	104.22	103.73	103.70	104.59	3.88	4.37	4.40	3.51
6	165.8	163.20	163.66	163.57	163.48	2.60	2.14	2.23	2.32
7	102.3	98.44	98.67	98.64	98.83	3.86	3.63	3.66	3.47
8	166.4	167.21	167.47	167.47	167.13	0.81	1.07	1.07	0.73
8a	101.8	100.45	100.42	100.43	100.19	1.35	1.38	1.37	1.61
9	42.2	43.97	40.97	43.72	45.32	1.77	1.23	1.52	3.12
10	75.2	66.34	68.35	75.57	74.23	8.86	6.85	0.37	0.97
11	32.5	34.67	33.04	33.68	34.59	2.17	0.54	1.18	2.09
12	22.6	22.22	22.10	27.49	27.31	0.38	0.50	4.89	4.71
13	33.8	33.13	33.71	35.67	35.97	0.67	0.09	1.87	2.17
14	75.5	70.78	70.14	76.32	75.88	4.72	5.36	0.82	0.38
15	24.7	18.69	20.13	23.93	23.97	6.01	4.57	0.77	0.73
MAE[b]						2.83	2.32	1.88	2.13

[a] **$|\Delta\delta|(^{13}C)$** = $|\delta_{exp} - \delta_{calc}|$ (^{13}C), ppm: absolute differences for experimental versus calculated ^{13}C NMR chemical shifts
[b] **MAE** = $\Sigma[|(\delta_{exp} - \delta_{calcd})|]/n$, summation through n of the absolute error values (difference of the absolute values between corresponding experimental and ^{13}C chemical shifts), normalized to the number of the chemical shifts

Table S8. ^{13}C experimental related to exp_**1d** set of data and calculated NMR chemical shifts (MPW1PW91/6-311+g(d,p) level of theory) related to calc_**1a**, calc_**1b**, calc_**1c**, calc_**1d** sets of data, with [a]|Δδ|(^{13}C) and [b]MAE values. Chemical shift data here reported were produced using benzene as reference compound for sp^2 carbons (highlighted in yellow), and tetramethylsilane (TMS) for sp^3 carbons.

	δ_{exp}(^{13}C), ppm	δ_{calc}(^{13}C), ppm				|Δδ| (^{13}C), ppm[a]			
**Position**	exp_**1d**	calc_**1a**	calc_**1b**	calc_**1c**	calc_**1d**	calc_**1a**	calc_**1b**	calc_**1c**	calc_**1d**
1	171.7	174.92	174.85	174.99	174.84	3.22	3.15	3.29	3.14
3	77.6	76.11	77.30	77.23	75.96	1.49	0.30	0.37	1.64
4	34.6	37.32	35.68	35.96	37.41	2.72	1.08	1.36	2.81
4a	143.7	143.90	144.02	144.05	143.74	0.20	0.32	0.35	0.04
5	108.0	104.22	103.73	103.70	104.59	3.78	4.27	4.30	3.41
6	165.8	163.20	163.66	163.57	163.48	2.60	2.14	2.23	2.32
7	102.3	98.44	98.67	98.64	98.83	3.86	3.63	3.66	3.47
8	166.4	167.21	167.47	167.47	167.13	0.81	1.07	1.07	0.73
8a	101.7	100.45	100.42	100.43	100.19	1.25	1.28	1.27	1.51
9	43.0	43.97	40.97	43.72	45.32	0.97	2.03	0.72	2.32
10	74.8	66.34	68.35	75.57	74.23	8.46	6.45	0.77	0.57
11	33.1	34.67	33.04	33.68	34.59	1.57	0.06	0.58	1.49
12	22.5	22.22	22.10	27.49	27.31	0.28	0.40	4.99	4.81
13	34.5	33.13	33.71	35.67	35.97	1.37	0.79	1.17	1.47
14	75.3	70.78	70.14	76.32	75.88	4.52	5.16	1.02	0.58
15	24.8	18.69	20.13	23.93	23.97	6.11	4.67	0.87	0.83
MAE[b]						2.70	2.30	1.75	1.95

[a] **|Δδ|(^{13}C)** = $|\delta_{exp} - \delta_{calc}|$ (^{13}C), ppm: absolute differences for experimental versus calculated ^{13}C NMR chemical shifts
[b] **MAE** = $\Sigma[|(\delta_{exp} - \delta_{calcd})|]/n$, summation through n of the absolute error values (difference of the absolute values between corresponding experimental and ^{13}C chemical shifts), normalized to the number of the chemical shifts

Table S9. ^{13}C experimental related to exp_**1a** set of data and calculated NMR chemical shifts (B97-2/cc-pVTZ level of theory) related to calc_**1a**, calc_**1b**, calc_**1c**, calc_**1d** sets of data, with $^{a}|\Delta\delta|(^{13}C)$ and ^{b}MAE values. Chemical shift data here reported were produced using benzene as reference compound for sp^2 carbons (highlighted in yellow), and tetramethylsilane (TMS) for sp^3 carbons.

	$\delta_{exp}(^{13}C)$, ppm	$\delta_{calc}(^{13}C)$, ppm				$\|\Delta\delta\|$ (^{13}C), ppm^{a}			
**Position**	exp_**1a**	calc_**1a**	calc_**1b**	calc_**1c**	calc_**1d**	calc_**1a**	calc_**1b**	calc_**1c**	calc_**1d**
1	169.9	163.65	163.79	163.82	163.62	6.25	6.11	6.08	6.29
3	76.3	72.81	73.56	73.37	72.48	3.49	2.74	2.93	3.82
4	33.6	34.93	33.52	33.73	35.02	1.33	0.08	0.13	1.42
4a	141.8	145.27	145.07	145.11	145.30	3.47	3.27	3.31	3.50
5	106.7	104.00	104.01	104.00	104.01	2.70	2.69	2.70	2.69
6	163.1	164.20	164.19	164.17	164.08	1.10	1.09	1.07	0.98
7	102.0	98.85	98.84	98.80	98.83	3.15	3.16	3.20	3.17
8	164.3	168.76	168.71	168.70	168.71	4.46	4.41	4.40	4.41
8a	101.5	101.38	101.20	101.15	101.45	0.12	0.30	0.35	0.05
9	39.3	40.76	38.21	40.74	42.54	1.46	1.09	1.44	3.24
10	66.6	64.23	65.24	71.87	71.21	2.37	1.36	5.27	4.61
11	30.9	31.53	30.46	30.94	31.66	0.63	0.44	0.04	0.76
12	18.1	20.24	20.19	24.80	24.74	2.14	2.09	6.70	6.64
13	30.9	30.21	30.54	32.33	32.55	0.69	0.36	1.43	1.65
14	68.0	66.65	66.18	71.98	71.73	1.35	1.82	3.98	3.73
15	18.9	18.45	19.27	22.82	22.76	0.45	0.37	3.92	3.86
MAE^{b}						2.20	1.96	2.93	3.18

^{a} **$|\Delta\delta|(^{13}C)$** = $|\delta_{exp} - \delta_{calc}|$ (^{13}C), ppm: absolute differences for experimental versus calculated ^{13}C NMR chemical shifts
^{b} **MAE** = $\Sigma[|(\delta_{exp} - \delta_{calcd})|]/n$, summation through n of the absolute error values (difference of the absolute values between corresponding experimental and ^{13}C chemical shifts), normalized to the number of the chemical shifts

Table S10. ^{13}C experimental related to exp_**1b** set of data and calculated NMR chemical shifts (B97-2/cc-pVTZ level of theory) related to calc_**1a**, calc_**1b**, calc_**1c**, calc_**1d** sets of data, with [a]$|\Delta\delta|(^{13}C)$ and [b]MAE values. Chemical shift data here reported were produced using benzene as reference compound for sp^2 carbons (highlighted in yellow), and tetramethylsilane (TMS) for sp^3 carbons.

	$\delta_{exp}(^{13}C)$, ppm	$\delta_{calc}(^{13}C)$, ppm				$\lvert\Delta\delta\rvert$ (^{13}C), ppm[a]			
**Position**	exp_**1b**	calc_**1a**	calc_**1b**	calc_**1c**	calc_**1d**	calc_**1a**	calc_**1b**	calc_**1c**	calc_**1d**
1	170.0	163.65	163.79	163.82	163.62	6.35	6.21	6.18	6.38
3	76.6	72.81	73.56	73.37	72.48	3.79	3.04	3.23	4.12
4	32.6	34.93	33.52	33.73	35.02	2.33	0.92	1.13	2.42
4a	141.6	145.27	145.07	145.11	145.30	3.67	3.47	3.51	3.70
5	106.7	104.00	104.01	104.00	104.01	2.70	2.69	2.70	2.69
6	163.0	164.20	164.19	164.17	164.08	1.20	1.19	1.17	1.08
7	102.0	98.85	98.84	98.80	98.83	3.15	3.16	3.20	3.17
8	164.4	168.76	168.71	168.70	168.71	4.36	4.31	4.30	4.31
8a	101.6	101.38	101.20	101.15	101.45	0.22	0.40	0.45	0.15
9	37.3	40.76	38.21	40.74	42.54	3.46	0.91	3.44	5.24
10	67.2	64.23	65.24	71.87	71.21	2.97	1.96	4.67	4.01
11	29.8	31.53	30.46	30.94	31.66	1.73	0.66	1.14	1.86
12	18.1	20.24	20.19	24.80	24.74	2.14	2.09	6.70	6.64
13	31.5	30.21	30.54	32.33	32.55	1.29	0.96	0.83	1.05
14	67.4	66.65	66.18	71.98	71.73	0.75	1.22	4.58	4.33
15	19.7	18.45	19.27	22.82	22.76	1.25	0.43	3.12	3.06
MAE[b]						2.58	2.10	3.15	3.39

[a] **$|\Delta\delta|(^{13}C)$** = $|\delta_{exp} - \delta_{calc}|$ (^{13}C), ppm: absolute differences for experimental versus calculated ^{13}C NMR chemical shifts
[b] **MAE** = $\Sigma[|(\delta_{exp} - \delta_{calcd})|]/n$, summation through n of the absolute error values (difference of the absolute values between corresponding experimental and ^{13}C chemical shifts), normalized to the number of the chemical shifts

Table S11. ^{13}C experimental related to exp_**1c** set of data and calculated NMR chemical shifts (B97-2/cc-pVTZ level of theory) related to calc_**1a**, calc_**1b**, calc_**1c**, calc_**1d** sets of data, with [a]|Δδ|(^{13}C) and [b]MAE values. Chemical shift data here reported were produced using benzene as reference compound for sp^2 carbons (highlighted in yellow), and tetramethylsilane (TMS) for sp^3 carbons.

	δ_{exp}(^{13}C), ppm	δ_{calc}(^{13}C), ppm				|Δδ| (^{13}C), ppm[a]			
**Position**	exp_**1c**	calc_**1a**	calc_**1b**	calc_**1c**	calc_**1d**	calc_**1a**	calc_**1b**	calc_**1c**	calc_**1d**
1	171.7	163.65	163.79	163.82	163.62	8.05	7.91	7.88	8.08
3	78.0	72.81	73.56	73.37	72.48	5.19	4.44	4.63	5.52
4	34.5	34.93	33.52	33.73	35.02	0.43	0.98	0.77	0.52
4a	143.6	145.27	145.07	145.11	145.30	1.67	1.47	1.51	1.70
5	108.1	104.00	104.01	104.00	104.01	4.10	4.09	4.10	4.09
6	165.8	164.20	164.19	164.17	164.08	1.60	1.61	1.63	1.72
7	102.3	98.85	98.84	98.80	98.83	3.45	3.46	3.50	3.47
8	166.4	168.76	168.71	168.70	168.71	2.36	2.31	2.30	2.31
8a	101.8	101.38	101.20	101.15	101.45	0.42	0.60	0.65	0.35
9	42.2	40.76	38.21	40.74	42.54	1.44	3.99	1.46	0.34
10	75.2	64.23	65.24	71.87	71.21	10.97	9.96	3.33	3.99
11	32.5	31.53	30.46	30.94	31.66	0.97	2.04	1.56	0.84
12	22.6	20.24	20.19	24.80	24.74	2.36	2.41	2.20	2.14
13	33.8	30.21	30.54	32.33	32.55	3.59	3.26	1.47	1.25
14	75.5	66.65	66.18	71.98	71.73	8.85	9.32	3.52	3.77
15	24.7	18.45	19.27	22.82	22.76	6.25	5.43	1.88	1.94
MAE[b]						3.86	3.96	2.65	2.63

[a] **|Δδ|(^{13}C)** = $|\delta_{exp} - \delta_{calc}|$ (^{13}C), ppm: absolute differences for experimental versus calculated ^{13}C NMR chemical shifts
[b] **MAE** = $\Sigma[|(\delta_{exp} - \delta_{calcd})|]/n$, summation through n of the absolute error values (difference of the absolute values between corresponding experimental and ^{13}C chemical shifts), normalized to the number of the chemical shifts

Table S12. ^{13}C experimental related to exp_**1d** set of data and calculated NMR chemical shifts (B97-2/cc-pVTZ level of theory) related to calc_**1a**, calc_**1b**, calc_**1c**, calc_**1d** sets of data, with [a]$|\Delta\delta|(^{13}C)$ and [b]MAE values. Chemical shift data here reported were produced using benzene as reference compound for sp^2 carbons (highlighted in yellow), and tetramethylsilane (TMS) for sp^3 carbons.

	$\delta_{exp}(^{13}C)$, ppm	$\delta_{calc}(^{13}C)$, ppm				$\|\Delta\delta\|$ (^{13}C), ppm[a]			
**Position**	exp_**1d**	calc_**1a**	calc_**1b**	calc_**1c**	calc_**1d**	calc_**1a**	calc_**1b**	calc_**1c**	calc_**1d**
1	171.7	163.65	163.79	163.82	163.62	8.05	7.91	7.88	8.08
3	77.6	72.81	73.56	73.37	72.48	4.79	4.04	4.23	5.12
4	34.6	34.93	33.52	33.73	35.02	0.33	1.08	0.87	0.42
4a	143.7	145.27	145.07	145.11	145.30	1.57	1.37	1.41	1.60
5	108.0	104.00	104.01	104.00	104.01	4.00	3.99	4.00	3.99
6	165.8	164.20	164.19	164.17	164.08	1.60	1.61	1.63	1.72
7	102.3	98.85	98.84	98.80	98.83	3.45	3.46	3.50	3.47
8	166.4	168.76	168.71	168.70	168.71	2.36	2.31	2.30	2.31
8a	101.7	101.38	101.20	101.15	101.45	0.32	0.50	0.55	0.25
9	43.0	40.76	38.21	40.74	42.54	2.24	4.79	2.26	0.46
10	74.8	64.23	65.24	71.87	71.21	10.57	9.56	2.93	3.59
11	33.1	31.53	30.46	30.94	31.66	1.57	2.64	2.16	1.44
12	22.5	20.24	20.19	24.80	24.74	2.26	2.31	2.30	2.24
13	34.5	30.21	30.54	32.33	32.55	4.29	3.96	2.17	1.95
14	75.3	66.65	66.18	71.98	71.73	8.65	9.12	3.32	3.57
15	24.8	18.45	19.27	22.82	22.76	6.35	5.53	1.98	2.04
MAE[b]						3.90	4.01	2.72	2.64

[a] **$|\Delta\delta|(^{13}C)$** = $|\delta_{exp} - \delta_{calc}|$ (^{13}C), ppm: absolute differences for experimental versus calculated ^{13}C NMR chemical shifts
[b] **MAE** = $\Sigma[|(\delta_{exp} - \delta_{calcd})|]/n$, summation through n of the absolute error values (difference of the absolute values between corresponding experimental and ^{13}C chemical shifts), normalized to the number of the chemical shifts

Table S13. Comparison of the results obtained by computing the $MAE_{\Delta\Delta\delta}$ values (bottom) and the AVER_MAE values (top), the latter defined as the average of the four possible MAE values obtained from the comparison of the calculated and experimental chemical shifts, for each of the 24 comparison alignments. The comparison of the predicting power between the two approaches has been evaluated considering the "% error from the best value" parameter, defined as (e.g. for the AVER_MAE data): ((AVER_MAE/$AVER\_MAE_{best\ value}$)-1)×100).

**MPW1PW91/6-31g(d,p)**			**MPW1PW91/6-311+g(d,p)**			**B97-2/cc-pVTZ**		
**comparison alignment[a]**	**AVER_MAE**	**% error from best AVER_MAE value**	**comparison alignment[a]**	**AVER_MAE**	**% error from best AVER_MAE value**	**comparison alignment[a]**	**AVER_MAE**	**% error from best AVER_MAE value**
exp_**1a** exp_**1b** exp_**1c** exp_**1d**	2.177	0.000	exp_**1a** exp_**1b** exp_**1c** exp_**1d**	2.190	0.000	exp_**1a** exp_**1b** exp_**1c** exp_**1d**	2.397	0.000
exp_**1a** exp_**1b** exp_**1d** exp_**1c**	2.191	0.643	exp_**1a** exp_**1b** exp_**1d** exp_**1c**	2.202	0.548	exp_**1a** exp_**1b** exp_**1d** exp_**1c**	2.411	0.584
exp_**1b** exp_**1a** exp_**1c** exp_**1d**	2.239	2.848	exp_**1b** exp_**1a** exp_**1c** exp_**1d**	2.243	2.420	exp_**1b** exp_**1a** exp_**1c** exp_**1d**	2.459	2.587
exp_**1b** exp_**1a** exp_**1d** exp_**1c**	2.253	3.491	exp_**1b** exp_**1a** exp_**1d** exp_**1c**	2.255	2.968	exp_**1b** exp_**1a** exp_**1d** exp_**1c**	2.473	3.171
exp_**1c** exp_**1b** exp_**1a** exp_**1d**	2.671	22.692	exp_**1a** exp_**1c** exp_**1d** exp_**1b**	2.703	23.425	exp_**1c** exp_**1b** exp_**1a** exp_**1d**	2.883	20.275
exp_**1d** exp_**1b** exp_**1a** exp_**1c**	2.678	23.013	exp_**1a** exp_**1d** exp_**1c** exp_**1b**	2.730	24.658	exp_**1d** exp_**1b** exp_**1a** exp_**1c**	2.891	20.609
exp_**1c** exp_**1a** exp_**1b** exp_**1d**	2.689	23.519	exp_**1a** exp_**1c** exp_**1b** exp_**1d**	2.742	25.205	exp_**1c** exp_**1a** exp_**1b** exp_**1d**	2.901	21.026
exp_**1d** exp_**1a** exp_**1b** exp_**1c**	2.697	23.886	exp_**1b** exp_**1c** exp_**1d** exp_**1a**	2.746	25.388	exp_**1d** exp_**1a** exp_**1b** exp_**1c**	2.909	21.360
exp_**1d** exp_**1b** exp_**1c** exp_**1a**	2.739	25.815	exp_**1b** exp_**1d** exp_**1c** exp_**1a**	2.773	26.621	exp_**1d** exp_**1b** exp_**1c** exp_**1a**	2.957	23.363
exp_**1c** exp_**1b** exp_**1d** exp_**1a**	2.746	26.137	exp_**1a** exp_**1d** exp_**1b** exp_**1c**	2.781	26.986	exp_**1c** exp_**1b** exp_**1d** exp_**1a**	2.963	23.613
exp_**1d** exp_**1a** exp_**1c** exp_**1b**	2.758	26.688	exp_**1c** exp_**1b** exp_**1d** exp_**1a**	2.788	27.306	exp_**1d** exp_**1a** exp_**1c** exp_**1b**	2.975	24.113
exp_**1c** exp_**1a** exp_**1d** exp_**1b**	2.764	26.964	exp_**1d** exp_**1b** exp_**1c** exp_**1a**	2.788	27.306	exp_**1c** exp_**1a** exp_**1d** exp_**1b**	2.981	24.364
exp_**1a** exp_**1c** exp_**1b** exp_**1d**	2.768	27.147	exp_**1b** exp_**1c** exp_**1a** exp_**1d**	2.795	27.626	exp_**1a** exp_**1c** exp_**1b** exp_**1d**	2.985	24.531
exp_**1a** exp_**1d** exp_**1b** exp_**1c**	2.778	27.607	exp_**1c** exp_**1a** exp_**1d** exp_**1b**	2.797	27.717	exp_**1a** exp_**1d** exp_**1b** exp_**1c**	2.996	24.990
exp_**1b** exp_**1c** exp_**1a** exp_**1d**	2.812	29.169	exp_**1d** exp_**1a** exp_**1c** exp_**1b**	2.797	27.717	exp_**1b** exp_**1c** exp_**1a** exp_**1d**	3.029	26.366
exp_**1b** exp_**1d** exp_**1a** exp_**1c**	2.822	29.628	exp_**1b** exp_**1d** exp_**1a** exp_**1c**	2.834	29.406	exp_**1b** exp_**1d** exp_**1a** exp_**1c**	3.039	26.783
exp_**1a** exp_**1d** exp_**1c** exp_**1b**	2.839	30.409	exp_**1c** exp_**1a** exp_**1b** exp_**1d**	2.836	29.498	exp_**1a** exp_**1d** exp_**1c** exp_**1b**	3.062	27.743
exp_**1a** exp_**1c** exp_**1d** exp_**1b**	2.843	30.593	exp_**1c** exp_**1b** exp_**1a** exp_**1d**	2.836	29.498	exp_**1a** exp_**1c** exp_**1d** exp_**1b**	3.065	27.868
exp_**1b** exp_**1d** exp_**1c** exp_**1a**	2.883	32.430	exp_**1d** exp_**1a** exp_**1b** exp_**1c**	2.849	30.091	exp_**1b** exp_**1d** exp_**1c** exp_**1a**	3.105	29.537
exp_**1b** exp_**1c** exp_**1d** exp_**1a**	2.886	32.568	exp_**1d** exp_**1b** exp_**1a** exp_**1c**	2.849	30.091	exp_**1b** exp_**1c** exp_**1d** exp_**1a**	3.108	29.662
exp_**1d** exp_**1c** exp_**1a** exp_**1b**	3.330	52.963	exp_**1d** exp_**1c** exp_**1b** exp_**1a**	3.340	52.511	exp_**1d** exp_**1c** exp_**1a** exp_**1b**	3.544	47.851
exp_**1d** exp_**1c** exp_**1b** exp_**1a**	3.330	52.963	exp_**1d** exp_**1c** exp_**1a** exp_**1b**	3.350	52.968	exp_**1d** exp_**1c** exp_**1b** exp_**1a**	3.544	47.851
exp_**1c** exp_**1d** exp_**1a** exp_**1b**	3.333	53.101	exp_**1c** exp_**1d** exp_**1b** exp_**1a**	3.367	53.744	exp_**1c** exp_**1d** exp_**1a** exp_**1b**	3.548	48.018
exp_**1c** exp_**1d** exp_**1b** exp_**1a**	3.333	53.101	exp_**1c** exp_**1d** exp_**1a** exp_**1b**	3.376	54.155	exp_**1c** exp_**1d** exp_**1b** exp_**1a**	3.548	48.018

MPW1PW91/6-31g(d,p)			MPW1PW91/6-311+g(d,p)			B97-2/cc-pVTZ		
**comparison alignment[a]**	**$MAE_{\Delta\Delta\delta}$**	**% error from best $MAE_{\Delta\Delta\delta}$ value**	**comparison alignment[a]**	**$MAE_{\Delta\Delta\delta}$**	**% error from best $MAE_{\Delta\Delta\delta}$ value**	**comparison alignment[a]**	**$MAE_{\Delta\Delta\delta}$**	**% error from best $MAE_{\Delta\Delta\delta}$ value**
exp_1a exp_1b exp_1c exp_1d	1.066	0.000	exp_1a exp_1b exp_1c exp_1d	1.089	0.000	exp_1a exp_1b exp_1c exp_1d	1.070	0.000
exp_1a exp_1b exp_1d exp_1c	1.120	5.066	exp_1a exp_1b exp_1d exp_1c	1.157	6.244	exp_1a exp_1b exp_1d exp_1c	1.124	5.047
exp_1b exp_1a exp_1c exp_1d	1.224	14.822	exp_1b exp_1a exp_1c exp_1d	1.291	18.549	exp_1b exp_1a exp_1c exp_1d	1.232	15.140
exp_1b exp_1a exp_1d exp_1c	1.298	21.764	exp_1b exp_1a exp_1d exp_1c	1.380	26.722	exp_1b exp_1a exp_1d exp_1c	1.308	22.243
exp_1c exp_1b exp_1a exp_1d	2.487	133.302	exp_1c exp_1b exp_1a exp_1d	2.617	140.312	exp_1c exp_1b exp_1a exp_1d	2.471	130.935
exp_1d exp_1b exp_1a exp_1c	2.515	135.929	exp_1d exp_1b exp_1a exp_1c	2.635	141.965	exp_1d exp_1b exp_1a exp_1c	2.496	133.271
exp_1c exp_1a exp_1b exp_1d	2.541	138.368	exp_1c exp_1a exp_1b exp_1d	2.674	145.546	exp_1c exp_1a exp_1b exp_1d	2.525	135.981
exp_1d exp_1b exp_1c exp_1a	2.581	142.120	exp_1d exp_1b exp_1c exp_1a	2.702	148.118	exp_1d exp_1a exp_1b exp_1c	2.568	140.000
exp_1d exp_1a exp_1b exp_1c	2.585	142.495	exp_1a exp_1c exp_1b exp_1d	2.706	148.485	exp_1d exp_1b exp_1c exp_1a	2.582	141.308
exp_1c exp_1b exp_1d exp_1a	2.601	143.996	exp_1d exp_1a exp_1b exp_1c	2.706	148.485	exp_1c exp_1b exp_1d exp_1a	2.603	143.271
exp_1a exp_1c exp_1b exp_1d	2.609	144.747	exp_1c exp_1b exp_1d exp_1a	2.725	150.230	exp_1a exp_1c exp_1b exp_1d	2.613	144.206
exp_1a exp_1d exp_1b exp_1c	2.647	148.311	exp_1a exp_1d exp_1b exp_1c	2.743	151.882	exp_1a exp_1d exp_1b exp_1c	2.650	147.664
exp_1d exp_1a exp_1c exp_1b	2.649	148.499	exp_1a exp_1c exp_1d exp_1b	2.746	152.158	exp_1d exp_1a exp_1c exp_1b	2.653	147.944
exp_1b exp_1c exp_1d exp_1a	2.656	149.156	exp_1b exp_1c exp_1d exp_1a	2.749	152.433	exp_1c exp_1a exp_1d exp_1b	2.678	150.280
exp_1c exp_1a exp_1d exp_1b	2.671	150.563	exp_1a exp_1d exp_1c exp_1b	2.773	154.637	exp_1b exp_1c exp_1d exp_1a	2.681	150.561
exp_1a exp_1c exp_1d exp_1b	2.674	150.844	exp_1d exp_1a exp_1c exp_1b	2.778	155.096	exp_1b exp_1c exp_1a exp_1d	2.688	151.215
exp_1b exp_1c exp_1a exp_1d	2.681	151.501	exp_1b exp_1d exp_1c exp_1a	2.784	155.647	exp_1a exp_1c exp_1d exp_1b	2.698	152.150
exp_1b exp_1d exp_1c exp_1a	2.691	152.439	exp_1b exp_1c exp_1a exp_1d	2.805	157.576	exp_1b exp_1d exp_1c exp_1a	2.715	153.738
exp_1a exp_1d exp_1c exp_1b	2.711	154.315	exp_1c exp_1a exp_1d exp_1b	2.812	158.219	exp_1b exp_1d exp_1a exp_1c	2.729	155.047
exp_1b exp_1d exp_1a exp_1c	2.721	155.253	exp_1b exp_1d exp_1a exp_1c	2.856	162.259	exp_1a exp_1d exp_1c exp_1b	2.734	155.514
exp_1d exp_1c exp_1b exp_1a	3.205	200.657	exp_1d exp_1c exp_1b exp_1a	3.410	213.131	exp_1d exp_1c exp_1b exp_1a	3.203	199.346
exp_1c exp_1d exp_1b exp_1a	3.236	203.565	exp_1c exp_1d exp_1b exp_1a	3.433	215.243	exp_1c exp_1d exp_1b exp_1a	3.235	202.336
exp_1d exp_1c exp_1a exp_1b	3.289	208.537	exp_1d exp_1c exp_1a exp_1b	3.496	221.028	exp_1d exp_1c exp_1a exp_1b	3.290	207.477
exp_1c exp_1d exp_1a exp_1b	3.324	211.820	exp_1c exp_1d exp_1a exp_1b	3.528	223.967	exp_1c exp_1d exp_1a exp_1b	3.328	211.028

[a]considering calc_**1a** calc_**1b** calc_**1c** and calc_**1d** starting fixed sequence related to the calculated sets of data

Table S14. ^{13}C experimental related to exp_**2a** set of data and calculated NMR chemical shifts (MPW1PW91/6-31g(d,p) level of theory) related to calc_**2a**, calc_**2b**, calc_**2c**, calc_**2d** sets of data, with [a]|Δδ|(^{13}C) and [b]MAE values.

	δ_{exp}(^{13}C), ppm	δ_{calc}(^{13}C), ppm				|Δδ| (^{13}C), ppm[a]			
**Position**	exp_**2a**	calc_**2a**	calc_**2b**	calc_**2c**	calc_**2d**	calc_**2a**	calc_**2b**	calc_**2c**	calc_**2d**
1	69.2	73.07	70.28	71.09	75.87	3.87	1.08	1.89	6.67
2	40	33.70	41.02	43.23	41.97	6.30	1.02	3.23	1.97
3	62.8	62.50	65.76	58.40	61.16	0.30	2.96	4.40	1.64
5	60.9	63.02	71.07	62.53	67.53	2.12	10.17	1.63	6.63
6	78.4	74.87	76.99	81.77	72.30	3.53	1.41	3.37	6.10
7	71.8	68.76	71.10	70.07	71.28	3.04	0.70	1.73	0.52
7a	75.6	74.03	75.46	68.45	66.39	1.57	0.14	7.15	9.21
8	62.4	60.06	64.71	62.69	65.55	2.34	2.31	0.29	3.15
9	42.8	44.23	45.18	46.38	39.33	1.43	2.38	3.58	3.47
11	173.9	163.51	164.23	165.97	164.45	10.39	9.67	7.93	9.45
12	22.6	23.07	23.00	23.35	22.78	0.47	0.40	0.75	0.18
MAE[b]						3.21	2.93	3.27	4.45

[a] **|Δδ|(^{13}C)** = $|\delta_{exp} - \delta_{calc}|$ (^{13}C), ppm: absolute differences for experimental versus calculated ^{13}C NMR chemical shifts
[b] **MAE** = $\Sigma[|(\delta_{exp} - \delta_{calcd})|]/n$, summation through n of the absolute error values (difference of the absolute values between corresponding experimental and ^{13}C chemical shifts), normalized to the number of the chemical shifts

Table S15. ^{13}C experimental related to exp_**2b** set of data and calculated NMR chemical shifts (MPW1PW91/6-31g(d,p) level of theory) related to calc_**2a**, calc_**2b**, calc_**2c**, calc_**2d** sets of data, with $^{a}|\Delta\delta|(^{13}C)$ and ^{b}MAE values.

	$\delta_{exp}(^{13}C)$, ppm	$\delta_{calc}(^{13}C)$, ppm				$\lvert\Delta\delta\rvert$ (^{13}C), ppm[a]			
**Position**	exp_**2b**	calc_**2a**	calc_**2b**	calc_**1c**	calc_**2d**	calc_**2a**	calc_**2b**	calc_**2c**	calc_**2d**
1	70.7	73.07	70.28	71.09	75.87	2.37	0.42	0.39	5.17
2	39	33.70	41.02	43.23	41.97	5.30	2.02	4.23	2.97
3	69.7	62.50	65.76	58.40	61.16	7.20	3.94	11.30	8.54
5	71.4	63.02	71.07	62.53	67.53	8.38	0.33	8.87	3.87
6	76.9	74.87	76.99	81.77	72.30	2.03	0.09	4.87	4.60
7	72.4	68.76	71.10	70.07	71.28	3.64	1.30	2.33	1.12
7a	76.5	74.03	75.46	68.45	66.39	2.47	1.04	8.05	10.11
8	65.9	60.06	64.71	62.69	65.55	5.84	1.19	3.21	0.35
9	42.3	44.23	45.18	46.38	39.33	1.93	2.88	4.08	2.97
11	173.9	163.51	164.23	165.97	164.45	10.39	9.67	7.93	9.45
12	22.6	23.07	23.00	23.35	22.78	0.47	0.40	0.75	0.18
MAE[b]						4.55	2.12	5.09	4.48

[a] **$|\Delta\delta|(^{13}C)$** = $|\delta_{exp} - \delta_{calc}|$ (^{13}C), ppm: absolute differences for experimental versus calculated ^{13}C NMR chemical shifts
[b] **MAE** = $\Sigma[|(\delta_{exp} - \delta_{calcd})|]/n$, summation through n of the absolute error values (difference of the absolute values between corresponding experimental and ^{13}C chemical shifts), normalized to the number of the chemical shifts

Table S16. ^{13}C experimental related to exp_**2c** set of data and calculated NMR chemical shifts (MPW1PW91/6-31g(d,p) level of theory) related to calc_**2a**, calc_**2b**, calc_**2c**, calc_**2d** sets of data, with [a]$|\Delta\delta|(^{13}C)$ and [b]MAE values.

	$\delta_{exp}(^{13}C)$, ppm	$\delta_{calc}(^{13}C)$, ppm				$\|\Delta\delta\|$ (^{13}C), ppm[a]			
**Position**	exp_**2c**	calc_**2a**	calc_**2b**	calc_**2c**	calc_**2d**	calc_**2a**	calc_**2b**	calc_**2c**	calc_**2d**
1	73.7	73.07	70.28	71.09	75.87	0.63	3.42	2.61	2.17
2	43.6	33.70	41.02	43.23	41.97	9.90	2.58	0.37	1.63
3	61.6	62.50	65.76	58.40	61.16	0.90	4.16	3.20	0.44
5	62.5	63.02	71.07	62.53	67.53	0.52	8.57	0.03	5.03
6	78.7	74.87	76.99	81.77	72.30	3.83	1.71	3.07	6.40
7	68.9	68.76	71.10	70.07	71.28	0.14	2.20	1.17	2.38
7a	73.9	74.03	75.46	68.45	66.39	0.13	1.56	5.45	7.51
8	63.3	60.06	64.71	62.69	65.55	3.24	1.41	0.61	2.25
9	40.8	44.23	45.18	46.38	39.33	3.43	4.38	5.58	1.47
11	173.7	163.51	164.23	165.97	164.45	10.19	9.47	7.73	9.25
12	22.6	23.07	23.00	23.35	22.78	0.47	0.40	0.75	0.18
MAE[b]						3.03	3.62	2.78	3.52

[a] **$|\Delta\delta|(^{13}C)$** = $|\delta_{exp} - \delta_{calc}|$ (^{13}C), ppm: absolute differences for experimental versus calculated ^{13}C NMR chemical shifts
[b] **MAE** = $\Sigma[|(\delta_{exp} - \delta_{calcd})|]/n$, summation through n of the absolute error values (difference of the absolute values between corresponding experimental and ^{13}C chemical shifts), normalized to the number of the chemical shifts

Table S17. ^{13}C experimental related to exp_**2d** set of data and calculated NMR chemical shifts (MPW1PW91/6-31g(d,p) level of theory) related to calc_**2a**, calc_**2b**, calc_**2c**, calc_**2d** sets of data, with $^{a}|\Delta\delta|(^{13}C)$ and [b]MAE values..

	$\delta_{exp}(^{13}C)$, ppm	$\delta_{calc}(^{13}C)$, ppm				$\lvert\Delta\delta\rvert$ (^{13}C), ppm[a]			
**Position**	exp_**2d**	calc_**2a**	calc_**2b**	calc_**2c**	calc_**2d**	calc_**2a**	calc_**2b**	calc_**2c**	calc_**2d**
1	76.6	73.07	70.28	71.09	75.87	3.53	6.32	5.51	0.73
2	39.8	33.70	41.02	43.23	41.97	6.10	1.22	3.43	2.17
3	66.2	62.50	65.76	58.40	61.16	3.70	0.44	7.80	5.04
5	71.5	63.02	71.07	62.53	67.53	8.48	0.43	8.97	3.97
6	74.1	74.87	76.99	81.77	72.30	0.77	2.89	7.67	1.80
7	73.9	68.76	71.10	70.07	71.28	5.14	2.80	3.83	2.62
7a	70.8	74.03	75.46	68.45	66.39	3.23	4.66	2.35	4.41
8	68.7	60.06	64.71	62.69	65.55	8.64	3.99	6.01	3.15
9	42.8	44.23	45.18	46.38	39.33	1.43	2.38	3.58	3.47
11	173.7	163.51	164.23	165.97	164.45	10.19	9.47	7.73	9.25
12	22.6	23.07	23.00	23.35	22.78	0.47	0.40	0.75	0.18
MAE[b]						4.70	3.18	5.24	3.34

[a] **$|\Delta\delta|(^{13}C)$** = $|\delta_{exp} - \delta_{calc}|$ (^{13}C), ppm: absolute differences for experimental versus calculated ^{13}C NMR chemical shifts
[b] **MAE** = $\Sigma[|(\delta_{exp} - \delta_{calcd})|]/n$, summation through n of the absolute error values (difference of the absolute values between corresponding experimental and ^{13}C chemical shifts), normalized to the number of the chemical shifts

Table S18. ^{13}C experimental related to exp_**2a** set of data and calculated NMR chemical shifts (MPW1PW91/6-311+g(d,p) level of theory) related to calc_**2a**, calc_**2b**, calc_**2c**, calc_**2d** sets of data, with [a]|Δδ|(^{13}C) and [b]MAE values.

	δ_{exp}(^{13}C), ppm	δ_{calc}(^{13}C), ppm				|Δδ| (^{13}C), ppm[a]			
**Position**	exp_**2a**	calc_**2a**	calc_**2b**	calc_**2c**	calc_**2d**	calc_**2a**	calc_**2b**	calc_**2c**	calc_**2d**
1	69.2	76.05	72.57	75.11	79.20	6.85	3.37	5.91	10.00
2	40	35.23	42.70	45.60	42.85	4.77	2.70	5.60	2.85
3	62.8	65.72	65.60	62.44	65.47	2.92	2.80	0.36	2.67
5	60.9	66.48	71.60	66.56	71.66	5.58	10.70	5.66	10.76
6	78.4	78.14	78.24	86.17	76.19	0.26	0.16	7.77	2.21
7	71.8	71.51	72.59	74.34	75.86	0.29	0.79	2.54	4.06
7a	75.6	80.71	76.59	73.28	70.73	5.11	0.99	2.32	4.87
8	62.4	64.21	65.42	66.53	68.98	1.81	3.02	4.13	6.58
9	42.8	44.91	42.35	48.05	41.42	2.11	0.45	5.25	1.38
11	173.9	177.01	177.14	178.89	177.26	3.11	3.24	4.99	3.36
12	22.6	24.52	24.20	25.08	24.29	1.92	1.60	2.48	1.69
MAE[b]						3.16	2.71	4.27	4.59

[a] **|Δδ|(^{13}C)** = $|\delta_{exp} - \delta_{calc}|$ (^{13}C), ppm: absolute differences for experimental versus calculated ^{13}C NMR chemical shifts
[b] **MAE** = $\Sigma[|(\delta_{exp} - \delta_{calcd})|]/n$, summation through n of the absolute error values (difference of the absolute values between corresponding experimental and ^{13}C chemical shifts), normalized to the number of the chemical shifts

Table S19. ^{13}C experimental related to exp_**2b** set of data and calculated NMR chemical shifts (MPW1PW91/6-311+g(d,p) level of theory) related to calc_**2a**, calc_**2b**, calc_**2c**, calc_**2d** sets of data, with [a]$|\Delta\delta|(^{13}C)$ and [b]MAE values.

	$\delta_{exp}(^{13}C)$, ppm	$\delta_{calc}(^{13}C)$, ppm				$\lvert\Delta\delta\rvert$ (^{13}C), ppm[a]			
**Position**	exp_**2b**	calc_**2a**	calc_**2b**	calc_**2c**	calc_**2d**	calc_**2a**	calc_**2b**	calc_**2c**	calc_**2d**
1	70.7	76.05	72.57	75.11	79.20	5.35	1.87	4.41	8.50
2	39	35.23	42.70	45.60	42.85	3.77	3.70	6.60	3.85
3	69.7	65.72	65.60	62.44	65.47	3.98	4.10	7.26	4.23
5	71.4	66.48	71.60	66.56	71.66	4.92	0.20	4.84	0.26
6	76.9	78.14	78.24	86.17	76.19	1.24	1.34	9.27	0.71
7	72.4	71.51	72.59	74.34	75.86	0.89	0.19	1.94	3.46
7a	76.5	80.71	76.59	73.28	70.73	4.21	0.09	3.22	5.77
8	65.9	64.21	65.42	66.53	68.98	1.69	0.48	0.63	3.08
9	42.3	44.91	42.35	48.05	41.42	2.61	0.05	5.75	0.88
11	173.9	177.01	177.14	178.89	177.26	3.11	3.24	4.99	3.36
12	22.6	24.52	24.20	25.08	24.29	1.92	1.60	2.48	1.69
MAE[b]						3.06	1.53	4.67	3.25

[a] **$|\Delta\delta|(^{13}C)$** = $|\delta_{exp} - \delta_{calc}|$ (^{13}C), ppm: absolute differences for experimental versus calculated ^{13}C NMR chemical shifts
[b] **MAE** = $\Sigma[|(\delta_{exp} - \delta_{calcd})|]/n$, summation through n of the absolute error values (difference of the absolute values between corresponding experimental and ^{13}C chemical shifts), normalized to the number of the chemical shifts

Table S20. ^{13}C experimental related to exp_**2c** set of data and calculated NMR chemical shifts (MPW1PW91/6-311+g(d,p) level of theory) related to calc_**2a**, calc_**2b**, calc_**2c**, calc_**2d** sets of data, with [a]|Δδ|(^{13}C) and [b]MAE values.

	δ_{exp}(^{13}C), ppm	δ_{calc}(^{13}C), ppm				|Δδ| (^{13}C), ppm[a]			
**Position**	exp_**2c**	calc_**2a**	calc_**2b**	calc_**2c**	calc_**2d**	calc_**2a**	calc_**2b**	calc_**2c**	calc_**2d**
1	73.7	76.05	72.57	75.11	79.20	2.35	1.13	1.41	5.50
2	43.6	35.23	42.70	45.60	42.85	8.37	0.90	2.00	0.75
3	61.6	65.72	65.60	62.44	65.47	4.12	4.00	0.84	3.87
5	62.5	66.48	71.60	66.56	71.66	3.98	9.10	4.06	9.16
6	78.7	78.14	78.24	86.17	76.19	0.56	0.46	7.47	2.51
7	68.9	71.51	72.59	74.34	75.86	2.61	3.69	5.44	6.96
7a	73.9	80.71	76.59	73.28	70.73	6.81	2.69	0.62	3.17
8	63.3	64.21	65.42	66.53	68.98	0.91	2.12	3.23	5.68
9	40.8	44.91	42.35	48.05	41.42	4.11	1.55	7.25	0.62
11	173.7	177.01	177.14	178.89	177.26	3.31	3.44	5.19	3.56
12	22.6	24.52	24.20	25.08	24.29	1.92	1.60	2.48	1.69
MAE[b]						3.55	2.79	3.64	3.95

[a] **|Δδ|(^{13}C)** = $|\delta_{exp} - \delta_{calc}|$ (^{13}C), ppm: absolute differences for experimental versus calculated ^{13}C NMR chemical shifts
[b] **MAE** = $\Sigma[|(\delta_{exp} - \delta_{calcd})|]/n$, summation through n of the absolute error values (difference of the absolute values between corresponding experimental and ^{13}C chemical shifts), normalized to the number of the chemical shifts

Table S21. ^{13}C experimental related to exp_**2d** set of data and calculated NMR chemical shifts (MPW1PW91/6-311+g(d,p) level of theory) related to calc_**2a**, calc_**2b**, calc_**2c**, calc_**2d** sets of data, with [a]|Δδ|(^{13}C) and [b]MAE values.

	δ_{exp}(^{13}C), ppm	δ_{calc}(^{13}C), ppm				|Δδ| (^{13}C), ppm[a]			
**Position**	exp_**2d**	calc_**2a**	calc_**2b**	calc_**2c**	calc_**2d**	calc_**2a**	calc_**2b**	calc_**2c**	calc_**2d**
1	76.6	76.05	72.57	75.11	79.20	0.55	4.03	1.49	2.60
2	39.8	35.23	42.70	45.60	42.85	4.57	2.90	5.80	3.05
3	66.2	65.72	65.60	62.44	65.47	0.48	0.60	3.76	0.73
5	71.5	66.48	71.60	66.56	71.66	5.02	0.10	4.94	0.16
6	74.1	78.14	78.24	86.17	76.19	4.04	4.14	12.07	2.09
7	73.9	71.51	72.59	74.34	75.86	2.39	1.31	0.44	1.96
7a	70.8	80.71	76.59	73.28	70.73	9.91	5.79	2.48	0.07
8	68.7	64.21	65.42	66.53	68.98	4.49	3.28	2.17	0.28
9	42.8	44.91	42.35	48.05	41.42	2.11	0.45	5.25	1.38
11	173.7	177.01	177.14	178.89	177.26	3.31	3.44	5.19	3.56
12	22.6	24.52	24.20	25.08	24.29	1.92	1.60	2.48	1.69
MAE[b]						3.53	2.51	4.19	1.60

[a] **|Δδ|(^{13}C)** = $|\delta_{exp} - \delta_{calc}|$ (^{13}C), ppm: absolute differences for experimental versus calculated ^{13}C NMR chemical shifts
[b] **MAE** = $\Sigma[|(\delta_{exp} - \delta_{calcd})|]/n$, summation through n of the absolute error values (difference of the absolute values between corresponding experimental and ^{13}C chemical shifts), normalized to the number of the chemical shifts

Table S22. ^{13}C experimental related to exp_**2a** set of data and calculated NMR chemical shifts (B97-2/cc-pVTZ level of theory) related to calc_**2a**, calc_**2b**, calc_**2c**, calc_**2d** sets of data, with [a]$|\Delta\delta|(^{13}C)$ and [b]MAE values.

	$\delta_{exp}(^{13}C)$, ppm	$\delta_{calc}(^{13}C)$, ppm				$|\Delta\delta|$ (^{13}C), ppm[a]			
**Position**	exp_**2a**	calc_**2a**	calc_**2b**	calc_**2c**	calc_**2d**	calc_**2a**	calc_**2b**	calc_**2c**	calc_**2d**
1	69.2	77.10	73.18	75.81	78.99	7.90	3.98	6.61	9.79
2	40	35.66	43.07	45.53	41.93	4.34	3.07	5.53	1.93
3	62.8	66.73	67.70	62.97	67.15	3.93	4.90	0.17	4.35
5	60.9	67.55	72.90	67.07	72.76	6.65	12.00	6.17	11.86
6	78.4	79.39	80.47	86.37	78.04	0.99	2.07	7.97	0.37
7	71.8	72.86	73.56	75.09	76.47	1.06	1.76	3.29	4.67
7a	75.6	78.75	77.54	73.28	71.30	3.15	1.94	2.32	4.30
8	62.4	64.06	65.84	67.08	69.30	1.66	3.44	4.68	6.90
9	42.8	46.80	44.15	48.68	42.94	4.00	1.35	5.88	0.14
11	173.9	173.34	174.13	175.50	174.14	0.56	0.23	1.60	0.24
12	22.6	24.09	23.94	24.38	23.86	1.49	1.34	1.78	1.26
MAE[b]						3.25	3.28	4.18	4.16

[a] **$|\Delta\delta|(^{13}C)$** = $|\delta_{exp} - \delta_{calc}|$ (^{13}C), ppm: absolute differences for experimental versus calculated ^{13}C NMR chemical shifts
[b] **MAE** = $\Sigma[|(\delta_{exp} - \delta_{calcd})|]/n$, summation through n of the absolute error values (difference of the absolute values between corresponding experimental and ^{13}C chemical shifts), normalized to the number of the chemical shifts

Table S23. ^{13}C experimental related to exp_**2b** set of data and calculated NMR chemical shifts (B97-2/cc-pVTZ level of theory) related to calc_**2a**, calc_**2b**, calc_**2c**, calc_**2d** sets of data, with [a]$|\Delta\delta|$(^{13}C) and [b]MAE values.

	δ_{exp}(^{13}C), ppm	δ_{calc}(^{13}C), ppm				$\|\Delta\delta\|$ (^{13}C), ppm[a]			
**Position**	exp_**2b**	calc_**2a**	calc_**2b**	calc_**2c**	calc_**2d**	calc_**2a**	calc_**2b**	calc_**2c**	calc_**2d**
1	70.7	77.10	73.18	75.81	78.99	6.40	2.48	5.11	8.29
2	39	35.66	43.07	45.53	41.93	3.34	4.07	6.53	2.93
3	69.7	66.73	67.70	62.97	67.15	2.97	2.00	6.73	2.55
5	71.4	67.55	72.90	67.07	72.76	3.85	1.50	4.33	1.36
6	76.9	79.39	80.47	86.37	78.04	2.49	3.57	9.47	1.13
7	72.4	72.86	73.56	75.09	76.47	0.46	1.16	2.69	4.07
7a	76.5	78.75	77.54	73.28	71.30	2.25	1.04	3.22	5.20
8	65.9	64.06	65.84	67.08	69.30	1.84	0.06	1.18	3.40
9	42.3	46.80	44.15	48.68	42.94	4.50	1.85	6.38	0.64
11	173.9	173.34	174.13	175.50	174.14	0.56	0.23	1.60	0.24
12	22.6	24.09	23.94	24.38	23.86	1.49	1.34	1.78	1.26
MAE[b]						2.74	1.75	4.46	2.82

[a] **$|\Delta\delta|$(^{13}C)** = $|\delta_{exp} - \delta_{calc}|$ (^{13}C), ppm: absolute differences for experimental versus calculated ^{13}C NMR chemical shifts
[b] **MAE** = $\Sigma[|(\delta_{exp} - \delta_{calcd})|]/n$, summation through n of the absolute error values (difference of the absolute values between corresponding experimental and ^{13}C chemical shifts), normalized to the number of the chemical shifts

Table S24. ^{13}C experimental related to exp_**2c** set of data and calculated NMR chemical shifts (B97-2/cc-pVTZ level of theory) related to calc_**2a**, calc_**2b**, calc_**2c**, calc_**2d** sets of data, with [a]$|\Delta\delta|(^{13}C)$ and [b]MAE values.

	$\delta_{exp}(^{13}C)$, ppm	$\delta_{calc}(^{13}C)$, ppm				$\lvert\Delta\delta\rvert$ (^{13}C), ppm[a]			
**Position**	exp_**2c**	calc_**2a**	calc_**2b**	calc_**2c**	calc_**2d**	calc_**2a**	calc_**2b**	calc_**2c**	calc_**2d**
1	73.7	77.10	73.18	75.81	78.99	3.40	0.52	2.11	5.29
2	43.6	35.66	43.07	45.53	41.93	7.94	0.53	1.93	1.67
3	61.6	66.73	67.70	62.97	67.15	5.13	6.10	1.37	5.55
5	62.5	67.55	72.90	67.07	72.76	5.05	10.40	4.57	10.26
6	78.7	79.39	80.47	86.37	78.04	0.69	1.77	7.67	0.67
7	68.9	72.86	73.56	75.09	76.47	3.96	4.66	6.19	7.57
7a	73.9	78.75	77.54	73.28	71.30	4.85	3.64	0.62	2.60
8	63.3	64.06	65.84	67.08	69.30	0.76	2.54	3.78	6.00
9	40.8	46.80	44.15	48.68	42.94	6.00	3.35	7.88	2.14
11	173.7	173.34	174.13	175.50	174.14	0.36	0.43	1.80	0.44
12	22.6	24.09	23.94	24.38	23.86	1.49	1.34	1.78	1.26
MAE[b]						3.60	3.21	3.61	3.95

[a] **$|\Delta\delta|(^{13}C)$** = $|\delta_{exp} - \delta_{calc}|$ (^{13}C), ppm: absolute differences for experimental versus calculated ^{13}C NMR chemical shifts
[b] **MAE** = $\Sigma[|(\delta_{exp} - \delta_{calcd})|]/n$, summation through n of the absolute error values (difference of the absolute values between corresponding experimental and ^{13}C chemical shifts), normalized to the number of the chemical shifts

Table S25. ^{13}C experimental related to exp_**2d** set of data and calculated NMR chemical shifts (B97-2/cc-pVTZ level of theory) related to calc_**2a**, calc_**2b**, calc_**2c**, calc_**2d** sets of data, with [a]$|\Delta\delta|(^{13}C)$ and [b]MAE values.

	$\delta_{exp}(^{13}C)$, ppm	$\delta_{calc}(^{13}C)$, ppm				$|\Delta\delta|$ (^{13}C), ppm[a]			
**Position**	exp_**2d**	calc_**2a**	calc_**2b**	calc_**2c**	calc_**2d**	calc_**2a**	calc_**2b**	calc_**2c**	calc_**2d**
1	76.6	77.10	73.18	75.81	78.99	0.50	3.42	0.79	2.39
2	39.8	35.66	43.07	45.53	41.93	4.14	3.27	5.73	2.13
3	66.2	66.73	67.70	62.97	67.15	0.53	1.50	3.23	0.95
5	71.5	67.55	72.90	67.07	72.76	3.95	1.40	4.43	1.26
6	74.1	79.39	80.47	86.37	78.04	5.29	6.37	12.27	3.94
7	73.9	72.86	73.56	75.09	76.47	1.04	0.34	1.19	2.57
7a	70.8	78.75	77.54	73.28	71.30	7.95	6.74	2.48	0.50
8	68.7	64.06	65.84	67.08	69.30	4.64	2.86	1.62	0.59
9	42.8	46.80	44.15	48.68	42.94	4.00	1.35	5.88	0.14
11	173.7	173.34	174.13	175.50	174.14	0.36	0.43	1.80	0.44
12	22.6	24.09	23.94	24.38	23.86	1.49	1.34	1.78	1.26
MAE[b]						3.08	2.64	3.75	1.47

[a] **$|\Delta\delta|(^{13}C)$** = $|\delta_{exp} - \delta_{calc}|$ (^{13}C), ppm: absolute differences for experimental versus calculated ^{13}C NMR chemical shifts
[b] **MAE** = $\Sigma[|(\delta_{exp} - \delta_{calcd})|]/n$, summation through n of the absolute error values (difference of the absolute values between corresponding experimental and ^{13}C chemical shifts), normalized to the number of the chemical shifts

Table S26. Sampled and DFT geometry optimized conformers related to calc_**1a**, with energy values (Hartree) and related % contribution on the final Boltzmann distribution for the three employed functional/basis set combinations.

Conformer	MPW1PW91/6-31g(d,p)		MPW1PW91/6-311+g(d,p)		B97-2/cc-pVTZ	
	**Energy (Hartree)**	**% contribution on the Boltzmann distribution**	**Energy (Hartree)**	**% contribution on the Boltzmann distribution**	**Energy (Hartree)**	**% contribution on the Boltzmann distribution**
calc_**1a** conf_1	-997.7100047	52.18%	-997.9450900	64.59%	-997.9038062	63.05%
calc_**1a** conf_2	-997.7088563	15.46%	-997.9435817	13.07%	-997.9025508	16.68%
calc_**1a** conf_3	-997.7083563	9.10%	-997.9428143	5.80%	-997.9016043	6.12%
calc_**1a** conf_4	-997.7082807	8.40%	-997.9430366	7.34%	-997.9014365	5.12%
calc_**1a** conf_5	-997.7077996	5.05%	-997.9421001	2.72%	-997.9009142	2.95%
calc_**1a** conf_6	-997.7069035	1.95%	-997.9413632	1.25%	-997.8999644	1.08%
calc_**1a** conf_7	-997.7065498	1.34%	-997.9410529	0.90%	-997.8992952	0.53%
calc_**1a** conf_8	-997.7065316	1.32%	-997.9406889	0.61%	-997.8985534	0.24%
calc_**1a** conf_9	-997.7065042	1.28%	-997.9411241	0.97%	-997.9001548	1.32%
calc_**1a** conf_10	-997.7064842	1.25%	-997.9412112	1.06%	-997.9001646	1.33%
calc_**1a** conf_11	-997.7064661	1.23%	-997.9405997	0.56%	-997.8988813	0.34%
calc_**1a** conf_12	-997.7051654	0.31%	-997.9390339	0.11%	-997.8973415	0.07%
calc_**1a** conf_13	-997.7049233	0.24%	-997.9399940	0.29%	-997.8990698	0.42%
calc_**1a** conf_14	-997.7049203	0.24%	-997.9398862	0.26%	-997.8989558	0.37%
calc_**1a** conf_15	-997.7046194	0.17%	-997.9391947	0.13%	-997.8975500	0.08%
calc_**1a** conf_16	-997.7045258	0.16%	-997.9391402	0.12%	-997.8975233	0.08%
calc_**1a** conf_17	-997.7036931	0.07%	-997.9381556	0.04%	-997.8968189	0.04%
calc_**1a** conf_18	-997.7036183	0.06%	-997.9383642	0.05%	-997.8971150	0.05%
calc_**1a** conf_19	-997.7031591	0.04%	-997.9383256	0.05%	-997.8969003	0.04%
calc_**1a** conf_20	-997.7031085	0.04%	-997.9380534	0.04%	-997.8965821	0.03%
calc_**1a** conf_21	-997.7030426	0.03%	-997.9372161	0.02%	-997.8963949	0.02%
calc_**1a** conf_22	-997.7025020	0.02%	-997.9365943	0.01%	-997.8939768	0.00%
calc_**1a** conf_23	-997.7024711	0.02%	-997.9366162	0.01%	-997.8944996	0.00%
calc_**1a** conf_24	-997.7023113	0.02%	-997.9370736	0.01%	-997.8957753	0.01%
calc_**1a** conf_25	-997.7022650	0.01%	-997.9364762	0.01%	-997.8944422	0.00%
calc_**1a** conf_26	-997.7005001	0.00%	-997.9343100	0.00%	-997.8923604	0.00%
calc_**1a** conf_27	-997.6985464	0.00%	-997.9338218	0.00%	-997.8915728	0.00%

Table S27. Cartesian coordinates of the optimized geometries for the conformers related to calc_**1a**. The related energies and % contribution on the final Boltzmann distribution for the three employed functional/basis set combinations are reported in Table S26.

calc_**1a** conf_1

C	-5.7028004057	0.1252675446	3.626082063
C	-6.4765350458	0.0458975075	2.4749069827
C	-5.9161264461	-0.3304778576	1.2446825126
C	-4.5672593435	-0.6137808691	1.1774870237
C	-3.7564570587	-0.5214768629	2.3249839931
C	-4.3399762212	-0.1702163411	3.5661743008
O	-3.6301790667	-0.1097732216	4.6906907846
O	-7.8000267818	0.3187252075	2.4791182461
C	-3.8982038905	-1.0601586625	-0.091751582
C	-2.4570278509	-0.5851596574	-0.1154207915
O	-1.7651406027	-1.0334757411	1.0716722742
C	-2.3309739461	-0.8258908194	2.2686448041
O	-1.623267428	-0.9203752227	3.2667984604
C	-1.659698452	-1.1035592403	-1.2971019195
C	-0.2596633373	-0.5138051864	-1.3851381537
C	0.5562434291	-1.1018517604	-2.5319097751
C	1.8954012064	-0.3760565276	-2.6527678917
C	1.6634304127	1.1311419816	-2.7550557685
C	0.7721733727	1.6443923556	-1.6205334802
O	-0.4336902832	0.885803272	-1.5579142293
C	1.4763108474	1.7209574503	-0.2688444473
H	-6.1329634183	0.4003750881	4.5839867927
H	-6.5554810117	-0.391688831	0.3716011196
H	-2.7128839711	-0.4081852107	4.4581081802
H	-8.0702488338	0.5654031683	3.3734010062
H	-4.4270663075	-0.6727810017	-0.9675286616
H	-3.9203387134	-2.1565228384	-0.1584225392
H	-2.4264446933	0.5096940577	-0.1138933834
H	-2.2017007566	-0.8527090717	-2.216278581
H	-1.5941410674	-2.1957054886	-1.2346703076
H	0.2607737731	-0.7117972903	-0.4370119364
H	-0.0130008634	-0.9870004661	-3.4638427703
H	0.7059647587	-2.1752632973	-2.3685874878
H	2.4497493952	-0.7337318876	-3.5268620676
H	2.5156056302	-0.6059932697	-1.7774212686
H	2.6141370993	1.6759605439	-2.7559030134
H	1.1648736111	1.3502558141	-3.7068032053
H	0.4265655219	2.651829159	-1.8750847356
H	2.2795288812	2.4632800296	-0.3067642443
H	1.9204233957	0.7691177925	0.0336210453
H	0.7661144616	2.022335651	0.5055526512

calc_**1a** conf_2

C	-5.7663930421	0.0425980704	3.5039931249
C	-6.3760227421	0.1729049941	2.262499665
C	-5.6872983739	-0.1279925238	1.0773578359
C	-4.3746473175	-0.5473850328	1.1493681451
C	-3.7266766812	-0.6682866902	2.3934086755
C	-4.4424073472	-0.3923368026	3.5833629037
O	-3.8946002167	-0.5324891286	4.7881601984
O	-7.6553707707	0.5873854259	2.1304282026
C	-3.5782429005	-0.9216428035	-0.0687700996

C	-2.1108554107	-0.6016212635	0.1511195908
O	-1.6350485467	-1.2496484873	1.3512147868
C	-2.3416007507	-1.1175033697	2.4831772868
O	-1.7937230186	-1.4010400261	3.5433609905
C	-1.2013581235	-1.0676329616	-0.9701158964
C	0.2396330712	-0.56877036	-0.8070907774
C	1.1978635872	-1.1306297478	-1.8588226554
C	1.0037643447	-0.4637356476	-3.2178951842
C	1.0835842081	1.0516393629	-3.0558734959
C	0.0978542072	1.5311198421	-1.9946788313
O	0.3014288138	0.8534005105	-0.7595607381
C	0.2278746289	3.0106002641	-1.6897378285
H	-6.2982495222	0.2578754135	4.4254609846
H	-6.2017930872	-0.0248833704	0.1289498455
H	-2.9859886126	-0.9028385363	4.6410963761
H	-8.0232135871	0.7654651881	3.0060065816
H	-3.9436120811	-0.3875378307	-0.9510021122
H	-3.6883911297	-1.9956368959	-0.2715554127
H	-1.9842862279	0.4775326266	0.2966436536
H	-1.2031122918	-2.163539855	-0.9908881527
H	-1.6271458038	-0.7322188322	-1.9220858985
H	0.5791609586	-0.8792648559	0.1849527158
H	1.0765928588	-2.2174282624	-1.9272798335
H	2.2222556387	-0.9436977602	-1.5161417766
H	0.0301026741	-0.7389012907	-3.6443069426
H	1.7607741354	-0.8178467012	-3.9255220353
H	0.877525738	1.5618145541	-4.0037006155
H	2.0965821642	1.3374894036	-2.7444218153
H	-0.9243562888	1.3332143356	-2.3600382062
H	1.2325498118	3.2313556417	-1.3179558363
H	-0.4917756626	3.3090636167	-0.9231767703
H	0.0486226964	3.6070497862	-2.5889406486

calc_**1a** conf_3

C	-5.8456043311	0.0791484077	3.2974420107
C	-6.0677400596	0.5095667	1.9950118303
C	-5.1263843087	0.2796207655	0.9803171559
C	-3.9513165547	-0.3762685795	1.2872317515
C	-3.6930150912	-0.8041970346	2.6036966006
C	-4.6641923556	-0.5942037404	3.611829829
O	-4.4908784037	-1.018059544	4.8621478626
O	-7.1961459501	1.1628874249	1.6383259593
C	-2.910482609	-0.6970488754	0.2539063637
C	-1.5306908686	-0.6757828807	0.8838374661
O	-1.4870346646	-1.5756107269	2.0156398475
C	-2.4580181302	-1.5040013726	2.9381040428
O	-2.2629445242	-2.04361677	4.0230665679
C	-0.3908539321	-1.1113700784	-0.0257523531
C	0.1128959472	-0.0359805289	-0.9772581621
C	1.3496886452	-0.4906695708	-1.7482044864
C	1.7596131016	0.5620249566	-2.7750895876
C	0.566666606	0.9091481457	-3.6627765473
C	-0.6602075121	1.2950749436	-2.8327548203
O	-0.9485885707	0.2842082134	-1.86641205
C	-0.5659603311	2.681812881	-2.2025196771
H	-6.5773432209	0.2391701538	4.0831268844
H	-5.3418056196	0.6193436049	-0.0260850432
H	-3.6347709068	-1.5195048539	4.8700788944

H	-7.763323996	1.2620574931	2.4142622216
H	-2.9323498654	0.0035500386	-0.582213112
H	-3.0943024179	-1.6979105098	-0.1597314802
H	-1.3263140321	0.3315746033	1.2721466075
H	-0.6894247371	-1.9956164973	-0.60014595
H	0.4421573665	-1.4105620167	0.6190497718
H	0.3790410698	0.8557403527	-0.384611778
H	1.1146868726	-1.4338243349	-2.2579352908
H	2.1662787292	-0.6930169118	-1.0463169665
H	2.5927907407	0.1987958229	-3.3855601681
H	2.1233499595	1.458960763	-2.2582215781
H	0.8134332073	1.7203887689	-4.3566467311
H	0.3077863617	0.0333717892	-4.269458568
H	-1.540957301	1.2827279322	-3.4829543923
H	0.3247873476	2.80656003	-1.5808019546
H	-1.4443709263	2.8709404241	-1.5794311863
H	-0.5311547344	3.4465706114	-2.9843397844

calc_**1a** conf_4

C	-5.9352955581	-0.9158737704	2.7619282609
C	-6.1582912927	0.4335186197	2.5192018545
C	-5.1038710293	1.2980296055	2.1855243393
C	-3.8241974638	0.7918551868	2.0874838464
C	-3.5737670635	-0.5759943881	2.3073783463
C	-4.6412967548	-1.4322883946	2.6693255483
O	-4.4571571866	-2.7254241434	2.9249014892
O	-7.3901713759	0.9834601784	2.5962178491
C	-2.6291742392	1.6535131446	1.7975672786
C	-1.5792920276	0.8800539618	1.0135505201
O	-1.2483273271	-0.3513748104	1.6964872419
C	-2.2288009703	-1.1273375434	2.1785860659
O	-1.944800474	-2.277701427	2.5007818183
C	-1.9735814295	0.5821534576	-0.4283278928
C	-0.8568774329	-0.0756711193	-1.2255887284
C	-1.2810039469	-0.4442906445	-2.6433280723
C	-0.0832290208	-0.9728862092	-3.4308584679
C	1.0695041791	0.0277853918	-3.3608116043
C	1.3923004576	0.4204397711	-1.9164479103
O	0.2121609834	0.8610554053	-1.2481453877
C	2.1306725272	-0.6602863485	-1.1317818053
H	-6.7435184221	-1.5871742331	3.0347300809
H	-5.3161199748	2.3475950821	2.017931765
H	-3.4797359563	-2.8857483829	2.8568365382
H	-8.0291794231	0.2990010557	2.8347197268
H	-2.188670367	1.9865381187	2.7461303657
H	-2.9142494464	2.5539668057	1.2458684508
H	-0.6360694474	1.4271871905	1.0049100428
H	-2.2331295985	1.5296368631	-0.9152532986
H	-2.8665810532	-0.0522156137	-0.4519183294
H	-0.5406320731	-0.9880422198	-0.6987610739
H	-1.6824521852	0.4518590768	-3.1343818006
H	-2.0855013875	-1.1877617457	-2.608561455
H	-0.3596682201	-1.1583905102	-4.4741582479
H	0.2278583587	-1.9402541358	-3.0170567439
H	1.9669542062	-0.3719533492	-3.8462634527
H	0.7852046547	0.9357375538	-3.9060456715
H	2.0255348619	1.313808727	-1.9300253879
H	1.6084752979	-1.6206482651	-1.1318781702

H 2.2563424178 -0.3460093152 -0.0923543845
H 3.1226342034 -0.8238686264 -1.5641135438

calc_**1a** conf_5

C	-5.7542268419	0.0617368229	3.3863784529
C	-5.764845985	0.9975475601	2.3591571451
C	-4.7754815531	0.9992220216	1.3641758127
C	-3.7665531984	0.0589696186	1.415948051
C	-3.7216601019	-0.8876075431	2.4571440923
C	-4.7421161113	-0.8977295226	3.4384972541
O	-4.7727558099	-1.7970061124	4.419722763
O	-6.7240666532	1.9444962435	2.2615584933
C	-2.6956170041	-0.0320527712	0.3672642591
C	-1.4049563596	-0.5402261475	0.9813145617
O	-1.6327534769	-1.7925397242	1.6661615743
C	-2.6656757346	-1.8910038136	2.5166887491
O	-2.6768969942	-2.8345712687	3.3010400675
C	-0.2766856313	-0.8203364262	0.0010283151
C	0.3729246609	0.4357173182	-0.5923103219
C	1.6893239825	0.1393874044	-1.3168908572
C	1.4504235774	-0.5155829347	-2.6743055436
C	0.4869872618	0.3432478041	-3.4885662973
C	-0.7926852151	0.6116209935	-2.702902918
O	-0.5043555035	1.1842194105	-1.4295538791
C	-1.7214314235	1.5836434942	-3.403632594
H	-6.5255668545	0.0453706068	4.1500436105
H	-4.8240877593	1.7387991354	0.5733875468
H	-4.0186316143	-2.4190927615	4.2511323852
H	-7.3436592184	1.8451834845	2.9963349844
H	-2.5104850731	0.9334251174	-0.1075949008
H	-3.0111948079	-0.7264855274	-0.4238544797
H	-1.0616827186	0.1786610194	1.7383198818
H	0.49297137	-1.3731973992	0.5505091577
H	-0.6403048084	-1.4959226605	-0.7804868545
H	0.5834340273	1.1213857622	0.2371498609
H	2.3313173216	-0.4808665845	-0.6823187557
H	2.2102808436	1.0918196803	-1.4697956886
H	1.0321446269	-1.52182467	-2.5440119092
H	2.3993939846	-0.641435282	-3.2058870579
H	0.2358160757	-0.1395241482	-4.4396898389
H	0.9588932355	1.3056428974	-3.7255557022
H	-1.3199692814	-0.3462243215	-2.5619342308
H	-1.2268085814	2.5506355108	-3.5326906602
H	-2.6317105713	1.740847292	-2.8192173496
H	-2.0040460821	1.2016504212	-4.3887571792

calc_**1a** conf_6

C	-5.9451858806	-1.0203796414	2.6503891195
C	-6.1709819289	0.247218393	2.1292870136
C	-5.1061667661	1.0770564893	1.7442130504
C	-3.8124637043	0.6173221685	1.8785857541
C	-3.5561700619	-0.670929283	2.3851872548
C	-4.6373292831	-1.4882187364	2.7939944327
O	-4.4532733506	-2.6996465481	3.3130572618
O	-7.4157468521	0.748460069	1.9712319423
C	-2.6128365535	1.4590935222	1.5522020373
C	-1.4655302207	0.5960044646	1.0491805354

O	-1.1819445976	-0.4611407444	1.9939709025
C	-2.1925446764	-1.1758477546	2.5096961673
O	-1.919887503	-2.2296346452	3.0770093742
C	-1.685778582	-0.0024321955	-0.3363158172
C	-0.4241439646	-0.6858193698	-0.8763124393
C	-0.6504798936	-1.4335378919	-2.1915069343
C	-0.7759250843	-0.4765698512	-3.3742139602
C	0.4263368016	0.4639511197	-3.3959221035
C	0.5859968062	1.167622737	-2.0514485492
O	0.6624234976	0.2291364178	-0.9846106358
C	1.8483655866	2.0037305369	-1.9681470203
H	-6.7628164249	-1.6619991308	2.9634678513
H	-5.3225476825	2.0656183896	1.3560015128
H	-3.4710765558	-2.8278434047	3.3794109842
H	-8.0618708381	0.0949454821	2.2698321229
H	-2.2876443126	1.9861937545	2.458403669
H	-2.8558602929	2.2241200046	0.8090654693
H	-0.5395184998	1.1736510414	1.0326917606
H	-2.5055645915	-0.7285887548	-0.2994665844
H	-2.0139011231	0.7972182566	-1.0096387642
H	-0.0892869272	-1.3992030452	-0.1175598323
H	-1.5325383531	-2.0775671646	-2.104587296
H	0.2110188522	-2.0907580377	-2.3580425512
H	-1.702387135	0.1075807934	-3.2971521491
H	-0.8435210353	-1.0387318669	-4.3115504983
H	0.3284626981	1.2122429349	-4.1907082102
H	1.3401986458	-0.1096035691	-3.5982367714
H	-0.2860942851	1.8267765414	-1.9016615175
H	1.9269687808	2.4842909685	-0.9895803263
H	1.8494661849	2.7792348503	-2.7395395584
H	2.7287791063	1.3699827	-2.107676697

calc_**1a** conf_7

C	-5.8757080863	-0.7794434174	2.3556051759
C	-5.7886106866	0.3726029519	1.5837378208
C	-4.5506811835	0.9704688489	1.3026466101
C	-3.3975974124	0.3946665824	1.7960914154
C	-3.4566902499	-0.785553789	2.5608584685
C	-4.7129978481	-1.3642931574	2.8603371919
O	-4.8293806289	-2.4579539883	3.611076637
O	-6.8842031035	0.9766676583	1.0713134601
C	-2.0404195157	1.004261287	1.5955721005
C	-0.9707420724	-0.0737686421	1.5159736053
O	-1.0506737215	-0.9524323428	2.6627016193
C	-2.2422850465	-1.4154184036	3.066310244
O	-2.2537042514	-2.3533284714	3.858453333
C	-0.9896373469	-0.917296879	0.2402698978
C	-0.3122580342	-0.2664196605	-0.9585234189
C	-0.2134740968	-1.2202622315	-2.1473155336
C	0.3581454215	-0.5026000716	-3.368165818
C	-0.4483647263	0.76354305	-3.6498897082
C	-0.5633677327	1.6462424108	-2.4057995466
O	-1.0609961702	0.8911521579	-1.301671406
C	0.7208071954	2.3943695966	-2.0588488614
H	-6.8315004004	-1.2380964173	2.5891618223
H	-4.5239300285	1.8742084114	0.7052997036
H	-3.9118437322	-2.6870707185	3.9121017877
H	-7.6702434497	0.477760841	1.3297644818

H	-1.8158055337	1.6636043035	2.4440949192
H	-2.0034892057	1.6019077973	0.6837921032
H	0.0170203874	0.382416331	1.6235747153
H	-2.0188977867	-1.1818631979	-0.0266215077
H	-0.457431907	-1.8490300966	0.459075394
H	0.7084046487	0.0306205012	-0.6624417841
H	-1.2191180859	-1.5958411302	-2.3754225067
H	0.4038921149	-2.0837202862	-1.8753210591
H	0.3436263134	-1.1627342713	-4.2416472646
H	1.4112832859	-0.2517206791	-3.1889713219
H	-0.0068218813	1.3379558942	-4.4718472063
H	-1.4601380975	0.4790766116	-3.9619777788
H	-1.3471044549	2.3914062712	-2.576748547
H	0.9706656621	3.0993222344	-2.8576080608
H	1.5789478414	1.7297869423	-1.9270539605
H	0.5853236063	2.9618071686	-1.1339372164

calc_**1a** conf_8

C	-5.6686246361	-0.5212398905	1.8334752355
C	-5.3780463147	0.711425831	1.2628782252
C	-4.0669415304	1.2122333987	1.2375894653
C	-3.046416518	0.4583863671	1.7823732395
C	-3.3121214848	-0.8026019298	2.3490729737
C	-4.6422196996	-1.2849109258	2.3927768807
O	-4.9554467527	-2.4523015138	2.9500808184
O	-6.3345082305	1.4906740097	0.710874891
C	-1.6277885924	0.9458351137	1.8531953461
C	-0.6530535149	-0.2172467957	1.7619620106
O	-0.9695941019	-1.2220604782	2.7532974603
C	-2.2405534164	-1.6139960164	2.9173114833
O	-2.4464280408	-2.6429436861	3.5540918693
C	-0.5555386786	-0.8924485184	0.3943495811
C	0.2497859732	-0.0993448409	-0.6440775795
C	0.5868754926	-0.9244606973	-1.8900533802
C	-0.626876741	-1.093575602	-2.7997272418
C	-1.2159672681	0.2781679794	-3.1169856011
C	-1.5113751287	1.0508597506	-1.8356019828
O	-0.3523893594	1.1388113749	-1.0076914472
C	-1.9478186139	2.4781842345	-2.1023468706
H	-6.6822358638	-0.9080570291	1.8697318792
H	-3.8838706337	2.1869522222	0.8006384054
H	-4.1136790067	-2.8125259516	3.3337020405
H	-7.1899361423	1.0488410988	0.7929314312
H	-1.4746379816	1.4603072476	2.8106454017
H	-1.4023698816	1.6555143956	1.0547333932
H	0.3435038283	0.119879103	2.0598052303
H	-0.0430934154	-1.8476981787	0.552260087
H	-1.5577850808	-1.1338381375	0.0249311557
H	1.1902417465	0.2029095774	-0.1673583159
H	1.0011546183	-1.8940819864	-1.5934588376
H	1.3717393736	-0.3937736293	-2.4418608267
H	-1.3846454106	-1.7204781097	-2.3127579791
H	-0.3403100472	-1.6131500941	-3.7200424623
H	-2.1356261075	0.1858111283	-3.705556837
H	-0.5014296723	0.8571134984	-3.7164682568
H	-2.311897557	0.5299255848	-1.2865878536
H	-1.1591644766	3.0221901572	-2.6302560265
H	-2.1547695553	3.0044974362	-1.1666620814

H -2.8541415772 2.4942145023   -2.7142149241

calc_**1a** conf_9

C	-5.5189209604	-0.3032289356	4.0931611035
C	-6.236356258	0.4648880254	3.184524649
C	-5.787246225	0.6445511125	1.8668711805
C	-4.6033281606	0.0558899567	1.472872824
C	-3.8468932127	-0.7113198859	2.3796506112
C	-4.3257838933	-0.9095575292	3.697340797
O	-3.6738420991	-1.6593640234	4.5828497232
O	-7.3973171536	1.0708132904	3.5153361369
C	-4.0725521817	0.1693302407	0.0721350949
C	-2.5533143417	0.1369746153	0.0821301009
O	-2.0890397444	-1.0450161078	0.7671960292
C	-2.5971994277	-1.3415980781	1.9739255571
O	-1.9874695108	-2.1447250171	2.6724490059
C	-1.9492691065	0.1377308309	-1.3087274112
C	-0.4298554535	0.2846368383	-1.3771891703
C	0.3471603698	-1.0036419842	-1.1218627894
C	1.837439006	-0.7866036457	-1.3742036013
C	2.0481656026	-0.2078880142	-2.7711559066
C	1.190558729	1.0396677369	-2.9950111508
O	-0.1760161559	0.7772129101	-2.6874219252
C	1.7110259501	2.2787445909	-2.2707139766
H	-5.8685156851	-0.4588831109	5.1089176762
H	-6.3816976838	1.2416254475	1.185071767
H	-2.8957779224	-2.0461140572	4.1047838386
H	-7.6043655521	0.8788026708	4.4394609518
H	-4.4174946374	1.0927663224	-0.4016608499
H	-4.4442439422	-0.6654130202	-0.5371768276
H	-2.1818802165	1.0014026495	0.6495048082
H	-2.37175338	0.9924995361	-1.848353572
H	-2.2623220768	-0.7655642094	-1.8445577942
H	-0.1198305166	1.0397658389	-0.6342507699
H	-0.0333816112	-1.7729359544	-1.8065014081
H	0.1605993135	-1.355652439	-0.1045562136
H	2.3831225434	-1.7299936951	-1.2679834328
H	2.2460256535	-0.1083569183	-0.6139977487
H	3.1028862939	0.0315371917	-2.9480251265
H	1.7588914835	-0.9602232872	-3.5145663698
H	1.1705381485	1.2649927953	-4.0665694985
H	2.6866790329	2.5673674141	-2.673656318
H	1.8333316736	2.1210238415	-1.195607665
H	1.0192433084	3.1128560566	-2.4164323292

calc_**1a** conf_10

C	-5.6148711677	-0.5450433466	4.0095492508
C	-6.0276236825	0.6213304456	3.3775319666
C	-5.3923288604	1.0845579323	2.2145830732
C	-4.3295138475	0.3723088022	1.6986701364
C	-3.8785029497	-0.8024487925	2.3310510417
C	-4.5470856355	-1.2755269104	3.4861438046
O	-4.1919291187	-2.4023236238	4.0988381365
O	-7.0566277516	1.3636384702	3.8402114987
C	-3.6208909065	0.7802735498	0.4380977626
C	-2.1601283248	0.3655517782	0.4981347176
O	-2.0600231958	-1.0475350654	0.7716215447

C	-2.7625326565	-1.5677812548	1.7912780106
O	-2.4371010631	-2.6747435163	2.2068994995
C	-1.4058282064	0.6377203045	-0.7915719757
C	0.1246904677	0.5081172008	-0.69760902
C	0.6671506363	-0.9217996626	-0.7431222294
C	0.5850442927	-1.5092453752	-2.1500186716
C	1.2413566996	-0.5590254374	-3.148225499
C	0.6524220146	0.8423493044	-3.0215916061
O	0.7531660002	1.3185789053	-1.6839512652
C	1.3598900026	1.8653887473	-3.8884451226
H	-6.111802447	-0.9173863722	4.8998351573
H	-5.7516512078	1.9928127987	1.7448163691
H	-3.467556004	-2.8032388204	3.5531756635
H	-7.4118986084	0.9525060544	4.6393030532
H	-3.6895825003	1.8611379017	0.2852496676
H	-4.0943252259	0.2996840859	-0.4286366909
H	-1.6750120829	0.8895474081	1.3332856646
H	-1.8241864745	-0.0029930852	-1.5751932886
H	-1.6129889006	1.6751425086	-1.0784136809
H	0.4313957742	0.9703798425	0.2482985947
H	0.1343566487	-1.546484613	-0.022630884
H	1.7194921464	-0.8846470959	-0.4367968007
H	-0.4628281913	-1.6771266263	-2.4312139024
H	1.0682133777	-2.4915173418	-2.1749851754
H	1.1179658682	-0.9177446101	-4.1766707388
H	2.3194293991	-0.5003477337	-2.9496615378
H	-0.4102075072	0.8016363233	-3.3154678091
H	2.4148830134	1.9319821641	-3.6069737386
H	0.9095022482	2.8530157727	-3.7601513129
H	1.2970679269	1.586298983	-4.9442436638

calc_**1a** conf_11

C	-5.7595852361	-0.2566692914	3.2475952216
C	-5.5908745571	1.1114469396	3.0736022628
C	-4.4913006865	1.6271483597	2.3702691899
C	-3.553022837	0.7562781753	1.8555170418
C	-3.6879286862	-0.6333766685	2.0379163465
C	-4.8196089806	-1.1427423241	2.7189952252
O	-5.0221510689	-2.4489137904	2.877794923
O	-6.4727917153	2.0113937456	3.5618025416
C	-2.37408164	1.2167815056	1.0472076124
C	-1.1950020905	0.2917658526	1.2937721885
O	-1.5607398608	-1.0729123854	0.9963299236
C	-2.7028493252	-1.5633871208	1.4983779357
O	-2.8669926044	-2.7795285215	1.4735168184
C	0.0689536681	0.6020150291	0.5011758366
C	-0.1288607863	1.0268353132	-0.9616368371
C	1.2018632585	1.19438877	-1.7036662895
C	1.806156309	-0.1539484345	-2.0910684605
C	0.7665589219	-0.9902173165	-2.8333985935
C	-0.5124910201	-1.1078938458	-2.0114873547
O	-1.0151136248	0.1882970529	-1.6821354501
C	-1.6311747614	-1.8183041364	-2.745493314
H	-6.6173258064	-0.6623948268	3.7747682258
H	-4.4044927303	2.699812803	2.2414300602
H	-4.2929706369	-2.9065928045	2.3849551193
H	-7.1832328362	1.5433136984	4.0198601672
H	-2.1014353509	2.2455001266	1.3024404518

H	-2.6265029431	1.189653535	-0.0189418286
H	-0.945734837	0.3147529137	2.3629944359
H	0.5885875595	1.4233872307	1.0094178557
H	0.7258068877	-0.2699614303	0.5832767655
H	-0.6392720199	1.9967996851	-0.9537608889
H	1.8991861536	1.7810765469	-1.0944808639
H	1.0080571638	1.7707735703	-2.6157118962
H	2.1392674408	-0.694311373	-1.1957453185
H	2.6977018734	-0.0026895251	-2.7088769297
H	1.1545960345	-1.9916616894	-3.0511280816
H	0.5251153906	-0.5174359911	-3.7943718683
H	-0.2947232568	-1.6549413209	-1.0836172459
H	-1.3255477476	-2.8315096306	-3.0215772354
H	-1.8961821193	-1.2720527443	-3.6558877052
H	-2.5178608953	-1.8869756822	-2.1110299874

calc_**1a** conf_12

C	-5.9249304362	-0.5357044603	3.023367344
C	-5.9327495481	0.7211107323	2.4306493957
C	-4.8199116534	1.2010472088	1.723789544
C	-3.6903882079	0.41354019	1.6274774475
C	-3.6499721856	-0.8564930325	2.2357912533
C	-4.7908717957	-1.3421936793	2.9198448433
O	-4.8227724165	-2.5520860826	3.4748141676
O	-7.0064111377	1.5391225048	2.4989154397
C	-2.474580505	0.8384033223	0.8544784903
C	-1.2325511351	0.249924011	1.5003746162
O	-1.3486967947	-1.1874697711	1.6051785448
C	-2.4683131225	-1.704600019	2.1338572706
O	-2.4457970628	-2.8770326212	2.4959931539
C	0.1016520069	0.5494945386	0.8309028825
C	0.2839343806	0.2726486859	-0.6599519715
C	0.001258536	-1.153550631	-1.1232385523
C	0.2218246768	-1.2562354665	-2.6315355269
C	-0.6154792524	-0.1988249825	-3.3500342085
C	-0.369268446	1.2015502644	-2.7823927427
O	-0.5245151953	1.2078755754	-1.3638156141
C	0.961097925	1.8139998562	-3.2134375046
H	-6.7884120403	-0.9191529107	3.5578754103
H	-4.8696799845	2.181971384	1.2655128638
H	-3.9622950418	-2.9881662541	3.2432418765
H	-7.7037315731	1.1046086758	3.0073719725
H	-2.3866625442	1.927625901	0.8212833856
H	-2.5427407253	0.5055401743	-0.1858310882
H	-1.1710680638	0.6268835407	2.5304523962
H	0.3102612171	1.6137617911	0.9880838096
H	0.8687655009	-0.0081235633	1.3793843471
H	1.3444960411	0.4875469289	-0.8728798144
H	-1.0316525228	-1.4215499037	-0.8810244487
H	0.6437198836	-1.8535155311	-0.5788849544
H	-0.0441473751	-2.2556629481	-2.9910165308
H	1.2866785	-1.1187102763	-2.8600682318
H	-1.6774174334	-0.4403202005	-3.2211099434
H	-0.4149677847	-0.2001851171	-4.4273189818
H	-1.1673734363	1.8664882124	-3.1287134356
H	0.9616972095	1.9815810684	-4.2948161512
H	1.8194538072	1.1781347113	-2.9798959479
H	1.1055177357	2.7777181733	-2.717674806

calc_**1a** conf_13

C	-5.611353231	-0.0793545962	3.9153905794
C	-5.9299432686	0.8710752571	2.953470279
C	-5.2345006433	0.9374809586	1.7361023439
C	-4.2066931543	0.0479831564	1.4987327675
C	-3.8493955103	-0.9107421812	2.4654334342
C	-4.5789262209	-0.9877681595	3.6764709117
O	-4.3137727339	-1.9022419164	4.6062586559
O	-6.9207492054	1.771432269	3.1338144595
C	-3.4398822977	0.028223636	0.2064919027
C	-2.0101512748	-0.4206146265	0.4549246829
O	-1.9991627301	-1.6941746034	1.1361417298
C	-2.7678437108	-1.8580544688	2.2236516664
O	-2.5303376534	-2.8107407214	2.9587590151
C	-1.1742562366	-0.6348831158	-0.7967137274
C	-0.9110904541	0.6257629749	-1.6329207422
C	-0.0209103419	1.6716131779	-0.9545145074
C	1.4396638444	1.2270124628	-0.9149015302
C	1.8951454042	0.8417603103	-2.3203608315
C	0.9501577224	-0.1898649729	-2.9297268694
O	-0.3943966623	0.2801888975	-2.9102727948
C	1.2662711842	-0.5008528822	-4.3791675427
H	-6.1558084881	-0.14665752	4.8520228896
H	-5.5219121513	1.6850848515	1.0058256997
H	-3.6018840694	-2.4834557602	4.232678474
H	-7.3235113967	1.631424187	4.0009357646
H	-3.4493864981	1.01829302	-0.2574696809
H	-3.9142826318	-0.6650867741	-0.5010824661
H	-1.5226774146	0.2985654771	1.125769159
H	-0.2347450528	-1.0974254172	-0.4799933528
H	-1.6837647576	-1.3620378381	-1.4384284117
H	-1.8754737769	1.0885642596	-1.8688667377
H	-0.3914636532	1.8993462622	0.0513130998
H	-0.094120377	2.5985933685	-1.5352333722
H	1.5606314279	0.3706838576	-0.2392935934
H	2.0667534911	2.0286827242	-0.5110671041
H	2.9157374	0.4429193774	-2.3072004912
H	1.8998225605	1.7311085845	-2.9638727062
H	1.0221335779	-1.1202636186	-2.343475163
H	2.2781613072	-0.9050424815	-4.4743901588
H	1.1940314173	0.4071154324	-4.9850300289
H	0.5588862596	-1.2336528485	-4.775205702

calc_**1a** conf_14

C	-5.75895123	0.0108768015	3.6460255225
C	-5.9975405948	0.7447655917	2.4908218592
C	-5.1512827634	0.6439913943	1.375768987
C	-4.0548012028	-0.1917681642	1.4362625852
C	-3.7783053389	-0.9301542729	2.6020360153
C	-4.6572224267	-0.8439390408	3.7087738575
O	-4.4703316052	-1.5538051872	4.8186985801
O	-7.0507849942	1.5834282568	2.3773480379
C	-3.1234567599	-0.3862457057	0.2732751008
C	-1.717637622	-0.6754325483	0.7708724959
O	-1.7292450055	-1.7983249858	1.6780501859
C	-2.6259054977	-1.8209286524	2.6751253741

O	-2.440310754	-2.6049894733	3.5999725747
C	-0.7204408531	-1.0531644058	-0.3108948549
C	-0.4411565952	0.0251358657	-1.3524750777
C	0.1509273404	1.3098931558	-0.7768819728
C	0.5699649275	2.2575472498	-1.8998333464
C	1.4875298188	1.530099801	-2.8800212354
C	0.8613690936	0.2213570329	-3.367547993
O	0.4614301957	-0.5837021004	-2.2618267005
C	-0.2674245526	0.4162877736	-4.3763848595
H	-6.418927579	0.0713516657	4.5057349906
H	-5.3791565571	1.2228056754	0.4881074023
H	-3.6858343959	-2.1371540298	4.6487381247
H	-7.5579658288	1.5691449072	3.1996663336
H	-3.1186645595	0.5007743956	-0.3665294312
H	-3.4649516647	-1.2279145697	-0.344454451
H	-1.3565349701	0.1884648023	1.3430287045
H	-1.0808307205	-1.9486971531	-0.8278877031
H	0.2251901358	-1.3249521222	0.1691498975
H	-1.3770218536	0.2756710822	-1.8792787744
H	1.0247756501	1.0469950082	-0.1666337076
H	-0.5729345435	1.8004120931	-0.1155903112
H	1.0749353897	3.1377282286	-1.488504195
H	-0.3228313735	2.6288233922	-2.4189951272
H	1.7334772577	2.1675549941	-3.736618314
H	2.4303484691	1.2905631159	-2.3743212935
H	1.6379408379	-0.3806653597	-3.8503296707
H	-1.060021456	1.0733228871	-4.007571187
H	-0.7141336157	-0.5500538816	-4.6252035525
H	0.1247177973	0.8598964824	-5.2966728706

calc_**1a** conf_15

C	-5.7652652314	-0.7110813385	2.2179141988
C	-5.7252099573	0.6682988225	2.3767283573
C	-4.5050535319	1.3544500149	2.4873465157
C	-3.325432795	0.6423686406	2.4288773054
C	-3.3372117081	-0.7544447318	2.2507881848
C	-4.5742640201	-1.4375628701	2.1628248655
O	-4.6465667471	-2.7591116945	2.0261200264
O	-6.8471624612	1.4175854827	2.4374243364
C	-1.9800395477	1.2864001892	2.6008583669
C	-0.9181958711	0.5548084438	1.7892401171
O	-0.9285151425	-0.8521052207	2.1164748173
C	-2.0959235278	-1.5128822613	2.1637005884
O	-2.0581437464	-2.7396273404	2.1363576021
C	-1.0370493366	0.7677327722	0.2835453734
C	0.1188315004	0.1713770251	-0.540503847
C	-0.0213741532	-1.3131512053	-0.8849311985
C	-1.0786523263	-1.5418694808	-1.9627993486
C	-0.7924710736	-0.6479664135	-3.1663332244
C	-0.6488099901	0.8071876781	-2.7307721779
O	0.3425561071	0.9393560826	-1.7177794308
C	-0.2254390915	1.7267860552	-3.8591473435
H	-6.7051122506	-1.2491405312	2.1442943724
H	-4.5162445126	2.4298082603	2.622071466
H	-3.7134525349	-3.0951072258	2.0523026175
H	-7.6190666321	0.8425621558	2.3518293925
H	-1.6988547074	1.2492104528	3.6611291577
H	-2.0062109685	2.3412815425	2.3137030039

H	0.0737295959	0.8781588678	2.1165079572
H	-2.0126392424	0.4071043127	-0.0589522329
H	-1.0206514772	1.8502261258	0.1109706198
H	1.0404798662	0.3122825593	0.0366016618
H	-0.2369342914	-1.8928660816	0.0159612163
H	0.9486855894	-1.6543516239	-1.2658520133
H	-2.0804411493	-1.3220706938	-1.5704421957
H	-1.0919695702	-2.5963440006	-2.2571548426
H	-1.5861565211	-0.7300462673	-3.9179218433
H	0.1448235723	-0.9600561701	-3.6449808099
H	-1.6206634075	1.1509427483	-2.3372652134
H	-0.1274582597	2.7543523016	-3.4996139109
H	-0.9602513735	1.7082165921	-4.669265546
H	0.7427809258	1.4112880251	-4.2588569417

calc_**1a** conf_16

C	-5.8528468508	-0.8767026667	2.6097247782
C	-5.9175756005	0.5068038721	2.7156381648
C	-4.7606137072	1.2989111158	2.6406352896
C	-3.5392272393	0.6883093806	2.447353547
C	-3.4477874955	-0.7106210503	2.3168620327
C	-4.6184055071	-1.5002434241	2.4189134003
O	-4.5873018038	-2.8281153889	2.3374021005
O	-7.0856368706	1.159287899	2.9006034389
C	-2.2465632717	1.4518824365	2.4192222262
C	-1.2389881394	0.7825749785	1.4928742156
O	-1.0809386375	-0.6075431089	1.8529738937
C	-2.1656468156	-1.3630466663	2.0850066458
O	-2.0194521691	-2.5818506176	2.0967614087
C	-1.571453685	0.9383520104	0.014724098
C	-0.5264360684	0.3798994801	-0.9508007093
C	-0.635791394	-1.1206469858	-1.2075801887
C	0.3419274161	-1.5483362957	-2.3001397796
C	0.1347030379	-0.6924865678	-3.547425869
C	0.1827468848	0.8009710494	-3.2151883189
O	-0.7169651403	1.1126858911	-2.1550655405
C	1.5915334263	1.322444758	-2.9418168749
H	-6.7416128076	-1.495891786	2.6800041289
H	-4.8513764333	2.374290987	2.7402605484
H	-3.6320071958	-3.0808972748	2.2492354678
H	-7.8070127752	0.5171368687	2.9332859276
H	-1.8272298436	1.4821542161	3.4330360358
H	-2.4060264626	2.4880577757	2.1080204313
H	-0.2452991909	1.1994033456	1.6780102629
H	-1.6448932501	2.0125461366	-0.1885810116
H	-2.555059524	0.5087885995	-0.2061471744
H	0.4764145736	0.5967858181	-0.5441585879
H	-1.6627743876	-1.3383523094	-1.529871505
H	-0.4582715609	-1.6741658554	-0.2819352008
H	0.2053878333	-2.6080337673	-2.539543484
H	1.3712937336	-1.4462927338	-1.9334163267
H	0.8790679807	-0.9249377613	-4.3175154056
H	-0.8507198383	-0.9171547543	-3.9725899585
H	-0.2207127375	1.3632179267	-4.0639974818
H	1.5480128375	2.3742526296	-2.646086418
H	2.2027598561	1.2455896335	-3.8463414578
H	2.1037788231	0.7669722056	-2.1513467499

calc_**1a** conf_17

C	-5.9467511321	-0.2222408025	2.9318451372
C	-6.095565431	0.0277006989	1.5731809724
C	-5.0324392026	-0.1595262301	0.6773458514
C	-3.8117626376	-0.5875085188	1.1594486027
C	-3.6288366603	-0.8274008321	2.5337928143
C	-4.7178654558	-0.6650673312	3.4235202661
O	-4.6123965558	-0.920974638	4.7256579702
O	-7.2644059365	0.4558153582	1.0459138802
C	-2.6379729261	-0.845974965	0.2568540567
C	-1.3415102975	-0.5633291333	0.9913867048
O	-1.290186581	-1.3116544889	2.2296214691
C	-2.3453096765	-1.2855250369	3.0533635708
O	-2.1860330973	-1.6655999609	4.2096215134
C	-0.0528007369	-0.9431933285	0.274302488
C	0.2331373776	-0.1958065441	-1.0341523584
C	-0.3074575899	-0.8699979747	-2.294310351
C	-0.0113900054	-0.0033050431	-3.5171754237
C	-0.5362545186	1.4138716106	-3.2914436356
C	-0.023916547	2.0060948022	-1.9761369363
O	-0.2860706319	1.1182134475	-0.8905684098
C	1.4388988078	2.4383601176	-2.0208699611
H	-6.7696214988	-0.0951375178	3.6283196561
H	-5.1911539695	0.0361927114	-0.3768111352
H	-3.6962707622	-1.2762889215	4.8657381879
H	-7.9153199912	0.5507759067	1.7536249152
H	-2.6893455107	-0.206099947	-0.62649017
H	-2.6520916288	-1.8906125597	-0.0827035802
H	-1.3077666676	0.4989957969	1.2508708014
H	-0.0525994886	-2.022596958	0.0845816068
H	0.7564846813	-0.7518990657	0.9853137818
H	1.3280739619	-0.1444795748	-1.1431657527
H	-1.3884849925	-1.0244472265	-2.2045630974
H	0.1515439016	-1.8598823909	-2.3980746941
H	-0.4642787925	-0.4356383266	-4.4154663583
H	1.0711216333	0.017342786	-3.6966243446
H	-0.2632474724	2.071824456	-4.1239382381
H	-1.6320059429	1.3857657332	-3.2515613272
H	-0.6235586143	2.8888120831	-1.7315959152
H	1.5633788354	3.261531834	-2.7310189182
H	2.1131651981	1.6344927533	-2.3287304339
H	1.7538665547	2.7873972213	-1.0339032055

calc_**1a** conf_18

C	-5.9614232783	-0.1246225212	3.0356710715
C	-6.2929897444	-0.320554015	1.7008087831
C	-5.331100221	-0.7249354769	0.7623518646
C	-4.0281378623	-0.9169068504	1.1749850493
C	-3.6618043994	-0.7052424847	2.5168999578
C	-4.6473591942	-0.32939477	3.4602722764
O	-4.3650009828	-0.161917846	4.7502039807
O	-7.5505314155	-0.1396578066	1.2392804281
C	-2.94310813	-1.3856299967	0.2474282861
C	-1.6091844758	-0.7878748842	0.6622088773
O	-1.3409661595	-1.1377710696	2.0417960674
C	-2.288618742	-0.9165756802	2.96263542
O	-1.9601988129	-0.9191906011	4.1450149802

C	-0.4004307954	-1.2770945746	-0.1287867228
C	0.0143686653	-0.3940386178	-1.3032624243
C	-1.0022079756	-0.256059154	-2.4339468916
C	-0.458018345	0.6864900286	-3.506291062
C	-0.0737131817	2.0200174222	-2.8664728969
C	0.8636279996	1.8275985511	-1.6712151531
O	0.3259945958	0.8763934415	-0.7546073799
C	2.3000208185	1.4923300598	-2.0630697088
H	-6.7045696185	0.1712357218	3.7695401818
H	-5.6323348896	-0.8799931054	-0.2672622541
H	-3.4115100325	-0.4138331234	4.8615698384
H	-8.1194564972	0.1364209751	1.9698408801
H	-3.1755536186	-1.1216533478	-0.7869165971
H	-2.8661932811	-2.4808953023	0.2894023527
H	-1.6537598933	0.3056729441	0.6142198289
H	-0.5624643005	-2.3024211256	-0.4811484884
H	0.4432226778	-1.3032624552	0.5663994399
H	0.9250588197	-0.8476249981	-1.7269692169
H	-1.9345977678	0.1622790682	-2.0355484724
H	-1.2330661746	-1.242565678	-2.8527209634
H	-1.2013859643	0.8451704091	-4.294474214
H	0.4138004187	0.2278559378	-3.9900464373
H	0.3933094799	2.6879232193	-3.5986397381
H	-0.9846403491	2.517720782	-2.5129081237
H	0.8857512001	2.7554649631	-1.0907534195
H	2.3719795188	0.6121610745	-2.7080764755
H	2.8954852467	1.3061489832	-1.1654423422
H	2.7467066621	2.3328319034	-2.6029705823

calc_**1a** conf_19

C	-5.841627879	-0.1987831443	2.5892284915
C	-5.4803007121	1.1341051437	2.4399827648
C	-4.136546218	1.5124685478	2.2912999889
C	-3.1608589211	0.5375042062	2.2834755146
C	-3.5014001654	-0.8220443399	2.4111130429
C	-4.8571119479	-1.1888869135	2.5866110139
O	-5.2336278406	-2.4548479614	2.7499036395
O	-6.3926305738	2.1302521537	2.4367233846
C	-1.6968605418	0.8581572079	2.1970774685
C	-0.9413424688	-0.2440449391	1.4660947051
O	-1.2152238757	-1.5241523115	2.0777567416
C	-2.4784481091	-1.8608813309	2.3819115081
O	-2.7114164595	-3.0383254174	2.6359631258
C	-1.2199050234	-0.343193173	-0.0323416953
C	-0.6455735938	0.8107908717	-0.8693054476
C	0.8823650686	0.8183908079	-0.9811976109
C	1.3853105513	-0.2842229982	-1.9103424458
C	0.6612436893	-0.1961012716	-3.2513494166
C	-0.8507551636	-0.1954902131	-3.0477702025
O	-1.2407575973	0.8470811437	-2.1584612561
C	-1.6236181647	0.0346784137	-4.3312428802
H	-6.8771601453	-0.4971148397	2.7194303712
H	-3.8938395997	2.5639636248	2.1909053046
H	-4.4006998274	-2.9935708001	2.7699469322
H	-7.2758987162	1.7526487623	2.5413895055
H	-1.2882863843	0.9478532191	3.2117130062
H	-1.533581913	1.8213498283	1.7060469507
H	0.1307253853	-0.1188153741	1.633518704

H	-0.8160768681	-1.3034813844	-0.3667792679
H	-2.3012054357	-0.3711069765	-0.204909131
H	-0.9668133052	1.7556372295	-0.4170772547
H	1.3441380589	0.7377522273	0.0093531799
H	1.1813647169	1.7909159733	-1.3895567647
H	1.2125343282	-1.2705967551	-1.4613185225
H	2.4678792713	-0.1949091454	-2.0485769515
H	0.944152539	-1.028579554	-3.9054170715
H	0.9430419677	0.7328279467	-3.7638716208
H	-1.1479535193	-1.1682197112	-2.6234506016
H	-1.4013850899	-0.7496212985	-5.06050134
H	-1.355289726	1.0021926872	-4.7654991095
H	-2.6985597907	0.0344198581	-4.1344767536

calc_**1a** conf_20

C	-5.8224991423	-0.2662873471	2.5047814668
C	-5.4949338161	0.9962516178	2.0277402624
C	-4.1586447681	1.3714103267	1.8164549582
C	-3.1558697407	0.4612093921	2.0789298663
C	-3.4614021345	-0.8322648325	2.5419042062
C	-4.8105120217	-1.1893926705	2.7767974088
O	-5.1558275184	-2.3839539446	3.2505302608
O	-6.4350829205	1.9264230377	1.7506906334
C	-1.6986821384	0.7969840914	1.9461712391
C	-0.899046535	-0.4290493967	1.5256738066
O	-1.1492904679	-1.5216660971	2.4370311968
C	-2.4089861456	-1.8100399419	2.7975583763
O	-2.6165007842	-2.8889623658	3.3432091085
C	-1.1508990009	-0.9187084859	0.1026667852
C	-0.7433326412	0.0450974895	-1.0073980784
C	0.7394450608	0.411647889	-1.0048296368
C	1.0939698173	1.2252813358	-2.2490588125
C	0.6313578422	0.4886322283	-3.5043059
C	-0.8454562225	0.0977078276	-3.4110938421
O	-1.0943573553	-0.6240470209	-2.2080058045
C	-1.8060307103	1.270306718	-3.5905903138
H	-6.8525328035	-0.5579186355	2.6844377079
H	-3.942564549	2.3714873153	1.4588253466
H	-4.3089007885	-2.8708537067	3.4249174348
H	-7.3095093247	1.5581763331	1.9335934784
H	-1.3202697212	1.1447396399	2.9159398718
H	-1.5435323534	1.6127353962	1.2347159866
H	0.1659482541	-0.2328628131	1.6655160471
H	-2.212578397	-1.1526602768	-0.0267088279
H	-0.5989919023	-1.8541756481	-0.0356749457
H	-1.3346611758	0.9714777464	-0.9214045023
H	1.3263136173	-0.5158614974	-0.9864593742
H	0.991768669	0.975540097	-0.0990435827
H	2.1729254753	1.4071783284	-2.2915506409
H	0.617244381	2.2120917594	-2.1900006504
H	0.8010562566	1.0947328404	-4.4012132058
H	1.2214477555	-0.4283792148	-3.6168856542
H	-1.0649231149	-0.6344898889	-4.1948833576
H	-2.8348354499	0.9348406848	-3.4351672912
H	-1.7246412879	1.6691726279	-4.6063845523
H	-1.6081821973	2.0934490618	-2.8984264755

calc_**1a** conf_21

C	-5.8695402111	-0.1997215474	3.2145434085
C	-5.7512769287	1.0981961222	2.7334596497
C	-4.624316131	1.5049160121	2.0031784173
C	-3.6089986816	0.5987042017	1.7731338859
C	-3.6936004353	-0.7161210332	2.2676425543
C	-4.8501718641	-1.123091694	2.9749537106
O	-5.0031746974	-2.3659427309	3.4259758875
O	-6.7104841541	2.0291499821	2.935981009
C	-2.3898337737	0.9347353351	0.9632941518
C	-1.195708564	0.1584002931	1.4881190889
O	-1.4764433512	-1.2557101662	1.4927005816
C	-2.6248805554	-1.6826565651	2.0298517971
O	-2.7324507526	-2.8764241631	2.2954224361
C	0.1110205832	0.3631438099	0.7291486564
C	0.0327809098	0.4671819469	-0.7893327933
C	1.4224634663	0.6162165506	-1.4070093283
C	1.3392707303	0.5868255851	-2.9309827447
C	0.5866921829	-0.6642349093	-3.3785566251
C	-0.7708989636	-0.7843113665	-2.6818772397
O	-0.6219475384	-0.6935747897	-1.2662407266
C	-1.8198016378	0.1885371946	-3.2150065649
H	-6.7462441672	-0.5252615089	3.7657142498
H	-4.5755634027	2.5223197274	1.6325245261
H	-4.2127212531	-2.8759380653	3.1074233214
H	-7.4305324492	1.6356821218	3.4461799117
H	-2.1809744709	2.008602688	0.9952772856
H	-2.5570886729	0.6582031325	-0.0840056569
H	-1.0276926961	0.4404534016	2.5362736175
H	0.7778058909	-0.4618448178	1.0011885994
H	0.5744026239	1.2849531499	1.1000591965
H	-0.5566782549	1.3617840875	-1.0544287392
H	2.0484805968	-0.214361197	-1.0565087121
H	1.8860830084	1.5454905373	-1.0561172794
H	2.3422059517	0.6042818348	-3.3702663529
H	0.8284223735	1.4888539469	-3.290943878
H	0.4473119372	-0.6736373231	-4.4652931958
H	1.1821906475	-1.5481798153	-3.1218090481
H	-1.1523488533	-1.7993175981	-2.8305248082
H	-2.7571142532	0.0679301389	-2.6648596134
H	-2.0192872766	-0.0166402289	-4.2713186923
H	-1.5093569124	1.2344077197	-3.1369639438

calc_**1a** conf_22

C	-5.5704414552	0.1482927901	1.6128158626
C	-5.2346277469	1.4568986853	1.9354385566
C	-3.9179088812	1.8086117732	2.2671213414
C	-2.9402160523	0.8327441474	2.2592901192
C	-3.2492679948	-0.4946341883	1.9227814866
C	-4.5850650141	-0.8408920397	1.6091515287
O	-4.9426661917	-2.0866015221	1.3087841265
O	-6.151470946	2.4507859228	1.9531276032
C	-1.5258852258	1.1138962282	2.6742241541
C	-0.51519802	0.2040453574	1.9809940664
O	-0.9294348379	-1.1749283334	2.0204034031
C	-2.2146988672	-1.5199319438	1.8667758234
O	-2.4660248898	-2.712429649	1.7159818931
C	-0.0933132179	0.6159565013	0.568988925

C	-1.1662708349	0.6794678882	-0.526469378
C	-0.6524172109	1.3483528703	-1.8061806564
C	0.2948651659	0.4350501047	-2.5803922745
C	-0.3832602883	-0.9117860597	-2.8181608311
C	-0.8867372102	-1.5111332504	-1.5080152556
O	-1.7322867935	-0.5908394782	-0.8186392726
C	-1.7063557114	-2.7698238796	-1.712409705
H	-6.5889037754	-0.1334757566	1.3643539404
H	-3.6960666695	2.8368147685	2.5289764521
H	-4.1219750337	-2.6380396599	1.393345295
H	-7.0133922323	2.0895110477	1.708534045
H	-1.4452525087	0.9573816509	3.7575059564
H	-1.261588008	2.1596690322	2.4900908903
H	0.3976933926	0.1999415295	2.5831935308
H	0.3574889134	1.6124954902	0.6608435271
H	0.7186752227	-0.0520780316	0.2649847045
H	-2.0112373653	1.2646510003	-0.1502120192
H	-0.1757761199	2.3050898807	-1.5641745129
H	-1.5209171799	1.5727699201	-2.4363303088
H	1.2274660681	0.2880625548	-2.0205239155
H	0.5762435235	0.9001038458	-3.5312607359
H	0.3015485462	-1.6177068708	-3.3015434532
H	-1.2406897739	-0.777836682	-3.4904634874
H	-0.0211543193	-1.7541231319	-0.872282649
H	-2.5910788986	-2.5492265914	-2.3172688224
H	-2.0309966668	-3.1707169254	-0.7504326576
H	-1.1124048912	-3.530388996	-2.2279472963

calc_**1a** conf_23

C	-5.8153531164	-0.905058447	2.271835407
C	-5.7580109645	0.1913598777	1.4209133233
C	-4.5375083458	0.813473724	1.1150567706
C	-3.3726950571	0.3155667941	1.6632929163
C	-3.400773544	-0.8073597564	2.5116457839
C	-4.6406550628	-1.4075330937	2.8358336117
O	-4.7306165157	-2.4444868288	3.6652766558
O	-6.8656122051	0.7182879519	0.8526274522
C	-2.03114255	0.9491122473	1.4443900304
C	-0.9296270483	-0.0979429928	1.4447062371
O	-0.9914677909	-0.8759742439	2.6646625858
C	-2.171784451	-1.3502187765	3.0847004765
O	-2.1684605867	-2.2215679076	3.9497110479
C	-0.900345281	-1.0814962783	0.2724181961
C	-0.6402327485	-0.4629872253	-1.1090983538
C	-1.9026010596	-0.1416323857	-1.9076795971
C	-1.5353830357	0.5447740784	-3.2225184865
C	-0.6371091524	1.7496360429	-2.9499621774
C	0.5743925899	1.3697101343	-2.0953209734
O	0.1570552967	0.696185716	-0.9086812024
C	1.6432379673	0.5862625462	-2.8518654454
H	-6.7576656412	-1.380298544	2.5259914266
H	-4.5361859046	1.6799675986	0.4637726244
H	-3.8106299829	-2.6135730542	3.998683564
H	-7.6389161775	0.2152647498	1.1400168451
H	-1.832016968	1.6540061766	2.2622372501
H	-1.9887678852	1.5222747457	0.5169721813
H	0.0375555522	0.3991039162	1.501179726
H	-0.1070564921	-1.7974858833	0.5071466294

```
H  -1.8371066076  -1.6487812351  0.2384854844
H  -0.0670557174  -1.1989146247  -1.6945780843
H  -2.5661175205  0.5042574259   -1.321553159
H  -2.4525682217  -1.0711884266  -2.0937516259
H  -2.4376733702  0.85810904     -3.7578500639
H  -1.0212642268  -0.1700971175  -3.8776117069
H  -1.2137639821  2.5097089438   -2.4089849424
H  -0.3004112846  2.2092689438   -3.8858736645
H  1.0389352598   2.2860670838   -1.7168866618
H  2.0777165001   1.2106871917   -3.6384325372
H  1.2526366364   -0.3172581925  -3.3276237567
H  2.4430486956   0.2917700852   -2.1672837874
```

calc_**1a** conf_24

```
C  -5.9792167008  -0.7194278962  3.3468630007
C  -6.2107189118  0.5458894058   2.8218163626
C  -5.2654057462  1.1771502779   1.9988959048
C  -4.0777394905  0.5327889793   1.7188440648
C  -3.8088662167  -0.7413464428  2.2553796434
C  -4.7847328884  -1.3809654497  3.0585682217
O  -4.6042441564  -2.6023860332  3.5553536237
O  -7.3500835578  1.2284603928   3.067969247
C  -3.035216657   1.1261921761   0.8135518385
C  -1.6515227337  0.6622939547   1.2415051303
O  -1.5908844085  -0.7774579807  1.3137024253
C  -2.5620366301  -1.4382140346  1.963117594
O  -2.3560366002  -2.6084360836  2.2673321665
C  -0.4904056578  1.1257853984   0.3657541965
C  -0.1405464005  0.279276469    -0.8582578547
C  -1.2311319139  0.1385054395   -1.9152808192
C  -0.6826310819  -0.5721934792  -3.1527833546
C  0.5792810596   0.1287292629   -3.648596838
C  1.601850118    0.2991124949   -2.5223897787
O  1.0005658813   0.9357094086   -1.399152485
C  2.3175825164   -0.9932705527  -2.1383138841
H  -6.7126280318  -1.2206646646  3.9706272091
H  -5.4886016026  2.1593179216   1.5984918667
H  -3.7383515745  -2.9263661416  3.1960740619
H  -7.9192995463  0.7004886761   3.6432701656
H  -3.0696171183  2.2192822028   0.8493239916
H  -3.2349851712  0.8355239395   -0.2236290088
H  -1.484002271   1.033936584    2.2606762776
H  -0.6879179564  2.1529126433   0.0378647438
H  0.4180563466   1.1658946181   0.9738117743
H  0.1355783838   -0.7253669682  -0.5099168826
H  -1.5951403698  1.1372900217   -2.1909645294
H  -2.0732801226  -0.4274887808  -1.5048644088
H  -1.4389966855  -0.5973759911  -3.9443327778
H  -0.4634118431  -1.6176065744  -2.9026091294
H  0.3116726138   1.1247025784   -4.0214421738
H  1.0312604921   -0.4179533847  -4.4838958103
H  2.3626497218   1.0177855332   -2.844964136
H  2.9813542745   -0.8115435948  -1.2888165388
H  2.9233470176   -1.3504607146  -2.9769335079
H  1.62845362     -1.7965036111  -1.8636495924
```

calc_**1a** conf_25

C	-5.68044338	-0.4532429345	2.2736636696
C	-5.5480286316	0.8250336917	2.8004363497
C	-4.2894487515	1.4316522988	2.9319453106
C	-3.1648945766	0.7457578627	2.5189520017
C	-3.2736124414	-0.5424249857	1.9627671609
C	-4.5450752368	-1.1537121247	1.8607988164
O	-4.7013887219	-2.3857614718	1.3817377183
O	-6.6140155437	1.5454838309	3.2162236229
C	-1.7768167134	1.3018722149	2.6737671881
C	-0.8770814493	0.7967885583	1.5531056806
O	-0.9107328955	-0.6423394063	1.5068997218
C	-2.0916802617	-1.2759116064	1.5193195708
O	-2.1172333825	-2.4588192212	1.1988481451
C	-1.2185040546	1.4351204079	0.2041939693
C	-0.6320835718	0.7779589098	-1.0433813744
C	0.8439487523	0.3995185775	-0.9443236081
C	1.2815812196	-0.3350293802	-2.2099205203
C	0.3561347944	-1.5266985445	-2.4494028156
C	-1.114461144	-1.102782469	-2.4730154276
O	-1.4462484479	-0.3513382341	-1.3092400126
C	-1.5252332604	-0.3760788375	-3.7514327005
H	-6.6490838141	-0.9347132374	2.1827320235
H	-4.2262411451	2.4257621393	3.359349733
H	-3.788409127	-2.7310656307	1.2010749564
H	-7.4188516332	1.0297523803	3.075477754
H	-1.3618810256	0.9807437209	3.6375692481
H	-1.7901797284	2.3959973947	2.6785397155
H	0.166402709	1.0072991872	1.7980465973
H	-0.8737675126	2.4752131794	0.2540362624
H	-2.3044089825	1.4665195038	0.0682538038
H	-0.7414414707	1.5051589533	-1.8660143883
H	0.987953495	-0.2591026198	-0.0816817739
H	1.4460324215	1.3025668766	-0.7851329788
H	2.3188772977	-0.6736004484	-2.1168992169
H	1.2545275117	0.3503059603	-3.0670073075
H	0.6069683795	-2.0396541937	-3.384642764
H	0.4881399889	-2.2483700593	-1.6353844605
H	-1.7365251741	-1.998299395	-2.3806233893
H	-0.9136016609	0.5079218438	-3.9530556563
H	-2.5686650026	-0.0578526052	-3.678750321
H	-1.4295278282	-1.0456300873	-4.6118303045

calc_**1a** conf_26

C	-5.4276304476	0.0479316898	1.3697794818
C	-5.1186574388	1.3877192406	1.5706642129
C	-3.836734173	1.7834389604	1.9778173302
C	-2.8653372106	0.820193832	2.173917798
C	-3.1428572909	-0.5384098105	1.9534833261
C	-4.4476313557	-0.9258046545	1.5671525989
O	-4.7739218738	-2.2017414873	1.3635851577
O	-6.0306822251	2.3700500968	1.39250661
C	-1.5065523776	1.1574962024	2.7121951104
C	-0.4304203024	0.1652447773	2.2790476493
O	-0.8666378422	-1.1972518725	2.4331665221
C	-2.1180987688	-1.5635032746	2.1384528601
O	-2.3655365848	-2.7636532059	2.0797377522
C	0.1903519473	0.4040875044	0.9029286517
C	-0.668701723	0.3234613341	-0.3568689517

C	0.0817380405	0.9409672784	-1.539017758
C	-0.6310290238	0.6586535808	-2.8571716061
C	-0.8557121972	-0.8438976373	-2.9925937961
C	-1.6005906597	-1.406183706	-1.7806705811
O	-0.9340912045	-1.0543928721	-0.5668101247
C	-3.0824040557	-1.0472427905	-1.7574608351
H	-6.4206298284	-0.2685388338	1.0660058801
H	-3.6381409146	2.8356416757	2.1466178862
H	-3.965395224	-2.7320749958	1.5997408257
H	-6.8705941887	1.9762084159	1.1223701343
H	-1.5633648708	1.1511821171	3.8082635323
H	-1.2078392518	2.1695182971	2.4222467186
H	0.3940862413	0.2365572351	2.993883564
H	1.0063016247	-0.3158735601	0.7771360032
H	0.6496862475	1.400029717	0.9453361518
H	-1.6127026285	0.8727712995	-0.2151144622
H	1.0922932701	0.5122482237	-1.5722997774
H	0.1917224399	2.0190990489	-1.3735793489
H	-0.041618796	1.03658795	-3.6992729112
H	-1.5897854454	1.1916009322	-2.8836977229
H	-1.4106209874	-1.081752204	-3.9066739691
H	0.1174259754	-1.3442726165	-3.0653509564
H	-1.5222288219	-2.4976887377	-1.7994802641
H	-3.5588882705	-1.3732781557	-2.6876902737
H	-3.2551731624	0.0278555582	-1.6550632104
H	-3.5813966411	-1.5529845527	-0.9292192084

calc_**1a** conf_27

C	-5.5098594803	-0.2213091951	1.4827671242
C	-5.2544179095	1.1428858401	1.5453841515
C	-3.9865838125	1.630095684	1.8967073734
C	-2.9720468988	0.7338743277	2.1730889227
C	-3.2010056159	-0.6510653808	2.1061496358
C	-4.4917367573	-1.1311279824	1.7729539389
O	-4.7733609347	-2.4302604908	1.7224010454
O	-6.2052423695	2.0651909762	1.2799040183
C	-1.6139421406	1.1832581144	2.628412193
C	-0.5032775295	0.1904732567	2.2923994875
O	-0.8920830366	-1.166660292	2.5973721763
C	-2.1373153588	-1.6083756758	2.3911737085
O	-2.3335392286	-2.817793143	2.4663798909
C	0.126192383	0.2454001863	0.9006982848
C	-0.7419928577	0.0301155522	-0.3393271181
C	-1.3921126716	1.3002729008	-0.8824582952
C	-2.1000030648	1.0151244902	-2.2057176093
C	-1.1298520343	0.3554649064	-3.1830270386
C	-0.4424387225	-0.8600842533	-2.5557430568
O	0.1497132757	-0.5111518948	-1.3073943456
C	-1.3440595836	-2.0870699496	-2.4430353625
H	-6.4913869411	-0.6062302655	1.2245476197
H	-3.8306209602	2.701551305	1.9500138995
H	-3.9470563519	-2.9052023015	2.0027668109
H	-7.0309821083	1.6156083489	1.0568868385
H	-1.6459072843	1.3080201506	3.7183378877
H	-1.3625642968	2.1625687913	2.2103923558
H	0.3172147939	0.3668214476	2.9926506205
H	0.8914347268	-0.5365315276	0.880698236
H	0.6568997752	1.2017450837	0.8111891571

H	-1.5275683669	-0.7068844198	-0.1168451074
H	-0.6014826184	2.0458062265	-1.0410315994
H	-2.097233413	1.711751426	-0.1543818085
H	-2.497885376	1.9415040276	-2.6335152338
H	-2.9638027597	0.3633718597	-2.0247185378
H	-1.6393286161	0.0595954021	-4.1070410725
H	-0.3559364524	1.0815157561	-3.459540653
H	0.4173850785	-1.1355980961	-3.1753490988
H	-2.2754814009	-1.8867970061	-1.90610862
H	-0.8170255689	-2.8886172906	-1.9187990264
H	-1.6117075109	-2.4472568953	-3.4412417928

Table S28. Sampled and DFT geometry optimized conformers related to calc_**1b**, with energy values (Hartree) and related % contribution on the final Boltzmann distribution for the three employed functional/basis set combinations.

**Conformer**	**MPW1PW91/6-31g(d,p)**		**MPW1PW91/6-311+g(d,p)**		**B97-2/cc-pVTZ**	
	**Energy (Hartree)**	**% contribution on the Boltzmann distribution**	**Energy (Hartree)**	**% contribution on the Boltzmann distribution**	**Energy (Hartree)**	**% contribution on the Boltzmann distribution**
calc_**1b** conf_1	-997.7082456	19.24%	-997.9430329	22.99%	-997.9016822	22.15%
calc_**1b** conf_2	-997.7082366	19.06%	-997.9426204	14.85%	-997.9010254	11.05%
calc_**1b** conf_3	-997.7080968	16.44%	-997.9428186	18.32%	-997.9017245	23.16%
calc_**1b** conf_4	-997.7075007	8.74%	-997.9415396	4.73%	-997.9000598	3.97%
calc_**1b** conf_5	-997.7072530	6.73%	-997.9418359	6.47%	-997.9003408	5.35%
calc_**1b** conf_6	-997.7072304	6.57%	-997.9422580	10.12%	-997.9012476	13.98%
calc_**1b** conf_7	-997.7069103	4.68%	-997.9416668	5.41%	-997.8997560	2.88%
calc_**1b** conf_8	-997.7068459	4.37%	-997.9414214	4.17%	-997.8997007	2.72%
calc_**1b** conf_9	-997.7066271	3.47%	-997.9406073	1.76%	-997.8995792	2.39%
calc_**1b** conf_10	-997.7064987	3.03%	-997.9413636	3.92%	-997.9004836	6.22%
calc_**1b** conf_11	-997.7064751	2.95%	-997.9409618	2.56%	-997.8994905	2.17%
calc_**1b** conf_12	-997.7056865	1.28%	-997.9399959	0.92%	-997.8980080	0.45%
calc_**1b** conf_13	-997.7052806	0.83%	-997.9402889	1.26%	-997.8986986	0.94%
calc_**1b** conf_14	-997.7045734	0.39%	-997.9389238	0.30%	-997.8970238	0.16%
calc_**1b** conf_15	-997.7045361	0.38%	-997.9385410	0.20%	-997.8965329	0.09%
calc_**1b** conf_16	-997.7044740	0.35%	-997.9390994	0.36%	-997.8978334	0.38%
calc_**1b** conf_17	-997.7043919	0.32%	-997.9389223	0.30%	-997.8977294	0.34%
calc_**1b** conf_18	-997.7043755	0.32%	-997.9392081	0.40%	-997.8978188	0.37%
calc_**1b** conf_19	-997.7041880	0.26%	-997.9384613	0.18%	-997.8968367	0.13%
calc_**1b** conf_20	-997.7035032	0.13%	-997.9385881	0.21%	-997.8976405	0.31%
calc_**1b** conf_21	-997.7034597	0.12%	-997.9377278	0.08%	-997.8974459	0.25%
calc_**1b** conf_22	-997.7030853	0.08%	-997.9380240	0.11%	-997.8967727	0.12%
calc_**1b** conf_23	-997.7029295	0.07%	-997.9378796	0.10%	-997.8969067	0.14%
calc_**1b** conf_24	-997.7027131	0.05%	-997.9376709	0.08%	-997.8968085	0.13%
calc_**1b** conf_25	-997.7023620	0.04%	-997.9373878	0.06%	-997.8955501	0.03%
calc_**1b** conf_26	-997.7023246	0.04%	-997.9373578	0.06%	-997.8963432	0.08%
calc_**1b** conf_27	-997.7018829	0.02%	-997.9369733	0.04%	-997.8941580	0.01%

calc_**1b** conf_28	-997.7011556	0.01%	-997.9360458	0.01%	-997.8944661	0.01%
calc_**1b** conf_29	-997.7011477	0.01%	-997.9361415	0.02%	-997.8947181	0.01%
calc_**1b** conf_30	-997.7009035	0.01%	-997.9359564	0.01%	-997.8943459	0.01%
calc_**1b** conf_31	-997.6997696	0.00%	-997.9345213	0.00%	-997.8926399	0.00%
calc_**1b** conf_32	-997.6993183	0.00%	-997.9344931	0.00%	-997.8923420	0.00%
calc_**1b** conf_33	-997.6991047	0.00%	-997.9343415	0.00%	-997.8923284	0.00%

Table S29. Cartesian coordinates of the optimized geometries for the conformers related to calc_**1b**. The related energies and % contribution on the final Boltzmann distribution for the three employed functional/basis set combinations are reported in Table S28.

calc_**1b** conf_1

C	-7.4862612556	-1.2758579582	-0.3769165576
C	-7.1823774215	-0.017648539	-0.8819287549
C	-5.9783307725	0.6260274706	-0.5581920551
C	-5.0746634381	-0.0103853821	0.2681769084
C	-5.3490086987	-1.2939938063	0.7769069794
C	-6.5805616351	-1.9203705952	0.4668597494
O	-6.9105385857	-3.1142731991	0.954529194
O	-8.0251622235	0.650075487	-1.7003736322
C	-3.7833178698	0.6319165625	0.6909127599
C	-2.7218160544	-0.4281933726	0.919217264
O	-3.1928143206	-1.4347005492	1.8417599496
C	-4.4054086653	-1.9748342259	1.6558125642
O	-4.6700866987	-3.0154227878	2.2501277223
C	-1.4225460226	0.0834829537	1.5183882456
C	-0.7388889097	1.159627862	0.6676232164
C	0.6548360216	1.5307483884	1.1806494653
C	1.6915760892	0.4639133764	0.8333349458
C	1.6264955337	0.146996986	-0.6587818107
C	0.2034881525	-0.2196199188	-1.0673571878
O	-0.7063020315	0.8186817493	-0.7130068423
C	0.0472887211	-0.4287440908	-2.5603699455
H	-8.4224335713	-1.7731168843	-0.6106126284
H	-5.7820352982	1.6115268258	-0.9643004927
H	-6.1776260189	-3.376778165	1.5698711867
H	-8.8127342479	0.111410555	-1.8528019778
H	-3.4354523692	1.3299662559	-0.0748022623
H	-3.9372197348	1.2025810471	1.61724611
H	-2.5185117914	-0.930064206	-0.0334808083
H	-1.632298266	0.5028391382	2.5093942351
H	-0.767286442	-0.7779632292	1.6822045716
H	-1.3644240536	2.0582701397	0.6957271171
H	0.6160421142	1.7075955037	2.2613360667
H	0.94686071	2.4765912787	0.7096842994
H	1.5076734018	-0.4505885355	1.4113081966
H	2.6927797581	0.8080979956	1.11281811
H	2.3053318703	-0.6739540877	-0.9156105893
H	1.9388744515	1.024089345	-1.2401662897
H	-0.0809453327	-1.148161503	-0.5442932754
H	0.3145149308	0.4847481132	-3.099483949
H	-0.9873330576	-0.6805013823	-2.8072541843
H	0.694623032	-1.2400146159	-2.905155614

calc_**1b** conf_2

C	-7.6455820726	-0.8334521337	0.0468593176
C	-7.2103107966	-0.7227126526	-1.268180886
C	-5.8443633544	-0.73689791	-1.58788376
C	-4.9157119853	-0.8781456406	-0.5764537345
C	-5.3316786328	-1.0131976675	0.7625865814
C	-6.711907538	-0.9646585681	1.075545581
O	-7.1577995716	-1.0412482894	2.3278700503
O	-8.0729511872	-0.5905741736	-2.3002095653
C	-3.4360043722	-0.8570135486	-0.8301230265

C	-2.73054578	-1.7413052392	0.1827118024
O	-3.0715669874	-1.3433688739	1.5288101463
C	-4.3626059732	-1.1561293366	1.8423578885
O	-4.6639401875	-1.1041170545	3.030855802
C	-1.2100897095	-1.7652104003	0.1188169023
C	-0.4933904122	-0.4558328407	0.4230096574
C	0.9980804176	-0.6782860138	0.6656548276
C	1.7246396529	0.655133998	0.8255951025
C	1.4135279072	1.5618216193	-0.3632874521
C	-0.0943719622	1.696557693	-0.5859798927
O	-0.6973955093	0.4069833315	-0.6905619732
C	-0.7899348884	2.5816168825	0.4448668029
H	-8.7002064216	-0.8062753764	0.302457433
H	-5.5448720097	-0.6341687288	-2.6244814294
H	-6.3544267544	-1.0664334379	2.9092559018
H	-8.9772287364	-0.5948242476	-1.9598208917
H	-3.2051242624	-1.2070399501	-1.8404301913
H	-3.0448374839	0.1626282792	-0.7571094336
H	-3.0948802959	-2.7700597565	0.0582349223
H	-0.8656966407	-2.5159180149	0.8383872353
H	-0.9077108546	-2.1080656491	-0.877225172
H	-0.9395448235	-0.0137016239	1.3255117
H	1.4137776358	-1.2257339138	-0.1907104725
H	1.1352048471	-1.3062202198	1.5531116471
H	2.8049147802	0.4958068277	0.9082521209
H	1.4106681273	1.1342296389	1.7612721964
H	1.8554518311	1.1277367699	-1.268065904
H	1.8593838673	2.5538636771	-0.2303103483
H	-0.2651447408	2.1370768542	-1.5738098336
H	-0.6314596004	2.2441342899	1.4724912774
H	-1.8671762878	2.6028602003	0.2588233021
H	-0.4121892339	3.6061412003	0.3723057683

calc_**1b** conf_3

C	-7.5365478045	-1.2026820033	-0.4196827823
C	-7.0519101815	-0.0779044448	-1.0755216093
C	-5.7919360579	0.4576302232	-0.76886179
C	-5.0154810511	-0.1546565342	0.1942009745
C	-5.474538399	-1.3068793135	0.8594677661
C	-6.7603835309	-1.819513578	0.5629174393
O	-7.2625784065	-2.8796201181	1.1919433745
O	-7.7642899893	0.558626108	-2.0317609464
C	-3.6723706015	0.3793544256	0.6054172497
C	-2.7675155235	-0.7598504465	1.0377162623
O	-3.4052070596	-1.54646078	2.0687023941
C	-4.6667313823	-1.957865255	1.8853625041
O	-5.0922337437	-2.8570677689	2.6040740256
C	-1.4355641256	-0.3322285762	1.6309232941
C	-0.5485354205	0.4433364038	0.6679084888
C	0.8201435022	0.7542942999	1.2668042028
C	1.7209052271	1.4111803678	0.2234106454
C	1.7737068609	0.5417231529	-1.0316626444
C	0.3716309679	0.2039696508	-1.5453757604
O	-0.426159303	-0.3522841291	-0.5015293993
C	-0.3253205061	1.3710264265	-2.2392459909
H	-8.5169553751	-1.6129850926	-0.6404707422
H	-5.4540860233	1.3430821539	-1.2948767526
H	-6.5979869287	-3.1337534091	1.8839737194

H	-8.6059879029	0.1010466979	-2.157213588
H	-3.2069358258	0.9213449962	-0.2220618851
H	-3.7914860969	1.0877564104	1.4369262704
H	-2.5881720671	-1.4209906309	0.1840733357
H	-1.6183627359	0.2772408564	2.5234254585
H	-0.908357991	-1.2349051571	1.9569816664
H	-1.0380238982	1.3988612625	0.4152205776
H	1.2722850616	-0.1858183663	1.6071903731
H	0.7012640784	1.3973592186	2.1462039387
H	2.7291197948	1.5585013473	0.6242791908
H	1.336097969	2.4097350611	-0.0197422804
H	2.2861351517	-0.3974120653	-0.7921230422
H	2.3498212768	1.0297732115	-1.8256175929
H	0.4506881565	-0.6144811481	-2.2680892539
H	0.2279931942	1.6574555782	-3.1387797585
H	-0.4011425782	2.2580741182	-1.6041964049
H	-1.3349907316	1.0779868466	-2.5403109284

calc_**1b** conf_4

C	-7.6347482852	-0.9970501824	-0.0129540801
C	-7.1438201099	-0.4741826556	-1.203040416
C	-5.7686350624	-0.279259466	-1.4014178162
C	-4.8862644622	-0.6262554714	-0.3984858924
C	-5.3565567159	-1.1769206691	0.809608557
C	-6.7489873675	-1.3395164641	1.0095745647
O	-7.2508404475	-1.8134627616	2.1479983941
O	-7.9583598876	-0.1234705307	-2.222784462
C	-3.4050220516	-0.4079070735	-0.5145558867
C	-2.6663423138	-1.4840242999	0.2618810248
O	-3.1181668844	-1.5168923779	1.6330282554
C	-4.4370217333	-1.5418388808	1.8805832471
O	-4.8049489656	-1.863585326	3.0058148323
C	-1.1507118141	-1.3546756531	0.3196635081
C	-0.6244462848	-0.0871160661	1.0069327275
C	0.8500811546	-0.1893556516	1.4044078872
C	1.7714688989	-0.0628242283	0.1938574569
C	1.4333261868	1.2157669016	-0.5686991107
C	-0.0512758669	1.259663547	-0.9177038351
O	-0.8566613164	1.0953076839	0.243806843
C	-0.472498603	2.5756724202	-1.5422040298
H	-8.6985007069	-1.1352455015	0.1531701298
H	-5.4267298067	0.14391407	-2.3388503768
H	-6.4822424568	-1.9366491898	2.7627847612
H	-8.874541868	-0.3011603005	-1.9723797047
H	-3.0887857638	-0.4342691963	-1.5620754514
H	-3.1277438448	0.5773882986	-0.1247855207
H	-2.9143063219	-2.4615330987	-0.1734830727
H	-0.7759615339	-2.2236748203	0.8718112088
H	-0.7614836134	-1.4458146434	-0.6998706789
H	-1.2200561462	0.0615892831	1.9121537134
H	1.0260293404	-1.1256156179	1.9447295071
H	1.0671976057	0.628303041	2.1017358529
H	1.6535299439	-0.9311492559	-0.467790274
H	2.8191737007	-0.0570472484	0.5122633979
H	2.0268145043	1.2975775446	-1.4863939719
H	1.6698140321	2.0895301652	0.0521576325
H	-0.2626535205	0.445245908	-1.63130988
H	0.0873513804	2.7616399668	-2.4633223685

H -0.2850264254 3.3992652902 -0.8472230931
H -1.5394465675 2.5666325107 -1.7796335802

calc_**1b** conf_5

C	-7.5032824587	-1.7218929886	-0.2114660432
C	-7.6332800334	-0.4115023576	-0.6545691829
C	-6.6242154968	0.5374322855	-0.4298474466
C	-5.4759030653	0.1557692196	0.233536374
C	-5.3106733912	-1.1705086829	0.6756977972
C	-6.3484678483	-2.1111375305	0.4687504384
O	-6.2635200411	-3.3664917525	0.9033288807
O	-8.7311638531	0.0172329262	-1.3154287866
C	-4.3673611717	1.1204805427	0.5460080788
C	-3.0294017432	0.4012574315	0.5656911701
O	-3.0857632046	-0.7190272708	1.4787209743
C	-4.1074135771	-1.5825338856	1.3880760249
O	-3.9868242099	-2.6774076215	1.9294922835
C	-1.8821217805	1.2916390758	1.0144046124
C	-0.4828123025	0.8096166822	0.6381780275
C	-0.0323843148	-0.4922015845	1.2944021212
C	1.3452815728	-0.886894359	0.7672386291
C	1.3119705441	-0.9488524955	-0.7591968621
C	0.7708003247	0.3473697606	-1.3672043874
O	-0.4793875664	0.700783796	-0.7787941313
C	1.7633705352	1.5064491404	-1.3290712342
H	-8.2865203703	-2.4569255031	-0.3681234081
H	-6.7680539041	1.5523257027	-0.7820666718
H	-5.4108651252	-3.430697112	1.4066980825
H	-9.350412216	-0.7181357567	-1.412612244
H	-4.3367737617	1.9270875971	-0.192128096
H	-4.5431574028	1.5849109634	1.5261929935
H	-2.8121681232	0.0013931198	-0.4306352156
H	-2.0231024745	2.2715417685	0.545099837
H	-1.9462590966	1.4358277052	2.0985236261
H	0.2175421311	1.6041645205	0.9450204753
H	-0.7568244298	-1.2819445912	1.0753297724
H	-0.0185571246	-0.3674705163	2.3829097265
H	1.6496602754	-1.8554832073	1.177113117
H	2.0953949836	-0.1590195599	1.1033487001
H	0.6548551603	-1.7716523287	-1.0648422885
H	2.3057547414	-1.1608909089	-1.1693688247
H	0.5159316898	0.1631109021	-2.4161657376
H	1.2939492964	2.4114953373	-1.7241118466
H	2.6354842579	1.2744011185	-1.9480880682
H	2.1246745746	1.7233804176	-0.3200412674

calc_**1b** conf_6

C	-7.7837670923	-1.6172330331	0.003169464
C	-7.9071750404	-0.3050508298	-0.436195352
C	-6.8293811186	0.5914364652	-0.3665504127
C	-5.6214552088	0.1540774911	0.136483536
C	-5.4633565537	-1.1767161885	0.5695764659
C	-6.5672317946	-2.0623232421	0.5228657157
O	-6.4881295942	-3.3172001412	0.9591152483
O	-9.0623639094	0.1762639573	-0.9447177244
C	-4.4310408819	1.0588510292	0.2819101822
C	-3.1486827449	0.2646012099	0.1053685781

O	-3.1196205753	-0.8492237214	1.0230279745
C	-4.1931015003	-1.6507461331	1.1058019363
O	-4.0546110821	-2.7444879993	1.6434921989
C	-1.8972134465	1.080313695	0.3624934955
C	-0.5850335926	0.3146151098	0.2310536164
C	-0.327031548	-0.2468149651	-1.1645509882
C	1.0779674519	-0.842152918	-1.2466341463
C	2.1085866106	0.1855412266	-0.7844707724
C	1.7510168876	0.7603776528	0.5887428484
O	0.4159598987	1.2581391547	0.5873034253
C	2.0056369276	-0.20173995	1.7459418449
H	-8.6178505529	-2.3109569266	-0.0326260589
H	-6.9694318365	1.6105974455	-0.7077801605
H	-5.5789248752	-3.4245297283	1.3409083139
H	-9.7270275842	-0.5251210372	-0.9430542937
H	-4.4645953875	1.870743239	-0.4504420439
H	-4.4324875305	1.5211634496	1.2780576296
H	-3.13121328	-0.1555531454	-0.9083670738
H	-1.8820096687	1.9230109587	-0.3387527975
H	-1.9437381809	1.5031096418	1.3717939433
H	-0.5937494532	-0.5158256983	0.9508892253
H	-0.4278325489	0.5667925458	-1.8955469772
H	-1.0759086121	-1.0080720904	-1.409754232
H	1.2972643007	-1.1660198145	-2.2695268356
H	1.1298325336	-1.7412727017	-0.6203560622
H	2.1349574025	1.0121998485	-1.5045476255
H	3.1139159495	-0.2497188867	-0.756406234
H	2.3564301333	1.6564539945	0.7615121046
H	1.6733311591	0.2507574782	2.684061376
H	3.0757966976	-0.4155499793	1.8280418024
H	1.4862692419	-1.1567364633	1.6286688655

calc_**1b** conf_7

C	-7.3937360349	-0.2741995704	0.6991464761
C	-7.0559208475	0.3183817752	-0.5108388602
C	-5.8219193931	0.0611354751	-1.1291729982
C	-4.9257388203	-0.7885332994	-0.5139011968
C	-5.233730378	-1.3835757689	0.7245987767
C	-6.4923713321	-1.1392896149	1.3233560848
O	-6.8527521841	-1.7056919731	2.4725810165
O	-7.891373982	1.1620138594	-1.1561120058
C	-3.6101749577	-1.1708326321	-1.1271664835
C	-2.5613353761	-1.391671596	-0.0478952845
O	-3.0344314008	-2.3627454639	0.9132245231
C	-4.2791117486	-2.2581237226	1.399471573
O	-4.564395526	-2.9154458768	2.396027907
C	-2.1184001209	-0.1366955316	0.6992057087
C	-1.4086407923	0.8858116266	-0.195397917
C	-0.8342417033	2.0684453021	0.58921169
C	0.4399948391	1.6872814864	1.3393935578
C	1.4248175586	1.0297569075	0.3758438393
C	0.7672465759	-0.1376141186	-0.3527627388
O	-0.4186479697	0.2848269389	-1.0222111669
C	1.6573782609	-0.7509115625	-1.4155025067
H	-8.3523642707	-0.0923695758	1.1748244154
H	-5.6023094846	0.5283989556	-2.0821940915
H	-6.1117316817	-2.3151488691	2.7270419863
H	-8.7041935185	1.258648001	-0.6426069492

H	-3.73287376	-2.1062379263	-1.6878669915
H	-3.2621152377	-0.4213198047	-1.8420559614
H	-1.6856370257	-1.8689071797	-0.4890969269
H	-2.9877452298	0.3503225172	1.1554558658
H	-1.4734895091	-0.4581322862	1.5232926988
H	-2.1411501593	1.2758704226	-0.9101812028
H	-1.5944697848	2.4623867364	1.2727626065
H	-0.5994184707	2.8651985349	-0.1261668445
H	0.2093090092	0.9956530379	2.1594759178
H	0.8845864173	2.5749651165	1.801241354
H	2.3157451854	0.6747145115	0.9059018385
H	1.7586498127	1.7615509216	-0.3711781958
H	0.5116885126	-0.9119478089	0.3898458775
H	2.581717888	-1.1303815061	-0.9708810301
H	1.9142853897	-0.0022227046	-2.170491544
H	1.1470012505	-1.5783637341	-1.9152228178

calc_**1b** conf_8

C	-7.2849970551	-0.1201415231	0.3710432166
C	-6.7125644842	0.3182212859	-0.8161153761
C	-5.4354594878	-0.1099870577	-1.2114190385
C	-4.7343358129	-0.9751615749	-0.3964289338
C	-5.281858463	-1.4162362881	0.8231568855
C	-6.5817432519	-0.9999145932	1.1968531961
O	-7.164568614	-1.4165191755	2.3184065196
O	-7.3513464363	1.1683849596	-1.6506074601
C	-3.3908472857	-1.534814745	-0.7640229323
C	-2.5412040474	-1.754426208	0.4777895261
O	-3.2537481642	-2.5787595496	1.4284013154
C	-4.5364030091	-2.3097706387	1.7056690925
O	-5.0355828802	-2.8391244943	2.6944008746
C	-2.0762064811	-0.485050262	1.1879274501
C	-1.1725344545	0.399549007	0.3405030703
C	-0.6093872571	1.5788535902	1.1286649555
C	0.3869902495	2.3594156663	0.2744372153
C	1.4524426886	1.4099008503	-0.270060833
C	0.8282976689	0.21029312	-0.9875568148
O	-0.1313874249	-0.4325873425	-0.1500971647
C	0.2549403526	0.5461505321	-2.3616209183
H	-8.2784644484	0.1947140472	0.6748160149
H	-5.0305336187	0.2400794962	-2.1539573549
H	-6.5374059048	-2.0650299944	2.7332230967
H	-8.2159989341	1.3871421337	-1.2789312528
H	-3.5287241588	-2.501109004	-1.2658972562
H	-2.8635637381	-0.8871697041	-1.4688030627
H	-1.6646781383	-2.3482039331	0.2210252643
H	-2.9420825726	0.1036988848	1.5104958286
H	-1.5350514575	-0.7930342954	2.0887603466
H	-1.7550290815	0.8006014179	-0.5054319419
H	-0.1062797637	1.1917712856	2.0237822254
H	-1.4298844821	2.222052017	1.4660678186
H	0.8549684601	3.1565236349	0.8614003077
H	-0.1423565877	2.8530549674	-0.5504433283
H	2.0531261894	1.0332978685	0.5661955693
H	2.1363876127	1.9314131613	-0.9487654353
H	1.5989096784	-0.5549774884	-1.1257683979
H	1.0552565718	0.8710202711	-3.0334105141
H	-0.4898892997	1.3462386792	-2.3273050711

H -0.2152026767 -0.340359004   -2.7963767031

calc_**1b** conf_9

C	-7.5543445892	-1.5259585858	0.1698284815
C	-7.7575476651	-0.1942377181	-0.1699603358
C	-6.7045187844	0.7332936403	-0.1454956796
C	-5.4410396619	0.3088478876	0.2116743267
C	-5.2045634339	-1.0390161961	0.5430350222
C	-6.2808899406	-1.95865473	0.542556015
O	-6.1227525604	-3.2340476259	0.8886545293
O	-8.9701013076	0.276875577	-0.5348785022
C	-4.2682115369	1.2453978673	0.2915859801
C	-2.9814722379	0.5053450585	-0.0338272925
O	-2.8489513616	-0.6427803223	0.8298094499
C	-3.8774576801	-1.4943285844	0.9391436878
O	-3.6552667669	-2.613361138	1.3917896534
C	-1.7272783071	1.347150012	0.1524214972
C	-0.5409263733	0.8873969164	-0.7089135752
C	0.7405237715	1.6821038019	-0.4369471383
C	1.4250545976	1.2327220359	0.8532098255
C	1.5993520597	-0.284733749	0.8457464038
C	0.2597503692	-0.9675317573	0.592793216
O	-0.3080612161	-0.5054933522	-0.632820411
C	0.3536737183	-2.4741524824	0.4716077563
H	-8.367443123	-2.2450722653	0.167687592
H	-6.9061765015	1.7653715052	-0.4081741438
H	-5.1781264198	-3.3318801985	1.1750572802
H	-9.6100465626	-0.4469628265	-0.5189613907
H	-4.3948658877	2.084000352	-0.3996414698
H	-4.1942683861	1.6683693772	1.3028139348
H	-3.0303752278	0.1267281414	-1.062742621
H	-1.9557182443	2.3824477743	-0.1284196938
H	-1.4823246914	1.3629071192	1.2196317822
H	-0.8316635743	1.0449441912	-1.7550111533
H	0.5199071927	2.7558343076	-0.4148685576
H	1.4241209341	1.5128436061	-1.2770763757
H	0.8246074202	1.5254644137	1.7244857631
H	2.3911473513	1.7373651462	0.9610191396
H	2.0165745816	-0.6338148692	1.7971263315
H	2.3005947344	-0.577819055	0.053666588
H	-0.4235542437	-0.7258973591	1.4188859203
H	0.7963444838	-2.9042014246	1.3748210108
H	0.9735652589	-2.7491951669	-0.3871981804
H	-0.643270188	-2.900269324	0.3388853336

calc_**1b** conf_10

C	-7.823636073	-1.5479135926	0.0553008187
C	-7.7432769478	-0.4308121699	-0.7666366645
C	-6.5723804295	0.3402380919	-0.8360265882
C	-5.4778723093	-0.0287096411	-0.0814954463
C	-5.5258127262	-1.1681243563	0.7444777395
C	-6.7228950709	-1.9195878641	0.8289475794
O	-6.8399213785	-2.9790527936	1.6255974173
O	-8.7797986512	-0.0264843814	-1.5326213417
C	-4.2015823443	0.7636643637	-0.0680182628
C	-3.0166162517	-0.1585886682	0.1623734431
O	-3.2102809604	-0.9281040478	1.3666275982

C	-4.3777501942	-1.5652840255	1.5508270164
O	-4.4249820239	-2.4455792525	2.4030949394
C	-1.7001206808	0.5809034532	0.3183167295
C	-0.4852968711	-0.3242233836	0.5734440971
C	-0.0459790067	-1.1587476022	-0.6323326836
C	0.6569099336	-0.3070800267	-1.6879482639
C	1.7825775083	0.4927576981	-1.0374149981
C	1.2579754689	1.2856934365	0.156193156
O	0.6047297014	0.4307803024	1.0860349142
C	2.355978383	1.9902949045	0.9285076158
H	-8.7312256469	-2.1390879851	0.12628453
H	-6.5535086243	1.2120306504	-1.4797360252
H	-5.9891618812	-3.0457256565	2.1311696113
H	-9.5247554802	-0.6280030581	-1.4031195358
H	-4.0693030649	1.3066144006	-1.008507335
H	-4.2343198082	1.5112153226	0.7358116707
H	-2.9599487632	-0.8737268167	-0.6688281168
H	-1.558602537	1.1920134604	-0.5800917061
H	-1.7817577611	1.2684523006	1.1673979714
H	-0.7502619221	-0.9959289673	1.393674316
H	-0.9009192106	-1.6939698013	-1.0606356997
H	0.6512483354	-1.9232128354	-0.2706989998
H	-0.0559962172	0.3801040068	-2.1629672115
H	1.0472056653	-0.9442652849	-2.4885895677
H	2.2485249604	1.1746338351	-1.7580471815
H	2.5640618205	-0.191196874	-0.6820867296
H	0.5446634046	2.0413758419	-0.2140159261
H	3.0727838039	1.2599464541	1.3148601035
H	1.9355420278	2.5367681332	1.7764064304
H	2.888761823	2.6969224288	0.2854705861

calc_**1b** conf_11

C	-7.439084488	-1.3326947211	-0.4890109461
C	-6.9152216088	-0.2846415637	-1.2360741194
C	-5.6651722192	0.2747527047	-0.931321992
C	-4.9376208202	-0.2359279199	0.1246685752
C	-5.4368096009	-1.3114592137	0.8842956499
C	-6.7129934046	-1.8466145434	0.5864646073
O	-7.2521340528	-2.8326841199	1.3006366417
O	-7.579634367	0.250641593	-2.2846139103
C	-3.6115117339	0.3316257862	0.5409670703
C	-2.7353665774	-0.7653575221	1.1155009742
O	-3.4216566866	-1.4344669928	2.1997695683
C	-4.6811382194	-1.8530701537	2.0079667799
O	-5.1444344494	-2.6684032199	2.7998485645
C	-1.4173315065	-0.2965851949	1.7158760052
C	-0.307973275	0.0745162321	0.7362462036
C	0.1601832947	-1.0645272427	-0.1675317325
C	1.2515948425	-0.5651656363	-1.1122037814
C	0.751885683	0.6608347148	-1.8741801588
C	0.2139857283	1.7356162151	-0.9254830609
O	-0.7545880053	1.181891568	-0.0342009985
C	1.3050464616	2.4990803452	-0.1798428013
H	-8.4130397766	-1.7584301062	-0.7093157621
H	-5.2961721209	1.0998025268	-1.5294531211
H	-6.6227774129	-3.012649257	2.0463396545
H	-8.4203349277	-0.2122809517	-2.396352683
H	-3.0906586327	0.8083918955	-0.2908367453

H	-3.7610173677	1.103360484	1.3081250191
H	-2.5569975316	-1.5246632955	0.3444006838
H	-1.6125641099	0.5611576893	2.3684682886
H	-1.0442790705	-1.1042804901	2.3547204714
H	0.5516537652	0.3854678427	1.3515341188
H	-0.6839484255	-1.4376650545	-0.7596525122
H	0.5217618423	-1.8972307047	0.4462642487
H	1.5438733522	-1.3532621027	-1.8138068084
H	2.1509602901	-0.315054608	-0.5354207362
H	-0.0607480821	0.3555281779	-2.5441929599
H	1.5435009162	1.0850471165	-2.5017156978
H	-0.3621710733	2.4621048598	-1.5075911984
H	0.852861801	3.1937014585	0.5329791156
H	1.9063383618	3.0772903192	-0.8879417067
H	1.9857332075	1.8423030853	0.3686711916

calc_**1b** conf_12

C	-7.5310598127	-0.5351012456	0.8408322238
C	-7.5241152324	-0.2835357338	-0.5253115062
C	-6.3797551043	-0.5176091787	-1.304217973
C	-5.2382006785	-0.9961668657	-0.6953445096
C	-5.2119336564	-1.2395149723	0.6912634198
C	-6.3802374198	-1.0258028545	1.4609425566
O	-6.423416867	-1.2755194586	2.7676185585
O	-8.6107129506	0.1898184873	-1.1741568546
C	-3.988688943	-1.3344265967	-1.4562494084
C	-2.7533147194	-1.0725734593	-0.6061374844
O	-2.8629317133	-1.7903501504	0.6456689698
C	-4.0030809567	-1.7256519789	1.3475045823
O	-3.9784939532	-2.0915533169	2.5193664032
C	-2.4788308144	0.4103718491	-0.3617785653
C	-1.0576375741	0.7415473477	0.0871511091
C	-0.6530224835	0.2038208346	1.4573068958
C	0.8097444241	0.5425102613	1.7357560782
C	1.681536143	0.0251872422	0.5931578527
C	1.1860801596	0.5242200743	-0.7663040877
O	-0.2025664439	0.2439481538	-0.9342208095
C	1.5040602975	1.9928396756	-1.034921623
H	-8.4170849375	-0.3709947736	1.4460647857
H	-6.4188305684	-0.3231610344	-2.369770679
H	-5.5407764633	-1.661758305	3.006466969
H	-9.3316394951	0.3083143534	-0.5417730774
H	-4.0106084975	-2.3981134018	-1.726364413
H	-3.9255923928	-0.7693149483	-2.3901844348
H	-1.8747983643	-1.5122975289	-1.0796193804
H	-2.6474132056	0.9352605401	-1.308709907
H	-3.1975073893	0.8158691444	0.3582417612
H	-0.9845741961	1.8412643339	0.1196410365
H	-0.7955027909	-0.8807181794	1.4814336481
H	-1.3090030477	0.628784697	2.225607117
H	1.1308491551	0.1008242454	2.6847514414
H	0.9279827485	1.6284994906	1.8442140958
H	1.6465841569	-1.0706052678	0.5900819511
H	2.7295199738	0.314115625	0.7315572214
H	1.6629882086	-0.0689935644	-1.5536529378
H	1.1236220535	2.6584367346	-0.2551961248
H	1.0661652488	2.3000859504	-1.9884985865
H	2.5871981024	2.1380437745	-1.0922163146

calc_**1b** conf_13

C	-7.3433489268	-0.2017467764	0.3802674666
C	-7.2896518442	-0.6203972949	-0.9429717088
C	-6.1778181075	-1.3169538628	-1.4426000899
C	-5.1159774582	-1.5798351353	-0.6024427861
C	-5.1359067019	-1.1493807273	0.7379332253
C	-6.2732455442	-0.4693162282	1.2361824011
O	-6.3624508529	-0.0695599945	2.5023087942
O	-8.2974785028	-0.3876765802	-1.8118112246
C	-3.9180478429	-2.3739300898	-1.0375353283
C	-2.6640730984	-1.9143547259	-0.3058702011
O	-2.8816214556	-1.9242974335	1.1226447181
C	-4.0087681313	-1.3993258826	1.6283455193
O	-4.0392485124	-1.1636496822	2.8323031122
C	-2.1605153179	-0.5501360516	-0.7582591677
C	-0.9686192439	-0.0109154528	0.0255456039
C	0.2613662208	-0.9138433491	-0.0028617454
C	1.4483964338	-0.2135002279	0.657455934
C	1.6637776503	1.16046555	0.0266410847
C	0.3736646465	1.9848754732	0.0247887742
O	-0.6871532301	1.2461214625	-0.5738449079
C	-0.0078548664	2.5343695103	1.3968007875
H	-8.2054132854	0.3256501821	0.7764654398
H	-6.1789536833	-1.6376909999	-2.4778610291
H	-5.5383947234	-0.3873499664	2.9544600653
H	-9.0010073321	0.0968094897	-1.3600842422
H	-4.0903836596	-3.4342358019	-0.8128109238
H	-3.7642205025	-2.2968115243	-2.1177473445
H	-1.8766365974	-2.6588967701	-0.4413335054
H	-1.872941459	-0.6249020734	-1.8139387582
H	-2.9675008185	0.1881960718	-0.7039532486
H	-1.2712042712	0.1328653739	1.0728959172
H	0.5032863798	-1.1486107126	-1.0484238519
H	0.0461882249	-1.857252521	0.5105336405
H	2.3539669619	-0.8217277089	0.5606942536
H	1.2578460111	-0.1129397832	1.7332064984
H	1.9864382126	1.0283248907	-1.0130684133
H	2.4575127041	1.711333846	0.5436940586
H	0.5029571638	2.8369097952	-0.6509498638
H	-0.9686519667	3.0523482433	1.3352605587
H	0.7468039127	3.250260625	1.7369935409
H	-0.0911165859	1.7557068431	2.1599469467

calc_**1b** conf_14

C	-7.206854015	-0.4289626081	-0.1455324212
C	-6.4776434722	-0.2733997271	-1.3180318836
C	-5.13650262	-0.6767859473	-1.401563571
C	-4.5297284834	-1.2305103049	-0.2927845122
C	-5.2394954231	-1.3789960639	0.9136255683
C	-6.599356836	-0.9916608476	0.978299206
O	-7.3283528529	-1.1441592339	2.0820895612
O	-7.0197933282	0.2650697507	-2.4334762208
C	-3.121690589	-1.7490186176	-0.3183075676
C	-2.4600445416	-1.5874881011	1.041972387
O	-3.2805839723	-2.1754397967	2.0791579088
C	-4.6007787693	-1.9420173552	2.097632444

O	-5.2176334786	-2.2259537167	3.1205335422
C	-2.1367955724	-0.1484438142	1.4469519518
C	-0.9554137993	0.518804428	0.745332747
C	0.3866945206	-0.1901897089	0.9174618852
C	1.4750925001	0.5678511718	0.1584113017
C	1.0560745281	0.7533817193	-1.2990971051
C	-0.3345059196	1.3832064025	-1.4132658815
O	-1.2810201991	0.6552776523	-0.6308662095
C	-0.3641332243	2.8765910426	-1.100732123
H	-8.2490721639	-0.1326086029	-0.0789213182
H	-4.6028346519	-0.5473004778	-2.3356808016
H	-6.7420702758	-1.6006489588	2.7400956655
H	-7.9398172938	0.5007494727	-2.2558062502
H	-3.1387803004	-2.815741068	-0.5774798086
H	-2.522028376	-1.2272954354	-1.0654829304
H	-1.5526776478	-2.1929482232	1.0800564451
H	-3.0226547949	0.47811765	1.2981724528
H	-1.9242128536	-0.1559806368	2.5214992111
H	-0.8606760214	1.5203938157	1.1947274476
H	0.3193656918	-1.211561077	0.5237673901
H	0.6303702846	-0.2666166158	1.9832361057
H	2.4274868383	0.030310477	0.2122496995
H	1.6430384296	1.5423453074	0.6343085553
H	1.0302821624	-0.2278399118	-1.7880809711
H	1.7847052863	1.3643729835	-1.8433156398
H	-0.6971763465	1.2513343864	-2.4378169228
H	-1.3946001427	3.2405996125	-1.1265507561
H	0.2128569871	3.4262398359	-1.8506054857
H	0.0579607361	3.1169211424	-0.121183096

calc_**1b** conf_15

C	-6.7006951234	-0.4376701581	1.4258431391
C	-7.1225576766	0.1686940314	0.2496456506
C	-6.3710716772	0.0690698247	-0.9313620346
C	-5.1846154783	-0.6361536166	-0.9165830004
C	-4.724585437	-1.2429765007	0.2669794078
C	-5.5050924278	-1.1587491048	1.4437854584
O	-5.1383638696	-1.747217961	2.5800393383
O	-8.2721312077	0.876737216	0.1782540398
C	-4.3459004429	-0.8332678756	-2.1484472931
C	-2.8725989742	-0.9153578755	-1.7740526262
O	-2.6689032746	-1.9385768878	-0.7800983711
C	-3.4649631764	-1.9790785337	0.2968226865
O	-3.1152908565	-2.6710987443	1.2477454647
C	-2.2935571619	0.4316897369	-1.3352313138
C	-0.9869657746	0.3317477179	-0.5554818813
C	-0.1240214439	1.5862970913	-0.6569652745
C	1.0904444843	1.4605119834	0.2622947085
C	0.6450070844	1.1152506892	1.6832619072
C	-0.290566731	-0.0967508599	1.7056140835
O	-1.3668772358	0.1057355457	0.791900097
C	0.407509686	-1.4356267878	1.4876682657
H	-7.2839780832	-0.3764763552	2.3393264884
H	-6.7399308605	0.5458822886	-1.8321665987
H	-4.3050323892	-2.2465091994	2.3777840772
H	-8.6973894984	0.8777201145	1.0458417731
H	-4.6429686276	-1.7650596911	-2.6462110588
H	-4.5038530583	-0.0218119695	-2.8653899814

H	-2.2942679558	-1.2894532348	-2.6226278901
H	-2.1444076147	1.0280232494	-2.2432555455
H	-3.0079304453	0.9735027499	-0.7066153673
H	-0.4054661249	-0.5258238215	-0.9296393176
H	-0.7295645202	2.4529945605	-0.3614449498
H	0.187849468	1.7421719726	-1.6965432058
H	1.6704402877	2.3895975817	0.2630379757
H	1.7582702953	0.6799780227	-0.1241382591
H	0.1050402367	1.9719786682	2.1038494617
H	1.5103809153	0.9303269228	2.3296402049
H	-0.7911558825	-0.138237152	2.6780553597
H	-0.3416733077	-2.2275746326	1.4135680165
H	1.0663342191	-1.6537842773	2.3341262661
H	1.0210996612	-1.4546547281	0.5821700988

calc_**1b** conf_16

C	-7.8382842972	-1.1701313421	0.6091087475
C	-7.7515732828	-0.2478397339	-0.4262946672
C	-6.5104688699	0.2331645344	-0.8723027363
C	-5.3559454201	-0.2326554387	-0.2785015773
C	-5.4141541361	-1.1840217567	0.7586583554
C	-6.6737814385	-1.6361008514	1.2214178946
O	-6.7908051228	-2.4986958168	2.2278923904
O	-8.8470077968	0.2366183357	-1.0502572843
C	-3.9904359462	0.2606024733	-0.6652225051
C	-2.9805965248	-0.8619134088	-0.496240347
O	-3.0126837335	-1.3589943969	0.8599693045
C	-4.2012332839	-1.6810484316	1.3972224028
O	-4.2066416263	-2.3719574284	2.4105383693
C	-1.5359152815	-0.5251395291	-0.8372435455
C	-0.9333438832	0.7536497654	-0.2347413419
C	-0.6902013697	0.7242653327	1.2755421271
C	0.5020639234	-0.158330258	1.6368568945
C	1.7209195436	0.2632232215	0.8199132594
C	1.392433719	0.2837711481	-0.6696922771
O	0.2573532477	1.1033014696	-0.9316756244
C	2.5180465854	0.8413139245	-1.5185248658
H	-8.7954490894	-1.5331247892	0.9701307484
H	-6.4855670597	0.9630769756	-1.6730743193
H	-5.8728720707	-2.6612007513	2.5663540371
H	-9.636977603	-0.162504388	-0.6626199078
H	-3.9784077967	0.6165420522	-1.6997609414
H	-3.717729957	1.1101628103	-0.027096243
H	-3.2889577457	-1.6973788943	-1.1384678849
H	-0.9339711622	-1.4019805833	-0.5801950944
H	-1.476990456	-0.414886054	-1.9261956588
H	-1.6086593056	1.5846546248	-0.4645971834
H	-1.5912816455	0.3932284692	1.7979121869
H	-0.4798809102	1.75194989	1.595902827
H	0.2651962187	-1.2120807661	1.4438197879
H	0.7113587271	-0.0838036878	2.7089620477
H	2.5682133825	-0.4087439108	0.9987250717
H	2.0353476062	1.2718032826	1.1186605075
H	1.1823218649	-0.7484443413	-0.9947209557
H	2.7402743067	1.870504729	-1.2216356042
H	2.2359642821	0.8438116495	-2.5745047629
H	3.4243234078	0.2403318702	-1.4000216322

calc_**1b** conf_17

C	-7.8232385658	-0.8480250894	0.4809358693
C	-7.5623525504	-0.3159439241	-0.7756952117
C	-6.247765288	-0.153516783	-1.2400302105
C	-5.1969995726	-0.5446231915	-0.436126826
C	-5.433931573	-1.1060101763	0.8335167533
C	-6.7636579334	-1.2343311019	1.3031985871
O	-7.0445671425	-1.7150112399	2.5119038508
O	-8.5505392629	0.0765838852	-1.6087872162
C	-3.7602337155	-0.3659964441	-0.8382648204
C	-2.9205949429	-1.4834296299	-0.2417221651
O	-3.0872018561	-1.5204807491	1.192154765
C	-4.3316801255	-1.5163328606	1.6958332962
O	-4.480304547	-1.84656385	2.8677413592
C	-1.4319378369	-1.4315501089	-0.5432899126
C	-0.6698365109	-0.148331791	-0.2189496504
C	-0.4745476437	0.1225544329	1.2696470613
C	0.4446794265	1.3250887151	1.473124572
C	1.749678577	1.1182434459	0.7084091909
C	1.4901632836	0.7747784713	-0.7604867504
O	0.5829607263	-0.3185848342	-0.8706697694
C	1.0504747836	1.9706103561	-1.601723563
H	-8.8367427052	-0.9605656448	0.8528895747
H	-6.0854008659	0.2780337303	-2.2209289905
H	-6.173860268	-1.8712907283	2.9607614057
H	-9.4048981551	-0.0830515733	-1.1865260877
H	-3.6536776385	-0.3659046458	-1.9271081058
H	-3.4001072646	0.605611788	-0.4781837645
H	-3.3034083827	-2.4389411403	-0.6236651489
H	-0.946560323	-2.2686585382	-0.03046712
H	-1.311275061	-1.6125051519	-1.6172803116
H	-1.1951140853	0.7105535289	-0.6683119065
H	-0.0284440689	-0.769198493	1.7271993319
H	-1.441967693	0.2767448911	1.7546568958
H	0.6491555437	1.4725697076	2.5386778911
H	-0.059085875	2.2371481156	1.1275311587
H	2.2989814389	0.285375671	1.163053611
H	2.3927083135	2.0032007731	0.7741110264
H	2.4140649669	0.3824658892	-1.198284147
H	0.8149519729	1.6435534319	-2.6181400787
H	1.857161801	2.7080421551	-1.6568635558
H	0.1709506197	2.4766887011	-1.1938408876

calc_**1b** conf_18

C	-7.2759784011	-0.1403709592	0.0837977407
C	-6.9882963882	-0.5038105385	-1.2253820592
C	-5.8170393119	-1.2088484742	-1.5454700744
C	-4.9328525979	-1.5352129229	-0.5384503013
C	-5.1902789319	-1.1616205549	0.7942439239
C	-6.3869061906	-0.4735457122	1.1077675063
O	-6.7008180578	-0.1263066048	2.3532355008
O	-7.8145915599	-0.2070538598	-2.2521523075
C	-3.689384964	-2.3408917111	-0.781201196
C	-2.5821579092	-1.933198551	0.1819956132
O	-3.0534366452	-2.0016314381	1.545015818
C	-4.2495902015	-1.4791373164	1.8626630838
O	-4.4996891397	-1.3040462344	3.0503446645

C	-1.9939871388	-0.5553864783	-0.1066561735
C	-0.8663390314	-0.1435131209	0.8516882576
C	0.4665476167	-0.8572548862	0.6051829651
C	1.1754822403	-0.3273646672	-0.640359336
C	1.2768296274	1.1942656293	-0.5701435522
C	-0.0969645179	1.8097983093	-0.3235191452
O	-0.688452911	1.2664848221	0.850017388
C	-0.0477758302	3.3109323959	-0.1191846189
H	-8.1857855046	0.3930724056	0.3404863832
H	-5.6345838824	-1.4869761325	-2.5770058819
H	-5.9817169549	-0.4847990336	2.9348869432
H	-8.5808417444	0.2751872955	-1.9144053746
H	-3.915466665	-3.4041483591	-0.6295017839
H	-3.3427246407	-2.2322527324	-1.8128647689
H	-1.7904796631	-2.6861005178	0.1553347612
H	-1.6499405384	-0.5574596293	-1.1473361974
H	-2.7835523301	0.2004733248	-0.0354539916
H	-1.2046714968	-0.3648001631	1.8675973945
H	0.3143461428	-1.9408316361	0.5409700727
H	1.104866556	-0.6831856678	1.4789370186
H	0.6250476142	-0.6153773309	-1.5458435353
H	2.1691271991	-0.7791841897	-0.7294883617
H	1.7054420354	1.6022153999	-1.4927688969
H	1.9394583636	1.4850229762	0.2551554572
H	-0.7383316227	1.5911968989	-1.1940930986
H	-1.0511996573	3.7038541837	0.0623644253
H	0.3689613107	3.8070974999	-1.0005976965
H	0.5757257227	3.5537082814	0.7461934338

calc_**1b** conf_19

C	-7.2482792327	0.2393562729	0.0104781697
C	-7.1694203342	-0.276497516	-1.2768325133
C	-6.0864254508	-1.075499318	-1.6773323984
C	-5.0774012695	-1.3403708548	-0.7751251797
C	-5.1221815617	-0.8131381001	0.5293689645
C	-6.2325172916	-0.0317367661	0.9295557278
O	-6.3447903347	0.4615169536	2.160395718
O	-8.1246899935	-0.0443353064	-2.2034729158
C	-3.9105630944	-2.2296756589	-1.0932706553
C	-2.6632121518	-1.7552669726	-0.3608976212
O	-2.9301654102	-1.677438503	1.057191505
C	-4.0447848255	-1.0654054822	1.48020182
O	-4.1090213139	-0.7437153357	2.6640554543
C	-2.1046790053	-0.4224405636	-0.8714332602
C	-0.5747107362	-0.339018804	-0.7685132473
C	-0.0211865584	1.0323705863	-1.1660832957
C	-0.222737892	2.0654103012	-0.0588457705
C	0.3188273205	1.5190479059	1.2596660749
C	-0.3073997816	0.1635822468	1.5710555066
O	-0.0850979256	-0.7483154056	0.4952656622
C	0.2651064075	-0.4982420492	2.8069523437
H	-8.0900582949	0.8451474302	0.331017397
H	-6.068953385	-1.4719691132	-2.6859079064
H	-5.5616910888	0.1236949039	2.6683589784
H	-8.8140142619	0.5114684587	-1.8166421208
H	-4.1410796543	-3.2541490887	-0.7734407274
H	-3.719879708	-2.2665566637	-2.1695840781
H	-1.8851612588	-2.5187239544	-0.4224121892

H	-2.3717640174	-0.2986441633	-1.9276991478
H	-2.5917310555	0.4015673838	-0.340917426
H	-0.1693523092	-1.0910176677	-1.4571738886
H	-0.4798102325	1.3630599265	-2.1051608564
H	1.0529081582	0.9205017113	-1.3546279356
H	-1.2888754328	2.3013767804	0.0536711363
H	0.2739135223	3.0043303944	-0.3256483756
H	0.1171356199	2.2134382917	2.0830472348
H	1.4078717707	1.3968480331	1.1937327008
H	-1.3890327699	0.2967786608	1.7164661971
H	0.1151843148	0.1355378331	3.6855629954
H	1.3372902824	-0.677565527	2.6817571813
H	-0.2315697639	-1.4553112605	2.9812207417

calc_**1b** conf_20

C	-7.8777869465	-1.1221354418	0.1008990155
C	-7.5311375631	-0.0284378605	-0.6826386062
C	-6.2191242213	0.4697317504	-0.6989963658
C	-5.2546146553	-0.1499094676	0.0688892534
C	-5.5745553661	-1.2721050234	0.8558002123
C	-6.908416862	-1.7455824658	0.888299777
O	-7.2764192187	-2.7741222835	1.6482631761
O	-8.4311408297	0.6124293482	-1.459964248
C	-3.8363734219	0.3427730277	0.1350071313
C	-2.8896474883	-0.8234490816	0.3582744954
O	-3.281263287	-1.5630793319	1.5336891674
C	-4.5639273969	-1.9306882098	1.6750430147
O	-4.8278858511	-2.798028107	2.5009603328
C	-1.4320112666	-0.4528949342	0.5789117662
C	-0.7696867943	0.1870903852	-0.6588547194
C	-0.6602212671	1.7144468075	-0.5923538
C	0.4319762686	2.1579671536	0.3795625749
C	1.7411040584	1.443195353	0.0536370571
C	1.5227016728	-0.0651604912	-0.000621905
O	0.4987259299	-0.3884300481	-0.9342097234
C	2.754940662	-0.8321852583	-0.4368131121
H	-8.8940281189	-1.5024642951	0.1304110877
H	-5.9914320685	1.3322571751	-1.3146210821
H	-6.4751474086	-3.0372796759	2.1706136262
H	-9.2919222019	0.1827115781	-1.369791384
H	-3.571476567	0.8693508016	-0.7863191462
H	-3.7264671876	1.0579723983	0.9616482907
H	-2.9643889977	-1.5111278481	-0.495199497
H	-1.3646956706	0.1886850217	1.4644145394
H	-0.8999788814	-1.3796168136	0.8130978829
H	-1.3626908132	-0.0835977193	-1.5408615934
H	-1.6257349885	2.1535803812	-0.3177798731
H	-0.4140607311	2.0795838082	-1.596385539
H	0.1378013345	1.9265907233	1.411801549
H	0.5592186784	3.2445272198	0.3319399325
H	2.5117442883	1.6803666765	0.7958150435
H	2.1141959932	1.7750739381	-0.9237850536
H	1.2221316479	-0.4119238218	1.0027690946
H	3.5835082497	-0.656629463	0.2553284336
H	3.0626695823	-0.5149754195	-1.4375266042
H	2.5465177049	-1.9045104865	-0.4673542018

calc_**1b** conf_21

C	-7.7176395556	-1.3738322877	-0.2508455708
C	-7.92125013	-0.0692632515	0.1813260185
C	-6.8535454606	0.7269038351	0.6236536274
C	-5.5762961779	0.2045737905	0.6166209681
C	-5.3395128381	-1.1082066694	0.1659966277
C	-6.4290841544	-1.9105841541	-0.2505205671
O	-6.2689574355	-3.1709331075	-0.6468915861
O	-9.1479464249	0.497061868	0.202832899
C	-4.3840769307	0.9771454328	1.1057272695
C	-3.1415345931	0.553704752	0.3417590993
O	-2.9580844039	-0.8705986958	0.4373914366
C	-3.9941211498	-1.6728904631	0.1592735597
O	-3.7625312224	-2.8550953444	-0.0758057217
C	-1.8676674151	1.2207029892	0.8313287498
C	-0.5917446352	0.715802936	0.1577187765
C	0.4868984445	1.788097144	0.0251460887
C	1.7587991191	1.177385634	-0.5613903512
C	2.1805956042	-0.0482904463	0.2497271184
C	1.0220050813	-1.0336921014	0.4330374429
O	-0.1191233694	-0.3573472559	0.9510543296
C	0.6909403857	-1.8504573963	-0.8136684339
H	-8.5410338129	-2.0000635937	-0.5797369774
H	-7.05479021	1.7363105605	0.9634116162
H	-5.3074512008	-3.3829693217	-0.5212817031
H	-9.7974735209	-0.1402481654	-0.1221297455
H	-4.5424386983	2.0537370884	0.9914649383
H	-4.2267267405	0.7843717398	2.1753527411
H	-3.282819852	0.7834960115	-0.7238609156
H	-1.9807232903	2.2969706959	0.6553686697
H	-1.7573113058	1.0739704098	1.9108849649
H	-0.8439057182	0.3524152516	-0.8519871008
H	0.6926258558	2.2028989118	1.0206251742
H	0.1235879177	2.6091837342	-0.6043720835
H	2.5682884725	1.91488825	-0.5802010304
H	1.5750071686	0.8943285271	-1.605816787
H	2.5093817349	0.2801619874	1.2429418622
H	3.0315349526	-0.5540039208	-0.2205625754
H	1.2820361479	-1.7369817515	1.2306392905
H	0.543624968	-1.2311142305	-1.7032812015
H	-0.2236357083	-2.4242249521	-0.6429264487
H	1.5061001016	-2.5473144405	-1.0320044689

calc_**1b** conf_22

C	-7.2796136087	-0.1422958164	0.0941220131
C	-6.9988772868	-0.4994604887	-1.2171398862
C	-5.8261175512	-1.1998185497	-1.5465276542
C	-4.9316988255	-1.5306509837	-0.5469704221
C	-5.1843915903	-1.1650554071	0.7858796444
C	-6.3838899131	-0.4791635514	1.1080641698
O	-6.6886233119	-0.1398552921	2.3579558784
O	-7.9027588351	-0.1487895478	-2.1586770302
C	-3.6871723789	-2.3302058082	-0.8051754962
C	-2.5763659103	-1.9290172181	0.1568820637
O	-3.042737709	-2.0113195051	1.5196486677
C	-4.2377620742	-1.4900318386	1.8474848221
O	-4.4803866482	-1.3242588473	3.037228647
C	-1.9916406572	-0.547894229	-0.1228704962

C	-0.8797470423	-0.1278804772	0.850345774
C	0.4553777824	-0.8492468139	0.6411846014
C	1.1917473027	-0.3400614683	-0.5971703187
C	1.296042699	1.1822897316	-0.5476044626
C	-0.0805364941	1.8051732363	-0.3382233924
O	-0.6971640689	1.281237073	0.8309405469
C	-0.0311499609	3.309107217	-0.1558507074
H	-8.1927961533	0.3867904965	0.3349497651
H	-5.6280326987	-1.4830585428	-2.5772293497
H	-5.9678083659	-0.5001919879	2.9341027191
H	-7.6010066681	-0.4611964678	-3.0213614396
H	-3.908543909	-3.3958996561	-0.6638096629
H	-3.3447996803	-2.2093138834	-1.8372176964
H	-1.7835234417	-2.6802087929	0.1194370359
H	-1.6331369478	-0.5483556752	-1.1587638907
H	-2.7857175457	0.2041960886	-0.0622621561
H	-1.2382356068	-0.3332961892	1.8625602731
H	0.3015309368	-1.9332763738	0.591206633
H	1.0760466058	-0.6632038121	1.525125946
H	0.6598326512	-0.6406368579	-1.5096976832
H	2.1857936726	-0.7956503024	-0.6583150595
H	1.7449304626	1.575385775	-1.4670478198
H	1.9423557065	1.4833269605	0.2868749664
H	-0.7048049648	1.5752270946	-1.2183534781
H	0.4045904397	3.7906291201	-1.0362338021
H	0.5756359493	3.5635683922	0.717978726
H	-1.0368443601	3.7073631987	-0.0004709885

calc_**1b** conf_23

C	-7.7815940579	-1.0851508318	-0.202011421
C	-7.4216965693	0.1845503679	-0.6363166121
C	-6.157403109	0.7223111848	-0.348709766
C	-5.2522946878	-0.033645858	0.3675216161
C	-5.5840732026	-1.3310198453	0.8011544004
C	-6.8736135043	-1.8492858301	0.5332071224
O	-7.2563609368	-3.0506522965	0.959246899
O	-8.2634948618	0.9659814143	-1.3478420173
C	-3.8929605704	0.4759178506	0.7529304141
C	-2.8975545288	-0.6715568504	0.788359419
O	-3.3745719215	-1.6941177713	1.6923233544
C	-4.6358682165	-2.1340536856	1.5650725178
O	-4.9364407695	-3.1906466733	2.1108776134
C	-1.4992114814	-0.2989193719	1.2673494493
C	-0.4913055983	-0.0299833235	0.1541516023
C	-0.8249013264	1.1566124036	-0.7470816004
C	0.325624976	1.4372576463	-1.713111767
C	1.632761051	1.588931804	-0.9384605427
C	1.8726713623	0.3971491337	-0.0084901592
O	0.7387753317	0.1807939821	0.8270244262
C	2.3036382575	-0.8739529465	-0.7348472294
H	-8.7636063039	-1.5001215831	-0.4062645005
H	-5.9195056264	1.7221371555	-0.693058825
H	-6.509092451	-3.4039089841	1.5079377563
H	-9.0965910491	0.4953641649	-1.4827304465
H	-3.5537011617	1.2514482264	0.0616872152
H	-3.9344271997	0.9320608776	1.7512214752
H	-2.8449704006	-1.1341423277	-0.2071989361
H	-1.5373629818	0.5706236592	1.9332232392

H	-1.1138016903	-1.1335039636	1.8590879436
H	-0.4237034013	-0.9350236343	-0.4732920436
H	-1.0026259109	2.0367928987	-0.1150710911
H	-1.7466810864	0.9584579255	-1.3064872767
H	0.1225341468	2.3411622762	-2.2968733241
H	0.4048126261	0.6149238467	-2.4353350547
H	1.581288648	2.4968824318	-0.32597971
H	2.4824586919	1.7072657683	-1.6200527538
H	2.6637854541	0.6594963787	0.7014108786
H	1.6039016131	-1.1741019709	-1.5197425814
H	2.3887542876	-1.6983451991	-0.0219676942
H	3.2804081592	-0.7239884502	-1.2048619898

calc_**1b** conf_24

C	-7.7691823038	-0.9976330888	-0.1438127667
C	-7.2934963115	0.154991461	-0.7564894723
C	-5.9731718057	0.5903390234	-0.5645363018
C	-5.1302120918	-0.1506037579	0.2384073613
C	-5.5803738144	-1.3335164029	0.853257422
C	-6.9237945962	-1.7449702265	0.6778350888
O	-7.4156096031	-2.8291927817	1.2722460225
O	-8.0708210311	0.9151922218	-1.5586205153
C	-3.7162798025	0.2689456799	0.5270306757
C	-2.8392602743	-0.953069219	0.7361035539
O	-3.3985366932	-1.7991191533	1.7626332279
C	-4.6995180276	-2.1204404661	1.70844739
O	-5.0915066872	-3.0577971185	2.395501902
C	-1.4135503586	-0.6745999937	1.1823525505
C	-0.5639142117	0.0822916214	0.1576393106
C	-0.4575605049	1.5823994967	0.4268376817
C	0.5031444511	2.2391723685	-0.5612255457
C	1.8473513419	1.5148149708	-0.5271682221
C	1.6778982324	0.0055554004	-0.7178314503
O	0.7232738546	-0.5165355006	0.203581566
C	1.3682464133	-0.4001208211	-2.1566136222
H	-8.7939488298	-1.330911931	-0.2745460183
H	-5.6439208989	1.502124612	-1.0494076625
H	-6.6925507047	-3.1820985392	1.8530042053
H	-8.9502199488	0.518964043	-1.616730104
H	-3.3225546425	0.8720439145	-0.2959916548
H	-3.6877038012	0.8927893133	1.4307358231
H	-2.8229797137	-1.5404530575	-0.1921488392
H	-1.4332847284	-0.1430382322	2.140400741
H	-0.930625886	-1.6387070068	1.3619582805
H	-0.9965574749	-0.0654704079	-0.8479536232
H	-0.0833303627	1.7164084803	1.4498653466
H	-1.4470120614	2.0503867641	0.3820484795
H	0.6371627185	3.2988878256	-0.3198845318
H	0.075163715	2.2047319922	-1.5713696586
H	2.3175083801	1.6870195484	0.4481718703
H	2.528901726	1.9111101294	-1.288087286
H	2.6085776615	-0.4913303532	-0.4253501477
H	2.2155976563	-0.1573097629	-2.8050731265
H	0.488846104	0.1077913284	-2.5624986079
H	1.1938049158	-1.4780423743	-2.2097193424

calc_**1b** conf_25

C	-7.0248812447	0.0924539983	0.1328744829
C	-6.5544619945	-0.0420133986	-1.1670274884
C	-5.4078843811	-0.8009804393	-1.4520999699
C	-4.7325113858	-1.4131050131	-0.4160460382
C	-5.1755297017	-1.2748879524	0.9126636858
C	-6.3489892974	-0.5315683305	1.183782293
O	-6.8364689153	-0.4007400921	2.4148173589
O	-7.1720068598	0.5380407558	-2.2191841711
C	-3.5358036048	-2.2929720261	-0.6308652882
C	-2.5735946269	-2.18693452	0.5439291747
O	-3.2684211105	-2.4788622007	1.7760649798
C	-4.4556126964	-1.9026335516	2.0166350439
O	-4.8910514918	-1.9393693596	3.1629186822
C	-1.8423835598	-0.8546414944	0.694693066
C	-0.8732443996	-0.5549416118	-0.4678249559
C	-1.4199066472	0.4456620094	-1.4920117647
C	-1.4035535473	1.8720341551	-0.946748101
C	-0.0141389773	2.2105949264	-0.4139601027
C	0.439073467	1.1549974628	0.5885487776
O	0.4023289174	-0.1417284793	0.0018171888
C	1.8594561914	1.3611123197	1.0755872556
H	-7.9193975391	0.6644120408	0.3586431132
H	-5.0846474053	-0.8999103878	-2.4820076091
H	-6.2579583308	-0.9512626522	3.0041725754
H	-7.9448372785	1.025851944	-1.9052454818
H	-3.8668194573	-3.336070703	-0.7140944013
H	-3.0233920794	-2.0507640844	-1.5658452791
H	-1.831307399	-2.986606184	0.476330852
H	-2.574527399	-0.0517130623	0.8312833791
H	-1.2597415899	-0.9222121911	1.6180935819
H	-0.6544393461	-1.499131425	-0.9822703479
H	-2.4344347305	0.1647782612	-1.7951229258
H	-0.7868745329	0.3972654057	-2.3858965102
H	-2.1405206895	1.9770908661	-0.1399505174
H	-1.703210576	2.5761996457	-1.7298814749
H	-0.0055690902	3.198606942	0.0599708205
H	0.7072493374	2.2371116611	-1.2408074498
H	-0.2440996766	1.1810915235	1.4545673898
H	2.138455939	0.5767381943	1.7835774155
H	1.9603393235	2.3310562533	1.5708519333
H	2.5563183862	1.3249507938	0.2330668276

calc_**1b** conf_26

C	-7.7937331577	-1.1192862544	-0.0813807873
C	-7.3861538447	-0.0334146311	-0.8461220124
C	-6.0770237512	0.4665937447	-0.7629221914
C	-5.1772174836	-0.1436059408	0.0862984859
C	-5.5582811947	-1.2583042553	0.8567741973
C	-6.8899445916	-1.7332096753	0.7876140952
O	-7.3171040555	-2.7538313722	1.5270852902
O	-8.2212193992	0.5986259247	-1.6994804903
C	-3.7701588417	0.3491155401	0.2690660352
C	-2.842538704	-0.8176474088	0.5635649775
O	-3.3257293734	-1.5337532252	1.7225535458
C	-4.6156551375	-1.9034651549	1.7638172955
O	-4.9442017143	-2.7607048728	2.576968169
C	-1.3970401963	-0.4322308882	0.8676822675
C	-0.4318482544	-0.6400860246	-0.3093209259

C	-0.6697890343	0.2540365136	-1.5282817942
C	-0.2348532351	1.6930023003	-1.2615173486
C	1.2102831441	1.7021976669	-0.7678027458
C	1.3783305949	0.7690724467	0.4282248116
O	0.9175719688	-0.5425177377	0.1206640474
C	2.8218777396	0.616996313	0.8647831547
H	-8.8090435495	-1.500273051	-0.1285136967
H	-5.8016341888	1.3240378217	-1.3660004313
H	-6.5608577654	-3.0090462584	2.1161184043
H	-9.087062092	0.1703838315	-1.6731562594
H	-3.4279283448	0.8943370523	-0.6146001615
H	-3.7281813904	1.0490421642	1.1145548365
H	-2.8760436116	-1.5250885777	-0.2770679504
H	-1.3628420734	0.5968058493	1.2409468337
H	-1.0459757876	-1.0751909303	1.6795064162
H	-0.5317901727	-1.6864219259	-0.620968928
H	-1.717095126	0.2022198483	-1.8464372848
H	-0.073628187	-0.1482453249	-2.3557482061
H	-0.8842221455	2.1556957378	-0.5064392352
H	-0.337414316	2.2960714048	-2.1697768615
H	1.5249111066	2.7146766157	-0.4906748533
H	1.8760435001	1.3616292681	-1.5713145851
H	0.7951071053	1.1741400945	1.2715226273
H	3.2415510487	1.5846378472	1.1546638277
H	3.4226026946	0.2056578411	0.048324642
H	2.8919318172	-0.0636523175	1.7167927887

calc_**1b** conf_27

C	-6.3517370232	0.3614967164	-0.3048366429
C	-5.8927031064	-0.1337591214	-1.5207216144
C	-4.8695173497	-1.0891183834	-1.5763590809
C	-4.3098361059	-1.5506576046	-0.4002419163
C	-4.7528664069	-1.0657312413	0.8415425823
C	-5.792752499	-0.1024322686	0.8862570698
O	-6.2661746774	0.3777876885	2.033825381
O	-6.4095205177	0.2751855971	-2.7012160124
C	-3.2973767055	-2.657453191	-0.4024323518
C	-2.4440343507	-2.7313700924	0.8631324842
O	-3.193488134	-2.4500970298	2.0636427257
C	-4.1976528046	-1.5667099379	2.0918562857
O	-4.6244199334	-1.2322082266	3.1943386102
C	-1.1435781147	-1.9299526181	0.8829260926
C	-1.1898299226	-0.4207131387	0.691208519
C	0.157236001	0.2027464548	1.0619861778
C	0.2095229362	1.6801008773	0.6829776549
C	-0.1828852166	1.8489093062	-0.7828740755
C	-1.5302388866	1.1851324293	-1.0740624453
O	-1.5040682578	-0.1859040199	-0.672485029
C	-2.7159658972	1.9463193738	-0.49035381
H	-7.149450065	1.0958617582	-0.2523258726
H	-4.5406243739	-1.4546200973	-2.5421167164
H	-5.7830919854	-0.1038670094	2.7561300813
H	-7.1070113079	0.9225132364	-2.533664111
H	-3.8416043988	-3.6043748032	-0.5101313986
H	-2.6384294404	-2.5653441712	-1.2687700152
H	-2.1503141906	-3.775140607	1.0032353692
H	-0.6669920192	-2.1381511717	1.8472456971
H	-0.4871779594	-2.3419918661	0.1065454369

H	-1.969947378	0.0137133259	1.3357981119
H	0.9474476361	-0.3400960447	0.5260700624
H	0.3386768777	0.0665154568	2.1340178715
H	1.2125354684	2.0831977722	0.8584840847
H	-0.4701668632	2.2518440238	1.3265417184
H	0.5797379439	1.3782887976	-1.4149682973
H	-0.2250290606	2.9079574768	-1.0606857387
H	-1.6698643611	1.1270032803	-2.1583564604
H	-2.7770441793	2.9395767967	-0.9464389706
H	-2.6403346088	2.0866464266	0.5912860394
H	-3.6464287618	1.4148958498	-0.6980074966

calc_**1b** conf_28

C	-7.2243521279	-0.0402002481	0.3786108263
C	-6.6803662567	0.2492077953	-0.8660806888
C	-5.4538380881	-0.3012609133	-1.2712633655
C	-4.773252356	-1.1345717703	-0.407188702
C	-5.2916325941	-1.4263591773	0.8682212826
C	-6.5432915049	-0.8896986112	1.2531833313
O	-7.1006713386	-1.1643112188	2.429642552
O	-7.2994156698	1.062778068	-1.7495917159
C	-3.4870302485	-1.8156472374	-0.7753265076
C	-2.5978843323	-1.9828430057	0.448774183
O	-3.3193258031	-2.6713748539	1.4929000269
C	-4.5681971531	-2.2890437164	1.7972883025
O	-5.0539559234	-2.6951631132	2.8480072519
C	-2.0051461492	-0.6988868003	1.0218607573
C	-1.0106672521	-0.0009169461	0.0874986971
C	-1.5756869249	1.2419225186	-0.5966179901
C	-0.4941670305	1.9386803415	-1.4185596295
C	0.7173426691	2.2241462815	-0.5329505108
C	1.1906364337	0.9655470088	0.1996526187
O	0.1092357339	0.3442618738	0.8899533037
C	1.9432195304	-0.0184593546	-0.6927913659
H	-8.1798075855	0.3688287819	0.6918740245
H	-5.0722892011	-0.0676179118	-2.2586050791
H	-6.4999968636	-1.8149002133	2.8781187427
H	-8.1277320182	1.3779137071	-1.3644669256
H	-3.7102939823	-2.8099578058	-1.1829212029
H	-2.9542384281	-1.2676126583	-1.557350048
H	-1.7814199339	-2.669655211	0.2108636174
H	-2.8085328538	-0.0125032648	1.3106787544
H	-1.4659879484	-0.9678351253	1.9339340165
H	-0.6888703559	-0.7136139698	-0.6931230204
H	-1.9418744397	1.9209522981	0.1838453838
H	-2.438371625	0.9778553672	-1.2185512352
H	-0.8774137281	2.8702674305	-1.8479463899
H	-0.2068975098	1.3029745596	-2.2662374744
H	0.4377193209	2.9724413502	0.2179615237
H	1.5430506022	2.6460991285	-1.1166237882
H	1.8669169238	1.2643478542	1.0071045904
H	2.1908399051	-0.9198640965	-0.1258344437
H	2.8762315947	0.4326701517	-1.0442697942
H	1.371414513	-0.3175972931	-1.5756739089

calc_**1b** conf_29

C	-7.3973210916	-0.2033074186	0.266852206

C	-6.9081200586	-0.0110929031	-1.0188180354
C	-5.653276033	-0.5096062079	-1.4033203241
C	-4.8901482549	-1.1939875624	-0.4797431283
C	-5.3529246189	-1.3834877772	0.8351675698
C	-6.6311417869	-0.9005532699	1.203138046
O	-7.1354299047	-1.0865703948	2.4204659455
O	-7.6091946583	0.6548523747	-1.9626627812
C	-3.56375861	-1.8100101601	-0.8224030649
C	-2.6350050959	-1.7880041524	0.3851095244
O	-3.2836056151	-2.4237735357	1.5102362543
C	-4.545485818	-2.0924773644	1.8211251014
O	-4.9695053154	-2.4175778067	2.9255390274
C	-2.1361900956	-0.3986859596	0.7932410866
C	-0.7673561413	-0.0227901887	0.2310089758
C	-0.698220651	0.0954397133	-1.2896471701
C	0.6635087198	0.6366151109	-1.7246785541
C	0.9703514938	1.935027049	-0.9824649629
C	0.8207238551	1.7629196168	0.5306354334
O	-0.4568446706	1.2164348039	0.8464984153
C	1.9577988139	0.9773083871	1.178209113
H	-8.3728499869	0.166575355	0.5665384464
H	-5.3126196325	-0.3511376841	-2.4199931595
H	-6.4758462002	-1.6396285846	2.9141428205
H	-8.4479530032	0.9509637515	-1.5851609577
H	-3.7185171899	-2.8516749896	-1.1317079177
H	-3.1003050264	-1.290495922	-1.6643560926
H	-1.7787861315	-2.4428750404	0.2009482893
H	-2.8606486866	0.376075904	0.5195703278
H	-2.0435061401	-0.3783445546	1.8827240388
H	-0.042659296	-0.79070894	0.5521850201
H	-1.492740604	0.7750958197	-1.6248944571
H	-0.8804243378	-0.8814554657	-1.7528004043
H	0.6801042142	0.8038595638	-2.8067566165
H	1.4379639884	-0.1123823988	-1.5164004852
H	0.2657344165	2.7081863348	-1.3107987347
H	1.9778440445	2.2946986858	-1.2193374355
H	0.7942133011	2.7539075866	0.9952288195
H	1.7537783048	0.8393303319	2.2432763053
H	2.8987450951	1.526845971	1.0771098003
H	2.1036184076	-0.0095080782	0.7299937146

calc_**1b** conf_30

C	-7.0411492462	-0.1145011113	-0.1664352005
C	-6.4167610027	-0.2802402471	-1.3959659338
C	-5.1895822971	-0.9539223584	-1.5052178443
C	-4.5919984091	-1.4509892044	-0.3649859361
C	-5.1920931771	-1.2765484134	0.8951427659
C	-6.4423240248	-0.6203599406	0.9893642785
O	-7.0748529421	-0.4638681565	2.1494305812
O	-6.9533335495	0.1879603847	-2.5441263034
C	-3.3116352298	-2.2352043559	-0.3978919871
C	-2.5004408019	-1.9873385512	0.8679215674
O	-3.3120900874	-2.2757950989	2.0295226062
C	-4.5570150396	-1.7834400113	2.1064274681
O	-5.1135921483	-1.7948068347	3.1995220576
C	-1.9022115368	-0.5805017257	0.9867793948
C	-0.4278605822	-0.4888194388	0.5606083598
C	-0.1372092649	-0.7628969115	-0.916179125

C	-0.6075544687	0.3881830136	-1.8014967134
C	0.0024665243	1.6931199771	-1.2954131241
C	-0.2897473512	1.8892729189	0.1901691532
O	0.1294957115	0.7583288749	0.9483058168
C	0.4313557048	3.0837659382	0.782253491
H	-7.9971886036	0.3916367315	-0.0766318694
H	-4.7423490296	-1.0783133075	-2.4847425939
H	-6.526429235	-0.9306599463	2.8321143089
H	-7.7917562567	0.6263264636	-2.3475572173
H	-3.5438557613	-3.305586403	-0.4670219355
H	-2.7224636107	-1.977691243	-1.2813022908
H	-1.7016543055	-2.7305345497	0.9438818698
H	-2.524027849	0.1362867984	0.4411577466
H	-1.9321705889	-0.2906844405	2.0410457777
H	0.1233464269	-1.2200078966	1.1639609555
H	-0.5741199143	-1.7183038161	-1.2275129316
H	0.9487468865	-0.8700575221	-1.022976869
H	-1.7031465435	0.4615909566	-1.7868315947
H	-0.3225764119	0.2077757633	-2.8434103488
H	-0.37913105	2.5510450622	-1.8603521766
H	1.0909458868	1.667655801	-1.4354212651
H	-1.3751359022	2.0330201536	0.3162857024
H	0.1336970934	4.0049832622	0.2730576527
H	1.5134692778	2.9610710536	0.6791871196
H	0.1979327092	3.181048331	1.8453345866

calc_**1b** conf_31

C	-6.6856202094	0.1594411994	0.1400087909
C	-6.1398422171	0.1087644402	-1.1370691676
C	-4.9896785344	-0.6448504202	-1.4082455299
C	-4.3801705419	-1.3388091057	-0.3833963854
C	-4.9042927702	-1.2952823229	0.9206601832
C	-6.0791160164	-0.5461148808	1.1791514831
O	-6.6323861313	-0.4884833771	2.3891165685
O	-6.6901069062	0.7725290537	-2.1783493352
C	-3.2024662986	-2.2379932201	-0.6253253213
C	-2.3162878624	-2.4294136759	0.6040992739
O	-3.0897068544	-2.6217054399	1.8082505861
C	-4.261742429	-2.0074603293	2.0162221223
O	-4.745497339	-2.0829243507	3.1425416016
C	-1.231459612	-1.3814503562	0.8622995195
C	-1.6493494037	0.093774038	0.8573422172
C	-0.6560655066	1.0002335364	1.5904264035
C	0.6188360634	1.2136681832	0.7794250582
C	0.2491535748	1.7211552424	-0.6115784192
C	-0.7522346351	0.7840019793	-1.2802968835
O	-1.8968029422	0.5739932416	-0.4615656909
C	-1.2706086967	1.3221278922	-2.6000778803
H	-7.5827369225	0.7316055338	0.3547064119
H	-4.5974200671	-0.661724154	-2.4180094054
H	-6.0873470426	-1.0782588156	2.972473497
H	-7.4688358421	1.2541965884	-1.8700704086
H	-3.5851028378	-3.2207809265	-0.9293011408
H	-2.5964327814	-1.8602432738	-1.4517606514
H	-1.7983411226	-3.3865485667	0.5000112636
H	-0.7958390629	-1.6284049286	1.8367279165
H	-0.4366345787	-1.5449344488	0.1259965324
H	-2.6192361451	0.1844176255	1.3545782775

H	-0.4377237336	0.585720481	2.5804320617
H	-1.1407505737	1.9708049333	1.7486555498
H	1.180876039	0.2740871551	0.6958634365
H	1.2787324387	1.9245440566	1.287637515
H	1.136988068	1.8176749419	-1.2468472389
H	-0.204847347	2.7174483132	-0.5317782676
H	-0.2478793363	-0.1799000642	-1.466004176
H	-0.4464481588	1.4825171132	-3.3011828781
H	-1.7866170046	2.2732497707	-2.441948184
H	-1.9799587202	0.6233273376	-3.0508193056

calc_**1b** conf_32

C	-6.3302112195	0.5584526836	0.1159147467
C	-6.0924954503	0.087783833	-1.1695262366
C	-5.2183109711	-0.9852053395	-1.4022134738
C	-4.5779466756	-1.575031194	-0.3306470663
C	-4.7874613407	-1.1078362848	0.9786075738
C	-5.6887254523	-0.0383334229	1.2029782774
O	-5.9452598228	0.4292406909	2.4216370302
O	-6.688806931	0.6285547057	-2.2542435349
C	-3.6939645866	-2.7775506222	-0.4868801618
C	-2.5965122032	-2.8536347083	0.5725499089
O	-3.11155782	-2.5889373679	1.8957694455
C	-4.0800774087	-1.692300191	2.1113036686
O	-4.3248242663	-1.3889035218	3.2763445781
C	-1.3309575638	-2.0471551988	0.2887121701
C	-1.4225108883	-0.5351464113	0.0750172384
C	-1.3784853849	0.2831633697	1.3632034504
C	-1.3059668472	1.7751462769	1.0452940268
C	-0.1332027482	2.0488821341	0.1065564427
C	-0.1759177635	1.1407336249	-1.1249740094
O	-0.2943722328	-0.2258471823	-0.7386689623
C	-1.2374825832	1.5421594458	-2.1465764958
H	-7.0140768508	1.3800627242	0.3045469401
H	-5.0698392474	-1.3340215135	-2.4175701668
H	-5.4314656508	-0.1405149064	3.0524007152
H	-7.2686205015	1.3484845363	-1.9726835254
H	-4.3201322076	-3.6751200599	-0.4046251646
H	-3.2384919026	-2.8031846845	-1.4810583981
H	-2.2758253471	-3.8958107651	0.6472499576
H	-0.6093256839	-2.255428138	1.0863673823
H	-0.9035453815	-2.4646872348	-0.6302957192
H	-2.3425380197	-0.2932391378	-0.476513592
H	-2.2415968224	0.066317514	1.9982133786
H	-0.4851194201	-0.0191265948	1.9248843066
H	-1.1969424225	2.3563538803	1.9668822319
H	-2.2490170565	2.098862316	0.5868365782
H	0.8030636865	1.8541384373	0.6430266614
H	-0.113015445	3.0989217294	-0.2065003872
H	0.7980208166	1.1800237319	-1.6242642683
H	-1.0054356494	2.5281143316	-2.5610388574
H	-2.2425647376	1.592680066	-1.7186995975
H	-1.2544819965	0.8209384482	-2.9683170923

calc_**1b** conf_33

C	-6.7937413613	0.3057345874	0.4043563784
C	-6.6245548314	0.0369544605	-0.9481632322

C	-5.6287255208	-0.8445633592	-1.3966242251
C	-4.7975086698	-1.44581634	-0.4733774227
C	-4.9351243588	-1.1778089907	0.9002079298
C	-5.9574302978	-0.302308718	1.3420957601
O	-6.1509998354	-0.0306646564	2.6298069861
O	-7.4062249973	0.5981302731	-1.8954933874
C	-3.7673266432	-2.4631531567	-0.8677770075
C	-2.5699484084	-2.5072160625	0.0788499925
O	-2.9770118036	-2.4795474305	1.4636689559
C	-4.0339598607	-1.7708046399	1.8791583824
O	-4.1898990697	-1.6455922431	3.0912154598
C	-1.4537160823	-1.4966012317	-0.1926806192
C	-1.7907978465	0.0055971031	-0.211847587
C	-1.7245259931	0.6922285481	1.1566294041
C	-0.2832841767	0.8918110827	1.6237243266
C	0.5293426358	1.5844128329	0.533280729
C	0.4012553191	0.8292984874	-0.7849992271
O	-0.9657089749	0.687035193	-1.1523306366
C	1.0790226171	1.5342642961	-1.943430653
H	-7.5684598646	0.9779916792	0.7594040901
H	-5.5369093182	-1.0391259098	-2.4588321058
H	-5.4907541646	-0.5761063538	3.1319890619
H	-8.0484564943	1.1834075406	-1.4727092282
H	-4.2463263576	-3.4505333659	-0.8669262313
H	-3.4140371372	-2.2876130497	-1.8881208122
H	-2.108590814	-3.4939188227	-0.0131517363
H	-0.6508383715	-1.7098629933	0.5208270945
H	-1.061802462	-1.7438839726	-1.1868359799
H	-2.7949949601	0.1342637308	-0.6241965078
H	-2.2910380452	0.1280210232	1.9025483618
H	-2.2056392382	1.672596383	1.0610239926
H	0.17611461	-0.0750405898	1.8669766663
H	-0.2697230563	1.476808101	2.5491581289
H	1.5856791388	1.6585939928	0.8164756459
H	0.157450192	2.6064085537	0.3842242401
H	0.8538517287	-0.1693256518	-0.6612231969
H	2.1487067098	1.6557301426	-1.7496018636
H	0.6362427583	2.5233906913	-2.0922718896
H	0.953393306	0.9618088357	-2.8660280374

Table S30. Sampled and DFT geometry optimized conformers related to calc_**1c**, with energy values (Hartree) and related % contribution on the final Boltzmann distribution for the three employed functional/basis set combinations.

Conformer	MPW1PW91/6-31g(d,p)		MPW1PW91/6-311+g(d,p)		B97-2/cc-pVTZ	
	**Energy (Hartree)**	**% contribution on the Boltzmann distribution**	**Energy (Hartree)**	**% contribution on the Boltzmann distribution**	**Energy (Hartree)**	**% contribution on the Boltzmann distribution**
calc_**1c** conf_1	-997.7128633	30.73%	-997.9473639	23.32%	-997.906243	18.46%
calc_**1c** conf_2	-997.7127837	28.25%	-997.9476911	32.98%	-997.906957	39.32%
calc_**1c** conf_3	-997.7119331	11.47%	-997.9466498	10.95%	-997.905516	8.54%
calc_**1c** conf_4	-997.7118445	10.45%	-997.9469827	15.57%	-997.906352	20.72%
calc_**1c** conf_5	-997.7115434	7.59%	-997.9463331	7.83%	-997.905056	5.25%
calc_**1c** conf_6	-997.7112678	5.67%	-997.9456922	3.97%	-997.904662	3.46%
calc_**1c** conf_7	-997.7103432	2.13%	-997.9447668	1.49%	-997.903149	0.70%
calc_**1c** conf_8	-997.7098307	1.24%	-997.9449555	1.82%	-997.903769	1.34%
calc_**1c** conf_9	-997.7093698	0.76%	-997.9438321	0.55%	-997.902300	0.28%
calc_**1c** conf_10	-997.7091792	0.62%	-997.9438379	0.56%	-997.903029	0.61%
calc_**1c** conf_11	-997.7090163	0.52%	-997.9432388	0.30%	-997.901909	0.19%
calc_**1c** conf_12	-997.7079788	0.17%	-997.9427438	0.17%	-997.902746	0.45%
calc_**1c** conf_13	-997.7075991	0.12%	-997.9426792	0.16%	-997.902091	0.23%
calc_**1c** conf_14	-997.7074178	0.10%	-997.9424855	0.13%	-997.902011	0.21%
calc_**1c** conf_15	-997.7073956	0.09%	-997.9422959	0.11%	-997.900200	0.03%
calc_**1c** conf_16	-997.7066684	0.04%	-997.9413475	0.04%	-997.901716	0.15%
calc_**1c** conf_17	-997.7059392	0.02%	-997.9409557	0.03%	-997.899743	0.02%
calc_**1c** conf_18	-997.7057660	0.02%	-997.9408909	0.02%	-997.899825	0.02%
calc_**1c** conf_19	-997.7038946	0.00%	-997.9392581	0.00%	-997.897492	0.00%
calc_**1c** conf_20	-997.7015944	0.00%	-997.9351053	0.00%	-997.892976	0.00%

Table S31. Cartesian coordinates of the optimized geometries for the conformers related to calc_**1c**. The related energies and % contribution on the final Boltzmann distribution for the three employed functional/basis set combinations are reported in Table S30.

calc_**1c** conf_1

C	-7.4302241253	-0.514733415	3.4644105364
C	-6.4045823942	-1.4037444659	3.7621244368
C	-5.344891342	-1.6203565001	2.8684921032
C	-5.3292943839	-0.9464897701	1.6639444379
C	-6.3679069808	-0.0557975357	1.3292320308
C	-7.4149551492	0.178310847	2.2533308167
O	-8.3968287604	1.0433702131	2.0074970962
O	-6.3704707534	-2.0967609776	4.921888837
C	-4.208091769	-1.0862066147	0.6748972863
C	-4.7440454681	-0.9158728226	-0.7352159142
O	-5.4467054963	0.340429772	-0.8612498573
C	-6.3613478112	0.6684084897	0.0639459614
O	-7.1455333817	1.574978675	-0.198808145
C	-3.7157810299	-0.9358480939	-1.8565630326
C	-2.6524992941	0.1539341574	-1.814197291
C	-1.9513557805	0.3217114276	-3.1604536542
C	-0.815460357	1.3363096926	-3.0501029768
C	0.1059557734	0.9757232826	-1.8870017594
C	-0.6996073842	0.7774445875	-0.6065355059
O	-1.7135103878	-0.2007490641	-0.8068610749
C	0.1434413278	0.3054895188	0.5610654268
H	-8.2440279357	-0.3300131561	4.1585979961
H	-4.5568253474	-2.3117403017	3.1436990574
H	-8.17936714	1.4735583236	1.1404721981
H	-7.1453758686	-1.8668628322	5.4513072441
H	-3.7223837507	-2.06210376	0.763965652
H	-3.4332690152	-0.3361397031	0.8634645624
H	-5.4802676883	-1.7079032765	-0.9271346627
H	-3.2156996052	-1.9108024963	-1.8598739206
H	-4.2642090351	-0.8494379674	-2.8005969296
H	-3.1265630107	1.1112464286	-1.5416800202
H	-1.5536564703	-0.6532157568	-3.4715349694
H	-2.6792788636	0.6351380168	-3.9170198118
H	-0.2531521676	1.3896168807	-3.9882161909
H	-1.2393867387	2.3351899013	-2.8805077448
H	0.6399166153	0.0423581671	-2.1067353012
H	0.8609567822	1.7547761581	-1.7316709274
H	-1.1845408026	1.7345593983	-0.3458179261
H	0.9309832167	1.0306784773	0.7846619531
H	0.6121291221	-0.6546212562	0.3258550204
H	-0.4722873486	0.1761673511	1.454924963

calc_**1c** conf_2

C	-7.3076771057	-1.1505263238	3.4048008439
C	-6.0496592961	-0.8786267753	3.9278987954
C	-5.0581247974	-0.2568543248	3.1540816957
C	-5.3362592072	0.0780493656	1.8443034401
C	-6.5958966076	-0.2046523021	1.2838783914
C	-7.5982292008	-0.803848414	2.0842841738
O	-8.8206065909	-1.0524837733	1.6198590911

O	-5.718006277	-1.1903120599	5.2005803353
C	-4.3413064866	0.7806443972	0.9640186634
C	-4.5458642125	0.3736724463	-0.4836671022
O	-5.9162363794	0.5985584128	-0.8829317759
C	-6.904753458	0.1524337127	-0.0965271871
O	-8.0337560377	0.079724985	-0.5720607751
C	-3.707991812	1.1423323941	-1.4920799762
C	-2.2076552486	0.9695144479	-1.3089287802
C	-1.4040978022	1.6445488398	-2.4174734509
C	0.0840510004	1.340512249	-2.2580269805
C	0.3028701574	-0.1654842436	-2.1274703302
C	-0.5842828943	-0.7441364999	-1.0291725496
O	-1.9485921583	-0.4239995879	-1.272999068
C	-0.5004844331	-2.2531795392	-0.9222426073
H	-8.0849754249	-1.6166325776	4.0021351429
H	-4.092839238	-0.0507070402	3.6019139443
H	-8.8489762356	-0.6840522207	0.6986628071
H	-6.4733932526	-1.6119427705	5.6309427108
H	-3.3208025301	0.5365972435	1.2709908138
H	-4.4621186328	1.8686573229	1.059416014
H	-4.344930061	-0.697202778	-0.587456399
H	-3.9903871531	0.7999611569	-2.4930881618
H	-3.9589587753	2.207428779	-1.4317236608
H	-1.8984881648	1.4101877232	-0.3434539922
H	-1.7607062901	1.2648667076	-3.3830699071
H	-1.5876963266	2.7248544091	-2.403151198
H	0.6513551406	1.7425784619	-3.1038353289
H	0.4648060527	1.8426809547	-1.3582372503
H	0.0510627212	-0.6632534342	-3.0724746244
H	1.3532702927	-0.3898529699	-1.9101535927
H	-0.287724436	-0.2914046167	-0.0658657785
H	-0.8041956989	-2.7168436024	-1.8652344217
H	-1.1617323202	-2.6188063541	-0.1325469754
H	0.5219891801	-2.5670018011	-0.693894989

calc_**1c** conf_3

C	-7.5538907057	-1.3447939653	3.0346003195
C	-6.6838997021	-0.8246443147	3.9848858879
C	-5.6177136657	0.0104724633	3.6173917067
C	-5.4236663114	0.3078310232	2.2839213619
C	-6.2784649592	-0.2224788186	1.2990730035
C	-7.369018542	-1.0384768598	1.6855623168
O	-8.2336556537	-1.5305253666	0.8010667442
O	-6.8196882025	-1.0885992785	5.3030903574
C	-4.3261512237	1.2187726979	1.8122581204
C	-3.8576469848	0.803220257	0.428347968
O	-4.9825864741	0.7569092596	-0.4789262043
C	-6.0862905324	0.0890431062	-0.1122105726
O	-6.8940854011	-0.2136271855	-0.9850645739
C	-2.8345698327	1.7567561119	-0.1678536396
C	-1.9925900915	1.1872670368	-1.3071864521
C	-2.7460983417	0.8629497289	-2.5944446142
C	-1.7906421164	0.2386946857	-3.6100027165
C	-1.0638722421	-0.9497186787	-2.9840322715
C	-0.4004205504	-0.5433136446	-1.6717121664
O	-1.351674101	0.0330832117	-0.7859559268
C	0.2346303459	-1.7056872056	-0.9354742082
H	-8.3900733957	-1.97824557	3.3136898803

H	-4.9672204649	0.4044855297	4.3896395756
H	-7.9736061243	-1.1580776907	-0.0809409649
H	-7.5788678633	-1.6720370015	5.4332457373
H	-3.4805250119	1.1971248046	2.5056428482
H	-4.6899496206	2.2548841597	1.7761050305
H	-3.4318562344	-0.204924604	0.4735294366
H	-3.3448991549	2.6675163609	-0.4993166318
H	-2.1445374695	2.0415482124	0.6340661049
H	-1.2177408531	1.9376901668	-1.5479505596
H	-3.5646557174	0.1722240392	-2.3729166894
H	-3.2007855783	1.7768404803	-2.9927984186
H	-2.3337447082	-0.0700719288	-4.5091295245
H	-1.052703099	0.9876637657	-3.9295347718
H	-1.7785196191	-1.7562799249	-2.7763155094
H	-0.307991323	-1.3517128137	-3.6684222447
H	0.3708833202	0.216437064	-1.8915553746
H	1.0126806516	-2.1706882347	-1.5475459041
H	-0.5199249809	-2.4620572866	-0.7001470984
H	0.6840325348	-1.3664537929	0.0013206384

calc_**1c** conf_4

C	-7.9641493562	-0.9651084611	3.2559244832
C	-7.0736344323	-0.4394728669	4.18373287
C	-5.874416458	0.1678892698	3.7799100992
C	-5.5710228921	0.2306920565	2.4355637001
C	-6.4462816001	-0.3128007607	1.475379385
C	-7.6671063788	-0.8954173541	1.8939053868
O	-8.5515212589	-1.385872648	1.0286797363
O	-7.3137330936	-0.479338293	5.512405506
C	-4.3282969022	0.8974092314	1.9179418455
C	-3.8608269006	0.2107163124	0.6466548493
O	-4.9259147637	0.1838488562	-0.3267287227
C	-6.1391847488	-0.249043834	0.0514202856
O	-6.938906774	-0.5587123306	-0.8253819916
C	-2.6806887604	0.8993418456	-0.0096465872
C	-2.2137522154	0.2786035199	-1.321506283
C	-1.7543516454	-1.1724470206	-1.203785843
C	-1.1630140645	-1.6497129933	-2.5299898635
C	-0.0983819301	-0.6725083117	-3.0232576722
C	-0.649483738	0.7504943804	-3.0436549978
O	-1.1473186107	1.1049937585	-1.7613270575
C	0.3886158137	1.7903295502	-3.4146657526
H	-8.9007476995	-1.4215895258	3.5602768925
H	-5.2117249847	0.5773198358	4.5335336234
H	-8.1953862874	-1.1922432946	0.1234336935
H	-8.160628067	-0.9172368672	5.6695769256
H	-3.5300656876	0.87230259	2.6655176767
H	-4.5341956163	1.9537157265	1.6992930913
H	-3.6141105862	-0.8329651845	0.8796529891
H	-2.9338995613	1.9477730589	-0.2002936083
H	-1.8415460872	0.8943321989	0.6959361218
H	-3.0349097602	0.3295982731	-2.0538946501
H	-0.9981114511	-1.2391171484	-0.4102977562
H	-2.5951972362	-1.812857986	-0.9156161531
H	-0.7469558551	-2.6571879417	-2.4245281009
H	-1.965686263	-1.7182082009	-3.2762462122
H	0.7719779784	-0.696394303	-2.3549835891
H	0.2510024825	-0.9499834339	-4.0243629688

H -1.4835313189 0.7919662976   -3.7665024124
H 0.785795247   1.5989746326   -4.4156435826
H 1.218137414   1.7691007337   -2.7016616682
H -0.0508459499 2.790816632   -3.3987636879

calc_**1c** conf_5

C -7.0696408206 0.2747556966   3.4375822878
C -5.875547279   -0.3085734157 3.8414532968
C -5.0075061315 -0.9084414127 2.9155945132
C -5.3462082242 -0.9074966502 1.5778530777
C -6.539774651   -0.3034984702 1.1393611496
C -7.4197633049 0.2734338705   2.0855983637
O -8.5792784229 0.8229310669   1.7324116569
O -5.4908076868 -0.335402475   5.1369112358
C -4.5114189802 -1.5769249601 0.5248218945
C -4.6106038236 -0.8304986136 -0.7967566944
O -5.9966289645 -0.6846631084 -1.1821068705
C -6.8951065613 -0.2753497644 -0.2768382938
O -7.991747915   0.0991227899   -0.6818047707
C -3.9521510721 0.5466263345   -0.8229980464
C -2.4386957597 0.5123487452   -0.6690345765
C -1.8072300742 1.8923980041   -0.8317981166
C -0.2839086201 1.7890729623   -0.7907861509
C 0.1999155757   0.7368120228   -1.7865287194
C -0.5323637494 -0.5846581446 -1.5712721631
O -1.939457523   -0.3905787146 -1.6420209128
C -0.182359549   -1.6387541559 -2.6019432731
H -7.7527378307 0.72649847   4.1500611918
H -4.0929242249 -1.3681863603 3.2720122808
H -8.6693433911 0.6774221073   0.7543458035
H -6.163713228   0.0999785496   5.676642855
H -4.8760199428 -2.6010066845 0.3736697758
H -3.4659584102 -1.6584876384 0.8333820643
H -4.1793412641 -1.4349438203 -1.5941068829
H -4.1999979704 1.0083848655   -1.7846317069
H -4.3741534759 1.1819390362   -0.0365732502
H -2.1743539369 0.1316472037   0.334304015
H -2.1273663656 2.3082132626   -1.7953015736
H -2.1761128247 2.5627502486   -0.0473775374
H 0.1736848315   2.7612042558   -1.0016851207
H 0.0347956028   1.5056983733   0.2215152245
H 0.006614115   1.0753421772   -2.8123137047
H 1.2804375799   0.5790993328   -1.693476788
H -0.2835337874 -0.9612504501 -0.5627416033
H -0.4330833727 -1.2847502428 -3.6061140913
H -0.741161144   -2.5591867329 -2.4143021049
H 0.8865525775   -1.8680275606 -2.5710077347

calc_**1c** conf_6

C -7.2329173675 -1.3152185264 3.2402709552
C -5.9002747923 -1.3486937135 3.6324195284
C -4.8951857156 -0.7582878565 2.8514783919
C -5.2376824998 -0.1431411063 1.6642766885
C -6.578366568   -0.1135421971 1.2343825734
C -7.5872422613 -0.685235689   2.0463391364
O -8.8749120625 -0.6452494772 1.7098909812
O -5.5054156137 -1.9425454874 4.7807168603

C	-4.2285938598	0.5452276391	0.7913226498
C	-4.6455542062	0.4363285269	-0.6631675422
O	-5.9834081019	0.957774813	-0.8398188993
C	-6.9539800861	0.5409468086	-0.0133983654
O	-8.1185201527	0.7422433311	-0.344260144
C	-3.7910972141	1.2186170662	-1.6495638894
C	-2.3852981085	0.6911459389	-1.9158330791
C	-2.3138463702	-0.7512686318	-2.4148210564
C	-0.8574581736	-1.150812013	-2.6457730938
C	-0.0280791585	-0.8639206549	-1.395100703
C	-0.2295575033	0.5761586892	-0.932247871
O	-1.6120026908	0.849181475	-0.7356066975
C	0.4745220145	0.892286513	0.3717438242
H	-8.0172251113	-1.7554867556	3.8480563898
H	-3.868859365	-0.7935276286	3.1979925184
H	-8.9287424293	-0.1088617254	0.8769075785
H	-6.2785984739	-2.3083569952	5.2301479914
H	-3.2282482629	0.1266925027	0.9146759756
H	-4.1613893399	1.6072341243	1.0626213804
H	-4.6846280214	-0.6222646674	-0.9481061657
H	-4.3311102862	1.2385395579	-2.6022685365
H	-3.7147735228	2.255455047	-1.3050878221
H	-1.9502852305	1.3367801558	-2.6994296313
H	-2.7578980055	-1.4196124924	-1.6676643748
H	-2.8992678108	-0.8559845277	-3.3353991038
H	-0.7863407758	-2.2075058262	-2.9233548809
H	-0.4533538136	-0.5761025659	-3.4900806703
H	-0.3332681201	-1.5350147039	-0.5818851337
H	1.0362236261	-1.0449186381	-1.5829530908
H	0.1440947358	1.252413294	-1.7215414719
H	1.5546393215	0.7562977116	0.2677789022
H	0.1187340698	0.2317969783	1.1682387164
H	0.2801673077	1.925431707	0.6701011812

calc_**1c** conf_7

C	-7.6031760476	0.3254319419	3.1524191012
C	-6.7553354562	-0.4346459666	3.9481054519
C	-5.6775393392	-1.1442812991	3.3952067186
C	-5.4523980012	-1.0736139838	2.0360940961
C	-6.2839446386	-0.2936865297	1.2098987569
C	-7.3837988631	0.394864811	1.7752074977
O	-8.2243960792	1.1153087895	1.0363939219
O	-6.9240659298	-0.5365147121	5.2848127254
C	-4.3636793982	-1.8462405571	1.3490946951
C	-3.8287780576	-1.0748510246	0.1504574904
O	-4.9203809288	-0.7275182799	-0.7334027315
C	-6.0405353616	-0.1948271637	-0.2249770129
O	-6.8216436133	0.3488591007	-1.0007681505
C	-3.0193779192	0.1640728307	0.5300048148
C	-2.1332973562	0.7230183814	-0.5801828581
C	-2.8610842652	1.3251304735	-1.7799338169
C	-1.8447757878	1.7814643679	-2.8244508456
C	-0.8999960618	0.6331143669	-3.1711814605
C	-0.2740269143	0.0530696889	-1.9066763823
O	-1.28023875	-0.3390503924	-0.9814413497
C	0.5820213956	-1.1699630845	-2.1669825565
H	-8.4473680543	0.8639026826	3.5715651353
H	-5.0477804989	-1.7380801432	4.0475282033

H	-7.9364778554	0.9988760344	0.0936470878
H	-7.6910626643	-0.0115188207	5.5490199284
H	-4.7703370086	-2.803458629	0.9980293204
H	-3.5467705464	-2.0806567319	2.0370674559
H	-3.1999421059	-1.7237924983	-0.4600832352
H	-3.6825085141	0.9498383953	0.9071456384
H	-2.3594158312	-0.1195479291	1.3574232223
H	-1.5108974337	1.5176602072	-0.1297067187
H	-3.5343442802	0.5785738454	-2.2110359282
H	-3.4841755005	2.1634857442	-1.4480322231
H	-2.3543583601	2.1472774163	-3.7217651044
H	-1.2616879708	2.6242898433	-2.427682133
H	-1.4548817541	-0.164293803	-3.6818282802
H	-0.1085501831	0.9674948268	-3.8517250517
H	0.3434441419	0.837073145	-1.4326067935
H	1.4064546879	-0.9261161116	-2.84297941
H	-0.0182196909	-1.9623308024	-2.6239650999
H	0.9993267959	-1.5518184302	-1.2317141191

calc_**1c** conf_8

C	-6.9856162526	0.1575908949	3.4788579687
C	-6.5784694713	-1.1438056255	3.7427891714
C	-6.0381251518	-1.9573549777	2.7341644408
C	-5.9007019441	-1.4470504485	1.4600888507
C	-6.2858634508	-0.1241465128	1.1702525038
C	-6.8529731106	0.6775862115	2.1898704217
O	-7.2677901036	1.9219310376	1.9656507354
O	-6.6847532568	-1.6949682455	4.9714729648
C	-5.394640773	-2.26848598	0.3093142576
C	-4.6464566894	-1.3988768532	-0.6924028407
O	-5.4614639182	-0.2757703277	-1.0941946833
C	-6.1208891947	0.4351522806	-0.1659722504
O	-6.5670693831	1.5297434135	-0.4954621586
C	-3.2916918106	-0.9205251665	-0.187022105
C	-2.5660988184	0.0529297556	-1.1093219318
C	-2.2934708712	-0.4862845472	-2.5109547063
C	-1.4335200509	0.5016789284	-3.2989192466
C	-0.1903479769	0.8855569129	-2.4997320882
C	-0.5791750036	1.3519347759	-1.0995655401
O	-1.3498695493	0.3547814401	-0.4440603228
C	0.6137414547	1.6396727064	-0.2103348142
H	-7.4169512699	0.7872538029	4.2506851743
H	-5.7446580568	-2.9720072726	2.9770661662
H	-7.1352853293	2.0904368491	0.9968633383
H	-7.0632054899	-1.0460544343	5.5792563046
H	-6.2467678069	-2.7394963376	-0.1970250979
H	-4.7427715048	-3.0753976167	0.6564026603
H	-4.5153618224	-1.9546758696	-1.6233349515
H	-3.3998051771	-0.4454541437	0.7938300412
H	-2.6523167342	-1.7996704413	-0.0416433313
H	-3.161601521	0.9758194891	-1.202604278
H	-1.7725160376	-1.4490814966	-2.4233732062
H	-3.2383369844	-0.6634345436	-3.0359856775
H	-1.1558422127	0.0792457223	-4.2703854041
H	-2.0241463308	1.4039528456	-3.5066055377
H	0.4762944178	0.0189404086	-2.4024214248
H	0.3728230496	1.6746264899	-3.011098709
H	-1.1942507063	2.2646297418	-1.1928927234

```
H  0.2823603799   1.9551894302   0.7821222654
H  1.2333070029   2.4318982586   -0.6402770912
H  1.2272774598   0.7409894448   -0.0970971448
```

calc_**1c** conf_9

```
C  -6.8689042652  -0.4293935992  3.4232670961
C  -5.704460115   -1.1844858334  3.4891072731
C  -4.9549224957  -1.4711825659  2.3380533991
C  -5.3808092852  -0.9845676491  1.1190199024
C  -6.5448239729  -0.1980769793  1.0283306106
C  -7.3063066681  0.0629695423   2.1924444877
O  -8.4347170771  0.7691379673   2.1586162041
O  -5.2388038432  -1.6839221784  4.6559546973
C  -4.6753220604  -1.3000684898  -0.1677056553
C  -4.806075021   -0.1478944244  -1.1523730394
O  -6.1954480017  0.2183516099   -1.3239497908
C  -6.9899397749  0.3425085882   -0.2509173838
O  -8.071073881   0.904374939    -0.4014931338
C  -4.0183645231  1.1110228699   -0.7874037503
C  -2.5127210817  1.0759939081   -1.0368255589
C  -2.1004167431  0.8491758819   -2.4906570696
C  -0.5768559525  0.8451432021   -2.6038215348
C  0.0216974868   -0.147766479   -1.6091077599
C  -0.5108552865  0.1095754303   -0.2021192265
O  -1.9334895405  0.0903886376   -0.1951007321
C  -0.0497090426  -0.9176479359  0.8117824716
H  -7.4610465258  -0.21659934    4.3078679229
H  -4.0568694719  -2.0709098833  2.4278511369
H  -8.6008667113  0.9818774124   1.203584973
H  -5.8339142811  -1.4236268634  5.3712480533
H  -5.127653405   -2.1962680065  -0.6118795394
H  -3.6164290483  -1.5031043447  -0.0006070384
H  -4.5203709738  -0.4862948675  -2.1498045671
H  -4.4317942006  1.9343208444   -1.3796643728
H  -4.1893218427  1.3545396314   0.2665453575
H  -2.1207584794  2.0626394821   -0.7315190792
H  -2.4906495907  -0.1145929201  -2.8384210875
H  -2.5381881447  1.6273624533   -3.1259805796
H  -0.2659406605  0.6043774967   -3.6256673052
H  -0.1947807229  1.8521075419   -2.3881929585
H  -0.245266612   -1.1726233414  -1.8978001599
H  1.1157691389   -0.0858503104  -1.6059324229
H  -0.1804651521  1.1131471298   0.1191493566
H  -0.3649547574  -1.9209056341  0.5092787344
H  -0.4786563626  -0.7037756904  1.7939230366
H  1.0404789487   -0.9104572325  0.8979190325
```

calc_**1c** conf_10

```
C  -7.8142626654  -0.1171483809  3.4386800573
C  -6.731993722   -0.6762385619  4.1067375596
C  -5.5151254059  -0.9170690674  3.4497523606
C  -5.4000527468  -0.6053421317  2.1107388742
C  -6.4883122388  -0.0575853259  1.4044364785
C  -7.6995455244  0.2113825957   2.0870204945
O  -8.7445374101  0.7710670823   1.4821588569
O  -6.7889928932  -1.0094520591  5.4144877322
C  -4.1217254732  -0.7912853818  1.3430891768
```

C	-4.4404392763	-1.126548554	-0.104499541
O	-5.2793794794	-0.1020776321	-0.6796096246
C	-6.3772458026	0.2830035016	-0.0091187135
O	-7.2315895161	0.9177856574	-0.6185326807
C	-3.2445698577	-1.3262504495	-1.0208871702
C	-2.1973144297	-0.2181132527	-1.097713709
C	-2.6483623944	1.0440759663	-1.8274073558
C	-1.4779790369	2.012666571	-1.9884923545
C	-0.2820399582	1.3025054209	-2.6180524729
C	0.0463241459	0.0279168334	-1.8464316446
O	-1.0946640433	-0.8141172994	-1.7657427923
C	1.1504356045	-0.7915101817	-2.4838168099
H	-8.7522058092	0.0879823435	3.945019922
H	-4.6897402406	-1.3419022623	4.0092416029
H	-8.4526543956	0.9856148439	0.5588190458
H	-7.6682386042	-0.8023198196	5.7577358661
H	-3.5125288721	-1.5877366678	1.7807187828
H	-3.5305137523	0.1315318357	1.3916737719
H	-5.027797957	-2.0540881334	-0.1186815891
H	-2.7275147869	-2.2332849566	-0.6881912608
H	-3.6154713218	-1.5311445826	-2.0306726762
H	-1.8712730217	0.0602177985	-0.079182751
H	-3.0315673744	0.7522032607	-2.8128581433
H	-3.4771656851	1.5151918994	-1.2925394618
H	-1.7775212933	2.8767943262	-2.5905703937
H	-1.1898542568	2.4037686951	-1.0027809578
H	-0.5138061403	1.0282708272	-3.6550640647
H	0.5961621589	1.9579795264	-2.6418030221
H	0.3456325173	0.3089354341	-0.8201622531
H	2.0766610047	-0.2124803747	-2.540228559
H	0.8641107432	-1.0888132268	-3.4969418432
H	1.339659211	-1.6973861174	-1.9023287373

calc_**1c** conf_11

C	-7.3872487536	0.6971484287	2.2474686633
C	-6.7090704648	0.486869927	3.4411119493
C	-5.568408246	-0.3288559026	3.4966846151
C	-5.1079415417	-0.921271148	2.3382459526
C	-5.7634176995	-0.7067051082	1.1115031644
C	-6.9295666799	0.0936298199	1.0742995191
O	-7.6142073876	0.2955122677	-0.0489962151
O	-7.1108565206	1.0462069742	4.6044516561
C	-3.9279206329	-1.8514033386	2.3191912328
C	-3.1907545166	-1.7423671212	0.9915213522
O	-4.1002896254	-1.9577573781	-0.1046875089
C	-5.2775282371	-1.318593504	-0.1214852795
O	-5.9159248761	-1.3087353142	-1.1691882826
C	-2.4223187097	-0.4289558411	0.8433102566
C	-1.9266493581	-0.141310583	-0.5689554581
C	-0.7083687372	0.7786797578	-0.6046286239
C	-0.3473036668	1.1154496266	-2.0512199687
C	-1.5736927815	1.6253030264	-2.8079791976
C	-2.7492369759	0.6658156855	-2.6401381117
O	-3.0078680709	0.4585268846	-1.2579041954
C	-4.0382175988	1.1676355286	-3.2574920396
H	-8.2792669553	1.3139385067	2.1998678441
H	-5.0750153622	-0.4801479475	4.4498544103
H	-7.1709064594	-0.2539912262	-0.7464911684

H	-7.8978649992	1.582810683	4.4423906829
H	-4.2765005184	-2.8834144345	2.4519725293
H	-3.2428021688	-1.6349171684	3.144348501
H	-2.4888092044	-2.5740704537	0.8887996628
H	-3.0410162974	0.4187551753	1.1561717818
H	-1.5711452428	-0.4780010733	1.533014667
H	-1.6646714413	-1.089948618	-1.0711326047
H	-0.9496361484	1.696279248	-0.0529973386
H	0.1372409281	0.3018007796	-0.0949175416
H	0.4627589518	1.8517968904	-2.0851836641
H	0.0313356634	0.2103049924	-2.5454647443
H	-1.8727802324	2.6065088313	-2.41785612
H	-1.3461473486	1.7533899629	-3.8724364276
H	-2.482907864	-0.3050254326	-3.0930666782
H	-3.9192733478	1.3091271017	-4.3356781876
H	-4.32520445	2.1247406021	-2.8113710536
H	-4.8405964223	0.4482408927	-3.0799380308

calc_**1c** conf_12

C	-7.7554800982	-1.076401486	3.1647219607
C	-6.992884387	-0.3442361131	4.0661564858
C	-5.9190777476	0.4497990563	3.6358204564
C	-5.6091291491	0.4905741566	2.2913531965
C	-6.3517341484	-0.2580975765	1.3599795257
C	-7.4528899573	-1.0294706247	1.8027294695
O	-8.2181543983	-1.7190950571	0.9599995766
O	-7.245996174	-0.3530580054	5.3939618873
C	-4.4987117409	1.3404922478	1.7430497111
C	-3.8945641329	0.6863000627	0.5112480907
O	-4.9247676238	0.4169281228	-0.4607402284
C	-6.0394299564	-0.2104642256	-0.0653881499
O	-6.7643005591	-0.7020957039	-0.924880518
C	-2.8429918382	1.5508817007	-0.1767673121
C	-1.7302467089	0.7658888841	-0.8562982359
C	-0.7129177944	1.6671243198	-1.552340015
C	0.3229697825	0.8222979125	-2.2923133483
C	-0.3729693061	-0.1941219617	-3.1958788064
C	-1.4073909804	-0.9947491491	-2.4091335278
O	-2.3313510687	-0.1209805709	-1.7752043455
C	-2.2204225349	-1.9377413861	-3.2724969514
H	-8.5980976549	-1.6790328488	3.4890878046
H	-5.3557515562	1.0160590259	4.3685672093
H	-7.8876846887	-1.5073893356	0.0478755531
H	-8.0016475537	-0.9293592091	5.5684700926
H	-3.7195646464	1.494027713	2.4958139564
H	-4.8844828302	2.3324226376	1.4703507895
H	-3.4608876599	-0.2810644272	0.7947156523
H	-3.3501888017	2.1852483388	-0.911085202
H	-2.3835126035	2.2150131382	0.5644925283
H	-1.1983086141	0.1729605143	-0.0869788021
H	-1.248590126	2.3074600693	-2.26443437
H	-0.2298811926	2.3237321562	-0.8192672594
H	0.9968231872	1.4607744428	-2.8732475921
H	0.9472105879	0.2913848542	-1.5606346516
H	-0.8892009494	0.3245474874	-4.0135721695
H	0.3558114797	-0.8752124811	-3.6497584055
H	-0.8810984604	-1.5728175375	-1.6278226659
H	-2.9632575371	-2.4630023774	-2.6675126166

H	-1.5723211731	-2.6756583151	-3.7540959483
H	-2.7499286848	-1.3778684491	-4.0485428248

calc_**1c** conf_13

C	-7.5876889952	-0.75212149	3.6003755176
C	-6.4039861811	-0.2923254145	4.1640807557
C	-5.3647603653	0.2124762422	3.3668373806
C	-5.5207523925	0.2385247063	1.9962927764
C	-6.7013056557	-0.2409229757	1.3982668758
C	-7.7547038856	-0.7175730894	2.2148569976
O	-8.9098697052	-1.1390445035	1.7058023341
O	-6.1930298854	-0.3006420637	5.4985429105
C	-4.4793377299	0.8003479163	1.0713453312
C	-4.500071867	0.0558394954	-0.2529277424
O	-5.8284318577	0.1172370981	-0.8196274457
C	-6.8780525724	-0.2079230119	-0.0491264975
O	-7.9459901992	-0.450320747	-0.6016506992
C	-3.5589573998	0.609337958	-1.3171803764
C	-2.2179067341	-0.1088318282	-1.4247805068
C	-1.3411221888	-0.0449418615	-0.1757097305
C	0.027524079	-0.6674851844	-0.4509651684
C	0.6481924817	-0.055294117	-1.7041825517
C	-0.3314246474	-0.125569162	-2.8716478805
O	-1.5608638933	0.495874167	-2.5236881686
C	0.1719781246	0.5696308034	-4.1203823341
H	-8.4019015928	-1.1277113103	4.2121577377
H	-4.4623725522	0.5752070431	3.8452910125
H	-8.8601120187	-0.9777850675	0.728149615
H	-6.9706364404	-0.6656978582	5.9410167731
H	-3.4846058041	0.7469258513	1.5208331594
H	-4.6868589375	1.8613502792	0.8775476941
H	-4.2850863798	-1.0068941358	-0.0735776749
H	-4.0563337132	0.5160638809	-2.2862560329
H	-3.3798662784	1.6781409262	-1.1545886417
H	-2.40529334	-1.1727179598	-1.6614078335
H	-1.2221304934	1.007200512	0.1141639831
H	-1.8286388605	-0.5655096148	0.6567744155
H	0.687756108	-0.5392888797	0.413132384
H	-0.0916106235	-1.7496781603	-0.5967017253
H	0.8959787855	0.9983924324	-1.5232101015
H	1.5792796101	-0.5691433027	-1.9686057836
H	-0.5291818774	-1.188331207	-3.0993345796
H	1.1017539361	0.1109582797	-4.4687752219
H	0.3612653452	1.6272921883	-3.9150173331
H	-0.5708434027	0.503953165	-4.9191236244

calc_**1c** conf_14

C	-7.5733800752	-0.7023863918	3.5974068427
C	-6.3252261167	-0.395695361	4.1248720588
C	-5.2674923663	0.0177608288	3.300350087
C	-5.471195805	0.1080926105	1.9385696797
C	-6.7197147351	-0.2167284369	1.3762415492
C	-7.7877114395	-0.6024370734	2.2217559714
O	-9.00107192	-0.8775805101	1.7494866918
O	-6.065666302	-0.4731802236	5.4485084998
C	-4.4025794642	0.5790539613	0.9930275878
C	-4.5655832768	-0.0945157505	-0.3580337456

O	-5.8997517934	0.121254373	-0.8637110639
C	-6.9476977915	-0.1167596389	-0.0607707991
O	-8.0559496641	-0.225230449	-0.5746636186
C	-3.6363587575	0.3955548945	-1.4562097063
C	-2.1552973725	0.0978554398	-1.2102983625
C	-1.365437261	1.2724555835	-0.6332301026
C	0.1148752444	0.917262145	-0.5180111701
C	0.6354984288	0.4033523203	-1.8579598968
C	-0.2575444349	-0.7154983384	-2.3861522215
O	-1.609921962	-0.2842418995	-2.4628422418
C	0.129816985	-1.1875091392	-3.7729949946
H	-8.4004955213	-1.0074719903	4.2307007129
H	-4.312204918	0.2600843675	3.7515215502
H	-8.9711514622	-0.6925739986	0.7752526955
H	-6.8609006243	-0.7680059998	5.9115858052
H	-3.4110520465	0.3614509599	1.3998986448
H	-4.4698541895	1.6676763156	0.8629069687
H	-4.4405776143	-1.178610703	-0.2316617599
H	-3.9237997697	-0.1118868741	-2.3809081786
H	-3.7982296571	1.4676580393	-1.615069502
H	-2.062286095	-0.7608320793	-0.5174839976
H	-1.491554523	2.1278244449	-1.3087299258
H	-1.7719055099	1.5680806673	0.3402012334
H	0.693895865	1.784317615	-0.1832273303
H	0.2448880605	0.1393790755	0.2468853728
H	0.6413626687	1.2180576383	-2.5931913444
H	1.6653783363	0.040749328	-1.764443093
H	-0.2037728949	-1.5657339057	-1.6821257951
H	0.0661311815	-0.3602808296	-4.4858858944
H	-0.5430733671	-1.9804818438	-4.1084663009
H	1.1535919605	-1.5722791717	-3.7771009062

calc_**1c** conf_15

C	-5.6452403417	0.0011588026	3.2361232899
C	-4.9803158058	-1.2133821803	3.1145861631
C	-4.8440134971	-1.8471819548	1.8719540301
C	-5.3689850325	-1.246760015	0.744456258
C	-6.0448120497	-0.0178704019	0.8394844386
C	-6.1940326004	0.6044191006	2.104218892
O	-6.8445417079	1.7560779775	2.2556854115
O	-4.4373926237	-1.8439245536	4.1803779778
C	-5.3256118477	-1.9174486302	-0.5976784524
C	-5.4227836725	-0.942643589	-1.7682994169
O	-6.4169373949	0.0817504405	-1.5454553435
C	-6.6234139222	0.618471764	-0.3373011381
O	-7.31627364	1.6308884633	-0.2774218802
C	-4.1267740158	-0.2907634005	-2.2490689689
C	-3.250126171	0.4265082167	-1.2320803626
C	-2.2707700558	1.3866585271	-1.9047318265
C	-1.3202205944	1.9867362934	-0.8721463654
C	-0.6702707667	0.8786248785	-0.0477406463
C	-1.7262876376	-0.0599440403	0.5301966342
O	-2.5521107183	-0.5758403429	-0.5086585917
C	-1.1227456397	-1.2499916936	1.2481929093
H	-5.7652946259	0.4926736927	4.196523834
H	-4.33135889	-2.8005279071	1.8193865411
H	-7.1983088113	1.9952639619	1.3590350368
H	-4.6018385092	-1.3202445583	4.9755507312

H	-6.1706821107	-2.6153539598	-0.6552343371
H	-4.4106201698	-2.5052082119	-0.699138263
H	-5.8283326831	-1.4848024775	-2.6264014019
H	-3.5113970077	-1.0661409956	-2.7209983853
H	-4.4099500168	0.415255598	-3.0370939258
H	-3.872514488	1.0058082834	-0.5299534479
H	-1.7016912406	0.8323076989	-2.6625746346
H	-2.8282662102	2.1738738366	-2.4244944097
H	-0.5590524531	2.6042754111	-1.3605182109
H	-1.8847617946	2.6525600899	-0.2055919567
H	0.004654567	0.2889595117	-0.6814446276
H	-0.0681523362	1.2998250161	0.7653790677
H	-2.3630786759	0.5069394705	1.2306174083
H	-0.5243806471	-1.8467982667	0.5531955723
H	-1.906767138	-1.8845869024	1.6665307101
H	-0.4755470233	-0.9166229531	2.0645316871

calc_**1c** conf_16

C	-7.6970936644	-1.0327655031	3.4056697935
C	-7.2957565351	0.1459129588	4.0194291181
C	-6.3227341141	0.9754952298	3.4384973739
C	-5.745787238	0.6060001776	2.2379694291
C	-6.1217439544	-0.5908394128	1.6059647122
C	-7.1266656423	-1.4050417033	2.1896816188
O	-7.5479868748	-2.5259727063	1.6090986102
O	-7.8844207181	0.4600069282	5.1952453212
C	-4.7268673322	1.4566122056	1.534317051
C	-3.7674468303	0.5777788745	0.7482121154
O	-4.496860211	-0.2774632951	-0.1476525793
C	-5.5293171268	-0.985696464	0.3314822943
O	-5.9413753143	-1.9285604322	-0.33573616
C	-2.7629971579	1.3728195972	-0.0690974554
C	-1.7690767874	0.5394525523	-0.8737773464
C	-0.473524346	1.2928236955	-1.1762410748
C	0.4303286901	0.4511267895	-2.0773040566
C	-0.340389821	-0.0444104268	-3.3004597648
C	-1.6424335552	-0.7173525514	-2.8751113576
O	-2.409574865	0.1716124074	-2.0770873275
C	-2.5224601513	-1.1297619392	-4.0372352345
H	-8.4591568035	-1.6548229317	3.857578025
H	-6.0289654518	1.900469672	3.9285266161
H	-7.0785137574	-2.5851711559	0.7377723687
H	-7.5321029101	1.301851983	5.5113502679
H	-4.1679157455	2.0693349869	2.2483119707
H	-5.2291131249	2.1443557802	0.8407615467
H	-3.2292358446	-0.0760198131	1.4490841849
H	-3.2921545309	2.034080501	-0.7639084122
H	-2.2124125733	2.0092959106	0.633966911
H	-1.5143169241	-0.3747997498	-0.3061458251
H	-0.7305701611	2.2350587354	-1.6771818884
H	0.0402174503	1.547638504	-0.2415798099
H	1.313198182	1.0242883848	-2.3798578701
H	0.7985112297	-0.4135076911	-1.508222886
H	-0.587959686	0.8008997238	-3.9549953859
H	0.2688702965	-0.7427929422	-3.8855043046
H	-1.3975969153	-1.6083616805	-2.2699480842
H	-2.0081410489	-1.856083371	-4.6731954938
H	-2.7818831753	-0.2574558564	-4.6445001205

H -3.4475749564 -1.5770359723 -3.6661768909

calc_**1c** conf_17

C	-6.8324690593	0.3461196024	3.4544488479
C	-5.7402888705	-0.4567041367	3.7571197673
C	-5.0605901875	-1.169668687	2.7565514605
C	-5.4821835392	-1.0575541762	1.4473963146
C	-6.5727494383	-0.234279119	1.1107411415
C	-7.2677491785	0.4563381737	2.1320356094
O	-8.3298142699	1.2162381539	1.8777291939
O	-5.2810928226	-0.600056364	5.0196997922
C	-4.8565079863	-1.8287230862	0.3219664041
C	-4.9081877577	-1.0296772588	-0.9728549898
O	-6.2660496571	-0.627097022	-1.2522471201
C	-7.0131138112	-0.0942141041	-0.2739592458
O	-8.0484634397	0.4825280724	-0.5904940783
C	-4.0060542685	0.1996966511	-1.031750653
C	-2.5099508017	-0.1279563527	-0.9898898105
C	-1.8463813647	0.1855524098	0.349455598
C	-0.3456131482	-0.0862940938	0.2789388303
C	0.2619511994	0.6412261102	-0.9189697901
C	-0.516101759	0.3247490159	-2.1939154994
O	-1.89581923	0.6204284572	-2.0261434256
C	-0.0420113902	1.1163443127	-3.3960166643
H	-7.3724267928	0.8875158809	4.2249279272
H	-4.2251942463	-1.8016729543	3.0353098008
H	-8.5117339748	1.1257977765	0.9062217641
H	-5.8238526947	-0.0689581263	5.6172573458
H	-5.4095506391	-2.7654553043	0.1761715409
H	-3.8242372366	-2.1058838976	0.5532870344
H	-4.6610961228	-1.6853164475	-1.8118479233
H	-4.2066227386	0.7073024401	-1.9788817256
H	-4.2706113875	0.8952781889	-0.2280564827
H	-2.3597245461	-1.2031530944	-1.2100350126
H	-2.0235238028	1.2449779888	0.5729397784
H	-2.3159128449	-0.3919074287	1.1538925292
H	0.1464559349	0.2187738202	1.2083707774
H	-0.1754003082	-1.1667397111	0.173835415
H	0.2267425334	1.7254453535	-0.7530246765
H	1.314443483	0.3657647388	-1.0507622192
H	-0.4156000904	-0.755653728	-2.4046861512
H	1.010121608	0.9045714548	-3.6067488513
H	-0.1504192834	2.1887002991	-3.209216362
H	-0.6336160697	0.8616161922	-4.2787961913

calc_**1c** conf_18

C	-6.8886100406	0.1203256729	3.5431486779
C	-5.863528391	-0.7819705815	3.7966693068
C	-5.1861511564	-1.4285799533	2.7506346726
C	-5.5416493181	-1.1509167021	1.4466789915
C	-6.5616138738	-0.2247674914	1.1621798763
C	-7.2561643939	0.3996037621	2.2253395468
O	-8.2556178292	1.2525615125	2.0147909264
O	-5.4698868769	-1.088328272	5.0524311328
C	-4.9092795374	-1.8392143245	0.2707195749
C	-4.8559996777	-0.9049410321	-0.931580941
O	-6.1802989091	-0.397538463	-1.2136434328

C	-6.9316791848	0.0863360387	-0.2135434502
O	-7.9124792292	0.7622412911	-0.5072156863
C	-3.8736266878	0.2625949024	-0.7999774945
C	-2.498764146	0.0155523162	-1.4150432301
C	-1.6881811302	-1.1121258373	-0.7792858036
C	-0.2805595757	-1.1614923323	-1.3737582663
C	0.373570773	0.2162437803	-1.311393632
C	-0.543430421	1.2676597915	-1.9286734524
O	-1.8140079908	1.2490681274	-1.2931365226
C	-0.0101250542	2.6799006165	-1.7983419393
H	-7.4262302758	0.6126183196	4.347438975
H	-4.4013774553	-2.1368545815	2.9899572661
H	-8.3989720247	1.2779094191	1.0332615049
H	-6.0014474743	-0.5852392857	5.6833521011
H	-5.5038688962	-2.7231984248	0.0068057532
H	-3.9053631681	-2.19023005	0.5204540324
H	-4.6224972435	-1.4789065822	-1.8326244813
H	-4.3000364804	1.1245354791	-1.3207876473
H	-3.7481085408	0.5529857729	0.248853905
H	-2.6287757183	-0.2151135368	-2.4890667716
H	-1.6320447393	-0.9352035583	0.3026414039
H	-2.1924135743	-2.0735303535	-0.9320885185
H	0.3293836603	-1.9065214203	-0.8521892537
H	-0.3430032233	-1.4855277417	-2.4215077262
H	0.5649366211	0.4941717696	-0.2671695205
H	1.3385838889	0.2125868129	-1.8307034496
H	-0.683620743	1.02628219	-2.9976720242
H	0.9559028088	2.7753056981	-2.3022964189
H	0.1203198545	2.9389020864	-0.7436010599
H	-0.7082846255	3.393815165	-2.2420569248

calc_**1c** conf_19

C	-5.8220617114	0.4257152175	3.2700119386
C	-5.3669409366	-0.8822096315	3.3816304109
C	-5.2990986331	-1.7277279634	2.2636291317
C	-5.6830540902	-1.2424507378	1.0295729054
C	-6.1310747832	0.0821912773	0.8851860026
C	-6.2169063299	0.918866311	2.0249935204
O	-6.6576640019	2.1719522791	1.9534614796
O	-4.9746074424	-1.4079055289	4.5624378004
C	-5.7169400373	-2.1060117794	-0.1970776081
C	-5.4856545102	-1.3209159775	-1.4864543664
O	-6.2673615241	-0.1071500606	-1.5209106143
C	-6.4952357616	0.6182724568	-0.4204274528
O	-7.000131122	1.728639518	-0.5664479712
C	-4.0285479236	-1.063201722	-1.8656220836
C	-3.1104849428	-0.3080417256	-0.9036066227
C	-3.174519606	1.2123902346	-1.0324398744
C	-2.1209364887	1.8668347312	-0.1413068939
C	-0.7448794928	1.2680986366	-0.4217767142
C	-0.7987947023	-0.2551236125	-0.3479681887
O	-1.7994083496	-0.762943304	-1.2193263347
C	0.5043862402	-0.9203153639	-0.7436575709
H	-5.8889890906	1.0814663587	4.1324413969
H	-4.9529384837	-2.7465836881	2.3931976054
H	-6.9219121013	2.320854311	1.0077780939
H	-5.069585019	-0.7406353947	5.2548937519
H	-6.703933112	-2.5826215786	-0.2526056661

H	-4.9820848158	-2.9133886712	-0.1293785311
H	-5.9098991254	-1.8998168655	-2.3107166667
H	-3.5745379888	-2.0493894342	-2.0174400671
H	-4.0160370756	-0.5644546317	-2.8410541772
H	-3.3323903835	-0.5924093831	0.1381005922
H	-2.9864430605	1.4688833388	-2.082776275
H	-4.1706742039	1.5895573629	-0.7868189723
H	-2.1103873136	2.9509631248	-0.294644619
H	-2.3852423776	1.7023187332	0.9121185611
H	-0.4120960296	1.5536172647	-1.4279497381
H	-0.0001200193	1.6466038719	0.2877939599
H	-1.0574674878	-0.5454952485	0.6864404525
H	1.316034863	-0.6021859484	-0.0830602498
H	0.7683561295	-0.6544327596	-1.7714919904
H	0.412262845	-2.0078140177	-0.684728355

calc_**1c** conf_20

C	-5.3228270666	0.1299785607	3.1837958212
C	-4.8788123361	-1.1853989271	3.2527719082
C	-4.8434312658	-1.9983539649	2.1118857781
C	-5.2521951506	-1.4766325296	0.8975547512
C	-5.7009346062	-0.151938334	0.8002173014
C	-5.7474353459	0.6540390351	1.9616352565
O	-6.1791847385	1.9148532518	1.9309764591
O	-4.4650927443	-1.7451301314	4.4124558595
C	-5.3002876191	-2.3162062232	-0.3458330895
C	-5.2210515262	-1.5088411873	-1.6417730749
O	-6.038037734	-0.325167386	-1.586901011
C	-6.1397421049	0.4086856377	-0.4745782182
O	-6.6549951493	1.5180035292	-0.5682855451
C	-3.8470487928	-1.1837714502	-2.2425616561
C	-2.7841250168	-0.3851772435	-1.4896831631
C	-2.0682836814	-1.1066131775	-0.3488248811
C	-1.033707589	-0.1731747657	0.2790382276
C	-1.688108781	1.1524915544	0.6640362304
C	-2.4253196751	1.7540128689	-0.5290367076
O	-3.3683795064	0.8247009461	-1.0507188596
C	-3.1871149177	3.0199963299	-0.1933194273
H	-5.3639644364	0.7648034998	4.0634334456
H	-4.4993332801	-3.0222576125	2.2047128349
H	-6.4832062745	2.0774146457	0.9975854015
H	-4.5419472577	-1.0946665648	5.122694489
H	-6.2527528889	-2.8615928698	-0.3458782885
H	-4.5115599048	-3.074485268	-0.3378056052
H	-5.7183292052	-2.1014445848	-2.4149796253
H	-3.3963748581	-2.1386273823	-2.5419803771
H	-4.0647556768	-0.6410862139	-3.1685885249
H	-2.0107643908	-0.1421339534	-2.2428528478
H	-2.7902699972	-1.4090868398	0.4148072219
H	-1.5877645919	-2.0141710012	-0.7339935046
H	-0.5702968744	-0.6449088873	1.1518564596
H	-0.2269218866	0.0156327922	-0.4427719587
H	-2.4124943744	0.9911479754	1.4719674735
H	-0.9400352192	1.8644967557	1.030903464
H	-1.6872853645	1.9708618765	-1.3223682758
H	-2.4988742266	3.800144242	0.1448652564
H	-3.916587253	2.8340486892	0.5994483356
H	-3.7253666914	3.3835543077	-1.0719073341

Table S32. Sampled and DFT geometry optimized conformers related to calc_**1d**, with energy values (Hartree) and related % contribution on the final Boltzmann distribution for the three employed functional/basis set combinations.

Conformer	MPW1PW91/6-31g(d,p)		MPW1PW91/6-311+g(d,p)		B97-2/cc-pVTZ	
	Energy (Hartree)	% contribution on the Boltzmann distribution	Energy (Hartree)	% contribution on the Boltzmann distribution	Energy (Hartree)	% contribution on the Boltzmann distribution
calc_**1d** conf_1	-997.7145519	69.45%	-997.9497948	79.39%	-997.9088768	81.25%
calc_**1d** conf_2	-997.7129280	12.44%	-997.9478343	9.95%	-997.9065739	7.09%
calc_**1d** conf_3	-997.7129263	12.41%	-997.9475148	7.10%	-997.9066714	7.86%
calc_**1d** conf_4	-997.7111995	1.99%	-997.9458455	1.21%	-997.9044298	0.73%
calc_**1d** conf_5	-997.7111538	1.90%	-997.9459094	1.30%	-997.9053266	1.89%
calc_**1d** conf_6	-997.7100113	0.57%	-997.9439393	0.16%	-997.9026267	0.11%
calc_**1d** conf_7	-997.7096731	0.40%	-997.9447707	0.39%	-997.9042392	0.60%
calc_**1d** conf_8	-997.7092679	0.26%	-997.9439922	0.17%	-997.9027621	0.13%
calc_**1d** conf_9	-997.7085747	0.12%	-997.9430913	0.07%	-997.9020797	0.06%
calc_**1d** conf_10	-997.7083106	0.09%	-997.9431711	0.07%	-997.9022704	0.07%
calc_**1d** conf_11	-997.7080874	0.07%	-997.9423688	0.03%	-997.9018509	0.05%
calc_**1d** conf_12	-997.7077698	0.05%	-997.9428325	0.05%	-997.9017539	0.04%
calc_**1d** conf_13	-997.7075785	0.04%	-997.9414658	0.01%	-997.8999649	0.01%
calc_**1d** conf_14	-997.7075210	0.04%	-997.9409407	0.01%	-997.8992183	0.00%
calc_**1d** conf_15	-997.7074126	0.04%	-997.9424322	0.03%	-997.9016501	0.04%
calc_**1d** conf_16	-997.7073787	0.03%	-997.9420120	0.02%	-997.9011330	0.02%
calc_**1d** conf_17	-997.7072016	0.03%	-997.9414065	0.01%	-997.8996308	0.00%
calc_**1d** conf_18	-997.7069172	0.02%	-997.9418101	0.02%	-997.9008934	0.02%
calc_**1d** conf_19	-997.7069151	0.02%	-997.9415833	0.01%	-997.9003288	0.01%
calc_**1d** conf_20	-997.7067265	0.02%	-997.9411055	0.01%	-997.9009285	0.02%
calc_**1d** conf_21	-997.7031610	0.00%	-997.9385785	0.00%	-997.8966992	0.00%

Table S33. Cartesian coordinates of the optimized geometries for the conformers related to calc_**1d**. The related energies and % contribution on the final Boltzmann distribution for the three employed functional/basis set combinations are reported in Table S32.

calc_**1d** conf_1

C	-7.7998868851	-0.9073248861	1.3262749071
C	-7.6726863531	0.4229759537	1.7061205275
C	-6.4127789054	1.0016176843	1.9229288898
C	-5.2794391032	0.2357601728	1.7407686565
C	-5.3794200913	-1.109171306	1.3362482676
C	-6.6573762969	-1.6898083263	1.1513081671
O	-6.812501915	-2.9677180606	0.8125022376
O	-8.7460771385	1.2227587777	1.8910335204
C	-3.8964532542	0.7688724944	1.9885468804
C	-2.9029960377	0.0924496384	1.061853545
O	-2.9865352586	-1.3433661457	1.2025930671
C	-4.1895045575	-1.9317680073	1.1487018077
O	-4.2321562616	-3.1438063333	0.9616009761
C	-1.4578954543	0.4638403555	1.3355628968
C	-0.4875610211	-0.092107393	0.3043259693
C	0.9707618847	0.2047617254	0.6402826841
C	1.8797738735	-0.273855046	-0.4909911646
C	1.3975330665	0.2805642207	-1.83073566
C	-0.0867103095	-0.0131008416	-2.0329784102
O	-0.8407363087	0.4936317909	-0.9394701404
C	-0.6553073549	0.6145267334	-3.2894761093
H	-8.7718071611	-1.3656808767	1.17277511
H	-6.3558173438	2.0395166625	2.2299419993
H	-5.9032778829	-3.3647752422	0.7967820183
H	-9.551243994	0.7199119795	1.7107973709
H	-3.8633466722	1.8514435715	1.8344918136
H	-3.6063862316	0.5809991902	3.0314088198
H	-3.1451255038	0.3306884454	0.0203860349
H	-1.3745371902	1.5567801801	1.3373868437
H	-1.1794104223	0.1063461797	2.3332359128
H	-0.6234160868	-1.1837731186	0.2369410764
H	1.0848762654	1.2876790254	0.7792671023
H	1.2403172282	-0.2780455409	1.5865415165
H	2.9180114344	0.0187114717	-0.3021591004
H	1.8638046485	-1.3713934409	-0.5260153792
H	1.5408209723	1.3684027553	-1.8587068601
H	1.9753578408	-0.1456169362	-2.658759963
H	-0.2259732037	-1.107453093	-2.0752275518
H	-0.5380030233	1.7018254761	-3.2571535587
H	-1.7202053823	0.3868290375	-3.3823709104
H	-0.1396846099	0.2358710722	-4.1765638105

calc_**1d** conf_2

C	-7.131285893	-0.5450466879	2.6435951439
C	-7.4836031782	0.3966120257	1.6851355258
C	-6.7468239365	0.5401749139	0.4989627546
C	-5.6462000732	-0.2648334117	0.2890131093
C	-5.2550518343	-1.2129931395	1.2534517715
C	-6.0214632982	-1.3659533846	2.4337450164
O	-5.720249746	-2.2706256121	3.3622291793

O	-8.5487234677	1.2138599185	1.8384073247
C	-4.8398776382	-0.2242472125	-0.9768892822
C	-3.3809015077	-0.5574678912	-0.7031756257
O	-3.2765790191	-1.8135954551	0.0062825916
C	-4.0787921945	-2.0528574119	1.0527785851
O	-3.7882915464	-2.9846254849	1.7978323073
C	-2.6247310418	0.5282310196	0.0546567163
C	-1.1393046586	0.2309091158	0.190748399
C	-0.3961616123	1.2751509634	1.0184533703
C	1.103003748	0.9794528997	1.0157835642
C	1.6083546968	0.8109548284	-0.4161676765
C	0.7539549211	-0.2020432786	-1.1737213558
O	-0.6153614084	0.1779351361	-1.1277661654
C	1.1279174125	-0.3255106578	-2.6369395468
H	-7.7052721713	-0.6715710892	3.5561985229
H	-7.0621231338	1.2765258547	-0.2311997278
H	-4.943891353	-2.7807776647	3.0118673983
H	-8.9765388489	1.022464386	2.6834005787
H	-5.2443991611	-0.9610158359	-1.6827550894
H	-4.911923119	0.7536395425	-1.4615629003
H	-2.8564946523	-0.7505414736	-1.6396829169
H	-2.7390474429	1.4720234237	-0.4915303592
H	-3.0651514598	0.6718790003	1.0474669512
H	-1.0108896116	-0.7563768723	0.6644057314
H	-0.5829519514	2.2652305289	0.5829957502
H	-0.788489028	1.2872631966	2.0417085452
H	1.6542313756	1.7739381439	1.5297279872
H	1.2887374018	0.0541010873	1.5773876892
H	1.5570642489	1.7707176834	-0.9462295063
H	2.6562628497	0.4901104707	-0.4237693887
H	0.8589231197	-1.1861273602	-0.6846549476
H	2.1663116298	-0.6520082362	-2.7432420261
H	1.0138250788	0.6390800296	-3.1404899454
H	0.4819875042	-1.052036009	-3.1364580537

calc_**1d** conf_3

C	-7.7089809947	-0.5688712357	1.3053582625
C	-7.2318512235	0.6436330171	0.8226026828
C	-5.8558275598	0.9060908044	0.7421672526
C	-4.9602131496	-0.0662248913	1.1389332563
C	-5.4176715325	-1.3112897919	1.6115830098
C	-6.8084850436	-1.5520913569	1.7181444401
O	-7.2951129679	-2.6934506952	2.2012284337
O	-8.0617124631	1.6289977992	0.4132565825
C	-3.4742716932	0.1476879155	1.1234305621
C	-2.7645630537	-1.163357819	0.8435467285
O	-3.1719610956	-2.1639283933	1.8052778201
C	-4.4805297704	-2.3437709394	2.0387771774
O	-4.8270601331	-3.3709377197	2.614166636
C	-1.2457258799	-1.1135381809	0.9280543147
C	-0.5546265584	-0.5726589582	-0.3142475884
C	0.9650315135	-0.6910417621	-0.2240851549
C	1.624939111	-0.05543794	-1.4453480704
C	1.1024999345	1.3645642046	-1.6487148119
C	-0.4233203566	1.377700012	-1.6698467477
O	-0.9410590163	0.7827296035	-0.4847455053
C	-1.0075957812	2.7731036873	-1.7549004182
H	-8.7724090903	-0.7712313411	1.3860951054

```
H  -5.5230230704  1.8690699648   0.3726100803
H  -6.5124028726  -3.2279636938  2.4945659633
H  -8.9765587403  1.3351195373   0.5145667905
H  -3.1734322917  0.8839861899   0.3766582292
H  -3.1438344205  0.5263322722   2.1002544518
H  -3.0657481009  -1.5334070711  -0.1461393517
H  -0.9401993284  -0.5257707635  1.8010711066
H  -0.8958960913  -2.1382039331  1.0911604583
H  -0.8972540265  -1.1489166817  -1.1937060524
H  1.2992202513   -0.1821854191  0.688790498
H  1.2479127807   -1.7455987558  -0.1327017206
H  2.7145512125   -0.0555189235  -1.3377493123
H  1.397888411    -0.6575418895  -2.3355342571
H  1.4419012852   2.0098890464   -0.8285173048
H  1.4877155563   1.7923854052   -2.5811551786
H  -0.7666136778  0.7833554455   -2.5351590174
H  -2.0999253026  2.7341022586   -1.7727153504
H  -0.6651224625  3.2782836978   -2.6623588745
H  -0.6986723074  3.3669072942   -0.889675126
```

calc_**1d** conf_4

```
C  -6.9328357728  0.1651356259   2.1516654372
C  -6.6636609256  0.9060147722   1.0074830418
C  -5.6875976527  0.4966399173   0.0862309955
C  -4.9764758548  -0.6610530978  0.3284701125
C  -5.2150593986  -1.4182431776  1.4909499735
C  -6.2194953561  -1.0087505534  2.4001214765
O  -6.5117583432  -1.7066837817  3.4958077659
O  -7.3262878075  2.0490445413   0.7210742753
C  -3.9603308344  -1.2008862717  -0.6359079798
C  -2.8545587998  -1.9391111595  0.1021136741
O  -3.4135681262  -2.9419953796  0.9822477412
C  -4.4559135361  -2.6325339339  1.7666042591
O  -4.7300167682  -3.4007039488  2.6841907526
C  -1.9027102853  -1.050554336   0.9041319708
C  -0.7973795758  -0.3990835742  0.084948902
C  0.2100544589   0.3362788026   0.9659066599
C  1.2514939355   1.0501800579   0.1076892913
C  0.563218677    1.9193726324   -0.9424713683
C  -0.4592859739  1.1036166386   -1.7283624661
O  -1.3930438938  0.493370436    -0.8444544964
C  -1.2602084668  1.9356993066   -2.7094238392
H  -7.6964747155  0.4688883251   2.8609154735
H  -5.5122981992  1.0963681905   -0.7991762269
H  -5.9439519388  -2.520160947   3.4666240807
H  -7.9675282783  2.2269127637   1.4216312051
H  -4.4551259647  -1.8991864105  -1.3233864224
H  -3.5126291748  -0.4027863276  -1.2301531899
H  -2.2720231821  -2.531520141   -0.608453705
H  -2.4633882689  -0.2741538087  1.4364371343
H  -1.4263926605  -1.6845614102  1.6592823704
H  -0.2589072514  -1.1840207245  -0.4785101352
H  -0.3335807518  1.0660305391   1.5788772758
H  0.6887313949   -0.3733226432  1.6498595599
H  1.9183392481   1.6530273288   0.7327409359
H  1.8817599353   0.3036477154   -0.3944149988
H  0.0403604249   2.7520776337   -0.4551961266
H  1.2963973776   2.3531720892   -1.6318001514
```

```
H  0.0746051696   0.3055713817   -2.2743481989
H  -1.8164651403  2.7143426362   -2.1793796205
H  -1.9749962521  1.3114316592   -3.2520960415
H  -0.599011472   2.4154886334   -3.4364693977
```

calc_**1d** conf_5

```
C  -7.8935127021  -1.4309898852  1.603007208
C  -8.0165800565  -0.0535878202  1.4687541501
C  -6.888056073   0.7687861597   1.3260324942
C  -5.6340857771  0.193899135    1.3067662078
C  -5.4825478488  -1.2015102048  1.4207823191
C  -6.62693863    -2.0171760074  1.5940990244
O  -6.5398925322  -3.3361359906  1.7493634611
O  -9.2177043861  0.5641202374   1.4733426089
C  -4.3755028438  1.0066147909   1.1954880526
C  -3.3032319851  0.215449977    0.465172534
O  -3.0978866304  -1.0554713265  1.1162746548
C  -4.163053762   -1.8196715998  1.4064139712
O  -3.9682885233  -3.0035822369  1.6611170006
C  -1.9672553604  0.9308230451   0.4100796456
C  -0.8965932452  0.2732412411   -0.4585754673
C  -0.1503399622  -0.8804691444  0.2062832056
C  0.9833094719   -1.3688345547  -0.6930395694
C  1.8674082068   -0.1984377131  -1.1159281017
C  1.0186715264   0.9211471924   -1.7103058194
O  0.0029518021   1.3184004917   -0.799732143
C  1.8162469838   2.1655224598   -2.0456322502
H  -8.7610520123  -2.0709571647  1.7295258106
H  -7.0256014645  1.8400683323   1.2356846494
H  -5.5725483043  -3.552877914   1.7750068045
H  -9.9151883454  -0.0967605975  1.5758932798
H  -4.5634702006  1.9457017792   0.6672647323
H  -4.0088103108  1.2664686432   2.1975733318
H  -3.6514503336  -0.0000896339  -0.5543556835
H  -2.146054665   1.9266838907   -0.0100582193
H  -1.5879346487  1.0751095589   1.4280674723
H  -1.3687391311  -0.0984726096  -1.3874866321
H  0.2606901435   -0.5162442968  1.1566763035
H  -0.8463890211  -1.688644153   0.4430464514
H  1.5731709572   -2.1364314747  -0.1813271684
H  0.5588929284   -1.8459012292  -1.5871614906
H  2.4038059099   0.1988706318   -0.2446971331
H  2.6206125319   -0.5175321812  -1.8455508333
H  0.5336770399   0.5399220124   -2.6273657974
H  2.2912657762   2.5643647667   -1.1444954393
H  1.1637611732   2.9387243953   -2.4591805719
H  2.5962443046   1.9378589976   -2.7778230534
```

calc_**1d** conf_6

```
C  -7.3935786474  -0.3422831099  1.979666225
C  -7.3291068686  0.4461239843   0.83721131
C  -6.1741489363  0.4839110282   0.0415487618
C  -5.0862437201  -0.2892030512  0.3936236134
C  -5.133904723   -1.113499875   1.5350476712
C  -6.2934086821  -1.117721236   2.3479478509
O  -6.3752904319  -1.8420836544  3.4621023045
O  -8.3652886836  1.2165884308   0.4382913859
```

C	-3.7991851017	-0.2708508037	-0.3800134615
C	-3.1384577866	-1.6357016555	-0.298382001
O	-2.9518332025	-2.0278550469	1.0801201872
C	-3.9905482248	-1.9300688752	1.9241951406
O	-3.9207178464	-2.5275470897	2.9938895262
C	-1.796972666	-1.791475189	-1.001310729
C	-0.6637195456	-0.8141400758	-0.6980314902
C	-0.2355644745	-0.699262893	0.7624240946
C	0.912158901	0.3021977414	0.8823173068
C	0.5266986249	1.6269017311	0.2262074265
C	0.031498739	1.4000137517	-1.1997246346
O	-1.0324958329	0.456159582	-1.2178718736
C	-0.4843544447	2.6602795143	-1.8643282535
H	-8.2778772926	-0.3630136103	2.6088683812
H	-6.1584128189	1.1220299837	-0.8343934754
H	-5.4872095898	-2.2667702902	3.5849706375
H	-9.098704504	1.1067519495	1.0576832134
H	-3.97085376	-0.0116924637	-1.4282506672
H	-3.1211721765	0.4912505476	0.0165367928
H	-3.8230705511	-2.3747499195	-0.7366037091
H	-1.4389481795	-2.8056978897	-0.7932204312
H	-1.9895604423	-1.7383006704	-2.0787732366
H	0.2101386959	-1.1739235721	-1.2720565681
H	-1.0837613882	-0.3687118368	1.3691144184
H	0.0574755517	-1.6838655579	1.1414012541
H	1.1808935915	0.4558166579	1.9325098918
H	1.8045836569	-0.1031113281	0.3857045047
H	-0.2760815778	2.1073379289	0.8004796276
H	1.3759724284	2.3196772742	0.2141900489
H	0.8651232265	0.9878733638	-1.7962827952
H	0.3077239484	3.4114273742	-1.9314493194
H	-1.3135547522	3.0829459118	-1.2891684538
H	-0.8432405126	2.4412429386	-2.8731904759

calc_**1d** conf_7

C	-7.8797032857	-1.1376980819	1.2951402811
C	-7.6489825732	-0.0125146718	0.5136229965
C	-6.3548057767	0.5026932006	0.3422639827
C	-5.2919298512	-0.1303423889	0.9536565493
C	-5.4953980865	-1.2841653879	1.7334934794
C	-6.8093734428	-1.7762809567	1.9236549642
O	-7.0652755735	-2.8363900837	2.6861711377
O	-8.6496091494	0.6438962319	-0.1135198264
C	-3.882892134	0.3815681887	0.8532823268
C	-2.899057611	-0.7740461171	0.9163840691
O	-3.1306043279	-1.5648853787	2.1015056876
C	-4.3803880814	-1.957799285	2.3890103726
O	-4.5271851564	-2.8608104031	3.2060020828
C	-1.4381387958	-0.3655154577	0.9948840663
C	-0.9003311422	0.386945149	-0.2168821543
C	-0.9445267235	-0.4079969401	-1.520796511
C	-0.2393342159	0.3594968019	-2.638578238
C	1.1558464215	0.7889033015	-2.1907985973
C	1.0839876903	1.5254868849	-0.8564756777
O	0.4348504101	0.7211085952	0.1182845234
C	2.4448991749	1.8821607168	-0.2937173681
H	-8.8799882444	-1.5317823527	1.4450825828
H	-6.2176168688	1.3902928703	-0.2645323538

H	-6.1994553924	-3.1076150454	3.0879057844
H	-9.4875692332	0.2021415849	0.0771500347
H	-3.7437787066	0.9412764327	-0.0760625504
H	-3.6751823723	1.0737477167	1.6805176306
H	-3.0688517292	-1.4357673305	0.0576702807
H	-1.2919087814	0.2584675873	1.8828223076
H	-0.8345490688	-1.2673406849	1.1396440601
H	-1.4679220596	1.3244228678	-0.3606789195
H	-0.4473018277	-1.3722187592	-1.3553046693
H	-1.9818534894	-0.6205592599	-1.8045687242
H	-0.1862838894	-0.2491759117	-3.5471996779
H	-0.8285705094	1.2511872486	-2.8925837621
H	1.7967642383	-0.092785177	-2.0645629224
H	1.6266881099	1.4302388406	-2.944451378
H	0.4930285478	2.4485203584	-0.9976460826
H	3.0388529777	0.9770498713	-0.1368498507
H	2.3392907542	2.3923101741	0.6668902544
H	2.9861597748	2.5387750508	-0.9808301912

calc_**1d** conf_8

C	-6.9813488606	-0.1744873646	2.4480072324
C	-7.4584101115	0.1947107702	1.1967899422
C	-6.7865647034	-0.1769884144	0.0212591967
C	-5.6245070906	-0.9138120731	0.1155332321
C	-5.1088465035	-1.2850432223	1.3721438836
C	-5.8087741402	-0.9256280463	2.548848333
O	-5.3869308493	-1.2783946105	3.7606778694
O	-8.5874717094	0.9199619063	1.0433991318
C	-4.8749725004	-1.4044813786	-1.0896336519
C	-3.3793261138	-1.474039102	-0.807606156
O	-3.1364408114	-2.2605233404	0.3795782927
C	-3.8677337644	-2.0407400088	1.4833681084
O	-3.4525551193	-2.4997290512	2.5433704335
C	-2.7114900362	-0.1080672984	-0.7136994892
C	-1.1911376158	-0.131889036	-0.5656622275
C	-0.6879541644	-0.3626171472	0.8567374232
C	0.8323250258	-0.2248185675	0.91368975
C	1.2705009978	1.0974063425	0.2894596015
C	0.6647445299	1.255745402	-1.1019278036
O	-0.7491132738	1.1275830638	-1.0516264077
C	0.9585532111	2.601719953	-1.7336990187
H	-7.5033804089	0.0979248393	3.3598542062
H	-7.1973179157	0.1205492757	-0.9365534265
H	-4.5785741412	-1.8370219047	3.6243608492
H	-8.9583746379	1.1188764626	1.913245137
H	-5.2308448397	-2.4100255377	-1.347861357
H	-5.0604045469	-0.7661224683	-1.9579143148
H	-2.8896998751	-2.0554615582	-1.593473288
H	-2.9275863749	0.4237710571	-1.6470644627
H	-3.1576154761	0.4816179606	0.0947530174
H	-0.7787222767	-0.9236711695	-1.2193551803
H	-1.1518432871	0.3896888553	1.507983308
H	-1.0052033934	-1.3454587264	1.2144190266
H	1.1839116791	-0.3020295457	1.947782391
H	1.2933708284	-1.0571181211	0.3643271324
H	0.931519021	1.9354981653	0.9118831718
H	2.3630468138	1.1593582173	0.2257531072
H	1.0641272812	0.4536500208	-1.7490639667

H	2.0369251934	2.7508670631	-1.8395949309
H	0.5555299221	3.4066662388	-1.1120926399
H	0.4975900381	2.6695720991	-2.7223954559

calc_**1d** conf_9

C	-7.5031310275	-0.2865922142	1.3610731373
C	-6.9015284313	0.9291606125	1.0600523491
C	-5.5055122021	1.0590913652	1.0129343019
C	-4.7167484419	-0.0469120168	1.2582419698
C	-5.301044539	-1.2942982874	1.5444355248
C	-6.7105741984	-1.405958079	1.619509792
O	-7.314521317	-2.5507986155	1.9303149698
O	-7.6232550119	2.0430674892	0.8038950087
C	-3.2153717054	0.0251517732	1.2656542654
C	-2.6243431683	-1.2989375952	0.8211438172
O	-3.1552834382	-2.3842917387	1.6180333202
C	-4.4775763945	-2.4675034574	1.8130638663
O	-4.934969803	-3.5285973368	2.2271417902
C	-1.1137870178	-1.4483604429	0.9499550156
C	-0.2734384724	-0.484204639	0.104751589
C	0.1059098591	0.8233147548	0.7999978304
C	0.894207977	1.7159764837	-0.1572477093
C	0.14109522	1.8779803407	-1.4763100076
C	-0.2369113231	0.5159988786	-2.0515448684
O	-0.9807083524	-0.2351584266	-1.0996671491
C	-1.0880651469	0.6060776062	-3.3016565388
H	-8.5823552669	-0.390454939	1.4149461126
H	-5.0738946736	2.0269236125	0.7854154108
H	-6.5920907105	-3.1998792186	2.1351628694
H	-8.5641164241	1.8305151975	0.8604887869
H	-2.8686013969	0.8086621043	0.5888097379
H	-2.8599634823	0.2710087367	2.2756025232
H	-2.916127628	-1.4840109019	-0.2171297531
H	-0.8333599759	-1.3488735605	2.0046151043
H	-0.8850620731	-2.4786744435	0.6620799531
H	0.670841418	-0.9975721276	-0.1479126104
H	-0.7969597737	1.3446532681	1.1359401789
H	0.6978941023	0.5971210204	1.6942719801
H	1.0920593635	2.6915375096	0.2988514612
H	1.8730891983	1.2579225945	-0.3540666144
H	-0.7798021896	2.4535164331	-1.3158689382
H	0.7458542235	2.4306958811	-2.2040654997
H	0.6910472714	-0.0381581467	-2.2778677653
H	-0.5523488697	1.1347331816	-4.0950549781
H	-2.0163740016	1.1452823569	-3.0909715452
H	-1.346172176	-0.393155013	-3.6610186889

calc_**1d** conf_10

C	-7.619897697	-0.5435134271	1.1217729405
C	-7.1991975618	0.7798600531	1.1656773096
C	-5.8522585834	1.1102146986	1.3810079039
C	-4.9281944046	0.0971116698	1.5370063455
C	-5.324676244	-1.2515057757	1.4748943417
C	-6.6907912575	-1.5716410085	1.2907467974
O	-7.1266630226	-2.8291357778	1.2721055432
O	-8.0583714491	1.8117070603	1.0105488905
C	-3.4739518498	0.3590020465	1.8100348369

C	-2.6224410062	-0.7301916792	1.1800757972
O	-3.0516582898	-2.0185878564	1.6831613858
C	-4.3549236262	-2.3276775431	1.6474497901
O	-4.6752163289	-3.5048917345	1.7790757483
C	-1.1292482542	-0.6510800186	1.4812669982
C	-0.2907980716	0.0904768852	0.4436364798
C	-0.5816804758	1.580146419	0.2738568701
C	0.3223154877	2.1619777703	-0.8122019101
C	0.1897363517	1.3433707415	-2.0950101119
C	0.4073492936	-0.1414725043	-1.8152021932
O	-0.4661019776	-0.5900105247	-0.7867099992
C	0.1539968696	-1.0224061208	-3.021441648
H	-8.6629847793	-0.8048169399	0.9731443437
H	-5.5663402606	2.1549326557	1.4220323984
H	-6.3393774616	-3.3997978685	1.4707732614
H	-8.948520633	1.4639112392	0.8680805995
H	-3.1793648406	1.3413605899	1.4338932719
H	-3.2945669457	0.3631496184	2.8940139074
H	-2.7716133051	-0.7455962452	0.0948640517
H	-0.9607009881	-0.2117054853	2.471300362
H	-0.7602514101	-1.6798306691	1.5170617343
H	0.7646794282	-0.0086503401	0.7545160603
H	-1.6284871171	1.713875019	-0.0241544413
H	-0.4390197057	2.101948775	1.2273347243
H	0.0781769744	3.2133134663	-0.9968238952
H	1.3650901314	2.1367989809	-0.4678267356
H	-0.8152986032	1.4727291001	-2.5167963335
H	0.9058228079	1.6843656825	-2.8511671357
H	1.4471748927	-0.2834958648	-1.4706259234
H	0.8321735414	-0.7613591967	-3.8388287023
H	-0.8752159392	-0.9003533501	-3.3714441051
H	0.3052963107	-2.0735325413	-2.764099559

calc_**1d** conf_11

C	-7.5241640742	-0.4241226331	1.836660622
C	-7.5563984077	0.0257246895	0.5226419574
C	-6.4088905167	0.0010886399	-0.2845281911
C	-5.2306340155	-0.4944232372	0.2362872699
C	-5.1757776224	-0.9764572695	1.5572892605
C	-6.3320340585	-0.9158037053	2.3716097728
O	-6.3251823937	-1.3153540419	3.6411568978
O	-8.6840868019	0.5127967267	-0.0419145576
C	-3.945368749	-0.5141726405	-0.5406044926
C	-3.1100378507	-1.7104395148	-0.1197991056
O	-2.8903627418	-1.6973321668	1.3050928564
C	-3.9349395456	-1.5037130092	2.1180163287
O	-3.8013301112	-1.775060831	3.3079135353
C	-1.7434207772	-1.829724036	-0.7866038664
C	-0.9647297983	-0.5396507035	-1.0111901007
C	0.4232593348	-0.8098446311	-1.5898966141
C	1.214166013	0.490997176	-1.711187942
C	1.1999699806	1.2401194856	-0.3803107233
C	-0.2295022651	1.4080487894	0.126216563
O	-0.874958991	0.1443538513	0.222746621
C	-0.3091720142	2.045046536	1.4986251717
H	-8.4041460851	-0.3920335652	2.4714845458
H	-6.4711449063	0.3741049433	-1.3003259405
H	-5.3871878692	-1.568755753	3.8470712771

```
H  -9.4014528146  0.4791435942   0.6044804931
H  -4.1382080977  -0.5536487877  -1.6171721997
H  -3.3779966991  0.4007760661   -0.3352156271
H  -3.6764038955  -2.6266287472  -0.3342319108
H  -1.1445285046  -2.5154523934  -0.1781562162
H  -1.8825669485  -2.3079327648  -1.7631505162
H  -1.5153998678  0.0971358095   -1.7289215131
H  0.9474202065   -1.5023320741  -0.9193877044
H  0.3313162451   -1.3041314533  -2.5639807672
H  2.2413251601   0.2883670637   -2.0320790934
H  0.7613335064   1.1216957888   -2.4883762473
H  1.7681700192   0.6785508448   0.3718895465
H  1.6741354963   2.222971677    -0.4809898715
H  -0.7855179243  2.0285903772   -0.6002147647
H  0.2209266515   1.4303967064   2.2315149249
H  -1.3498181688  2.1339837134   1.8200168686
H  0.1403399026   3.0421214797   1.4875234532
```

calc_**1d** conf_12

```
C  -7.1946979261  0.0472167203   1.5974549378
C  -6.9963524361  0.2802058763   0.2426717328
C  -5.923471008   -0.3053323488  -0.4481107606
C  -5.0451599686  -1.1170410554  0.2391960617
C  -5.2101612508  -1.3517472732  1.6170852948
C  -6.3115901329  -0.7788036768  2.2961058855
O  -6.539423697   -0.997543306   3.5887386346
O  -7.8214679678  1.0698294512   -0.479395122
C  -3.9082013715  -1.8343487035  -0.42859218
C  -2.725447107   -1.9684838038  0.5213609202
O  -3.1473089839  -2.6010442234  1.7498557626
C  -4.2698320698  -2.1864663575  2.3574289624
O  -4.4598489289  -2.5424611899  3.5158285083
C  -2.0135493137  -0.6645490411  0.8678493888
C  -1.3133890047  0.0326367483   -0.293924537
C  -0.2109813192  -0.7941695025  -0.9533691468
C  0.5389900213   0.0430110349   -1.9887557767
C  1.0026477002   1.3606486576   -1.3726981234
C  -0.166199167   2.0744607218   -0.6997654821
O  -0.7798941503  1.223277961    0.2580353719
C  0.2433157841   3.3348706985   0.0349848707
H  -8.030526264   0.484682583    2.1344107266
H  -5.8118233666  -0.1119905758  -1.5087105405
H  -5.8441149192  -1.6379541867  3.8904312957
H  -8.5161755266  1.4143666678   0.0971199825
H  -4.2378362694  -2.8401494677  -0.7190569257
H  -3.6029240514  -1.3251054681  -1.3467976691
H  -2.0091620136  -2.6830202225  0.1104155524
H  -2.7265685437  0.0419736124   1.3047687337
H  -1.2670079401  -0.8822299552  1.6386098571
H  -2.054312574   0.3078551737   -1.0665930844
H  0.4816598126   -1.1331344244  -0.1723777788
H  -0.6329114206  -1.6887814112  -1.4262280984
H  1.3883676755   -0.5179853323  -2.3923206713
H  -0.1279439122  0.2538358766   -2.8359918788
H  1.7728484551   1.1693828069   -0.6147777763
H  1.4476048727   2.0117679959   -2.1336407931
H  -0.9137648215  2.3280331628   -1.4728652679
H  -0.6241926101  3.7967898146   0.5126508945
```

H	0.6883235336	4.056734218	-0.6557579874
H	0.976482181	3.0987617441	0.8117262255

calc_**1d** conf_13

C	-6.7474625594	0.2386425027	1.9354200056
C	-7.343511065	-0.2086617281	0.7634025104
C	-6.6466223182	-1.0216501281	-0.1438443905
C	-5.3404200142	-1.3719980648	0.1320207675
C	-4.7062356773	-0.9146701217	1.3023160099
C	-5.4283866739	-0.1190237011	2.2223106464
O	-4.8918374931	0.3060067482	3.3638660538
O	-8.6173788679	0.1067435855	0.4385117744
C	-4.5412134818	-2.2810034831	-0.7592550667
C	-3.0646379266	-1.9106149968	-0.7038135044
O	-2.6082394177	-1.91036136	0.6628874285
C	-3.3223781218	-1.2716883903	1.5995816592
O	-2.7911490213	-1.0520691213	2.6828068331
C	-2.7709342418	-0.5895581911	-1.4179085058
C	-1.4056168015	0.0447653202	-1.1634324595
C	-0.220146769	-0.9165799899	-1.219770626
C	1.069157665	-0.1790097521	-0.8630487002
C	0.9000426434	0.5402409173	0.4733790441
C	-0.3356298431	1.4352245762	0.4468971836
O	-1.4909519085	0.6885647374	0.0939814585
C	-0.6345799821	2.0817991253	1.7844742087
H	-7.284989979	0.8553317975	2.6489193084
H	-7.1508997975	-1.3611565057	-1.0412446853
H	-3.9851267582	-0.0948534262	3.4104544575
H	-8.9917246829	0.6664981657	1.1314170751
H	-4.6620858598	-3.3184934727	-0.4227903113
H	-4.900000726	-2.234311149	-1.7918151091
H	-2.4698464626	-2.7063271201	-1.1579803351
H	-2.8796739962	-0.7833344689	-2.4919030279
H	-3.5257835291	0.1572347447	-1.1511846832
H	-1.2531961651	0.817757409	-1.9391934114
H	-0.3833712815	-1.7251430485	-0.4999542008
H	-0.1525197892	-1.3623496097	-2.2197993309
H	1.9126393968	-0.8763597685	-0.8251222053
H	1.3016188536	0.5551262267	-1.6469553736
H	0.7776609446	-0.193860404	1.27918884
H	1.7848492522	1.143260304	0.7072760648
H	-0.1814695898	2.2185555932	-0.3181087682
H	0.2121124669	2.688466199	2.1185346248
H	-0.8359275488	1.3101450405	2.5324933211
H	-1.5161328725	2.7237150086	1.70998542

calc_**1d** conf_14

C	-6.17695401	0.8831143423	0.9265396282
C	-6.4212820798	0.3134830065	-0.3173614868
C	-5.773691895	-0.8641825244	-0.7165433163
C	-4.8663114098	-1.4571935785	0.1411027723
C	-4.5838912249	-0.8900452939	1.3938938521
C	-5.2687232073	0.2799394088	1.7986170904
O	-5.0729471566	0.835758136	2.9936319402
O	-7.2941534741	0.8553424849	-1.1968789767
C	-4.1999813642	-2.7642492959	-0.1769579337
C	-2.8337776511	-2.905853387	0.4886978002

O	-2.8792582239	-2.5335194557	1.878739779
C	-3.6080950067	-1.4938422694	2.2969600611
O	-3.4489156335	-1.1112504726	3.4521979518
C	-1.6670219043	-2.2325491108	-0.2353800271
C	-1.7354112853	-0.7379182386	-0.5210039558
C	-0.6546584941	-0.3033012571	-1.5109853142
C	-0.6613740878	1.2152469417	-1.673221972
C	-0.5885515589	1.8907408189	-0.3064406428
C	-1.6689581425	1.3476456288	0.624351077
O	-1.5741289584	-0.070334683	0.7169361537
C	-1.5608375923	1.8886723507	2.0353438568
H	-6.6882054637	1.7842802138	1.2506360132
H	-6.00563083	-1.2913744588	-1.6854410756
H	-4.447803979	0.2315499925	3.4770026728
H	-7.6799798468	1.6518218954	-0.8093086358
H	-4.8467267322	-3.576149696	0.1793846641
H	-4.0950747677	-2.9007340757	-1.25770659
H	-2.5891667936	-3.9705696845	0.5341421993
H	-0.7575606158	-2.4218753426	0.345232848
H	-1.5508913353	-2.7610487152	-1.1900801937
H	-2.7197662159	-0.4760848929	-0.9457166936
H	0.3201503563	-0.6349017781	-1.1306403847
H	-0.8153104577	-0.7982874863	-2.4759739403
H	0.1678504499	1.538289659	-2.31158256
H	-1.5864716583	1.520353719	-2.1809169544
H	0.3900110829	1.7005338062	0.1526751096
H	-0.6975737413	2.977010412	-0.402121316
H	-2.6583773613	1.6023001403	0.2063578998
H	-1.644804564	2.9793356413	2.0334524081
H	-0.5962321586	1.6135176047	2.4715964873
H	-2.3515110073	1.4803294942	2.6667697043

calc_**1d** conf_15

C	-7.7370913546	-0.7446304562	2.0321583009
C	-7.8209819714	0.0492768964	0.8951641857
C	-6.6916160653	0.3106643998	0.1034515995
C	-5.4799881713	-0.2467104538	0.4562686333
C	-5.3718052815	-1.0733038537	1.5909211403
C	-6.5108985316	-1.3013586166	2.3999095561
O	-6.4567899303	-2.0354219889	3.5085487339
O	-8.9821738112	0.6132934322	0.4969486477
C	-4.2123578966	0.0147828591	-0.3070497792
C	-3.324094575	-1.2159682457	-0.25035471
O	-3.0596243566	-1.5666063672	1.125143642
C	-4.0944339293	-1.6648136395	1.9743154776
O	-3.9134220015	-2.2411842353	3.0415263213
C	-1.9737195209	-1.12650084	-0.9429749741
C	-1.0201092298	-0.0342206168	-0.4564594364
C	-1.1164242617	1.2835300271	-1.2253163356
C	-0.0423794274	2.2605004242	-0.7487911799
C	1.3327652212	1.59825116	-0.7971583448
C	1.3051607164	0.2577796839	-0.0682199209
O	0.2850050243	-0.5745063737	-0.6012027516
C	2.6042653386	-0.5141048289	-0.180860676
H	-8.6029362469	-0.9364210419	2.6581306607
H	-6.7950536048	0.9469040242	-0.767903001
H	-5.5049021135	-2.285747491	3.6323731851
H	-9.684114749	0.3642745779	1.1125645263

H	-4.4249942583	0.2745670157	-1.3482641527
H	-3.689985433	0.8680101245	0.1427281216
H	-3.8754349955	-2.0565881458	-0.6917501011
H	-1.4659500913	-2.0821376288	-0.783677231
H	-2.1439739721	-1.0318732291	-2.0219976371
H	-1.2036743313	0.1547329878	0.6150414313
H	-0.973058891	1.0651084989	-2.2914879412
H	-2.112154023	1.7265694759	-1.1180428966
H	-0.0559375636	3.1724854498	-1.355045306
H	-0.2628748903	2.5663095914	0.2829376041
H	1.6241690734	1.4189208668	-1.83989237
H	2.0949165135	2.2479773901	-0.3521078557
H	1.0819449054	0.443268208	0.997604426
H	3.4310782184	0.0558324403	0.2526786898
H	2.8350339022	-0.7175848298	-1.2307696297
H	2.5256165663	-1.4693566514	0.3439113472

calc_**1d** conf_16

C	-7.5042482974	-0.2791492712	1.366160629
C	-6.8996834687	0.9348030906	1.0693033896
C	-5.5021376311	1.0598589732	1.0202108312
C	-4.7119068927	-0.0489702035	1.2599131847
C	-5.3001857456	-1.2922854104	1.5422450981
C	-6.7133034191	-1.3983973815	1.6187305084
O	-7.3168881105	-2.5442480029	1.9264199061
O	-7.7146079498	1.988131319	0.8348275325
C	-3.2104905812	0.0220889328	1.2639109254
C	-2.62359767	-1.3022022153	0.8144027328
O	-3.1549879539	-2.3866645465	1.6100475505
C	-4.4780963137	-2.4679569815	1.8073967194
O	-4.9351543378	-3.5284954324	2.2208181007
C	-1.1130585387	-1.4554865465	0.939131527
C	-0.271431217	-0.4900671408	0.0968073481
C	0.1157625474	0.8118551514	0.7981574214
C	0.9067356708	1.7054831534	-0.1560320072
C	0.1527934661	1.8776558712	-1.473379065
C	-0.2344163843	0.5205382238	-2.0541379902
O	-0.9810506297	-0.2300565712	-1.1041171646
C	-1.0867973583	0.6210806245	-3.3025867961
H	-8.5828183851	-0.3577407035	1.4151439994
H	-5.0461519214	2.021130241	0.7966645995
H	-6.5977254533	-3.1955369267	2.1294543942
H	-7.1772215917	2.7689938023	0.6498800615
H	-2.8626708292	0.8061379146	0.5878454784
H	-2.8517170167	0.2644256501	2.2735549425
H	-2.9185153817	-1.4831284454	-0.2237806429
H	-0.8303611062	-1.3616481495	1.9936932623
H	-0.8878156292	-2.4851847897	0.6464713075
H	0.6697349453	-1.0059543003	-0.1619250825
H	-0.78417795	1.3355720441	1.138588625
H	0.7082571293	0.5783542948	1.6901661956
H	1.1104459949	2.6778153107	0.3044174959
H	1.8829679526	1.2435356734	-0.3565460273
H	-0.76459312	2.4579018388	-1.3091150779
H	0.7597172953	2.4301722285	-2.1994649288
H	0.6897593095	-0.0380781138	-2.2845268721
H	-0.5490379008	1.1495632857	-4.0946963392
H	-2.0115430141	1.1651467929	-3.0884843659

H -1.351782512   -0.3749932843 -3.6655714071

calc_**1d** conf_17

C -7.0042349774 0.0348018857   1.9373761032
C -6.6490201414 0.8311172286   0.8560793439
C -5.5551001835 0.5051236607   0.0390037366
C -4.8135856256 -0.6230129737 0.3263458609
C -5.1398680845 -1.4356276   1.4287017057
C -6.2626955677 -1.1125615591 2.227209364
O -6.6417697734 -1.8674194611 3.2556817619
O -7.3358457556 1.9491418372   0.5312848617
C -3.6654786707 -1.0846040999 -0.5217589812
C -2.6075824325 -1.7717030745 0.3251876415
O -3.2082521454 -2.8452030965 1.0885088049
C -4.351858301   -2.6228083707 1.7500242557
O -4.6955207423 -3.4409521381 2.598448173
C -1.8202214965 -0.8959666163 1.3025541704
C -0.9529221216 0.1966980546   0.6618494391
C -1.6182765718 1.5695226062   0.575736037
C -0.6951626097 2.5592719678   -0.1333068287
C -0.219333697   1.9833057996   -1.4655460016
C 0.3799334518   0.5940113802   -1.2671599194
O -0.5546697911 -0.2614654029 -0.6215067898
C 0.771914752   -0.0824941369 -2.5649491469
H -7.8587548065 0.2723250517   2.5633341215
H -5.3220259741 1.1404300959   -0.8077930549
H -6.0337132302 -2.6524230218 3.2626228574
H -8.0633266743 2.0665417903   1.1562872465
H -4.0353359846 -1.8087392683 -1.2594370443
H -3.2086296352 -0.2679366668 -1.082926918
H -1.9002562799 -2.28017716   -0.3284406489
H -2.504908139   -0.4333760022 2.0218749946
H -1.1841505126 -1.5823082114 1.8693457279
H -0.0467757611 0.3160513415   1.2811870059
H -2.5703427966 1.4919834295   0.0390118825
H -1.8544509203 1.9167278119   1.5880166783
H -1.2003630175 3.5189820384   -0.2833712131
H 0.1767750098   2.7603931513   0.5040698234
H -1.0639192897 1.8997669322   -2.1618772201
H 0.5219215534   2.641872868   -1.9322434773
H 1.2687959881   0.6852457142   -0.61813655
H 1.5280918273   0.5057169313   -3.0924509113
H -0.1003645412 -0.1891502444 -3.2167076165
H 1.1782836691   -1.0781024721 -2.3701292756

calc_**1d** conf_18

C -7.6470071758 -0.8324414368 2.2770009992
C -7.8074566252 0.1778480827   1.336913968
C -6.7528294742 0.5641752778   0.4955733897
C -5.5380540115 -0.0825341029 0.5966454462
C -5.3547211763 -1.124027   1.5268741947
C -6.4178454224 -1.483315118   2.391305673
O -6.2888200844 -2.4290159183 3.3188046403
O -8.9747648619 0.8408065335   1.1869455235
C -4.3505539574 0.302393752   -0.239819626
C -3.4774632315 -0.9167014245 -0.4936275619
O -3.115942336   -1.5582469041 0.7469299934

C	-4.0750938227	-1.8117298128	1.6514275407
O	-3.8196441365	-2.6034341655	2.5525794475
C	-2.1882967105	-0.6647863368	-1.27087124
C	-0.9541438436	-0.2316206751	-0.4801830247
C	-1.0668470565	1.0802274425	0.2910479027
C	0.2880300266	1.4604976278	0.8890473771
C	1.375182661	1.4375089001	-0.1814471674
C	1.3592476011	0.1039615027	-0.9236169015
O	0.0688135908	-0.1429064566	-1.4627043042
C	2.3359044683	0.049854978	-2.0809697979
H	-8.4533240859	-1.1256030436	2.9419431396
H	-6.9141644141	1.3651071656	-0.216651233
H	-5.3430964139	-2.7262164979	3.2873450211
H	-9.622277851	0.4904108812	1.81280318
H	-4.6725759833	0.714497825	-1.200969284
H	-3.7784277114	1.0870130458	0.2671006323
H	-4.0826478682	-1.6397921523	-1.0555185524
H	-1.9048743748	-1.5824638578	-1.7944832481
H	-2.389291645	0.083550758	-2.0461450938
H	-0.6976121456	-1.0300698766	0.233496388
H	-1.4101450003	1.8690249399	-0.3911867775
H	-1.8070674617	0.9782190475	1.0907209233
H	0.2325635049	2.4446056041	1.3662139092
H	0.5432960797	0.7425882686	1.6798836931
H	1.2033302311	2.2405050895	-0.9097236185
H	2.3630982593	1.6050777427	0.2626375652
H	1.5984364158	-0.6996168731	-0.2045492308
H	2.104147764	0.8328187743	-2.8090769471
H	2.2752042788	-0.916954912	-2.586898916
H	3.3607340001	0.1957833254	-1.7277980232

calc_**1d** conf_19

C	-7.436762891	-0.3116068365	1.9399609314
C	-7.3411115681	0.3738947727	0.7356305326
C	-6.2360490948	0.2051189528	-0.1134096175
C	-5.2222530919	-0.6519930375	0.2645013654
C	-5.2847403854	-1.3411569796	1.488985046
C	-6.4166452435	-1.1848524175	2.3233388708
O	-6.5457746539	-1.8436056537	3.47241366
O	-8.3005661115	1.2291200027	0.3172338622
C	-4.030165441	-0.933522492	-0.6049760819
C	-2.7953400152	-1.2017557673	0.245879971
O	-3.0785226627	-2.2665657844	1.1847825414
C	-4.2080491648	-2.2350518107	1.9028491521
O	-4.2929998122	-2.9750607379	2.8784745716
C	-2.2509304629	0.0126105604	1.0058297303
C	-1.145488906	0.7817579097	0.2864517929
C	-1.5248644203	1.4391593297	-1.0390899542
C	-0.3068192161	2.1463708187	-1.6320850832
C	0.8671455595	1.1732424919	-1.7255562904
C	1.1158570613	0.4949745299	-0.3808670407
O	-0.0721685512	-0.1244617565	0.0951774792
C	2.1797499515	-0.5821603016	-0.4397851969
H	-8.2956491589	-0.1973382577	2.5938991836
H	-6.2054483278	0.7463722373	-1.0520633886
H	-5.7582353341	-2.444639006	3.537553541
H	-9.0060917623	1.2615752891	0.9766634189
H	-4.2331919698	-1.8185650791	-1.2217900033

H	-3.846302305	-0.102684564	-1.2895759658
H	-1.9929768149	-1.6184668148	-0.3642076441
H	-3.0609857196	0.7005721072	1.272412634
H	-1.817637	-0.3602169684	1.9383864094
H	-0.8130716003	1.5818829055	0.9716118366
H	-1.8650695916	0.6689033866	-1.7416669766
H	-2.353546237	2.140705151	-0.8880105521
H	-0.5427536732	2.564547166	-2.6162395579
H	-0.0303348778	2.9925287368	-0.98862043
H	0.6486797546	0.3968737479	-2.4701076139
H	1.7771001896	1.6894148916	-2.0518642364
H	1.4172601758	1.2676201467	0.3485533527
H	3.137040112	-0.1611081905	-0.7598791578
H	1.8895764328	-1.36370842	-1.1481431391
H	2.311136828	-1.0427242587	0.5423480474

calc_**1d** conf_20

C	-7.7172594296	-1.2931976791	1.2070203994
C	-7.8991523217	0.0784281939	1.3323290756
C	-6.8069465009	0.9592855887	1.3511358592
C	-5.5300142287	0.4501344411	1.2302781168
C	-5.3184166538	-0.9333986549	1.0821955598
C	-6.4270134258	-1.8140549625	1.0943426453
O	-6.2838690697	-3.1337872213	1.0005222887
O	-9.1263010346	0.6325698775	1.4466704238
C	-4.3056785629	1.3182169026	1.2805782136
C	-3.203293315	0.7190889723	0.4221282775
O	-2.9392879206	-0.6377668025	0.8227405104
C	-3.9717579013	-1.4823946766	0.9596397162
O	-3.7340719971	-2.6857490978	0.9892691166
C	-1.9065551906	1.5060490052	0.5115608294
C	-0.6991900299	0.978605119	-0.2658062126
C	-1.0083553485	0.4459247335	-1.6633562133
C	0.2706573972	-0.0762822228	-2.3158652319
C	0.9566519439	-1.0758144389	-1.3870947229
C	1.1552689732	-0.4710251788	0.0002755527
O	-0.0795498726	-0.0123686417	0.5293898425
C	1.7206816536	-1.4540452081	1.0053275459
H	-8.5565151866	-1.9817091935	1.2077159929
H	-6.9888715024	2.0219151264	1.4629056271
H	-5.3065773145	-3.3079222629	0.9906518775
H	-9.7933574296	-0.0660080852	1.4200124549
H	-4.5323577216	2.3323161287	0.9382121381
H	-3.9477792009	1.3982247753	2.3158189434
H	-3.5487484611	0.6846074844	-0.6193840672
H	-2.1322945723	2.5187964694	0.1566868862
H	-1.6064823605	1.5909428139	1.561436962
H	0.0062511709	1.8237018375	-0.3749990815
H	-1.729133956	-0.3745020919	-1.5769099079
H	-1.4661844173	1.2339885777	-2.2738069272
H	0.0481568236	-0.5359901342	-3.2845570689
H	0.9497655587	0.7642274011	-2.5146317993
H	0.3377699989	-1.975895872	-1.2856884561
H	1.9247696696	-1.3869225169	-1.7960381696
H	1.837735872	0.3942983309	-0.0926949593
H	2.6959046418	-1.8249616397	0.676835006
H	1.0425931168	-2.3042092844	1.1210050243
H	1.8388081062	-0.9763159137	1.9811479321

calc_**1d** conf_21

C	-6.5533573767	0.5366852297	1.2263597118
C	-6.662736375	0.1861500608	-0.1135179534
C	-5.8572401112	-0.8183621478	-0.670586424
C	-4.9303786831	-1.4602705115	0.1280124265
C	-4.7916358627	-1.1168569242	1.4835557379
C	-5.6270948238	-0.1172126982	2.0405880283
O	-5.560315886	0.2280962721	3.3234122286
O	-7.5462722158	0.7833053146	-0.9428808207
C	-4.0899323059	-2.594896395	-0.3814980167
C	-2.7679268305	-2.7529059994	0.3662157014
O	-2.947668173	-2.6473098679	1.7949401116
C	-3.8137386389	-1.7837332069	2.3366030663
O	-3.753144198	-1.5999623361	3.5486988792
C	-1.5792762285	-1.8961640313	-0.0692696651
C	-1.6707227179	-0.3717840826	-0.005881669
C	-2.2512102856	0.283821317	-1.2571264991
C	-2.1634994181	1.805466919	-1.1518342153
C	-0.7362727495	2.2323406523	-0.8172699941
C	-0.2214836233	1.4677887642	0.3990903907
O	-0.333013028	0.0671350405	0.1893777086
C	1.2342534653	1.7492039845	0.7124388119
H	-7.1818220302	1.3035540642	1.6681599687
H	-5.9832462133	-1.0746735099	-1.7163361166
H	-4.8867261442	-0.3723976636	3.7387677132
H	-8.0447371153	1.4488005415	-0.4505737539
H	-4.6648772686	-3.5222659356	-0.2635084006
H	-3.8897044473	-2.4870829449	-1.4516833791
H	-2.4428260689	-3.7890258524	0.2419095921
H	-0.7440160515	-2.1810266653	0.5777977304
H	-1.3032322369	-2.2019444422	-1.0863979495
H	-2.2682023787	-0.0639235796	0.8682925701
H	-1.6694686832	-0.0624452637	-2.1214530136
H	-3.2892890207	-0.0263766932	-1.4078874065
H	-2.5003299017	2.273830282	-2.0827711402
H	-2.8441977264	2.1523542465	-0.3628338585
H	-0.0738498021	2.0178016467	-1.6657654665
H	-0.6844418739	3.3105014153	-0.6270482319
H	-0.84200984	1.7399560965	1.2715025066
H	1.5560507787	1.1699123197	1.5813717017
H	1.3842537892	2.811528673	0.9253212371
H	1.8653383011	1.4723879116	-0.1372918485

Table S34. Sampled and DFT geometry optimized conformers related to calc_**2a**, with energy values (Hartree) and related % contribution on the final Boltzmann distribution for the three employed functional/basis set combinations.

**Conformer**	**MPW1PW91/6-31g(d,p)**		**MPW1PW91/6-311+g(d,p)**		**B97-2/cc-pVTZ**	
	**Energy (Hartree)**	**% contribution on the Boltzmann distribution**	**Energy (Hartree)**	**% contribution on the Boltzmann distribution**	**Energy (Hartree)**	**% contribution on the Boltzmann distribution**
calc_**2a** conf_1	-916.6428199	47.75%	-916.8801926	15.17%	-916.8298210	48.81%
calc_**2a** conf_2	-916.6426963	41.89%	-916.8814972	60.41%	-916.8295482	36.56%
calc_**2a** conf_3	-916.6409860	6.85%	-916.8804583	20.10%	-916.8283295	10.06%
calc_**2a** conf_4	-916.6403030	3.32%	-916.8789241	3.96%	-916.8274255	3.86%
calc_**2a** conf_5	-916.6372323	0.13%	-916.8755818	0.11%	-916.8243186	0.14%
calc_**2a** conf_6	-916.6353451	0.02%	-916.8738058	0.02%	-916.8227599	0.03%
calc_**2a** conf_7	-916.6353118	0.02%	-916.8757050	0.13%	-916.8244998	0.17%
calc_**2a** conf_8	-916.6350563	0.01%	-916.8739557	0.02%	-916.8235548	0.06%
calc_**2a** conf_9	-916.6342671	0.01%	-916.8746861	0.04%	-916.8247115	0.22%
calc_**2a** conf_10	-916.6339973	0.00%	-916.8726469	0.01%	-916.8225701	0.02%
calc_**2a** conf_11	-916.6338765	0.00%	-916.8728533	0.01%	-916.8217229	0.01%
calc_**2a** conf_12	-916.6332930	0.00%	-916.8727808	0.01%	-916.8218994	0.01%
calc_**2a** conf_13	-916.6326067	0.00%	-916.8712038	0.00%	-916.8207010	0.00%
calc_**2a** conf_14	-916.6322320	0.00%	-916.8726368	0.01%	-916.8224439	0.02%
calc_**2a** conf_15	-916.6316901	0.00%	-916.8710877	0.00%	-916.8211578	0.01%
calc_**2a** conf_16	-916.6309166	0.00%	-916.8688316	0.00%	-916.8178010	0.00%
calc_**2a** conf_17	-916.6303572	0.00%	-916.8710994	0.00%	-916.8211567	0.01%

Table S35. Cartesian coordinates of the optimized geometries for the conformers related to calc_**2a**. The related energies and % contribution on the final Boltzmann distribution for the three employed functional/basis set combinations are reported in Table S34.

calc_**2a** conf_1

```
C  -9.0252381199  -1.7881653883  -0.8340015369
C  -8.9238618601  -0.291809802   -0.4272179486
C  -7.5642500875  -0.1647885362  0.2940965892
N  -6.9502011441  -1.5103604141  0.2626743554
C  -7.577111816   -2.2371786111  -0.8258827118
C  -5.5041333845  -1.6361879407  -0.0200156221
C  -5.3544704201  -1.7150888947  -1.5686017551
C  -6.7950979     -1.8294093483  -2.0807155869
O  -9.6564036524  -1.9205144199  -2.0943029598
O  -8.9593777057  0.5267787961   -1.5703677302
C  -4.5748083355  -0.6123064358  0.6263043268
C  -7.7016584877  0.2890026453   1.7497766721
O  -7.0579357614  -2.8039349737  -3.0875187481
O  -4.6298490006  0.6613932047   0.0360719763
N  -8.0795040973  1.6819110385   1.8762343934
O  -5.9544527089  2.4559794855   1.7372688796
C  -7.1620050594  2.6739830458   1.8232614211
C  -7.6955613695  4.0847687615   1.8903144489
H  -7.4670441167  -3.3218492086  -0.6930319835
H  -9.6025787066  -2.3401410309  -0.0849066504
H  -9.7532037384  -0.0212322161  0.2459875998
H  -6.9715623246  0.5770060492   -0.2506407566
H  -5.2138847062  -2.6078484041  0.4021486778
H  -4.7796482631  -2.5979653704  -1.8650389732
H  -4.8556840303  -0.8237148635  -1.9552472697
H  -7.1502971345  -0.848474263   -2.4180453022
H  -9.027190334   -2.3622921977  -2.691710041
H  -9.4623018481  0.0004101101   -2.2198532519
H  -3.5544457316  -1.001964572   0.5072334962
H  -4.7655185618  -0.5713852055  1.7064647669
H  -8.4422788662  -0.3278618861  2.2672290951
H  -6.7529038788  0.161087639    2.2717522379
H  -6.6548310904  -2.5043833515  -3.9122882833
H  -5.0533778978  1.2830478143   0.6574504372
H  -9.0558943554  1.9267095436   1.8425444651
H  -7.1360219625  4.6338656911   2.650022302
H  -7.5118561255  4.5751619188   0.9306786438
H  -8.7625554173  4.140751591    2.1178723266
```

calc_**2a** conf_2

```
C  -8.9091610546  -1.0652231709  -1.061132996
C  -8.606556514   0.2426218929   -0.3449356825
C  -7.0875593197  0.3437783976   -0.5200133663
N  -6.6903398594  -1.0021476989  -0.0808471435
C  -7.7016704791  -1.9324348669  -0.6731685038
C  -5.4047998589  -1.5653348708  -0.5325676356
C  -5.6466049015  -2.0034203345  -1.9729764484
C  -7.0039833171  -2.6938339507  -1.8435802948
O  -8.9408622414  -0.8374004415  -2.4556000059
O  -9.3297215892  1.300525271    -0.9035817301
C  -4.2412275081  -0.6658105846  -0.1808968141
C  -6.5051914474  1.5580495933   0.2026253616
```

O	-6.7465779483	-4.0496815888	-1.5038245095
O	-4.3356495958	-0.3039837295	1.1901396226
N	-6.767226238	1.6425877156	1.6248781093
O	-8.7512091386	2.7246633783	1.418590544
C	-7.8449415457	2.2843512583	2.1274342671
C	-7.8844781582	2.449717176	3.6280488772
H	-7.9863046708	-2.6721621678	0.0833869799
H	-9.8564070571	-1.5120415445	-0.7307156184
H	-8.8234880193	0.1234901105	0.724351821
H	-6.8994240569	0.5152637496	-1.5928618671
H	-5.2751741309	-2.4857052116	0.0553044407
H	-4.8959246283	-2.6953061861	-2.3610295361
H	-5.7165614356	-1.1430527891	-2.6458348116
H	-7.5912152977	-2.628069462	-2.7627589943
H	-9.388145585	0.018013406	-2.5615080579
H	-9.2937276892	2.0173679495	-0.238806938
H	-3.2954224482	-1.1931345214	-0.3451779593
H	-4.2270592803	0.2324866079	-0.8143751343
H	-5.427842499	1.6339035794	0.0664133491
H	-6.9616104359	2.4345346431	-0.2679041964
H	-7.5950814127	-4.5078459486	-1.4628967692
H	-5.2200517833	-0.6081532401	1.4549316763
H	-6.0261431536	1.3591255085	2.2458858632
H	-7.0674829698	1.9433161497	4.1466829421
H	-7.8474989733	3.516516402	3.8633936603
H	-8.8376737582	2.0684295189	3.9999274985

calc_**2a** conf_3

C	-8.9959640431	-1.6193951558	-0.910622647
C	-8.8767397137	-0.359612495	-0.0104255835
C	-7.3902711901	-0.2768134808	0.408072903
N	-6.912388065	-1.6611619917	0.2694991253
C	-7.5727670741	-2.167445106	-0.9383703575
C	-5.4869948082	-1.8822798727	-0.0390224351
C	-5.3104077119	-1.6591159542	-1.5694088786
C	-6.7399978342	-1.6302489723	-2.1176550943
O	-9.4652392619	-1.3236883353	-2.2081892675
O	-9.3145800696	0.755890853	-0.7398660996
C	-4.4620510622	-1.1782694943	0.8387040236
C	-7.1947355465	0.2873196432	1.8270526077
O	-6.8771297719	-2.3948967428	-3.2895302073
O	-4.4258034658	0.2364160947	0.7354777686
N	-6.7704474649	1.6720836213	1.8444329296
O	-8.8053781805	2.5109493959	1.3090592666
C	-7.6072805441	2.6852402816	1.5493482419
C	-7.0093435216	4.0722083237	1.5529667215
H	-7.5308935717	-3.2633620153	-0.9658250549
H	-9.6782409135	-2.3402057777	-0.4473446575
H	-9.4963966004	-0.4937375254	0.8851686713
H	-6.8945338462	0.4120759943	-0.2926647307
H	-5.3229377965	-2.9513760639	0.1520705913
H	-4.7555656834	-2.481710325	-2.0282119887
H	-4.7734977245	-0.7352979121	-1.8134876011
H	-7.0413958026	-0.5933766058	-2.3162259114
H	-9.6905581552	-0.3762246019	-2.1878806348
H	-9.3183414356	1.5099782436	-0.1144809666
H	-3.4744282025	-1.6005847413	0.6047152598
H	-4.660570056	-1.3959699407	1.890661277

H	-8.1257169479	0.2136293231	2.3955262821
H	-6.4436464202	-0.2892171241	2.3640565478
H	-7.8057368603	-2.2801272109	-3.5439538643
H	-4.1835901792	0.4573128143	-0.1724836705
H	-5.7734977037	1.8272828364	1.8260415515
H	-5.9507107573	4.0931961961	1.8208990242
H	-7.1407529529	4.5183124416	0.5639951213
H	-7.567469061	4.6882213819	2.2619017369

calc_**2a** conf_4

C	-8.945953715	-1.6949082974	-0.6364476942
C	-8.7882312137	-0.3008487952	0.025238083
C	-7.2782987821	-0.1537527352	0.3508222841
N	-6.718733443	-1.5145109376	0.1755964308
C	-7.5098078545	-2.1112044561	-0.9057943905
C	-5.3510416566	-1.5969857054	-0.386165126
C	-5.464366166	-1.2595096947	-1.8881198992
C	-6.9409213054	-1.5308647848	-2.2204854829
O	-9.7249854459	-1.6636171732	-1.808956521
O	-9.2450699825	0.6674710364	-0.8873179786
C	-4.326177465	-0.8235071448	0.4122821063
C	-7.0012940003	0.4114478237	1.7575061507
O	-7.0772102749	-2.4170847745	-3.3073365463
O	-4.2733968114	-1.2707693638	1.75611044
N	-6.7597084739	1.8459109952	1.7832163643
O	-8.8224900463	2.4856555623	1.099169124
C	-7.6882594879	2.77621477	1.4802268114
C	-7.2681270978	4.218811477	1.6403597069
H	-7.3682813524	-3.1980268388	-0.9262206452
H	-9.4104271717	-2.385072168	0.0762903658
H	-9.3777350773	-0.2431869041	0.9465937952
H	-6.8667730157	0.5559969867	-0.3828775731
H	-5.077877686	-2.6576353223	-0.3010121242
H	-4.8189678113	-1.8984429775	-2.4954383342
H	-5.1909630893	-0.2199519379	-2.0955590298
H	-7.4512721371	-0.5859002364	-2.4464152429
H	-9.9374746486	-0.7226067928	-1.9445180712
H	-9.3011194059	1.495170134	-0.3721889562
H	-4.5515375336	0.2536296521	0.3794256299
H	-3.3355773952	-0.9577385032	-0.0360586616
H	-7.8373160947	0.1762199498	2.4251255445
H	-6.1076753586	-0.05282886	2.1732505366
H	-8.0329891451	-2.5093509179	-3.4357852133
H	-5.0561277076	-1.8263200272	1.8847027482
H	-5.8582478911	2.1600831519	2.1037883087
H	-7.9346595173	4.6972867497	2.3619141424
H	-6.2351459041	4.346666973	1.9716204723
H	-7.4027588352	4.732060087	0.6854584454

calc_**2a** conf_5

C	-8.9300797112	-1.5715042537	-0.4871691338
C	-8.6739907838	-0.1151911245	-0.0383440022
C	-7.1776485978	-0.0644980167	0.3513114492
N	-6.6569832509	-1.4439059621	0.1733445309
C	-7.5336003128	-2.0804222513	-0.8137673977
C	-5.3286321765	-1.5792842307	-0.4660159001
C	-5.5372943149	-1.2976675693	-1.9634408785

C	-7.0090982103	-1.6665578035	-2.2137666807
O	-9.8270696538	-1.6674319852	-1.5652810269
O	-8.9705275168	0.7143923444	-1.1379727939
C	-4.2472691016	-0.7961599908	0.2451300786
C	-6.9525520197	0.3614566303	1.803121061
O	-7.1134163965	-2.7165782947	-3.1532136033
O	-4.1366099569	-1.197215644	1.5988563179
N	-7.5267120999	1.6460234644	2.1993947702
O	-7.7436868079	2.7555429834	0.2107713495
C	-7.8148177356	2.7255416035	1.439536553
C	-8.2577297584	3.9551877432	2.2031530837
H	-7.4522666679	-3.1712863152	-0.7416536719
H	-9.3416383778	-2.143564581	0.3510169517
H	-9.3102484066	0.1540859489	0.816058339
H	-6.6881013138	0.6474718765	-0.3200851968
H	-5.0729403791	-2.641629279	-0.354388496
H	-4.8822275754	-1.903132037	-2.5940929245
H	-5.3503112139	-0.2453724327	-2.2013409283
H	-7.5580756195	-0.788377778	-2.5713816165
H	-9.8718258919	-0.7703389614	-1.9384214225
H	-8.6456870544	1.6042638175	-0.8973316514
H	-4.4469078419	0.2844641598	0.1779258982
H	-3.2834104238	-0.9747818236	-0.2429851298
H	-7.4085012654	-0.4038969429	2.4412783955
H	-5.8797480592	0.3639762802	2.023088746
H	-8.0615035683	-2.8766634515	-3.2615436131
H	-4.9737332393	-1.6345312965	1.8129158208
H	-7.5970900635	1.7799103942	3.1958055205
H	-8.3459217571	3.8093554381	3.2821396821
H	-7.539926758	4.7567274225	2.011637716
H	-9.2212161176	4.2825919182	1.8067098039

calc_**2a** conf_6

C	-9.0095328405	-1.4432301304	-0.7004649528
C	-8.673132616	-0.0483529952	-0.1157772281
C	-7.2261610508	-0.1572840526	0.420255519
N	-6.8521066941	-1.584231128	0.2816751552
C	-7.6449532926	-2.1008144748	-0.8312585337
C	-5.4786300756	-1.9135518734	-0.1372116177
C	-5.4175072944	-1.7015856517	-1.6730390926
C	-6.8864401528	-1.7134058547	-2.1187989785
O	-9.7010092557	-1.3734137988	-1.9262196172
O	-8.7852551303	0.881643606	-1.1698271377
C	-4.3445172812	-1.296390912	0.6668551061
C	-7.0811441672	0.2418466306	1.8884430988
O	-7.1059238005	-2.6204205754	-3.1755778293
O	-4.2080115311	0.1073005784	0.5695971524
N	-7.6493980533	1.5348489925	2.2687260534
O	-7.465515926	2.7380643273	0.3350346434
C	-7.7315456594	2.6655368441	1.5354851444
C	-8.1996202145	3.8949483634	2.2846639998
H	-7.693163416	-3.1962376548	-0.789046709
H	-9.6279802139	-2.000106141	0.0107274761
H	-9.3704687923	0.2164901862	0.6907159436
H	-6.5937788913	0.4963623295	-0.1861251518
H	-5.3881864625	-2.9940202323	0.0415183757
H	-4.8667755316	-2.5035926765	-2.1712659341
H	-4.9368641314	-0.7560275292	-1.9477401135

H	-7.1892075425	-0.7030203334	-2.421862133
H	-9.615972422	-0.4452809387	-2.2079282387
H	-8.359643044	1.7047795109	-0.8556518354
H	-3.41104352	-1.7995366429	0.3736789647
H	-4.5018107115	-1.5054344595	1.728602995
H	-7.5865255105	-0.5185289126	2.4912681695
H	-6.0185850062	0.2315973138	2.1471565454
H	-8.057408089	-2.5708956953	-3.3515680953
H	-3.9956659066	0.3258219914	-0.3462041387
H	-7.8618762567	1.6294023411	3.2491489651
H	-7.4016193671	4.6411392138	2.2542370913
H	-9.0584938776	4.3180022044	1.7592537615
H	-8.4725262714	3.7115782297	3.3265231766

calc_**2a** conf_7

C	-9.0389938442	-1.6023677418	-0.8076975225
C	-8.8942408553	-0.2090541948	-0.1407805263
C	-7.4474239363	-0.1543536582	0.4160876571
N	-6.9541883753	-1.5411743247	0.3340042064
C	-7.6114109251	-2.1245969079	-0.8369306913
C	-5.5232204355	-1.7191408635	0.0130287319
C	-5.3751128977	-1.544623204	-1.5215845274
C	-6.8078132995	-1.6418048372	-2.0589735897
O	-9.6131403314	-1.5400039417	-2.0935568456
O	-9.136598499	0.7707022014	-1.1206905458
C	-4.5594158732	-0.8718796651	0.8309389781
C	-7.3616339902	0.3890520824	1.8561877173
O	-6.9063655556	-2.5179566976	-3.1575959642
O	-3.2188040065	-1.0917136522	0.4489361488
N	-6.9684763881	1.7863602403	1.9420921292
O	-8.7936434346	2.6092058969	0.8871762776
C	-7.7225386166	2.798347977	1.4629135405
C	-7.1869212311	4.1945548745	1.6768639783
H	-7.5511386934	-3.2192334952	-0.8017571209
H	-9.6644827991	-2.2432769844	-0.1771756353
H	-9.6166865006	-0.099172862	0.6758944969
H	-6.8867458003	0.5298374574	-0.2402007895
H	-5.3070862761	-2.7698418685	0.2578783463
H	-4.761775064	-2.3348856983	-1.9607234217
H	-4.901913594	-0.591781505	-1.7747740487
H	-7.1652510412	-0.6436326685	-2.3469097446
H	-9.7234933256	-0.5895886303	-2.2752591001
H	-9.1754991108	1.6159480587	-0.6346608919
H	-4.7015100083	-1.067659458	1.905251661
H	-4.7377023009	0.1940423147	0.6563013218
H	-8.3240661801	0.2583831423	2.3622659597
H	-6.6274288897	-0.1855834464	2.4201943523
H	-7.8459011741	-2.5062469442	-3.3956387727
H	-3.0275894166	-2.0298704948	0.5762754389
H	-6.1022404218	2.0111796559	2.403294825
H	-7.0742908063	4.6805170448	0.7049006124
H	-7.9253314551	4.7679582891	2.2421674516
H	-6.230924647	4.2273545085	2.2042559071

calc_**2a** conf_8

C	-8.9364183686	-1.26106666	-0.4254765719
C	-8.6525066805	0.1729288926	-0.9448394261

```
C  -7.1530473372  0.422355723    -0.6577830258
N  -6.6578177862  -0.8171470137  0.0023263031
C  -7.5559030094  -1.8869243853  -0.4057809288
C  -5.3294695534  -1.3413646531  -0.385628424
C  -5.5362204186  -2.1052262047  -1.7188436474
C  -7.0579954844  -2.3338783732  -1.7920970608
O  -9.8538813631  -1.929264798   -1.2694009831
O  -8.8811410154  0.2409018826   -2.3334589614
C  -4.2227315708  -0.3044613204  -0.3917100308
C  -6.9026374054  1.6417847388   0.2285288693
O  -7.5056951623  -3.6691852604  -1.9867201761
O  -3.9432792971  0.2021219564   0.892390602
N  -7.3185278598  1.3550708403   1.5805426673
O  -7.8655545201  3.4896729061   2.1027007189
C  -7.7777268657  2.3167609915   2.427947558
C  -8.1697344279  1.8321154861   3.808526406
H  -7.4774944469  -2.7442725106  0.2745713773
H  -9.3484225465  -1.2125304758  0.5885072742
H  -9.2751332271  0.9114495297   -0.4228241247
H  -6.6779544735  0.5988668139   -1.6318435121
H  -5.0687140196  -2.0687145656  0.3925625597
H  -5.0008157635  -3.0587885334  -1.7149125748
H  -5.1806672436  -1.5245294507  -2.5765891966
H  -7.4943622415  -1.6825304467  -2.5570165629
H  -9.4287819098  -2.7440146793  -1.5839713153
H  -9.5931186638  -0.4052169131  -2.490986761
H  -4.4582452828  0.5007398451   -1.1054226399
H  -3.3004851018  -0.7772764717  -0.7412351677
H  -5.8445693652  1.934135941    0.1960764706
H  -7.4710774302  2.5017868793   -0.1341130173
H  -7.3232752571  -3.9226569043  -2.9005428009
H  -4.7831389988  0.4577698406   1.2960912926
H  -7.2500855029  0.3870192795   1.8629505229
H  -7.5198460153  2.3094569884   4.5463300068
H  -9.1909977887  2.1581583803   4.017298225
H  -8.1075265957  0.7479527046   3.9318460558
```

calc_**2a** conf_9

```
C  -8.8454894554   -1.0225025795  -0.6812051774
C  -8.1965345749   0.3667505145   -0.7140601033
C  -6.8847190564   0.1608237863   0.0476235806
N  -6.6357390955   -1.3060476932  0.0832401656
C  -7.6546792368   -1.9681762172  -0.7436646569
C  -5.366630498     -1.7842403623  -0.4987219472
C  -5.5882315165   -1.7153960831  -2.0094626736
C  -7.0224610659   -2.231060491   -2.1524061993
O  -9.7266724768   -1.2326836684  -1.7743378432
O  -7.9065233543   0.7181778029   -2.0531376653
C  -4.1575636208   -1.1071899055  0.1105712139
C  -6.9248275019   0.6939553038   1.4858987086
O  -6.9686201574   -3.6204360168  -2.4348523609
O  -4.1485642671   -1.2787771066  1.5154298082
N  -7.0890176199   2.1285070101   1.571610348
O  -9.3223232957   2.0798071948   1.9635498479
C  -8.2867851972   2.7183122075   1.8319002271
C  -8.2618344267   4.2273774816   1.948292518
H  -7.8983605629   -2.9487633129  -0.3190189053
H  -9.3692085845   -1.1626703071  0.2715651169
```

H	-8.8303093912	1.1260066715	-0.2420608348
H	-6.1045468404	0.6956374922	-0.5068459287
H	-5.3021550291	-2.846022103	-0.2225784719
H	-4.8961486	-2.3294247088	-2.5900270955
H	-5.5307014077	-0.6811651378	-2.3615491314
H	-7.5621272869	-1.7069366322	-2.944710004
H	-10.6009358269	-0.9139739719	-1.5173167744
H	-8.6420731299	0.3559786125	-2.5736693589
H	-4.1306846197	-0.0378490568	-0.1476801267
H	-3.2448211923	-1.5560823876	-0.2954072483
H	-7.7564924754	0.239506378	2.0287209806
H	-5.9950789069	0.4158868963	1.9894584212
H	-7.8771754777	-3.9204344172	-2.565305973
H	-5.0627054452	-1.4887911661	1.7584451155
H	-6.2755303388	2.7117756019	1.4620812649
H	-7.2729477585	4.6689374235	1.8025074546
H	-8.9502894109	4.6474948063	1.2114005334
H	-8.6334912979	4.5076881411	2.9367231747

calc_**2a** conf_10

C	-8.935405349	-1.8835560535	-0.5962126635
C	-8.9174379342	-0.4183917732	-0.0759531598
C	-7.4534308321	-0.1632771089	0.3538310248
N	-6.7428594136	-1.4546895612	0.1620895354
C	-7.473725145	-2.1457953559	-0.8896744947
C	-5.3750024034	-1.3960207215	-0.4008988715
C	-5.5360307902	-1.1051994119	-1.9117442048
C	-6.9921167712	-1.5125624163	-2.2115635328
O	-9.7777748287	-2.0028456593	-1.7279981575
O	-9.2968293118	0.4701879282	-1.0984778311
C	-4.4453890738	-0.4965817279	0.3864567078
C	-7.331546948	0.3246561327	1.7996506782
O	-7.2039590553	-2.4762479893	-3.2363796689
O	-4.2721702483	-0.9909751999	1.7025553417
N	-7.9142042663	1.6360654402	1.9848536369
O	-6.1401209888	2.7174836749	1.0847016148
C	-7.2685953908	2.7551384148	1.553288011
C	-8.0420072375	4.0489277504	1.68357371
H	-7.2192779637	-3.2126137318	-0.9104420637
H	-9.2948153927	-2.555659304	0.1895863638
H	-9.5992524181	-0.3085316748	0.7812668318
H	-7.0627955444	0.6286556172	-0.2977072109
H	-4.983211551	-2.4157224323	-0.2932275618
H	-4.8341949814	-1.6963685074	-2.5068135734
H	-5.3651959407	-0.0486139857	-2.1410378473
H	-7.5885257851	-0.6178259499	-2.422488127
H	-9.2393495453	-2.3399032359	-2.4630890924
H	-9.8632630059	-0.0676720274	-1.6823422651
H	-4.8146279517	0.5386315313	0.3980771169
H	-3.4585583273	-0.4899190079	-0.0871553875
H	-7.8287114115	-0.3800474333	2.4735386308
H	-6.2817184758	0.3841237025	2.0933288016
H	-7.0189446405	-2.059907024	-4.0879059445
H	-5.1082724443	-1.4139439343	1.9435495825
H	-8.8858888505	1.7057059949	2.2393512776
H	-8.8960318672	3.9812626423	2.3621633222
H	-7.3628173694	4.8273246166	2.0343567984
H	-8.4039405456	4.3457077813	0.6948926721

calc_**2a** conf_11

C	-8.9948258629	-1.4598172151	-0.6345882926
C	-8.6594651123	-0.0609527874	-0.0545972288
C	-7.1906437564	-0.1449517496	0.4218244718
N	-6.8151413159	-1.5738698287	0.3050392211
C	-7.6294910094	-2.1113219826	-0.782823291
C	-5.449457274	-1.9158845765	-0.1381890914
C	-5.4196307832	-1.7623222206	-1.680944202
C	-6.8979295764	-1.7408586213	-2.0888709941
O	-9.7014045193	-1.3936553951	-1.8525964454
O	-8.8351284245	0.8763300236	-1.0935209421
C	-4.3020386551	-1.2537522058	0.597203364
C	-6.9876928875	0.2921435296	1.8747449189
O	-7.1731132809	-2.6410760383	-3.1388414947
O	-4.2326510688	0.1250279447	0.2845873143
N	-7.6197319117	1.5488980512	2.2722402705
O	-7.4670048356	2.7648315383	0.3485405191
C	-7.7567082703	2.6762372309	1.5415865205
C	-8.3255237965	3.8689822861	2.2804135753
H	-7.6709356699	-3.2064914162	-0.722905233
H	-9.6041642463	-2.0183460185	0.0831536493
H	-9.3286724773	0.1820586124	0.7820745845
H	-6.5862192828	0.4949374035	-0.2265091655
H	-5.3480122497	-2.9845422776	0.0953153844
H	-4.9161298832	-2.6072682345	-2.1585719391
H	-4.9040857427	-0.8459925467	-1.9754734677
H	-7.1852050425	-0.7225434058	-2.3827663297
H	-9.6221185015	-0.4651140847	-2.1355218684
H	-8.376713764	1.6936806169	-0.8138962398
H	-3.3786628942	-1.7676533549	0.293694371
H	-4.4284283953	-1.4117112221	1.678966037
H	-7.4056510197	-0.4884988833	2.5164480848
H	-5.9124172897	0.3531650939	2.0688733864
H	-8.1294583962	-2.5695421017	-3.2785387666
H	-3.4063162999	0.4739558797	0.6384051072
H	-7.8707297424	1.6147894534	3.245586888
H	-8.5412361029	3.6850377799	3.3355431959
H	-7.614488889	4.6946745362	2.2015137895
H	-9.2437717701	4.1844161866	1.7794003382

calc_**2a** conf_12

C	-9.0203484916	-1.397402325	-0.6914499079
C	-8.7203904857	0.0257810319	-0.1583571334
C	-7.2930823426	-0.0460427783	0.4347909491
N	-6.8818155028	-1.4620047793	0.3388436602
C	-7.6433829246	-2.0397396336	-0.7659321428
C	-5.4866095196	-1.7589837937	-0.0352867532
C	-5.4057486735	-1.660418195	-1.5831247925
C	-6.8660189064	-1.6970527614	-2.0525993734
O	-9.6883042122	-1.3895210887	-1.9319100447
O	-8.8021013615	0.9100159025	-1.2530184604
C	-4.4193939672	-0.9563839267	0.6941484823
C	-7.2309374983	0.3746798029	1.9058854949
O	-7.0633941485	-2.6418560237	-3.0795284622
O	-3.1243478877	-1.4154787102	0.3703627356
N	-7.8264975247	1.6658732704	2.2410706079

O	-7.5736945389	2.8248020017	0.2908282602
C	-7.9047840868	2.7766452619	1.4757307187
C	-8.4517392746	4.007013128	2.166227601
H	-7.6773391575	-3.1324695658	-0.6751564372
H	-9.6461128139	-1.9332436266	0.0294056551
H	-9.4491261407	0.3160143152	0.6107127214
H	-6.6595508659	0.6178076179	-0.1633172189
H	-5.3222486616	-2.8063550212	0.2442575414
H	-4.8593214456	-2.5017331143	-2.0170902302
H	-4.9135413088	-0.7359885971	-1.9096582873
H	-7.1670481154	-0.6989970462	-2.3965700556
H	-9.6022430371	-0.4772519928	-2.2597219923
H	-8.4189245526	1.7568389665	-0.9509004167
H	-4.5198790662	-1.0919870486	1.774393432
H	-4.5211661062	0.1189939586	0.4776999192
H	-7.762912814	-0.3866495927	2.483787431
H	-6.1890334724	0.363350951	2.244408055
H	-8.012191966	-2.6070969912	-3.2725798532
H	-2.98872818	-1.2600744224	-0.5730028141
H	-8.1036815457	1.7689714048	3.2043956995
H	-9.3113198095	4.3718124307	1.5996406004
H	-8.7507122306	3.8458867472	3.2044778424
H	-7.6883273631	4.7882442431	2.1341369685

calc_**2a** conf_13

C	-8.9625560157	-1.4332445971	-0.5170484612
C	-8.6088695982	0.0209431887	-0.1826249012
C	-7.1757002644	-0.0337390022	0.3687392521
N	-6.6833355432	-1.4192899515	0.1452522751
C	-7.5983775412	-2.0694533255	-0.8102378416
C	-5.3730690807	-1.5160149385	-0.5302529044
C	-5.6638836579	-1.1619668193	-1.9891083702
C	-7.0107828288	-1.8687426552	-2.2424761868
O	-9.9193896317	-1.5450426523	-1.5279177056
O	-8.6721586191	0.7440880987	-1.3932644243
C	-4.2797709196	-0.7755231407	0.2101861108
C	-7.0788282599	0.2901260842	1.8586585435
O	-6.8804555722	-3.0896659419	-2.9431139087
O	-4.1595820188	-1.2645911123	1.5334505516
N	-7.586975831	1.6021652385	2.2532527849
O	-7.6938135527	2.7625973041	0.2794629276
C	-7.8012585162	2.7103218085	1.5022091192
C	-8.2213714383	3.9274824088	2.3046907443
H	-7.6303240888	-3.1493855105	-0.6187815334
H	-9.3714062779	-1.9132078136	0.3790997325
H	-9.3101079611	0.4451375313	0.5472828561
H	-6.5859863812	0.696260232	-0.1891514566
H	-5.1009139019	-2.5822583975	-0.482163043
H	-4.9023993763	-1.5046437462	-2.6942081624
H	-5.7713574155	-0.0784651623	-2.1071987512
H	-7.6691684995	-1.2630610891	-2.8638948027
H	-9.8214925954	-0.7428855661	-2.0658105508
H	-8.4181523906	1.6534452895	-1.1551125123
H	-4.4733639223	0.3074345617	0.2120713811
H	-3.3216198015	-0.9295163171	-0.296522703
H	-7.6498877165	-0.4657404318	2.4102613389
H	-6.0330725816	0.1996164575	2.1742681004
H	-6.3554195745	-3.6897578963	-2.3968879514

H	-5.0176524285	-1.6646383081	1.7402780982
H	-7.6855488724	1.7280819198	3.2492573023
H	-7.4734615812	4.1829444483	3.0615865437
H	-8.3393787393	4.7674501119	1.6221848558
H	-9.1721070044	3.7487396918	2.8165836529

calc_**2a** conf_14

C	-9.0500828562	-1.7721434218	-0.778782263
C	-8.9940485441	-0.2867717058	-0.3231123136
C	-7.615705539	-0.1289222415	0.3589372061
N	-6.9818237255	-1.4656901654	0.3169227663
C	-7.5879552344	-2.169278864	-0.800724759
C	-5.5405977111	-1.5375494566	0.0096202449
C	-5.4056985351	-1.4513529908	-1.5372143115
C	-6.836271186	-1.6768622524	-2.0486376874
O	-9.6903072019	-1.8853527134	-2.0367411478
O	-9.1010114142	0.5693093379	-1.4343895497
C	-4.674037877	-0.555264807	0.7740140657
C	-7.715752924	0.3611380212	1.8052646351
O	-7.0244965807	-2.6533301731	-3.0688199793
O	-3.3395639753	-0.8056950029	0.3712627302
N	-8.1804940861	1.7284738123	1.9001998153
O	-6.132742417	2.6211693916	1.5217546796
C	-7.3350458258	2.7739812124	1.6882796359
C	-7.9812681598	4.1420509256	1.6665135122
H	-7.4336692279	-3.2524507279	-0.7143312964
H	-9.5994481838	-2.3684516742	-0.0431540531
H	-9.8078288098	-0.0731825126	0.3873465119
H	-7.062958906	0.6237817725	-0.2189095562
H	-5.2199774168	-2.5400512251	0.3175024845
H	-4.730057167	-2.2224416078	-1.9155573301
H	-5.0082279909	-0.4819340021	-1.8508046067
H	-7.2697565264	-0.7235547618	-2.3735262065
H	-9.0568321379	-2.2922238827	-2.6529043174
H	-9.5934175089	0.0414227391	-2.0907125937
H	-4.7989537708	-0.7259981385	1.8536061368
H	-4.9668536328	0.4819116692	0.5661434798
H	-8.3910224477	-0.2871350223	2.3713829922
H	-6.7370433022	0.3146918339	2.2836649615
H	-6.6361359211	-2.3170185223	-3.8864914433
H	-2.7893067429	-0.1055485199	0.7410273771
H	-9.1723171623	1.9039483234	1.9035436596
H	-7.3220780211	4.8482758176	2.1730623341
H	-8.0837265154	4.4697093378	0.6276927143
H	-8.9674848152	4.1653401975	2.1370714714

calc_**2a** conf_15

C	-8.9439919306	-1.7557997992	-0.6930427533
C	-8.7617613403	-0.2347670347	-0.4357239497
C	-7.2828920487	-0.0643893324	-0.0092013905
N	-6.7375797543	-1.4381585112	0.1030873827
C	-7.5162657413	-2.2414139911	-0.8310269067
C	-5.3554317745	-1.6692159803	-0.3743842947
C	-5.4224173534	-1.7118514651	-1.9220919397
C	-6.9136416644	-1.9551322306	-2.219671275
O	-9.7390903682	-1.9713249086	-1.8440590364
O	-9.0103031564	0.5091060856	-1.606392951

C	-4.3352799929	-0.7252894138	0.2266074237
C	-7.1566865725	0.7150667225	1.2931945186
O	-7.2415971551	-3.057923741	-3.0545648106
O	-4.2251724156	-0.891995437	1.6210290413
N	-7.6988863658	2.0441837509	1.1048762613
O	-7.2467796437	2.6844854318	3.2209664885
C	-7.7104519428	2.9459516789	2.1224046855
C	-8.328270925	4.2914454775	1.8032921869
H	-7.3967605087	-3.3110235605	-0.6205392219
H	-9.4278672607	-2.2226972997	0.1703229713
H	-9.4327098597	0.1012824097	0.3664281336
H	-6.7930441989	0.5095720312	-0.8115713584
H	-5.0969483213	-2.673135469	-0.0162278859
H	-4.8089637656	-2.5241312616	-2.3217572839
H	-5.073403271	-0.7759910474	-2.3710013562
H	-7.3651933069	-1.0422580376	-2.6258261246
H	-9.2257274858	-2.5173319922	-2.4627585167
H	-9.6229646482	-0.0482118441	-2.1214919854
H	-4.5772208603	0.3162330987	-0.0393747524
H	-3.3502097484	-0.9415870837	-0.1980477336
H	-7.7018393278	0.1911798011	2.0882695364
H	-6.1158683362	0.7937321419	1.6215000941
H	-6.9746440314	-2.8479114986	-3.9586761856
H	-5.1158973042	-1.0638905291	1.9565979261
H	-8.128372854	2.2717207757	0.220901075
H	-9.1534484252	4.472205462	2.4961883553
H	-7.5816823283	5.0697685396	1.9780147643
H	-8.6987340118	4.3784980618	0.7787508677

calc_**2a** conf_16

C	-8.9644114101	-0.8944784265	-0.5175079932
C	-8.4785861238	0.4518892069	-1.102766798
C	-6.989726908	0.5528160301	-0.6898763165
N	-6.6854364586	-0.6904253691	0.0591913402
C	-7.673170133	-1.6735502849	-0.3615609194
C	-5.4137563664	-1.3814662069	-0.2272969828
C	-5.605747431	-2.1136319363	-1.5715453366
C	-7.138726933	-2.2734369106	-1.6816517977
O	-9.8970692387	-1.5108873509	-1.3910464386
O	-8.5617788787	0.4279779631	-2.5155100257
C	-4.2253284259	-0.4587008373	-0.0665762011
C	-6.6128644956	1.8483564289	0.0692035321
O	-7.6378779081	-3.6019794994	-1.7664987465
O	-4.2486709653	0.1205124982	1.2234025921
N	-6.6774293066	1.8081059854	1.515777935
O	-8.9232069479	1.5657299588	1.6739891631
C	-7.8348549794	1.6943374649	2.215885684
C	-7.6941030325	1.7209102736	3.7232932878
H	-7.7436096984	-2.4885000802	0.3701034693
H	-9.4274548772	-0.7174652333	0.4562358619
H	-9.0566214244	1.2863379399	-0.6976938108
H	-6.4418435833	0.5977800724	-1.6426492982
H	-5.324258613	-2.1428680843	0.5594157856
H	-5.1071559106	-3.086982303	-1.5766842924
H	-5.211715778	-1.5289672109	-2.4094578738
H	-7.5092018862	-1.6828204968	-2.525576085
H	-9.5855771871	-2.4109655928	-1.5707122589
H	-9.3312048758	-0.1410095737	-2.6961069596

H	-4.2327438346	0.317682585	-0.8477269849
H	-3.2919387159	-1.0202698418	-0.1783869058
H	-5.5825283775	2.1179952534	-0.1694179054
H	-7.2550907412	2.6522990146	-0.307495519
H	-7.4412001403	-3.9424191513	-2.6483542471
H	-5.1781030857	0.063381844	1.5033117404
H	-5.8226797862	1.9924524657	2.0157844022
H	-8.3749755708	2.4741129178	4.1252330505
H	-8.0091383762	0.7525637585	4.120374429
H	-6.680211595	1.9305827287	4.072897424

calc_**2a** conf_17

C	-8.9726266831	-1.8163430188	-0.9490636829
C	-8.9944051157	-0.6252996374	0.0434147369
C	-7.5166287435	-0.2231247779	0.2231923242
N	-6.8528837172	-1.5307890883	0.1228619407
C	-7.4943161891	-2.186007421	-1.0284252624
C	-5.4224647425	-1.591738583	-0.2552694576
C	-5.3543385557	-1.3115373594	-1.7671669057
C	-6.7522978701	-1.6667087678	-2.2918380411
O	-9.4481397595	-1.4279843007	-2.2337660605
O	-9.8548407853	0.4113355954	-0.3614313106
C	-4.5148546731	-0.752976027	0.6177653921
C	-7.3152904339	0.5132807782	1.5513646701
O	-6.6824208584	-2.6399477589	-3.3117066083
O	-4.6085050317	-1.1379820891	1.974677388
N	-7.9231650771	1.8238702343	1.5121466566
O	-5.9542243339	2.906273804	1.1631875426
C	-7.1726004501	2.9327273499	1.2471931451
C	-7.9530877849	4.2219342171	1.1007723236
H	-7.3251791997	-3.2675345188	-0.9840506097
H	-9.5754164529	-2.6499536922	-0.5747369806
H	-9.372306003	-0.9709918368	1.0104976137
H	-7.2304196379	0.4688353388	-0.5891138091
H	-5.1418253845	-2.639983683	-0.0878205511
H	-4.6019901718	-1.9288789079	-2.2634160427
H	-5.1103498688	-0.2639144264	-1.9687947788
H	-7.2475284272	-0.7697967444	-2.6776120013
H	-10.4132824626	-1.4409254993	-2.1956392339
H	-9.666486846	0.5528618541	-1.3021119558
H	-4.7326100182	0.3178610783	0.4897975354
H	-3.4773605773	-0.9089297598	0.3049214533
H	-7.7613023058	-0.0762628207	2.3617362634
H	-6.2644821105	0.6663865879	1.790618758
H	-7.5743166207	-2.712660734	-3.6768139071
H	-5.5071790088	-1.4730046349	2.1012685627
H	-8.9155624799	1.867052201	1.3328149343
H	-7.689737738	4.6879107798	0.1485564331
H	-9.0367206624	4.0912522644	1.1514937522
H	-7.6438532194	4.9076940044	1.8934957729

Table S36. Sampled and DFT geometry optimized conformers related to calc_**2b**, with energy values (Hartree) and related % contribution on the final Boltzmann distribution for the three employed functional/basis set combinations.

Conformer	MPW1PW91/6-31g(d,p)		MPW1PW91/6-311+g(d,p)		B97-2/cc-pVTZ	
	**Energy (Hartree)**	**% contribution on the Boltzmann distribution**	**Energy (Hartree)**	**% contribution on the Boltzmann distribution**	**Energy (Hartree)**	**% contribution on the Boltzmann distribution**
calc_**2b** conf_1	-916.6558506	66.58%	-916.8926282	19.95%	-916.8414899	26.77%
calc_**2b** conf_2	-916.6545098	16.09%	-916.8935168	51.14%	-916.8420943	50.77%
calc_**2b** conf_3	-916.6542962	12.83%	-916.8927758	23.33%	-916.8404895	9.28%
calc_**2b** conf_4	-916.6527150	2.40%	-916.8886848	0.31%	-916.8374863	0.39%
calc_**2b** conf_5	-916.6516843	0.81%	-916.8908171	2.93%	-916.8403837	8.29%
calc_**2b** conf_6	-916.6513944	0.59%	-916.8889588	0.41%	-916.8384095	1.02%
calc_**2b** conf_7	-916.6511811	0.47%	-916.8900930	1.36%	-916.8388397	1.62%
calc_**2b** conf_8	-916.6498398	0.11%	-916.8873110	0.07%	-916.8364653	0.13%
calc_**2b** conf_9	-916.6493910	0.07%	-916.8890092	0.43%	-916.8387838	1.52%
calc_**2b** conf_10	-916.6473366	0.01%	-916.8841813	0.00%	-916.8329573	0.00%
calc_**2b** conf_11	-916.6471566	0.01%	-916.8857487	0.01%	-916.8352583	0.04%
calc_**2b** conf_12	-916.6467867	0.00%	-916.8849720	0.01%	-916.8341934	0.01%
calc_**2b** conf_13	-916.6467284	0.00%	-916.8856732	0.01%	-916.8350680	0.03%
calc_**2b** conf_14	-916.6466014	0.00%	-916.8852076	0.01%	-916.8354701	0.05%
calc_**2b** conf_15	-916.6464851	0.00%	-916.8840561	0.00%	-916.8326546	0.00%
calc_**2b** conf_16	-916.6452414	0.00%	-916.8835624	0.00%	-916.8341376	0.01%
calc_**2b** conf_17	-916.6447394	0.00%	-916.8859038	0.02%	-916.8357834	0.06%
calc_**2b** conf_18	-916.6435924	0.00%	-916.8831703	0.00%	-916.8330099	0.00%
calc_**2b** conf_19	-916.6435609	0.00%	-916.8827571	0.00%	-916.8328211	0.00%
calc_**2b** conf_20	-916.6433206	0.00%	-916.8835240	0.00%	-916.8331326	0.00%
calc_**2b** conf_21	-916.6422478	0.00%	-916.8820942	0.00%	-916.8320366	0.00%
calc_**2b** conf_22	-916.6416031	0.00%	-916.8819705	0.00%	-916.8322649	0.00%
calc_**2b** conf_23	-916.6408353	0.00%	-916.8800713	0.00%	-916.8287722	0.00%
calc_**2b** conf_24	-916.6380444	0.00%	-916.8766959	0.00%	-916.8264854	0.00%

Table S37. Cartesian coordinates of the optimized geometries for the conformers related to calc_**2b**. The related energies and % contribution on the final Boltzmann distribution for the three employed functional/basis set combinations are reported in Table S36.

calc_**2b** conf_1

C	-6.8075237742	-1.5203746315	-1.692037999
C	-7.6191987634	-0.8858882976	-0.56727615
C	-6.581974372	0.041774871	0.0961781365
N	-5.3349084474	-0.7256721602	0.0236376069
C	-5.4807780387	-1.8000679438	-1.0030273798
C	-4.1092991292	0.0494561195	-0.2594844501
C	-3.7151822452	-0.3329055522	-1.6930986438
C	-4.2079038224	-1.7710618226	-1.8714369634
O	-6.6384471252	-0.5796564967	-2.7320250464
O	-8.7316069063	-0.2367976838	-1.1147382967
C	-3.0651863108	-0.2913727507	0.8141387799
C	-6.8821010552	0.4415244932	1.5489181032
O	-3.2676912123	-2.7451004369	-1.4868629739
O	-3.0785261749	-1.6763639748	1.1070625828
N	-7.7383715298	1.605977508	1.7052144703
O	-9.696073753	0.5434742718	1.3106124739
C	-9.0837396968	1.5696303716	1.6087683202
C	-9.8140736157	2.8602565732	1.8939882499
H	-5.5414059147	-2.7827504959	-0.5198032007
H	-7.2767530975	-2.4381996317	-2.071556434
H	-7.9215550748	-1.6638677909	0.1493488504
H	-6.5249195476	0.954958447	-0.5159432868
H	-4.327664788	1.1224634593	-0.1914404961
H	-4.2561900489	0.3104940419	-2.3912121449
H	-2.6433047942	-0.2491173684	-1.8925677217
H	-4.4499726636	-1.979608061	-2.9151001208
H	-7.5005878126	-0.1424016302	-2.8202077091
H	-9.2985768899	0.0248475707	-0.3628970019
H	-3.274406827	0.2814682738	1.7299809383
H	-2.0543407358	-0.0384884667	0.4846666517
H	-5.9396704443	0.6765371772	2.0496886994
H	-7.3346944788	-0.3994907	2.083247507
H	-3.0017516268	-2.5403559504	-0.5697015708
H	-4.0265381042	-1.8551232846	1.2551706674
H	-7.3011476228	2.4869259121	1.9212562788
H	-10.386136178	3.144511506	1.00753463
H	-10.5313277039	2.6811209106	2.698280526
H	-9.1604696743	3.6882436239	2.1777241174

calc_**2b** conf_2

C	-6.9389606379	-1.839455981	-1.2721040309
C	-7.6643916	-0.7270460284	-0.5172259632
C	-6.683556628	0.4379975352	-0.6773121372
N	-5.425638052	-0.2159527286	-0.3500246069
C	-5.4970903486	-1.6320150334	-0.797669786
C	-4.1692956724	0.3933204573	-0.8067922099
C	-3.3197217889	-0.8123920443	-1.2113322464
C	-4.3437483078	-1.7784758096	-1.7952163786
O	-7.0424630926	-1.6312697578	-2.6653900863
O	-8.9397584633	-0.4896157829	-1.0357888864
C	-3.5864128341	1.2555389255	0.3404498571
C	-7.0072273806	1.6668622773	0.1631086458

O	-3.8145843815	-3.0806406646	-1.8830376119
O	-4.0001352933	0.7460678757	1.5869940198
N	-7.1409369391	1.4160551705	1.5807626077
O	-9.3396936944	0.8868101273	1.4423064299
C	-8.3263605318	1.0563129132	2.122472709
C	-8.3516877528	0.8870070285	3.6224976283
H	-5.2922865702	-2.3034225192	0.0462794793
H	-7.3188255583	-2.8343150537	-1.004133733
H	-7.7074021724	-1.0063365411	0.5438478937
H	-6.7102826136	0.7544656105	-1.7335573394
H	-4.3445046021	1.0305279116	-1.6866554493
H	-2.5243146642	-0.5528428712	-1.9136536928
H	-2.8610511811	-1.2715736863	-0.3276041451
H	-4.6754310276	-1.4197033271	-2.7774743209
H	-7.9634430919	-1.3697015885	-2.8216995201
H	-9.3984947843	0.0356801209	-0.3525887745
H	-3.9142596707	2.2994224861	0.2267608238
H	-2.491045148	1.256701104	0.2901123808
H	-7.9556482368	2.0785195256	-0.1923283337
H	-6.2438235161	2.4367969317	0.0229072592
H	-4.4157950009	-3.6003035764	-2.4308024794
H	-4.7144767021	0.1200714281	1.3231505469
H	-6.3059019756	1.4636850552	2.1469886859
H	-8.7217239227	-0.1127852516	3.8603437418
H	-7.3782788871	1.0335442573	4.0954372286
H	-9.0623472755	1.6034615044	4.0419717942

calc_**2b** conf_3

C	-6.8665426371	-1.9521762691	-1.1442396092
C	-7.5843345575	-0.8237112527	-0.3971881649
C	-6.618325309	0.3449010962	-0.589820495
N	-5.3561149999	-0.3030517478	-0.2375425611
C	-5.3805222042	-1.6281353845	-0.9007687544
C	-4.1078998756	0.3910155101	-0.6200883662
C	-3.3841108758	-0.5513863185	-1.5960449074
C	-4.4970401078	-1.4389446947	-2.1406044317
O	-7.1685717011	-1.8907644382	-2.523153709
O	-8.8671774741	-0.6233873339	-0.9141112405
C	-3.3233369926	0.7095637308	0.669840099
C	-6.9538142489	1.6112603009	0.1868363287
O	-3.9849173428	-2.6343129206	-2.6769891636
O	-4.2221643818	0.9874732152	1.7209595982
N	-7.1666286883	1.4435551192	1.6089596454
O	-9.3510317921	0.8715669626	1.4101073345
C	-8.3779811606	1.1310933113	2.1197715583
C	-8.4870582043	1.1254451948	3.6257482244
H	-4.9113225322	-2.3838270301	-0.2601088141
H	-7.1375858821	-2.9386835919	-0.7472719004
H	-7.6131738573	-1.0745301634	0.6710890874
H	-6.6460189652	0.6258806152	-1.6576241265
H	-4.367118168	1.3331155197	-1.1198444117
H	-2.8442296139	-0.0200361359	-2.3830757029
H	-2.663175592	-1.185173249	-1.0661854339
H	-5.0705087612	-0.8920282475	-2.8981418641
H	-8.105588133	-1.6405996418	-2.5704559856
H	-9.3262091991	-0.0656315584	-0.2553808501
H	-2.673830589	1.5785909819	0.5347350524
H	-2.6842129826	-0.1479967134	0.9314002437

H	-7.868824643	2.028217815	-0.2438434113
H	-6.1587735641	2.3499567723	0.0588628761
H	-4.715549544	-3.0569181322	-3.1475095872
H	-4.8981510221	0.2925112091	1.5712355586
H	-6.3948180946	1.6639006006	2.2204803741
H	-9.194560119	1.9017643639	3.9282735993
H	-8.9014568555	0.16709694	3.9456617991
H	-7.5353193285	1.294385565	4.1340321117

calc_**2b** conf_4

C	-6.9454285826	-1.5962470955	-1.4525350484
C	-7.7020871632	-0.7980389477	-0.3813418488
C	-6.6200641575	0.1400963137	0.2041748072
N	-5.3285716924	-0.4882687582	-0.0925036855
C	-5.5741254736	-1.7608995152	-0.8102763205
C	-4.3189477212	0.3088636215	-0.8236196746
C	-3.9600950849	-0.520477894	-2.068498622
C	-4.3616118715	-1.9579662519	-1.730666486
O	-6.8685017511	-0.8190246037	-2.6223579318
O	-8.7786229448	-0.1225465562	-0.9885652265
C	-3.1389401219	0.5508274477	0.1259111798
C	-6.7303546616	0.3496501435	1.7221799525
O	-3.3335194263	-2.689982267	-1.1042733151
O	-2.8038154556	-0.6480171188	0.8007433753
N	-8.0598691316	0.6259489266	2.2533227565
O	-8.7644152419	2.0898632108	0.6726429279
C	-8.9426847702	1.5123714368	1.7465231395
C	-10.195918577	1.7450635385	2.557373268
H	-5.6179029209	-2.5994193733	-0.1038471903
H	-7.4218017453	-2.5659161001	-1.6567875684
H	-8.0718881422	-1.4811345835	0.3984968245
H	-6.6949944711	1.1077337733	-0.2996749859
H	-4.7395258355	1.2789020027	-1.1158503774
H	-4.5566594827	-0.174218228	-2.9131480525
H	-2.9006363568	-0.4624981981	-2.3336860576
H	-4.6409370437	-2.5211674987	-2.6238710466
H	-7.7038282833	-0.3170492435	-2.626432313
H	-8.8558495824	0.7592643796	-0.5743168842
H	-3.3993767072	1.33170601	0.8562444564
H	-2.2508961099	0.8823534895	-0.4178246302
H	-6.0390789954	1.1481360338	2.0190286754
H	-6.3894815494	-0.5697455202	2.2051580208
H	-2.9124504666	-2.0943423959	-0.4553897956
H	-3.6759629191	-0.9542226171	1.1183332084
H	-8.2778452185	0.2181638785	3.1483564247
H	-10.2745831546	2.8129496534	2.7738710408
H	-11.0613091086	1.4731604315	1.9483296784
H	-10.2314180779	1.1871284751	3.4957773246

calc_**2b** conf_5

C	-6.7825739263	-1.7426225167	-1.5318658426
C	-7.5158053712	-1.191293345	-0.3110484506
C	-6.5906368096	-0.0443975341	0.1520302395
N	-5.2475636134	-0.5418203151	-0.1492806356
C	-5.3276688178	-1.6943381984	-1.0801430769
C	-4.27921142	0.4319192744	-0.6744512773
C	-3.3257839517	-0.4431580865	-1.4795789466

C	-4.2532106109	-1.4447053792	-2.1531571207
O	-6.9872649152	-0.8669691933	-2.6188018638
O	-8.7988095648	-0.7958968096	-0.6996052958
C	-3.6234129514	1.1778275681	0.4910803878
C	-6.7426129805	0.3361550779	1.642174229
O	-3.5364128721	-2.6025833964	-2.5261551058
O	-3.2967994206	0.2845613804	1.5257439645
N	-7.5414987659	1.5226563676	1.8898064285
O	-9.5477579447	0.5320982123	1.5550079474
C	-8.8896040392	1.5288430501	1.8567625093
C	-9.5682476657	2.8257394199	2.2262186318
H	-5.0761334377	-2.6233734555	-0.5521434546
H	-7.1123810615	-2.7611968487	-1.7816061264
H	-7.5503210091	-1.9640152578	0.4736548489
H	-6.8231148521	0.8404254188	-0.4608168284
H	-4.7737119172	1.1597635656	-1.3392444396
H	-2.7259133284	0.128157053	-2.1918391917
H	-2.6476428247	-0.9776911884	-0.8054790271
H	-4.7259588442	-0.9794634746	-3.0255862063
H	-7.9055619009	-0.5632067874	-2.5187949978
H	-9.2186768437	-0.3631794905	0.0738853534
H	-4.3065412206	1.9654978226	0.8528131629
H	-2.7096352009	1.679164622	0.1541090988
H	-5.7548978502	0.5206979778	2.0674903729
H	-7.1859234297	-0.4957917541	2.1988714925
H	-4.0829468116	-3.1005319826	-3.1458196174
H	-3.9166336559	-0.4585027595	1.3839256382
H	-7.0623065327	2.3751942123	2.1297075326
H	-8.8755963834	3.6323587464	2.476616264
H	-10.2004797485	3.141895855	1.3933448879
H	-10.2247475061	2.6427821491	3.0801745152

calc_**2b** conf_6

C	-6.5767415182	-1.9398427352	-1.5604375083
C	-7.4736655297	-1.3879157914	-0.4534155569
C	-6.6976758772	-0.1418093715	0.0046955261
N	-5.2992481762	-0.5638022284	-0.0605105899
C	-5.1841656164	-1.7442166485	-0.9615285098
C	-4.3332706093	0.4680875957	-0.4834114389
C	-3.9241130538	0.0670346832	-1.9079995737
C	-4.0147185945	-1.4614409573	-1.9297891709
O	-6.843091169	-1.1148054346	-2.6905152969
O	-8.792784055	-1.1313965855	-0.8641490497
C	-3.1878283287	0.5129107974	0.5347656522
C	-7.0946855214	0.345788497	1.3914580923
O	-2.8292005456	-2.1076328581	-1.5480944255
O	-2.7888366103	-0.7957347211	0.9096676997
N	-8.4765834347	0.7660536843	1.4047166989
O	-8.1301749414	2.5564017466	2.7534644928
C	-8.8879197128	1.8692862227	2.0848185115
C	-10.364101368	2.1884712131	1.9684292742
H	-4.9317185768	-2.6447633112	-0.3874034075
H	-6.7909654626	-2.9889172567	-1.7935756768
H	-7.526293069	-2.1122824889	0.3667445998
H	-6.9081693652	0.6610331051	-0.7212390974
H	-4.8216877247	1.4493432674	-0.4820703516
H	-4.6546714648	0.4793026798	-2.610224331
H	-2.9282569325	0.4115030043	-2.1989370462

H	-4.2357466686	-1.8313361447	-2.9360917645
H	-6.5095059056	-1.5531851678	-3.4817697437
H	-8.709754399	-0.7715690718	-1.7625737494
H	-3.5094532532	1.0689545063	1.4252697286
H	-2.3065762261	1.0109865596	0.1233542189
H	-6.4932394414	1.2047868174	1.7002184484
H	-6.9096496785	-0.4579448076	2.1182628632
H	-2.5826710767	-1.7510165674	-0.6705958045
H	-3.6195012854	-1.2046611606	1.2109382858
H	-9.1229079276	0.2535115683	0.8206694377
H	-10.8208623736	2.101620098	2.957672875
H	-10.4739722599	3.2277015792	1.6509554747
H	-10.9035922464	1.5404956833	1.2732302132

calc_**2b** conf_7

C	-6.68367873	-2.0734876136	-1.1824130631
C	-7.3750501844	-1.2714673245	-0.0614807053
C	-6.4668177379	-0.0483406862	0.1470105273
N	-5.135642442	-0.647328554	0.0011858894
C	-5.2279385466	-1.5894504296	-1.1318823041
C	-4.0215706956	0.2842947058	-0.3060003243
C	-3.5954506036	0.0042159177	-1.7614661851
C	-4.7753156372	-0.7610906122	-2.3406029447
O	-7.2564432595	-1.7879805556	-2.4409616886
O	-8.6874064943	-1.0001839245	-0.4564537296
C	-2.8997901437	0.0614070178	0.7067904331
C	-6.6106005332	0.6445106796	1.5107019136
O	-4.4199276916	-1.524860907	-3.4633798466
O	-3.3965309511	0.0877597301	2.0241915342
N	-7.5637455553	1.7395458626	1.5403271881
O	-9.4207109738	0.4566923128	1.7069150576
C	-8.8949103654	1.5706549299	1.675463921
C	-9.7245106872	2.8258391931	1.7996494167
H	-4.5215926268	-2.4174409114	-1.000327317
H	-6.7581237475	-3.1500875988	-0.9861710049
H	-7.349862218	-1.8678641642	0.8619942006
H	-6.6884847366	0.6880226586	-0.6451708958
H	-4.3833315315	1.3137726302	-0.1941539332
H	-3.3614941425	0.9136788433	-2.3193798611
H	-2.7100064045	-0.6424134212	-1.7917723599
H	-5.5774364908	-0.0577863651	-2.5969951457
H	-8.1722212283	-1.5208074422	-2.2506902278
H	-9.1348197166	-0.5643464942	0.2988046787
H	-2.1439790723	0.848014737	0.6256443266
H	-2.4053261667	-0.8990154549	0.491822852
H	-5.6423772956	1.0512391368	1.8124111094
H	-6.905261146	-0.0890860976	2.2683998659
H	-5.2535500301	-1.8807392842	-3.8031518282
H	-4.1128469055	-0.5689766204	2.0054765282
H	-7.2024127898	2.6790404376	1.5122429221
H	-10.4593281525	2.8429357974	0.991369694
H	-10.2786310036	2.783351148	2.7402669367
H	-9.1408733628	3.7487787231	1.7717843696

calc_**2b** conf_8

C	-6.8140483469	-1.8719041619	-2.0699457526
C	-7.9100716628	-0.8359618986	-1.8090415256

C	-7.2052863147	0.2248985022	-0.9170043031
N	-6.0723885629	-0.4558073618	-0.310476889
C	-6.054464409	-1.8598509655	-0.7441081235
C	-4.7292551007	0.1262043057	-0.3862348935
C	-3.8592306253	-0.9591600352	-1.0445182002
C	-4.5755193572	-2.2760393636	-0.7326660147
O	-6.0703654552	-1.3505638038	-3.1684942797
O	-8.4912536095	-0.3186694263	-2.9729339626
C	-4.2469031852	0.5255874546	1.0162900559
C	-8.2010270651	0.7619449047	0.114585197
O	-4.1929092519	-2.8348998254	0.4953385675
O	-4.5013659371	-0.4765583482	1.9766760608
N	-7.7039473765	1.8325219591	0.953676968
O	-7.0981281501	0.5103161105	2.6970873652
C	-7.2300951928	1.6282382256	2.2074724286
C	-6.8882018917	2.8746358774	2.9944944493
H	-6.5963110203	-2.5016308517	-0.0356654757
H	-7.2255469427	-2.856815234	-2.3211496642
H	-8.713873944	-1.313685213	-1.2362900262
H	-6.8683798449	1.0494670132	-1.5625752253
H	-4.7412641891	1.0326508973	-1.0083132739
H	-3.8394604889	-0.7931367615	-2.1241901838
H	-2.8302582167	-0.9791146784	-0.6755756772
H	-4.3705221167	-3.0432811168	-1.4877812394
H	-5.5146897478	-2.0529284075	-3.5255050985
H	-7.7655394015	-0.2827019376	-3.6170781872
H	-4.7297163812	1.4648640939	1.3155947947
H	-3.1664208544	0.7026071353	1.0089685743
H	-8.4850709307	-0.0544808274	0.7831778232
H	-9.0989518541	1.1166021031	-0.4040557674
H	-4.2585412518	-2.1200761102	1.1641353564
H	-5.458915462	-0.4249134673	2.1662771209
H	-7.7931540715	2.7800557988	0.6242373149
H	-6.9570224252	3.7992732822	2.4159914381
H	-7.5665019493	2.94191558	3.8491670669
H	-5.8763974124	2.7733965522	3.3924331819

calc_**2b** conf_9

C	-6.8419608461	-1.7519588169	-1.7003288569
C	-7.6095191294	-1.3222091149	-0.4520827964
C	-6.6316442673	-0.3368318234	0.2197166153
N	-5.3056112009	-0.8546366028	-0.1065303266
C	-5.4289572112	-1.8995874968	-1.1454040614
C	-4.304209041	0.1152422968	-0.5405780482
C	-3.330630328	-0.7402914704	-1.3424060195
C	-4.2584818371	-1.6799067895	-2.1200706403
O	-6.885255592	-0.6986287687	-2.6371271063
O	-8.8296478343	-0.7459604196	-0.826732576
C	-3.649863154	0.8693617895	0.6069396111
C	-6.8655397304	-0.1721942315	1.7377107555
O	-3.6540923545	-2.8619687295	-2.5882045722
O	-4.5157155474	1.8103952136	1.231230852
N	-7.2969903757	1.1520901669	2.1280561492
O	-9.4046531663	0.8791606794	1.3504125619
C	-8.5447307781	1.5914598052	1.8748360647
C	-8.8599893054	3.0071316133	2.2954387775
H	-5.3358707667	-2.8981999758	-0.6901813378
H	-7.2256535971	-2.6850630604	-2.1347484117

H	-7.7616059048	-2.1958666001	0.2032647865
H	-6.7848923629	0.6434813179	-0.2573650617
H	-4.7575423397	0.8638166253	-1.222459716
H	-2.6726696059	-0.1571336895	-1.9910680507
H	-2.7026286471	-1.3274423589	-0.6578932142
H	-4.6305721176	-1.1726815993	-3.0122293622
H	-7.7750919487	-0.3197012655	-2.5286972851
H	-9.1528832125	-0.208171062	-0.0719070609
H	-2.7423975458	1.3745827535	0.245109863
H	-3.3578162969	0.1623316686	1.3869096884
H	-5.9340922327	-0.3834294786	2.2618209759
H	-7.6183547408	-0.8842430775	2.0893422847
H	-3.3186339771	-3.3419334191	-1.8191263365
H	-4.7625040039	2.4531905236	0.553396579
H	-6.5693900479	1.8036635034	2.3803854133
H	-8.0114678991	3.5290654409	2.743583857
H	-9.2124679606	3.5667148645	1.4257875301
H	-9.6799730928	2.9813515882	3.0171984754

calc_**2b** conf_10

C	-7.0754529885	-1.2147352621	-1.0376276931
C	-7.2430362325	0.3173168345	-0.9212526647
C	-6.253868544	0.68976625	0.1825084699
N	-5.1160207824	-0.2144672175	0.0026704978
C	-5.6595487891	-1.5112611741	-0.4675628199
C	-4.0415562319	0.2324965837	-0.9204567615
C	-4.0068352049	-0.8136261507	-2.044081202
C	-4.6061260824	-2.0811438697	-1.4330394941
O	-7.2046681717	-1.6241359295	-2.3964799566
O	-6.8323119124	0.9353324891	-2.1215214442
C	-2.748743967	0.329834308	-0.0969916079
C	-6.815391256	0.5037110195	1.5994705288
O	-3.6604230499	-2.8859932026	-0.7723658687
O	-2.6425145749	-0.7722038377	0.7850333367
N	-7.9602337561	1.333294016	1.9011582758
O	-9.4979444132	-0.2671562575	1.4442370645
C	-9.2361178046	0.8674415027	1.8202628833
C	-10.3187635682	1.8444519134	2.2235806288
H	-5.7558421244	-2.2069365673	0.3718234746
H	-7.8327711053	-1.7125720022	-0.4263100615
H	-8.276598067	0.5908272127	-0.6805312728
H	-5.9296865289	1.727723328	0.0573603354
H	-4.2791357779	1.2183791253	-1.3293652504
H	-4.6412008114	-0.4713035906	-2.8652754339
H	-3.0040037704	-0.9987759841	-2.4381290701
H	-5.0783976506	-2.714385569	-2.1847331809
H	-8.107583473	-1.9347538577	-2.5317801689
H	-6.9943583836	0.2661958184	-2.8096021792
H	-2.7451119681	1.2679541035	0.4775128864
H	-1.8649113396	0.3262858258	-0.7398571102
H	-6.0173408056	0.7156124252	2.3168549292
H	-7.1286264329	-0.5318918012	1.7504096676
H	-3.1159998636	-2.2982062199	-0.2123535049
H	-3.5360033095	-0.7945512562	1.1845543188
H	-7.8053882888	2.2836281812	2.1955521644
H	-10.902961988	1.4028182192	3.034247352
H	-9.9412856699	2.817582808	2.5470750461
H	-10.9952353116	1.9894477853	1.3780048855

calc_**2b** conf_11

```
C  -6.9327349157   -1.6318355617  -1.5156860139
C  -7.663895802    -0.8719537647  -0.4015862099
C  -6.5667123964   0.0474383173   0.1866153342
N  -5.2849011443   -0.5785470525  -0.1557008287
C  -5.5377598835   -1.8128365345  -0.9276086424
C  -4.3204804134   0.2471375229   -0.8987059991
C  -3.426365592    -0.8088648559  -1.5388380831
C  -4.3879587336   -1.9352645782  -1.9352978575
O  -6.8722344317   -0.8026571103  -2.6519541191
O  -8.7568053168   -0.1837030829  -0.9619480138
C  -3.5966208968   1.1672144873   0.0830315712
C  -6.6311891365   0.2025859572   1.7121996323
O  -3.8349240597   -3.2352789067  -1.8354673163
O  -3.1361238331   0.4400708908   1.1954465117
N  -7.9272566271   0.5407553638   2.290454103
O  -8.6872501278   1.9915439192   0.7168764062
C  -8.8087094179   1.4455732229   1.8151626385
C  -9.994978296    1.7471408251   2.7016631451
H  -5.5254000657   -2.6899693563  -0.2688664526
H  -7.4156609875   -2.5907614265  -1.7509480152
H  -8.0114129884   -1.5795078123  0.3669461691
H  -6.6457125849   1.0306907839   -0.2867667237
H  -4.8211877021   0.8484003367   -1.6729708333
H  -2.8525649778   -0.4119574385  -2.3813557881
H  -2.7284402697   -1.1933059646  -0.78804921
H  -4.7784510989   -1.7600558359  -2.9439643156
H  -7.7049043215   -0.2968426545  -2.6165763089
H  -8.8276404265   0.6840857032   -0.5159287182
H  -4.2787564391   1.9765212276   0.3930336823
H  -2.7366658831   1.6401375696   -0.4026833893
H  -5.8804940636   0.938314777    2.0250255557
H  -6.3398403923   -0.7571043625  2.1485375537
H  -3.1988480284   -3.3365278962  -2.5539139274
H  -3.7882660365   -0.2807084036  1.278002373
H  -8.1036891679   0.1754604407   3.2127880976
H  -10.0014150808  1.1955436231   3.6444587873
H  -10.0016610158  2.8184874683   2.9162026506
H  -10.910087445   1.5215801618   2.1493725546
```

calc_**2b** conf_12

```
C  -6.2288353468  -2.6407197421  -0.9094431273
C  -7.4923459948  -1.8561088308  -0.4740284837
C  -6.9766550827  -0.5289738008  0.1181374122
N  -5.5009457887  -0.5735803124  0.0191758276
C  -5.1416074405  -1.5765965195  -0.9831690269
C  -4.8510108532  0.6902059586   -0.409202261
C  -4.3855458016  0.4856961429   -1.86945276
C  -5.0667967956  -0.8092553397  -2.3066987513
O  -6.3987907168  -3.3280989243  -2.1274860754
O  -8.2691479958  -1.6588978173  -1.6479392507
C  -3.7063560855  0.993130783    0.562765182
C  -7.4411184768  -0.2865638874  1.5539667471
O  -4.3371662538  -1.4782299461  -3.3023481084
O  -4.1236801571  0.786137128    1.8979397988
N  -7.0895492806  1.0219071996   2.0487992853
```

O	-9.1869272014	1.8612989029	1.8113017928
C	-8.0120869837	2.0212233462	2.1125402723
C	-7.4868884297	3.352234719	2.6063130946
H	-4.1476250683	-1.9958214365	-0.7822380426
H	-5.9815992453	-3.3726848778	-0.1332406475
H	-8.0749625104	-2.4276811862	0.2586070961
H	-7.3853523718	0.2799530848	-0.5037266142
H	-5.5879808738	1.4957614081	-0.3318063643
H	-4.6282167092	1.3331860176	-2.5138740947
H	-3.3012912909	0.3303147497	-1.9175886154
H	-6.0893812896	-0.5906914635	-2.6517532454
H	-7.2319795837	-2.9885499263	-2.4988932283
H	-9.1654645152	-1.4066025059	-1.3959339919
H	-3.3814289036	2.0329300111	0.4741463386
H	-2.842527984	0.3517991193	0.3318606975
H	-7.0144517574	-1.0572798761	2.2078913661
H	-8.531227573	-0.3503625876	1.6075017687
H	-4.7887836324	-2.3292183799	-3.4131203095
H	-4.5046547739	-0.1114167428	1.8564659857
H	-6.1072605384	1.2236340761	2.2063960915
H	-7.7450554799	4.1247551458	1.8783246792
H	-7.9963080703	3.6047701398	3.5397896491
H	-6.407993144	3.3623961707	2.7780199136

calc_**2b** conf_13

C	-6.4317990895	-2.4728624896	-1.3430893019
C	-7.2681817166	-2.0852505268	-0.1040193035
C	-6.7688634939	-0.6952245648	0.2701901471
N	-5.3319947314	-0.7710034167	0.0033809379
C	-5.1630741509	-1.6132185022	-1.2059201508
C	-4.6444506205	0.5214507947	-0.254844523
C	-4.0388545013	0.4146632313	-1.6628326227
C	-4.9275848386	-0.6118537155	-2.3422523993
O	-7.1311565383	-2.1485729052	-2.5220471016
O	-8.6515699609	-2.1312097354	-0.3976875469
C	-3.6016828217	0.7865482783	0.8266725333
C	-7.1450917273	-0.3397457828	1.7073252415
O	-4.3245200845	-1.1645456362	-3.4837925682
O	-4.1483001649	0.6314222567	2.1227692239
N	-7.0769635203	1.0694598695	2.0050865882
O	-9.0888853047	1.4358836579	1.0419120651
C	-8.0871187306	1.8792802176	1.5946794863
C	-7.9106112481	3.3541987283	1.8731724611
H	-4.2663190344	-2.2364439677	-1.1095060372
H	-6.19176016	-3.5434450929	-1.3387640436
H	-7.0551274482	-2.7852589888	0.7138429878
H	-7.2678348381	0.03299804	-0.3943794986
H	-5.3896252737	1.3266812709	-0.2225003531
H	-4.013329401	1.3727580459	-2.1868297235
H	-3.0141847672	0.0249439946	-1.6228070395
H	-5.8882341086	-0.1493360662	-2.5992178747
H	-8.07142765	-2.2156519325	-2.2765368224
H	-9.0371968368	-1.2628985968	-0.2097140902
H	-3.2245573661	1.8105813339	0.7579073473
H	-2.7500143114	0.1046782588	0.689679643
H	-6.5082383102	-0.8908900027	2.4054490406
H	-8.1765499996	-0.6566334119	1.8857751497
H	-5.0123798151	-1.6844867095	-3.9216282574

```
H  -4.4655586445  -0.286043688   2.1232191987
H  -6.1767258224  1.4369191708   2.286936274
H  -7.9300194178  3.8998731023   0.9264303034
H  -8.7608124729  3.7023024674   2.4643159182
H  -6.986401078   3.5899330133   2.4056247112
```

calc_**2b** conf_14

```
C  -6.5354555209  -2.201367372   -1.8058537946
C  -7.653096493   -1.6705506776  -0.9046169271
C  -7.049179126   -0.4080581765  -0.2633299399
N  -5.5925724952  -0.6269720388  -0.2829308644
C  -5.2993759796  -1.9090434758  -0.9634931476
C  -4.765181638   0.4187163769   -0.9045093468
C  -3.4892872128  -0.3437116607  -1.2478411731
C  -3.9871422347  -1.7094206067  -1.7302743434
O  -6.5109719077  -1.4350994747  -2.9882873194
O  -8.8126476136  -1.3143844096  -1.6363259861
C  -4.5579447937  1.5558102771   0.0976390545
C  -7.6299844065  -0.1941175065  1.135193886
O  -3.1218129163  -2.7886734061  -1.4340638459
O  -4.3207483609  1.0257444878   1.3890109935
N  -7.2452342727  1.0441207846   1.7633333507
O  -9.3652723077  1.8454249232   1.9586713837
C  -8.1639906147  1.9766311675   2.1402530006
C  -7.5876822514  3.2045498921   2.8143174376
H  -5.1585262875  -2.7112001884  -0.2287680077
H  -6.6404235945  -3.2726040934  -2.032646164
H  -7.893745529   -2.406750877   -0.1252825988
H  -7.3124024958  0.4522401524   -0.8923373861
H  -5.2325105395  0.8132233244   -1.8194075919
H  -2.8779440286  0.1825180161   -1.9865601795
H  -2.8913044172  -0.4901088062  -0.3416318906
H  -4.2027198727  -1.6710255145  -2.8042163709
H  -7.4357892319  -1.1771385079  -3.1436016806
H  -9.379226348   -2.0920764249  -1.7151111554
H  -5.4473259932  2.2006558504   0.1130118753
H  -3.7033822868  2.1742998682   -0.1967531998
H  -7.33811939    -1.0438678722  1.7678974792
H  -8.7210968433  -0.1754306218  1.0678761239
H  -2.3569648494  -2.7203903063  -2.0183630018
H  -4.6162759445  0.0942926426   1.3020872059
H  -6.2615948467  1.1965353731   1.9620169209
H  -7.9330419482  4.0921808047   2.2792611576
H  -7.9841404632  3.2655749516   3.831107274
H  -6.4958849447  3.2114731254   2.857528772
```

calc_**2b** conf_15

```
C  -6.6562235621   -2.1598478158  -0.8617009442
C  -7.5046303845   -1.0018979589  -0.2770771102
C  -6.4874692415   0.0073042853   0.2978490161
N  -5.146376412    -0.5757396353  0.0867094263
C  -5.2569310051   -1.5683412673  -0.9825635252
C  -4.1027272345   0.3866112942   -0.3492596803
C  -3.8654201622   0.1404918869   -1.8572999085
C  -5.0029816103   -0.7870692232  -2.2756642599
O  -7.1522843264   -2.6255498416  -2.0971332729
O  -8.2485320153   -0.4585651359  -1.3410866205
```

```
C  -2.8569165216   0.1698128505   0.5079341216
C  -6.6333454182   0.2827657825   1.7922399411
O  -4.6501672854   -1.6038222445  -3.3624322014
O  -3.1913839551   0.1630711251   1.8747166881
N  -7.8846242848   0.8867762211   2.2421653496
O  -8.8848203882   1.6064164268   0.3099545434
C  -8.8519398902   1.5140469909   1.5364079944
C  -9.9550442048   2.1336444008   2.3679668708
H  -4.4762790822   -2.33385867    -0.8905919357
H  -6.6507881779   -2.9997574384  -0.1593453325
H  -8.1812724016   -1.3696665195  0.5068766926
H  -6.6020715664   0.943679068    -0.2578146451
H  -4.4701343524   1.400658308    -0.1566785354
H  -3.8342939861   1.0653564492   -2.437252603
H  -2.9185038127   -0.3874149317  -2.0223378655
H  -5.8993587873   -0.1924218734  -2.5093414151
H  -7.7970182557   -1.9513390676  -2.3782163352
H  -8.6649384672   0.3511795577   -0.9851460148
H  -2.1344141639   0.9751854478   0.34682491
H  -2.3721616664   -0.774960919   0.2129473543
H  -5.7962530126   0.9194407145   2.1041406543
H  -6.520253849    -0.6722518779  2.3195347302
H  -5.3957254062   -2.2171625577  -3.459143956
H  -3.9221339042   -0.4769925479  1.914055702
H  -7.9872224066   0.9200633318   3.2443164628
H  -9.9563161415   3.2118391988   2.1897997783
H  -10.9139898403  1.7465893405   2.016939634
H  -9.8680528174   1.9527268452   3.4417062918
```

calc_**2b** conf_16

```
C  -6.6697376016  -2.0736884741  -1.9458820629
C  -7.8009213761  -1.4103194651  -1.16207246
C  -7.0894279222  -0.2630190416  -0.405114541
N  -5.7175359884  -0.7596426616  -0.18382695
C  -5.5158419862  -2.0156658     -0.9455546505
C  -4.6000090755  0.1500848395   -0.4890385092
C  -3.4449538667  -0.8161576281  -0.7294260123
C  -4.0912686541  -1.9480901413  -1.5233673886
O  -6.4581675686  -1.2380035511  -3.0789140917
O  -8.8783508622  -1.006883446   -1.9509142021
C  -4.3717170855  1.1012499992   0.688986464
C  -7.8643871041  0.067323378    0.8710724843
O  -3.401255145   -3.1742248284  -1.5268795296
O  -4.543477751   0.4066356953   1.9093305331
N  -7.3754522639  1.1940078506   1.6256353724
O  -9.1442236488  2.5472113775   1.1661771947
C  -8.0711059014  2.3617280347   1.7189603672
C  -7.4173183805  3.4300928386   2.57162414
H  -5.5942695317  -2.8836676627  -0.2748405296
H  -6.9159527515  -3.0945592682  -2.2554445692
H  -8.1942229164  -2.1278198953  -0.4318571502
H  -7.0704251112  0.625247504    -1.052267703
H  -4.7961152998  0.7381447139   -1.3993374181
H  -2.6025842217  -0.3551442334  -1.2499906766
H  -3.0836352242  -1.1939548539  0.2364968889
H  -4.1488484207  -1.6522474419  -2.5741080828
H  -6.0913560639  -1.7701212489  -3.7939236853
H  -8.4794162453  -0.6195201096  -2.7459763901
```

```
H  -5.0751761417  1.9417514305   0.6272351553
H  -3.357193646   1.5122330331   0.6527887037
H  -7.8756052366  -0.824279189   1.511952069
H  -8.8950786302  0.3017292084   0.5917917239
H  -3.2371650478  -3.4227039461  -0.607266655
H  -5.0339729456  -0.3979245465  1.6337312849
H  -6.4863186224  1.0962321942   2.1024102175
H  -7.2944274023  4.33327246     1.9691094249
H  -8.0904330125  3.680853305    3.3952377336
H  -6.4476513468  3.1368395702   2.9814635004
```

calc_**2b** conf_17

```
C  -6.8973764431   -1.8138260195  -1.4150402913
C  -7.5575143158   -1.1394876066  -0.2083102055
C  -6.5651891263   -0.0172253797  0.1383803186
N  -5.2921746534   -0.7143697121  0.0043168385
C  -5.3997110454   -1.6577980766  -1.1284900132
C  -4.0775117463   0.100845579    -0.1691754151
C  -3.397305375    -0.4430296347  -1.4375120445
C  -4.5485099494   -1.0297517167  -2.237328164
O  -7.2528217396   -1.1272093695  -2.5974827265
O  -8.8460356872   -0.7369294857  -0.573416447
C  -3.1671793241   0.0130476992   1.0547097801
C  -6.7208187304   0.5773716635   1.5441361657
O  -4.088187993    -1.9382681912  -3.2094098124
O  -1.9992253316   0.7945110557   0.9149762484
N  -7.6881697871   1.6572241228   1.6481924453
O  -9.5293851777   0.3522555169   1.8195146814
C  -9.0130056137   1.471000764    1.8175759018
C  -9.8452977832   2.7153238167   2.0166943142
H  -4.9520393416   -2.6244331772  -0.86381733
H  -7.1913834564   -2.8686561467  -1.4934807069
H  -7.5781644488   -1.8466826868  0.6332799477
H  -6.6877460916   0.7945566772   -0.602034278
H  -4.345893606    1.1572463048   -0.3233983077
H  -2.8430343802   0.3212254278   -1.987294483
H  -2.7030634506   -1.2581949805  -1.1901587351
H  -5.1252704035   -0.222086644   -2.7024577046
H  -8.1760641606   -0.8562752072  -2.4610881879
H  -9.2937194524   -0.4341718394  0.2425715794
H  -2.9367462305   -1.0458119095  1.2469093448
H  -3.6768987619   0.3947653956   1.9433895505
H  -5.7635342831   0.9906386763   1.868451366
H  -6.9954012492   -0.208254067   2.2539431357
H  -4.8431632785   -2.1365716966  -3.7785583788
H  -1.4634530706   0.3944545745   0.218640044
H  -7.3410509583   2.6021967324   1.6222337388
H  -9.2646910072   3.6405284508   2.028153953
H  -10.5916958963  2.7715755537   1.2207616405
H  -10.3855666501  2.6222655362   2.9616222371
```

calc_**2b** conf_18

```
C  -6.5193844578   -2.2452481528  -1.4121000295
C  -7.3246183735   -1.6167199099  -0.2613871435
C  -6.5830381004   -0.3017443675  0.0271216544
N  -5.1894119617   -0.73795884    -0.0659525733
C  -5.1203183037   -1.6306653193  -1.2350467282
```

C	-4.1586648796	0.3111922895	-0.254127092
C	-3.6034360889	0.1311953597	-1.6848604803
C	-4.6728587557	-0.7122896603	-2.369543952
O	-7.1580658465	-1.8488063804	-2.6137770908
O	-8.6927277162	-1.4784766662	-0.5438618801
C	-3.0888159445	0.1531581824	0.823886134
C	-6.9410408259	0.3014459611	1.3793077037
O	-4.2748391659	-1.504183636	-3.4733589404
O	-3.6607124866	0.1298643426	2.1042286536
N	-8.319059446	0.7307514374	1.3870874675
O	-7.8946656941	2.821004362	2.1702795
C	-8.6866927402	1.9875622633	1.7581292112
C	-10.1678888164	2.2845600868	1.6412436143
H	-4.3529978891	-2.4014140367	-1.0952324383
H	-6.5000096774	-3.3386835337	-1.348490331
H	-7.2380113755	-2.2550525302	0.6245240128
H	-6.8507500755	0.4308137261	-0.7557881683
H	-4.6289827049	1.2929625874	-0.1302613222
H	-3.4170470029	1.0861213419	-2.1840756746
H	-2.6627052849	-0.4328372348	-1.6681956233
H	-5.515877777	-0.0694616386	-2.6552368457
H	-6.5468434567	-2.0293762424	-3.3437136188
H	-8.7468867583	-1.3075938291	-1.4982560661
H	-2.3882624152	0.9930943107	0.7934114531
H	-2.5144985621	-0.7670331064	0.6234622277
H	-6.3289954614	1.1753736223	1.6138275371
H	-6.7601178281	-0.4470598487	2.1643529884
H	-3.9893913322	-0.9150763743	-4.1833009915
H	-4.3435203652	-0.557280372	2.0471905789
H	-8.9937281007	0.1120090069	0.9588254651
H	-10.7491597651	1.4723372485	1.197807057
H	-10.5619934251	2.4966492601	2.6383536224
H	-10.297981139	3.1888662905	1.0425281086

calc_**2b** conf_19

C	-6.5376228404	-2.3211741446	-1.1438365085
C	-7.2694671874	-1.6569042886	0.0341846995
C	-6.5813133782	-0.2885605117	0.1591708615
N	-5.1778032953	-0.6622890648	-0.0208163011
C	-5.1531710417	-1.6492876833	-1.1128575473
C	-4.2245021638	0.418608267	-0.3760928268
C	-3.7552238443	0.1396570168	-1.8190586082
C	-4.8111330626	-0.8207991484	-2.355729405
O	-7.2917347873	-2.0084068957	-2.3016440822
O	-8.6629934223	-1.6112506865	-0.1254150958
C	-3.0785433337	0.4193738212	0.6334879937
C	-6.8602040692	0.4011146427	1.4887171516
O	-4.4860541083	-1.5899165759	-3.4936761819
O	-3.5590292937	0.4734947384	1.9494520598
N	-8.253907278	0.7631856983	1.5833231145
O	-7.8793427664	2.9236449188	2.1782450979
C	-8.6569734744	2.0252649416	1.8959354847
C	-10.1564518594	2.2419550899	1.8857645611
H	-4.3557950103	-2.3879758561	-0.9440737476
H	-6.4734443324	-3.4088593618	-1.0267072392
H	-7.0645895384	-2.2241350785	0.9485493825
H	-6.9515993499	0.3654419186	-0.6507223171
H	-4.7437759206	1.381096572	-0.3092511346

H	-3.6596429248	1.0455592711	-2.4210835501
H	-2.7746814538	-0.3576446274	-1.8113590642
H	-5.697442076	-0.2544794509	-2.6509599932
H	-6.7597479315	-2.2313278577	-3.0796437442
H	-8.8136951432	-1.4983924166	-1.0781203783
H	-2.4373330134	1.2929528875	0.4824937124
H	-2.4594191738	-0.4794216978	0.4695141717
H	-6.2793423645	1.3190308197	1.6044255582
H	-6.5750594428	-0.2740883994	2.3087650729
H	-3.6845042236	-2.0933569827	-3.2946942457
H	-4.201809444	-0.2518382703	1.9987339971
H	-8.927313096	0.0802421432	1.264828284
H	-10.72829391	1.3684705144	1.5629184884
H	-10.4765210311	2.5209479494	2.8927923133
H	-10.3845194134	3.0840677878	1.2284399662

calc_**2b** conf_20

C	-6.3794813935	-2.6002444532	-0.8847284408
C	-7.6003725815	-1.6779146461	-0.6965787158
C	-7.0509610335	-0.4304220964	0.0189739061
N	-5.5799448365	-0.5087785866	-0.0670944005
C	-5.2007675101	-1.6328828967	-0.9169304073
C	-4.8357453524	0.6678956483	-0.5502851016
C	-4.1876700278	0.2618280276	-1.8990533083
C	-4.902720711	-1.0233615309	-2.2909371357
O	-6.4676595955	-3.4009226116	-2.0419108641
O	-8.0870448375	-1.3982623173	-2.0055771783
C	-3.7518078179	1.1017542243	0.4249346208
C	-7.5234006635	-0.3497052436	1.4723685877
O	-4.1028376877	-1.8424653417	-3.1068871344
O	-4.335602489	1.6555412921	1.5951955491
N	-7.0903252461	0.8660182328	2.1092431712
O	-9.1806296387	1.7514554067	2.2717248065
C	-7.9691899991	1.8605606238	2.4088263153
C	-7.3364387525	3.1141002032	2.9781299101
H	-4.2788352342	-2.1135995906	-0.5615453369
H	-6.2975600231	-3.2638412618	-0.0178088596
H	-8.3880247909	-2.1687260567	-0.1122966248
H	-7.426230373	0.4607561472	-0.5040014159
H	-5.5295180928	1.5075702885	-0.6782371302
H	-4.2622665752	1.0476604373	-2.6538322264
H	-3.1238311484	0.0274589958	-1.767777415
H	-5.8522187314	-0.7786040622	-2.7883540953
H	-7.146803885	-2.9719710592	-2.590998037
H	-8.9738736613	-1.0256729653	-1.9339347564
H	-3.1088936377	1.8477380675	-0.0643913076
H	-3.1347040205	0.2276603563	0.6725995502
H	-7.1441413039	-1.2200065667	2.0227629754
H	-8.6155347942	-0.3543384596	1.520842375
H	-4.5940113503	-2.6750049876	-3.1813291518
H	-3.6459955488	1.7543117874	2.2620621893
H	-6.0935227992	1.063718807	2.1036894342
H	-7.3959795881	3.0831806866	4.0705664745
H	-6.2893983978	3.2276510293	2.6873932983
H	-7.9090558706	3.9788644722	2.6401758803

calc_**2b** conf_21

C	-6.8706444466	-1.7248768251	-0.912581398
C	-7.4262068504	-0.2911688396	-0.814738339
C	-6.34231908	0.4639527449	-0.0356811506
N	-5.1224715855	-0.369880122	-0.0849940288
C	-5.3611248419	-1.5403942838	-0.9261981074
C	-3.8691574193	0.2640230836	-0.5488353984
C	-3.4806594784	-0.4363243751	-1.8676987246
C	-4.7322549568	-1.1996537071	-2.2876667423
O	-7.2932174505	-2.3797185678	-2.1060849646
O	-7.552289813	0.2701753662	-2.1060235496
C	-2.7995286173	0.117398459	0.5354037977
C	-6.7104249603	0.7357890822	1.4283807106
O	-4.4067481983	-2.3312021848	-3.0589861322
O	-3.2902322626	0.5081947542	1.794244286
N	-7.8682472177	1.584726563	1.5994114682
O	-9.3261847218	-0.1088730976	1.9830512484
C	-9.1011118425	1.090616046	1.8947619344
C	-10.186061469	2.1226504414	2.1143519931
H	-4.8449732146	-2.4279335242	-0.5371482486
H	-7.1746638272	-2.2998179976	-0.0313286827
H	-8.3897946251	-0.2715058208	-0.2932018971
H	-6.1822014467	1.4235653708	-0.544388025
H	-4.0579218615	1.3330696431	-0.7005440607
H	-3.1383658986	0.2633828507	-2.6329779363
H	-2.6785220586	-1.1653761272	-1.7009575632
H	-5.4058043996	-0.5281527348	-2.8338375474
H	-8.1038601451	-2.868087256	-1.9176292767
H	-7.7648983955	-0.4769617934	-2.6891710608
H	-1.9357565319	0.7494416135	0.3087537806
H	-2.4494360133	-0.9273397443	0.5571578566
H	-5.8509778466	1.2027710543	1.9189118664
H	-6.9247099344	-0.2072244599	1.9368132912
H	-5.2486026777	-2.7666429181	-3.2522281555
H	-4.0933183405	-0.0275785633	1.8974641352
H	-7.7491946582	2.5819200755	1.5264607781
H	-9.858347648	3.153536683	1.9592698709
H	-11.0166419829	1.9076804309	1.4384631917
H	-10.5631232821	2.0208186798	3.1350007805

calc_**2b** conf_22

C	-6.5032116316	-2.2800054016	-1.3684617081
C	-7.3503508264	-1.5923495516	-0.2722166264
C	-6.5894387489	-0.2959449513	0.0320191442
N	-5.2014731115	-0.7446006439	-0.0509705228
C	-5.1225352319	-1.6345630377	-1.2229468285
C	-4.1734096545	0.3091559054	-0.242980208
C	-3.6450182422	0.1552741651	-1.6834385473
C	-4.6863838117	-0.7133807731	-2.3739678753
O	-7.0349312952	-1.9959045234	-2.66269525
O	-8.6973452509	-1.4176448643	-0.6443561515
C	-3.0905736323	0.1348181037	0.8189738727
C	-6.9488511985	0.3080536055	1.3844777547
O	-4.1546491673	-1.3924365086	-3.4827238604
O	-3.6487874712	0.0857670232	2.1060683457
N	-8.3210483932	0.7557716199	1.3937765448
O	-7.8662125665	2.8544231442	2.1361416823
C	-8.6720226888	2.0232928203	1.7493075529
C	-10.1515613091	2.3333158264	1.6468725064

H	-4.3374808706	-2.386941056	-1.084630703
H	-6.4649863824	-3.3641475057	-1.2242846513
H	-7.3571618902	-2.2162139905	0.6266827474
H	-6.8387726754	0.4483414306	-0.7481867167
H	-4.6444797711	1.2866343949	-0.0918766366
H	-3.4940768747	1.1131743046	-2.1853313442
H	-2.6881162354	-0.3807761071	-1.6936172603
H	-5.53811424	-0.0910213445	-2.6783857793
H	-7.73516152	-2.6399281517	-2.8295721546
H	-8.6687191945	-0.992796808	-1.5157243833
H	-2.3934768912	0.9775914518	0.7952811168
H	-2.514807927	-0.7790816856	0.5960258394
H	-6.3266169833	1.1738474311	1.6211868796
H	-6.7771912456	-0.4440550623	2.1675760434
H	-4.9064677946	-1.8277960641	-3.9071874798
H	-4.3350316731	-0.5970147041	2.0373125808
H	-9.0133786009	0.1360676714	0.9985782918
H	-10.5302312179	2.5654733114	2.6454422453
H	-10.2803728667	3.2291943256	1.0354356544
H	-10.7475509143	1.5204062002	1.2243958846

calc_**2b** conf_23

C	-7.110479108	-1.6175104069	-0.6852964058
C	-7.6806549415	-0.2923643254	-1.2283825977
C	-6.6760279708	0.7754059218	-0.7595747616
N	-5.5031085986	0.0492539702	-0.2447633925
C	-5.6153864679	-1.366532706	-0.5794804163
C	-4.1710312822	0.5057906493	-0.6944728682
C	-3.6046032974	-0.6122811679	-1.5956937732
C	-4.7923080351	-1.535289694	-1.8660100482
O	-7.3581876171	-2.7072888862	-1.5733173893
O	-7.6834885151	-0.3144143729	-2.6441164481
C	-3.3233775988	0.7922658318	0.554018119
C	-7.2007505581	1.8355908909	0.2331283091
O	-4.3674832754	-2.8564932821	-2.1007737886
O	-4.1067358481	1.4559042167	1.5158025504
N	-7.1825519443	1.4917141709	1.6413543782
O	-8.8254690331	-0.0647026992	1.5660368509
C	-8.0219269047	0.594688306	2.214090979
C	-7.9093547494	0.4565402428	3.7174959303
H	-5.1550758455	-1.9962500719	0.1934268079
H	-7.537559593	-1.8151108822	0.3006359247
H	-8.6867622372	-0.1014118297	-0.8428418419
H	-6.4190939282	1.3385470238	-1.6676637822
H	-4.2986798386	1.4440497366	-1.2460058414
H	-3.1543102705	-0.2293785883	-2.5139069605
H	-2.8387811179	-1.1922412176	-1.0670944533
H	-5.3749665607	-1.1510283324	-2.7132626487
H	-8.1670464809	-3.1509380685	-1.2909052917
H	-7.7988989921	-1.2495227159	-2.8817572758
H	-2.4658632179	1.4262012743	0.3098837466
H	-2.9314599131	-0.1573357204	0.9541727286
H	-8.2165522764	2.1185683191	-0.0649709696
H	-6.5716001227	2.7238312173	0.1421080731
H	-5.1768325918	-3.372916961	-2.2201951872
H	-4.9326855274	0.9323314928	1.4834353282
H	-6.5758884559	2.0317196823	2.2377757141
H	-7.0987693051	1.0416058622	4.1581335226

```
H  -8.8557554209  0.7642654697   4.1695381973
H  -7.7604925585  -0.5972623498  3.9624489819
```

calc_**2b** conf_24

```
C  -6.6444574678  -2.184341141   -1.5439792899
C  -7.7928595229  -1.1185926758  -1.4549436698
C  -7.3553356382  -0.1617549613  -0.3157641831
N  -5.9426902933  -0.4177609088  -0.0656946333
C  -5.7566591962  -1.8507396361  -0.33097286
C  -5.0211422642  0.287580397    -0.9839211187
C  -3.7975846082  -0.6373112487  -1.0594031117
C  -4.2428899206  -1.9986582863  -0.4864316395
O  -5.9317006264  -2.0470110343  -2.7470612587
O  -7.9711114456  -0.4793417766  -2.7120528839
C  -4.7097950079  1.7055582195   -0.5238291728
C  -8.2094961868  -0.4101778553  0.9423620856
O  -3.635236822   -2.2804181905  0.7633866897
O  -5.7865411953  2.5958040036   -0.703537772
N  -7.6466964902  0.1091651217   2.1708914593
O  -7.5528050665  2.2580502278   1.4806799047
C  -7.3141297762  1.4064011284   2.3357093169
C  -6.6406337932  1.7639015378   3.6403802555
H  -6.0913547113  -2.4444337333  0.5290134776
H  -7.0516408249  -3.2025030179  -1.4681548209
H  -8.7477565978  -1.6044467593  -1.227923814
H  -7.4886439553  0.8811861311   -0.6059830586
H  -5.4572319267  0.3700045766   -1.9904684662
H  -3.4491321485  -0.7294912364  -2.0891319789
H  -2.961877657   -0.2628374861  -0.457386619
H  -3.9771819182  -2.8304550254  -1.1388352875
H  -6.5287609583  -1.5477445166  -3.3345378499
H  -7.5811836235  0.4030331147   -2.6631745497
H  -3.877710357   2.0879738174   -1.126618764
H  -4.3710758003  1.6737124163   0.5228812824
H  -8.3571844808  -1.4823201379  1.0997726838
H  -9.1986939395  0.029945397    0.7780981128
H  -3.8839912593  -1.5521111049  1.3495889664
H  -6.3551753718  2.5694336504   0.0867969677
H  -7.3118668615  -0.5539209268  2.8499126183
H  -5.6168630144  2.0866238198   3.4328119476
H  -7.1658617496  2.6135602514   4.0810030993
H  -6.6140475228  0.9454378489   4.3635179347
```

Table S38. Sampled and DFT geometry optimized conformers related to calc_**2c**, with energy values (Hartree) and related % contribution on the final Boltzmann distribution for the three employed functional/basis set combinations.

Conformer	MPW1PW91/6-31g(d,p)		MPW1PW91/6-311+g(d,p)		B97-2/cc-pVTZ	
	Energy (Hartree)	% contribution on the Boltzmann distribution	Energy (Hartree)	% contribution on the Boltzmann distribution	Energy (Hartree)	% contribution on the Boltzmann distribution
calc_**2c** conf_1	-916.6593144	93.88%	-916.8940084	88.54%	-916.8411975	73.40%
calc_**2c** conf_2	-916.6561823	3.40%	-916.8918926	9.42%	-916.8399559	19.71%
calc_**2c** conf_3	-916.6553274	1.38%	-916.8898102	1.04%	-916.8382855	3.36%
calc_**2c** conf_4	-916.6552247	1.23%	-916.8894586	0.71%	-916.8381521	2.92%
calc_**2c** conf_5	-916.6523443	0.06%	-916.8866502	0.04%	-916.8339035	0.03%
calc_**2c** conf_6	-916.6511297	0.02%	-916.8883109	0.21%	-916.8362275	0.38%
calc_**2c** conf_7	-916.6510022	0.01%	-916.8856609	0.01%	-916.8346381	0.07%
calc_**2c** conf_8	-916.6507502	0.01%	-916.8850608	0.01%	-916.8344792	0.06%
calc_**2c** conf_9	-916.6496278	0.00%	-916.8839994	0.00%	-916.8329005	0.01%
calc_**2c** conf_10	-916.6487700	0.00%	-916.8857021	0.01%	-916.8337733	0.03%
calc_**2c** conf_11	-916.6487010	0.00%	-916.8832926	0.00%	-916.8332605	0.02%
calc_**2c** conf_12	-916.6468637	0.00%	-916.8815696	0.00%	-916.8310224	0.00%
calc_**2c** conf_13	-916.6457891	0.00%	-916.8846577	0.00%	-916.8328106	0.01%
calc_**2c** conf_14	-916.6445246	0.00%	-916.8827466	0.00%	-916.8317578	0.00%
calc_**2c** conf_15	-916.6441171	0.00%	-916.8810170	0.00%	-916.8291855	0.00%
calc_**2c** conf_16	-916.6436037	0.00%	-916.8797328	0.00%	-916.8289386	0.00%
calc_**2c** conf_17	-916.6434575	0.00%	-916.8820410	0.00%	-916.8310253	0.00%
calc_**2c** conf_18	-916.6429565	0.00%	-916.8777842	0.00%	-916.8273620	0.00%
calc_**2c** conf_19	-916.6428849	0.00%	-916.8786364	0.00%	-916.8278920	0.00%
calc_**2c** conf_20	-916.6425687	0.00%	-916.8787206	0.00%	-916.8274909	0.00%
calc_**2c** conf_21	-916.6424392	0.00%	-916.8778239	0.00%	-916.8258961	0.00%
calc_**2c** conf_22	-916.6423425	0.00%	-916.8785005	0.00%	-916.8281990	0.00%
calc_**2c** conf_23	-916.6422630	0.00%	-916.8760118	0.00%	-916.8255323	0.00%
calc_**2c** conf_24	-916.6422249	0.00%	-916.8773204	0.00%	-916.8270534	0.00%
calc_**2c** conf_25	-916.6421984	0.00%	-916.8772600	0.00%	-916.8268987	0.00%
calc_**2c** conf_26	-916.6421715	0.00%	-916.8781638	0.00%	-916.8280307	0.00%
calc_**2c** conf_27	-916.6420646	0.00%	-916.8772723	0.00%	-916.8268253	0.00%
calc_**2c** conf_28	-916.6419182	0.00%	-916.8790238	0.00%	-916.8283886	0.00%

calc_**2c** conf_29	-916.6414649	0.00%	-916.8763347	0.00%	-916.8263286	0.00%
calc_**2c** conf_30	-916.6413011	0.00%	-916.8762497	0.00%	-916.8250859	0.00%
calc_**2c** conf_31	-916.6409741	0.00%	-916.8755981	0.00%	-916.8241265	0.00%
calc_**2c** conf_32	-916.6408285	0.00%	-916.8778290	0.00%	-916.8273658	0.00%
calc_**2c** conf_33	-916.6407598	0.00%	-916.8769028	0.00%	-916.8266625	0.00%
calc_**2c** conf_34	-916.6401777	0.00%	-916.8778566	0.00%	-916.8273923	0.00%
calc_**2c** conf_35	-916.6401356	0.00%	-916.8744093	0.00%	-916.8230158	0.00%
calc_**2c** conf_36	-916.6398480	0.00%	-916.8748831	0.00%	-916.8243886	0.00%
calc_**2c** conf_37	-916.6397383	0.00%	-916.8757712	0.00%	-916.8254365	0.00%
calc_**2c** conf_38	-916.6396444	0.00%	-916.8767428	0.00%	-916.8261981	0.00%
calc_**2c** conf_39	-916.6392437	0.00%	-916.8793164	0.00%	-916.8282140	0.00%
calc_**2c** conf_40	-916.6392215	0.00%	-916.8762287	0.00%	-916.8262948	0.00%
calc_**2c** conf_41	-916.6387064	0.00%	-916.8780046	0.00%	-916.8274347	0.00%
calc_**2c** conf_42	-916.6386646	0.00%	-916.8756762	0.00%	-916.8253078	0.00%
calc_**2c** conf_43	-916.6382805	0.00%	-916.8747320	0.00%	-916.8248691	0.00%
calc_**2c** conf_44	-916.6374710	0.00%	-916.8750000	0.00%	-916.8236343	0.00%
calc_**2c** conf_45	-916.6373683	0.00%	-916.8729987	0.00%	-916.8230565	0.00%
calc_**2c** conf_46	-916.6368994	0.00%	-916.8726268	0.00%	-916.8215026	0.00%
calc_**2c** conf_47	-916.6364445	0.00%	-916.8725305	0.00%	-916.8229899	0.00%
calc_**2c** conf_48	-916.6363982	0.00%	-916.8750007	0.00%	-916.8236258	0.00%
calc_**2c** conf_49	-916.6348422	0.00%	-916.8716995	0.00%	-916.8210615	0.00%
calc_**2c** conf_50	-916.6347653	0.00%	-916.8732921	0.00%	-916.8227527	0.00%
calc_**2c** conf_51	-916.6329818	0.00%	-916.8725001	0.00%	-916.8220643	0.00%
calc_**2c** conf_52	-916.6306019	0.00%	-916.8703707	0.00%	-916.8204293	0.00%

Table S39. Cartesian coordinates of the optimized geometries for the conformers related to calc_**2c**. The related energies and % contribution on the final Boltzmann distribution for the three employed functional/basis set combinations are reported in Table S38.

calc_**2c** conf_1

C	-6.8736049234	-2.3148339446	-4.1837253314
C	-8.0757480558	-1.9796885431	-3.2814986425
C	-8.190143612	-3.2301994749	-2.3993650546
N	-8.0247543195	-4.2884271867	-3.3833464889
C	-6.9897673352	-3.8416360554	-4.3431241493
C	-7.7611583704	-5.6992180736	-3.0309292397
C	-6.455190287	-6.0594485708	-3.7924294181
C	-5.770681964	-4.7193089385	-4.0199086973
O	-5.6998886066	-1.8453259514	-3.5510546216
O	-7.8915021957	-0.7857121277	-2.5736938295
C	-7.6657274605	-6.0556036914	-1.5468125968
C	-9.503476252	-3.25968671	-1.6178102405
O	-5.077914424	-4.2903146342	-2.8543984408
O	-6.6206345504	-5.3765095523	-0.8716686891
N	-9.4298184646	-2.3751636908	-0.4695926922
O	-8.2779043008	-3.8756751952	0.7843221316
C	-8.7411927982	-2.741802114	0.6338883656
C	-8.5916844641	-1.694506271	1.7096425452
H	-7.30850659	-4.0806830652	-5.3675563628
H	-6.9394893521	-1.8030930557	-5.148106047
H	-8.9885357742	-1.8903336579	-3.8826456665
H	-7.3724835236	-3.1877027572	-1.6639354509
H	-8.5799048134	-6.3209946135	-3.4210584089
H	-6.6915622235	-6.5205945177	-4.7560172733
H	-5.8153456349	-6.7508310333	-3.2372583721
H	-5.0399144243	-4.7401375829	-4.8323353752
H	-5.2859795765	-2.6151897148	-3.0994390807
H	-6.9191197143	-0.7165804525	-2.5050440189
H	-7.4769089273	-7.135360732	-1.4908795786
H	-8.6106513084	-5.8753183863	-1.029428366
H	-10.336216103	-2.9425174877	-2.2518454058
H	-9.7385151673	-4.2562731906	-1.246240565
H	-5.5496234312	-4.6419091476	-2.0620111652
H	-7.0492278493	-4.7928709975	-0.2124764999
H	-9.5254658639	-1.3869793366	-0.6533718704
H	-9.1092737222	-0.7598832099	1.4845825177
H	-8.9779995757	-2.1011884061	2.6469243632
H	-7.5284840407	-1.4894979295	1.8586477163

calc_**2c** conf_2

C	-6.6615626247	-2.4177208805	-3.8469617348
C	-7.7869842054	-2.1179710375	-2.8491860751
C	-8.0074381884	-3.4643641146	-2.1334346733
N	-7.8660398941	-4.4012607085	-3.2354556211
C	-6.946328571	-3.8691123963	-4.25126095
C	-7.6243864916	-5.833053238	-3.0236081316
C	-6.5827508483	-6.1887182062	-4.1138315938
C	-5.8080013813	-4.8977559239	-4.3499462165
O	-5.4131241098	-2.2551023349	-3.1898771438
O	-7.3682233932	-1.0595921069	-2.0368620115
C	-7.2070935217	-6.2534341728	-1.6072569973
C	-9.389985811	-3.5873189563	-1.4583106875

O	-4.8297510425	-4.7865984028	-3.3302963895
O	-6.0796282003	-5.6187499325	-1.0775978645
N	-9.3754177649	-3.3780793508	-0.0220789377
O	-9.2785639815	-1.1193327946	-0.1177791882
C	-9.3134818938	-2.157817928	0.5464368395
C	-9.3084715099	-2.1160060869	2.0549739907
H	-7.442808665	-3.857314269	-5.2342567872
H	-6.6916188295	-1.7435547814	-4.7103167895
H	-8.700677055	-1.8640981769	-3.4073059702
H	-7.2327803519	-3.5939934244	-1.3623961145
H	-8.5454972225	-6.4016448496	-3.2261231843
H	-7.0914296315	-6.4871236786	-5.0359413787
H	-5.917028568	-7.0040615052	-3.8179867959
H	-5.322933716	-4.8722575888	-5.3338001085
H	-5.5997054502	-1.6448622095	-2.4505986811
H	-8.0388911339	-0.9413149697	-1.3282733647
H	-7.0857255709	-7.3508704333	-1.6318942172
H	-8.0383976207	-6.0612724759	-0.9154469179
H	-10.0973158739	-2.8773116061	-1.8970996967
H	-9.7902841087	-4.5879145692	-1.6317851489
H	-4.7265516445	-3.8281073963	-3.1495295389
H	-5.4740091105	-5.4149873476	-1.8227566018
H	-9.2940619176	-4.1866077821	0.5733816936
H	-8.3754652822	-1.6572614286	2.3914850759
H	-10.1255243044	-1.4716812266	2.3869615877
H	-9.4130605098	-3.096771709	2.5240163248

calc_**2c** conf_3

C	-6.4171740365	-2.5713102188	-3.8546736011
C	-7.3414695056	-1.9677100613	-2.7971445632
C	-7.8724773911	-3.2143941952	-2.0563197843
N	-7.9794591984	-4.199324267	-3.1148684624
C	-7.2444422302	-3.7696978872	-4.3158569461
C	-7.8304982262	-5.6314769017	-2.8742965
C	-7.4047617742	-6.1469680963	-4.2619624979
C	-6.5517837223	-5.0306052315	-4.8778894398
O	-5.2314704002	-2.9344040315	-3.1601259803
O	-6.7203150225	-1.0227228231	-1.9633344374
C	-6.8633713729	-6.0013254896	-1.744910966
C	-9.1895672477	-2.9640946864	-1.3272079231
O	-5.1705676468	-5.1568909246	-4.5651638915
O	-5.5330650574	-5.5480155265	-1.9456042685
N	-9.0036622484	-2.0035906325	-0.2640172949
O	-10.1841578334	-3.1727125029	1.2827113614
C	-9.4983898726	-2.2053688559	0.9874143921
C	-9.1490207227	-1.1353117166	2.0009637516
H	-7.9520082131	-3.4075015795	-5.0789659192
H	-6.1839060666	-1.8705219622	-4.6649921198
H	-8.1740758551	-1.4544719709	-3.2907907067
H	-7.124479443	-3.4768761505	-1.2868999652
H	-8.7976747085	-6.0741542524	-2.5951783522
H	-8.2986717538	-6.2932927533	-4.8756182223
H	-6.8637593916	-7.0968175256	-4.2286089379
H	-6.6024070395	-5.0366478459	-5.9688614462
H	-4.7774234529	-3.5682702631	-3.7547726985
H	-5.835688226	-1.3763671382	-1.7791276505
H	-6.8069732938	-7.0903223685	-1.6658119686
H	-7.2460193334	-5.6306483114	-0.7853513076

H -9.9421489928   -2.6241904457 -2.0514286698
H -9.5716790181   -3.8781247405 -0.8659384647
H -5.122478791   -5.5665205783 -3.6721152326
H -5.5362808814   -4.5726952781 -1.9718661142
H -8.3703122835   -1.2358129512 -0.4388120126
H -10.0743121811 -0.6822830508 2.3653006175
H -8.6615401525   -1.6083772656 2.856592208
H -8.500507413   -0.3481795197 1.6085340148

calc_**2c** conf_4

C -6.727583951   -2.4814269701 -3.9696822434
C -7.8267375142 -1.9397592954 -3.0531785659
C -8.1711603647 -3.1685744001 -2.1875653217
N -8.0378437597 -4.2715700928 -3.1182549064
C -7.2855291847 -3.8615109558 -4.3160482416
C -7.659398608   -5.6127429175 -2.6774352421
C -7.0274891003 -6.2034759196 -3.9519222941
C -6.3340484302 -5.0303464966 -4.6562699263
O -5.5554203207 -2.5283563148 -3.1682011646
O -7.4574989378 -0.7998889843 -2.3240412841
C -6.750568236   -5.6584507914 -1.4448263127
C -9.549041133   -3.1025004804 -1.5273332808
O -4.9852284111 -4.8599081587 -4.2398420132
O -5.4992403403 -5.0059981602 -1.6253608684
N -9.5773950642 -2.1589162234 -0.4317346773
O -9.1123720865 -3.7437874779 1.1283228835
C -9.2685526501 -2.5669618432 0.8328107557
C -9.159601544   -1.4654229908 1.8646922106
H -7.9754025622 -3.7345120077 -5.1658849659
H -6.5607540931 -1.8511391374 -4.8515875392
H -8.6985298519 -1.6618042088 -3.6565142035
H -7.4380036266 -3.2026667994 -1.3633115649
H -8.5551599987 -6.1903191524 -2.4067100039
H -7.8221310338 -6.5886803237 -4.5980581611
H -6.3313140471 -7.0233758779 -3.7540736672
H -6.2859803333 -5.1777018734 -5.7375359577
H -4.9564147461 -3.1470140666 -3.6373799492
H -6.5406328535 -0.9539868027 -2.0471407587
H -6.5178742146 -6.7016277089 -1.2143289303
H -7.2720695004 -5.2432846266 -0.5735892357
H -10.312244986 -2.8386092587 -2.2670495892
H -9.8065360064 -4.0763335602 -1.1074241191
H -4.9477148891 -5.140477452   -3.2967211464
H -5.6624888044 -4.0542794372 -1.7583447168
H -9.441438475   -1.1852180896 -0.6598585053
H -9.3881933072 -0.4712986424 1.4735533421
H -9.8425220484 -1.690057113   2.6870110904
H -8.1468849855 -1.4640153884 2.2758190747

calc_**2c** conf_5

C -7.2014432377   -2.3144769854 -4.3652207606
C -7.4757635893   -1.9542237828 -2.8875337085
C -8.0850199733   -3.2357690556 -2.2790763789
N -8.0479861437   -4.262892433   -3.3355896027
C -7.0783658451   -3.8365828716 -4.3456989877
C -7.7063338365   -5.6662701518 -3.0037095268
C -6.3094531717   -5.9233142949 -3.6513585756

C	-5.7711775291	-4.5210835566	-3.9157551686
O	-6.1002683798	-1.6291648557	-4.8871965076
O	-6.2766122128	-1.5492446564	-2.2618367545
C	-7.7298968413	-6.0520956591	-1.5292524786
C	-9.507849986	-3.0628479267	-1.7428920204
O	-5.2195575532	-3.9815303963	-2.7137518143
O	-6.6651518125	-5.466949656	-0.7878576682
N	-9.5276452754	-2.3158154602	-0.4961404674
O	-8.5139110617	-3.9360863252	0.7225487162
C	-8.9980858763	-2.8055966327	0.646046492
C	-9.0347501884	-1.8912553013	1.8472145488
H	-7.3444649052	-4.2518550825	-5.328300087
H	-8.0847346457	-2.0662568368	-4.9663731555
H	-8.1510936298	-1.097433236	-2.8039686463
H	-7.4623335785	-3.4936205621	-1.423909764
H	-8.4472590364	-6.316509555	-3.4880161741
H	-6.4185194787	-6.4667952001	-4.5941365262
H	-5.6395989257	-6.502780611	-3.0094310407
H	-4.9937611105	-4.478313657	-4.681695193
H	-5.6445581137	-1.2740622389	-4.1021075052
H	-5.7015398399	-2.3495493777	-2.2765758821
H	-7.6302296408	-7.1432845077	-1.4772864226
H	-8.6852232758	-5.7899286835	-1.0669694032
H	-10.1410770269	-2.5422325252	-2.4662646563
H	-9.9563179436	-4.0441601285	-1.5690591012
H	-5.5718571227	-4.5057927065	-1.9618449685
H	-7.0864888815	-4.9290692347	-0.09162718
H	-9.8470897287	-1.36125629	-0.5048515372
H	-9.5960561072	-0.9688588425	1.6823902729
H	-9.4746364227	-2.4343836627	2.6860621335
H	-8.008888072	-1.6356570603	2.1250255

calc_**2c** conf_6

C	-6.7767353238	-2.3396637531	-3.9914167253
C	-7.9638045582	-2.0062621222	-3.0937844449
C	-8.1095145358	-3.2830266789	-2.2569143141
N	-7.8201457853	-4.3586634489	-3.2028261332
C	-7.038079973	-3.8044416274	-4.3358752037
C	-7.1099626071	-5.561686799	-2.7248062181
C	-5.6465705221	-5.3484281546	-3.1124565167
C	-5.7672472926	-4.6849827758	-4.4871164178
O	-5.5973984383	-2.1983746997	-3.2149565419
O	-7.6720197846	-0.8660581169	-2.3299479179
C	-7.2799013947	-5.9213616322	-1.25901736
C	-9.4977949914	-3.3994066749	-1.5917708365
O	-4.6309585242	-4.0096680316	-4.948697762
O	-8.6143372063	-6.2033781635	-0.8640786819
N	-9.443249675	-3.5310107659	-0.1531736686
O	-8.8561389405	-1.3674804656	0.1484336295
C	-9.0859468062	-2.4859675356	0.617761554
C	-9.0026618436	-2.7365945845	2.1037468513
H	-7.633018685	-3.8372564407	-5.2575107943
H	-6.7192669144	-1.7062686167	-4.8847703526
H	-8.8649788304	-1.8582238751	-3.7088104512
H	-7.352771309	-3.2061548493	-1.4612018806
H	-7.4944601517	-6.4169059335	-3.3086910317
H	-5.0586087938	-6.2700058767	-3.1403695334
H	-5.1721013691	-4.646457343	-2.4171374494

H	-5.9659699941	-5.4614367768	-5.2341069954
H	-5.7922704151	-1.4492109057	-2.6203816899
H	-8.1318846965	-0.9446311677	-1.4647125906
H	-6.9492102769	-5.1044889172	-0.6135742172
H	-6.6283598458	-6.780880172	-1.047508782
H	-10.1002148055	-2.5145936381	-1.8199859515
H	-10.0113474192	-4.2718164874	-1.9920449107
H	-4.4948541013	-3.2656560769	-4.3382228222
H	-8.9645894977	-6.8779443869	-1.4598026793
H	-9.4454293914	-4.472037975	0.2145877336
H	-9.2181850473	-3.7701881437	2.3830760681
H	-8.0034576103	-2.4676933428	2.4542723443
H	-9.7115526429	-2.0766930443	2.6097926936

calc_**2c** conf_7

C	-6.5099237508	-2.5215859594	-4.1339453034
C	-6.8936606868	-1.5646215217	-3.0036938247
C	-7.3981490681	-2.5240346956	-1.8999149688
N	-7.9931977978	-3.6095990138	-2.6506825665
C	-7.653507676	-3.5289520035	-4.075643792
C	-8.079557692	-4.9846528566	-2.1807299773
C	-8.2479739762	-5.7621857558	-3.5001612266
C	-7.446160496	-4.9837264375	-4.5505443006
O	-5.2574455824	-3.0768008419	-3.7509636706
O	-5.8547628831	-0.7220319021	-2.5930547417
C	-6.8865763873	-5.4399961433	-1.3353897641
C	-8.3517945822	-1.8188235147	-0.9321789052
O	-6.0897742085	-5.4084917951	-4.6340213839
O	-5.6336211924	-5.342617371	-1.9971848522
N	-8.5455771331	-2.5098665304	0.3269015061
O	-10.5506276177	-3.4432866045	-0.1910897465
C	-9.6577159425	-3.2418293851	0.6166947476
C	-9.7177562985	-3.7983110317	2.0254518222
H	-8.4971247129	-3.1069008661	-4.647155703
H	-6.4209395337	-2.0178766142	-5.1037188231
H	-7.7177521969	-0.9218195445	-3.333764185
H	-6.5110314944	-2.8348767569	-1.3179475105
H	-8.9729619108	-5.1145906964	-1.5555053363
H	-9.30540617	-5.7547946397	-3.7804810005
H	-7.9261648503	-6.8057921056	-3.4381265753
H	-7.8527029243	-5.1185957881	-5.5552849316
H	-5.1624557661	-3.8786304992	-4.308041526
H	-5.0514328484	-1.2643612889	-2.6323778527
H	-7.0114079363	-6.4931606087	-1.068627945
H	-6.8594699156	-4.8705131173	-0.397127579
H	-7.9527071108	-0.8211766429	-0.7171765197
H	-9.3320263736	-1.7192262554	-1.4032608551
H	-5.8320087742	-5.7013213715	-3.7312028762
H	-5.4860230665	-4.4099802885	-2.2472761262
H	-7.8696924915	-2.3567937043	1.0576923059
H	-10.5836465201	-3.3674472025	2.5344580505
H	-9.8795606461	-4.8770458486	1.9699033968
H	-8.8237017861	-3.6026827969	2.6231725403

calc_**2c** conf_8

C	-7.1587817012	-2.3314264424	-4.3489187981
C	-7.2527184753	-2.0171172751	-2.8352914139

C	-7.8807907449	-3.2603092074	-2.2000926619
N	-7.992726737	-4.2667918068	-3.2759782639
C	-7.1415036279	-3.8639521412	-4.3928111626
C	-7.5621088882	-5.6404512304	-2.9700391119
C	-6.0336646389	-5.6219150866	-3.117742827
C	-5.7547042719	-4.5709761734	-4.2023064687
O	-6.0732363256	-1.6821887523	-4.963114182
O	-5.9516911787	-1.8076906936	-2.3036690019
C	-8.0550726673	-6.2488803949	-1.6585317736
C	-9.2813212557	-3.0014794348	-1.6319099701
O	-4.6804539281	-3.6950847218	-3.87381861
O	-7.4053372429	-5.7462396904	-0.5179468698
N	-9.2804750958	-2.2319944328	-0.404994782
O	-9.0677561	-4.0382136727	0.9465641458
C	-9.1575356194	-2.8197993831	0.8073386761
C	-9.1512555243	-1.8977071538	2.002685639
H	-7.5790828548	-4.2279490758	-5.3301010743
H	-8.0693649579	-1.9753227069	-4.8401126921
H	-7.8534008798	-1.1135575306	-2.6583922922
H	-7.2320514017	-3.5958869103	-1.3838268101
H	-7.9890947529	-6.2651700797	-3.7689055977
H	-5.6149745733	-6.6029020282	-3.3567754717
H	-5.5884080236	-5.2981803574	-2.1709153559
H	-5.4463274547	-5.0320831201	-5.1431681295
H	-5.3061216286	-2.2772983697	-4.8277785834
H	-5.5382228112	-1.1868054876	-2.9296389786
H	-7.8564330214	-7.3274377079	-1.7193551226
H	-9.1449558405	-6.1354920568	-1.5923720753
H	-9.8979363054	-2.486550298	-2.3748001588
H	-9.7648267517	-3.9550877515	-1.4178193931
H	-4.9184490781	-3.2521870375	-3.0346947757
H	-8.0327796199	-5.2088083615	0.0008511698
H	-9.2891803206	-1.2264251834	-0.4554140339
H	-9.3399620718	-0.8509379318	1.7535483324
H	-9.9088174695	-2.2401985137	2.7106006478
H	-8.1814761594	-1.9755017981	2.5006478316

calc_**2c** conf_9

C	-6.716259386	-2.3627110181	-3.6896297955
C	-7.7037752979	-2.1181408066	-2.5228160772
C	-8.0094333475	-3.5158864072	-1.9573730598
N	-7.8772689398	-4.3867639581	-3.114364075
C	-7.0877738138	-3.7665496544	-4.1822398887
C	-7.5839695737	-5.8159731985	-2.9937138231
C	-6.7330016284	-6.0988301455	-4.253360769
C	-5.996083608	-4.7937829866	-4.5355403271
O	-5.3952046318	-2.2669516864	-3.1942799866
O	-7.0924533139	-1.2442002364	-1.6036738416
C	-6.9344065753	-6.260179429	-1.674398872
C	-9.4377357906	-3.6573620662	-1.4059332582
O	-4.8465526661	-4.7514358391	-3.7088566487
O	-5.7747532248	-5.5742553582	-1.3035622024
N	-9.9231541972	-2.6223552186	-0.4970513091
O	-8.0471959231	-2.257525636	0.7282669339
C	-9.2516618775	-2.0789118166	0.5397773278
C	-10.056402701	-1.1968990762	1.4662813093
H	-7.7071992413	-3.6499797216	-5.0866716004
H	-6.84500349	-1.618599715	-4.484516442

H	-8.6246805745	-1.6662435981	-2.9174536876
H	-7.2769793065	-3.754274024	-1.1772548595
H	-8.5143036812	-6.4012906947	-3.0649288717
H	-7.3865271932	-6.341950927	-5.0973437934
H	-6.0329457465	-6.9277126908	-4.1189868903
H	-5.694226053	-4.7046460102	-5.5868039182
H	-5.4945068641	-1.7735785172	-2.3525250603
H	-7.2567784352	-1.5885024276	-0.6991943988
H	-6.7644323688	-7.3482826486	-1.759267333
H	-7.6536459793	-6.1289783711	-0.8561649329
H	-10.1105362333	-3.6555461171	-2.2674632916
H	-9.532106173	-4.6364139578	-0.9197161938
H	-4.7169325237	-3.8107131041	-3.4617018056
H	-5.2655572369	-5.3901152747	-2.1211080339
H	-10.9114676174	-2.4320823325	-0.5452522401
H	-9.5990728718	-0.2051854518	1.4896400566
H	-11.1089405884	-1.0991448251	1.1906691142
H	-9.9900713253	-1.6080450535	2.4765125452

calc_**2c** conf_10

C	-6.622332946	-2.4156912325	-3.8349604814
C	-7.766437461	-2.0461008462	-2.8983075912
C	-8.0552100857	-3.3942671617	-2.2243647534
N	-8.0033249417	-4.3298371681	-3.3504767749
C	-7.0593970072	-3.7814247352	-4.3632400809
C	-7.5604643454	-5.711523423	-3.0979259272
C	-6.0408174147	-5.678451177	-3.2701183668
C	-5.9187095823	-4.830395043	-4.5419501619
O	-5.4442119796	-2.5200050321	-3.0452252139
O	-7.329462409	-1.0336087303	-2.035541484
C	-8.0956429072	-6.4141540633	-1.8582871781
C	-9.3949364196	-3.4357368119	-1.4646322337
O	-4.6563621877	-4.3083191321	-4.837586059
O	-7.7014639042	-5.8770367694	-0.6063078974
N	-9.2405924365	-3.404982813	-0.025389024
O	-9.0543057258	-1.1494342718	0.0956956368
C	-9.0418952913	-2.2539622163	0.6469407668
C	-8.8331247245	-2.3745323377	2.1371147757
H	-7.5818787236	-3.6325139446	-5.315694724
H	-6.4704879912	-1.6864550344	-4.639089068
H	-8.6443024023	-1.7346937973	-3.48464603
H	-7.2518887641	-3.5633765857	-1.4930335169
H	-7.9605232205	-6.3164311327	-3.9284056673
H	-5.5833310908	-6.6662340481	-3.3784344409
H	-5.5553923729	-5.1486182963	-2.4408505486
H	-6.1719286087	-5.469939612	-5.395199145
H	-5.5409898602	-1.8217165725	-2.3718401229
H	-7.9705570994	-0.9535623801	-1.2942027687
H	-7.7971583756	-7.4699471025	-1.9286564396
H	-9.1880120287	-6.3928275242	-1.8578365815
H	-10.0278514476	-2.590046953	-1.7464845495
H	-9.9395786722	-4.3433346186	-1.725149655
H	-4.4685934321	-3.643556087	-4.1535231985
H	-6.736031331	-5.8688123746	-0.5804392885
H	-8.9781618531	-4.2804862966	0.4056160512
H	-9.6091382465	-1.7959575041	2.6439755654
H	-8.8633860808	-3.4038949622	2.5007042958
H	-7.8721166291	-1.9251322089	2.398751881

calc_**2c** conf_11

```
C  -7.3462853727   -2.4343435998  -4.3594814932
C  -7.4024804158   -2.0180040318  -2.8709722304
C  -7.8955112802   -3.2593372861  -2.1281071238
N  -7.7958501732   -4.3708376324  -3.0790242792
C  -7.1164071221   -3.9535084303  -4.2967309089
C  -7.16508744     -5.6339500917  -2.6765003891
C  -5.6787840176   -5.4852466203  -3.0582209189
C  -5.6392613035   -4.4584248435  -4.2045310132
O  -6.3989669168   -1.6931090399  -5.0874281341
O  -6.0991307932   -1.6682348099  -2.4196977994
C  -7.3470237157   -6.0556718685  -1.2233376311
C  -9.3569138617   -3.1484806144  -1.6619743273
O  -4.6750972153   -3.4321402037  -3.98942322
O  -8.6925352504   -6.2302490854  -0.8613728251
N  -9.521743852    -2.3711926784  -0.4499666362
O  -9.0137864792   -4.087257822   0.9434890001
C  -9.3128017975   -2.9065153635  0.7743611117
C  -9.4767577953   -1.9739433521  1.951213155
H  -7.5794051846   -4.444533433   -5.1622046447
H  -8.3236904087   -2.2454142607  -4.8144225213
H  -8.067441041    -1.1564835616  -2.7207949975
H  -7.266626013    -3.3915362275  -1.2394662782
H  -7.6219346379   -6.4158897798  -3.298277999
H  -5.2109251572   -6.4359474566  -3.3261317863
H  -5.1103417443   -5.0753919233  -2.2134589234
H  -5.3412122357   -4.9159391118  -5.1501070019
H  -5.5511613778   -2.1709865425  -4.9762427842
H  -5.7861489218   -1.023033536   -3.077608809
H  -6.8475808401   -5.3382643582  -0.5583849456
H  -6.8097952989   -7.0076962045  -1.1043177589
H  -9.9750998056   -2.7061084141  -2.4486327058
H  -9.7256750712   -4.158367171   -1.4861983509
H  -4.9358510695   -2.9706110276  -3.166830942
H  -8.8918492436   -5.5714124822  -0.1712664765
H  -9.7100338046   -1.384524074   -0.5179820097
H  -10.2432202625  -2.3810080377  2.6146819885
H  -8.5403206536   -1.9490723081  2.5132882117
H  -9.7522624263   -0.9543327162  1.6720643978
```

calc_**2c** conf_12

```
C  -7.0776509013   -1.9221148945  -3.370140069
C  -6.2310269962   -2.1564019854  -2.0942504782
C  -6.7339404998   -3.4993269876  -1.5234130004
N  -7.7613128389   -3.9807151192  -2.4581892494
C  -7.5592814185   -3.3229858896  -3.7491976643
C  -7.7456196853   -5.4142524475  -2.7953926049
C  -6.616860367    -5.5638564282  -3.8332515121
C  -6.584524278    -4.2219095167  -4.5716142823
O  -6.3334154325   -1.2613439946  -4.3771802876
O  -4.8532743698   -2.1440222118  -2.3976491365
C  -7.6464085416   -6.3692563573  -1.6158219831
C  -7.2910299031   -3.3799987139  -0.0990553143
O  -5.2435184181   -3.7348692668  -4.6037140008
O  -8.796543042    -6.4407646936  -0.8261131672
N  -8.4075680638   -2.4678209705  0.007955745
```

O	-10.0451851437	-4.0429688413	-0.0866616873
C	-9.7032336564	-2.8693014313	-0.0141189194
C	-10.7325166624	-1.7648182637	0.0652159916
H	-8.516228231	-3.2850168449	-4.2831339015
H	-7.9550855706	-1.3159385421	-3.1161835392
H	-6.3646547586	-1.3345435159	-1.384694816
H	-5.857543347	-4.1641196928	-1.4677496261
H	-8.707543958	-5.6194900372	-3.2832573543
H	-6.7709224256	-6.4152787439	-4.5002543978
H	-5.6484715014	-5.7062013318	-3.3393392723
H	-6.9425147629	-4.3178354597	-5.6033933272
H	-5.5010056732	-1.010259691	-3.9348545783
H	-4.7118997012	-2.883774444	-3.0278723328
H	-6.7460199694	-6.1273659033	-1.0226696195
H	-7.4776133894	-7.3746917301	-2.01759703
H	-7.6340403449	-4.3533592524	0.2554725018
H	-6.4941943005	-3.0450685154	0.57384948
H	-5.3213258803	-2.8558235451	-5.0157810852
H	-9.1479702194	-5.5461316443	-0.6480605512
H	-8.2181653171	-1.4828686013	0.0939180741
H	-11.2497009709	-1.6996656736	-0.8958215528
H	-11.4756859412	-2.0332282328	0.8180314388
H	-10.313503519	-0.7846105847	0.3059831095

calc_**2c** conf_13

C	-6.8213471006	-2.3001204701	-3.5216872072
C	-7.3355465181	-2.1050751042	-2.0985022439
C	-7.2618389432	-3.5228050779	-1.519024773
N	-7.801583547	-4.3088480959	-2.6138351508
C	-7.4566008561	-3.6455248664	-3.8935308264
C	-7.5602405943	-5.7571971853	-2.7221119904
C	-7.4061922074	-5.9621261487	-4.2407482619
C	-6.6978058915	-4.6977330558	-4.7174530153
O	-5.4026889824	-2.3402755633	-3.5066374526
O	-6.5592615264	-1.1571886267	-1.4229988899
C	-6.3552283083	-6.2806073531	-1.9234935642
C	-8.0038961781	-3.6782230635	-0.1908889436
O	-5.3287538384	-4.8315413096	-4.4092685101
O	-6.2203630491	-7.6822676483	-2.063912302
N	-9.3366289685	-3.1130214279	-0.156457561
O	-8.6370403201	-1.0514916693	0.4719144332
C	-9.5544179341	-1.8265417993	0.1980623839
C	-10.9962646348	-1.3832627337	0.2655562162
H	-8.3822537524	-3.4252934543	-4.4471972421
H	-7.1488763443	-1.4978458319	-4.1936672891
H	-8.3886081525	-1.8009965834	-2.1551197283
H	-6.1993704834	-3.7332718612	-1.3116079828
H	-8.4360366278	-6.3151150438	-2.3637857932
H	-8.3919323277	-6.0417419579	-4.7112327932
H	-6.8453927565	-6.8633562197	-4.4989007144
H	-6.8366237174	-4.5228057875	-5.7944759067
H	-5.126587696	-1.758799255	-2.7785414877
H	-7.0563598104	-0.9361979583	-0.6108665331
H	-6.5097155939	-6.1049905066	-0.8540348179
H	-5.4450038478	-5.7581347249	-2.2297108859
H	-8.0777503735	-4.7345419353	0.081047344
H	-7.4253618599	-3.1768221075	0.5891482568
H	-4.998238425	-3.9473245449	-4.172469401

H -5.7297736411   -7.8398444503 -2.8790089625
H -10.1078468849 -3.6749946444 -0.4769374158
H -11.708675652   -2.1541293106 -0.0367945238
H -11.2180199979 -1.0799261187 1.291770186
H -11.1268726575 -0.5030165047 -0.3675966504

calc_**2c** conf_14

C -6.6714499758   -2.3052590389 -3.8683590479
C -7.7402512583   -1.9973557647 -2.8128647986
C -7.9338568264   -3.3475805702 -2.0961143133
N -7.8901885198   -4.2707297504 -3.2139413166
C -6.9909220715   -3.7518336139 -4.2627027269
C -7.660616241   -5.7105025551 -3.0321886503
C -6.6943618474   -6.0772054767 -4.176713244
C -5.8788979474   -4.8050564168 -4.3869082956
O -5.3874691548   -2.1548122731 -3.2835639125
O -7.2758358805   -0.9453931037 -2.0180881872
C -7.0976123213   -6.1355735248 -1.6696313567
C -9.266523792   -3.4680785883 -1.3293733603
O -4.9168752096   -4.7441381217 -3.3610634861
O -7.0066440023   -7.5422748928 -1.5747309128
N -9.1776912806   -3.1921512671 0.0934691472
O -9.1853151921   -0.9370392089 -0.092607704
C -9.1596891987   -1.947600729   0.6122378164
C -9.1224833159   -1.8454333381 2.1177846154
H -7.5252865068   -3.7334840648 -5.2245200315
H -6.7467559634   -1.6286611353 -4.7276752465
H -8.6806246465   -1.73825543   -3.3213346076
H -7.1027669814   -3.4700931432 -1.3801329006
H -8.6134106361   -6.2525197929 -3.1639323419
H -7.2491992081   -6.3234874698 -5.0879533617
H -6.0637564515   -6.928664274   -3.9132074312
H -5.4059883124   -4.7707387459 -5.3797562863
H -5.5138046058   -1.5064590615 -2.5666719923
H -7.9439052863   -0.782503809   -1.3178790205
H -7.7157610911   -5.7339729659 -0.8499028253
H -6.0838295772   -5.7528236571 -1.5538989668
H -10.0142427152 -2.7979816296 -1.7635468838
H -9.6496014553   -4.4853673294 -1.4317943667
H -4.7351198625   -3.7985658861 -3.2107162759
H -7.8869798545   -7.9036374289 -1.7423803412
H -9.1019104924   -3.9734675675 0.7245307175
H -9.9789318212   -1.2523024449 2.4464391119
H -9.1406935906   -2.8110570543 2.6280044547
H -8.2207469061   -1.3029388757 2.4116883295

calc_**2c** conf_15

C -6.2417042566   -2.6029405763 -3.717873018
C -7.2770248313   -1.966808115   -2.7851662108
C -7.8770602612   -3.1865108831 -2.0498287823
N -7.8768629825   -4.225479672   -3.0663339565
C -7.0142638679   -3.8289496121 -4.2020239523
C -7.6330973657   -5.6270388403 -2.7313486038
C -7.231495717   -6.1824964643 -4.1050263196
C -6.2873611571   -5.1103776382 -4.6478104734
O -5.1259896598   -2.8940648838 -2.9017063864
O -6.732815243   -0.9985833139 -1.9230535502

C	-6.5380324601	-5.8372489317	-1.654069407
C	-9.2626120726	-2.9165753087	-1.4724004626
O	-4.9723406768	-5.2415610001	-4.1045459647
O	-5.7006660585	-6.9352708842	-2.0308644528
N	-9.1844866447	-1.89080851	-0.4561436429
O	-10.6451993801	-2.8865157796	0.9670688593
C	-9.8734877644	-1.9732242202	0.7119885951
C	-9.626915942	-0.8413933618	1.6890731011
H	-7.6466589375	-3.5024161552	-5.0423267192
H	-5.9552074178	-1.937037679	-4.5427201491
H	-8.056104831	-1.4685502539	-3.3732067764
H	-7.2033292573	-3.4117917054	-1.2082473488
H	-8.5603974327	-6.0982338702	-2.3894259496
H	-8.125576992	-6.229400353	-4.7333274922
H	-6.7808485318	-7.1751461202	-4.0694132643
H	-6.1880969935	-5.1462228883	-5.7365581879
H	-4.7240409094	-3.7048919763	-3.2768476019
H	-5.8755359458	-1.3652177658	-1.6493688356
H	-6.9957020834	-6.0367062758	-0.6784275599
H	-5.9308060154	-4.9289149509	-1.5721758438
H	-9.9438530894	-2.6277990567	-2.2842133195
H	-9.6823935399	-3.8083367092	-1.0002024863
H	-4.9880314997	-5.9738665674	-3.4554180854
H	-5.0946610871	-7.1117145264	-1.2994447643
H	-8.5018194198	-1.1576716071	-0.5932208196
H	-10.5718573949	-0.3236653257	1.8716217245
H	-9.3007960933	-1.2648832106	2.6419514121
H	-8.8858661869	-0.1146850075	1.3470366948

calc_**2c** conf_16

C	-7.4816816338	-2.1858258081	-3.4047906644
C	-6.7415461771	-2.3642794306	-2.0598187458
C	-7.1368905405	-3.7529164013	-1.5491830537
N	-7.9725481097	-4.363339653	-2.6198116772
C	-7.7360152321	-3.626262893	-3.8632296721
C	-7.6902059976	-5.7562541921	-2.9932822498
C	-6.4356266366	-5.6927408625	-3.8770019285
C	-6.5677385827	-4.3511843298	-4.6202362508
O	-6.7909501226	-1.36644266	-4.3130859283
O	-5.3308486772	-2.3400585027	-2.2720150956
C	-7.657284322	-6.7704261931	-1.86561311
C	-7.9297073762	-3.702901232	-0.2449593766
O	-5.3523811917	-3.6200651402	-4.686000782
O	-6.4941688791	-6.6089261418	-1.0786949886
N	-9.2040836759	-3.0527856507	-0.4308398097
O	-8.8622135222	-1.3846578133	1.0731727138
C	-9.5647157566	-1.917798766	0.2271151841
C	-10.9188708265	-1.3534325818	-0.1522498831
H	-8.6326080594	-3.6826175834	-4.4919878469
H	-8.4452258818	-1.7070652512	-3.2055785605
H	-7.0050507332	-1.5765039205	-1.3454717594
H	-6.2209374058	-4.3235394462	-1.3657410588
H	-8.5391606836	-6.0640232837	-3.6218910168
H	-6.3458900949	-6.5470828894	-4.553130068
H	-5.538273253	-5.6758870186	-3.2507698212
H	-6.8506010554	-4.5025807097	-5.6644341161
H	-6.1489140056	-1.9539084819	-4.7601941503
H	-5.1666793225	-1.5038185771	-2.7392843609

H -7.6827752218   -7.7715172036 -2.3204789942
H -8.569914184   -6.6562771381 -1.2627663938
H -8.0742406264   -4.7224089051 0.1288224481
H -7.3882196104   -3.1401638479 0.5181468204
H -5.0880525226   -3.4080881983 -3.7651152543
H -6.4951258866   -7.2897464313 -0.3955411793
H -9.7663166291   -3.4022653908 -1.1921673415
H -11.5511288919 -1.3334301473 0.7387302584
H -10.7895414577 -0.3192613621 -0.4799369035
H -11.4308672143 -1.9165159617 -0.9366853831

calc_**2c** conf_17

C -6.6750942072 -2.3507917887 -3.8877847348
C -7.7453035248 -1.9215536686 -2.8917137134
C -8.0478919826 -3.2385504137 -2.1561917208
N -8.0100242703 -4.2352567417 -3.2270042521
C -7.1850724061 -3.7136974024 -4.3447360165
C -7.5150602423 -5.5924368108 -2.9224151729
C -6.040585224   -5.577537671   -3.3258239436
C -6.0829381869 -4.7734803501 -4.6304491746
O -5.451750899   -2.4746011502 -3.1765433466
O -7.2243037358 -0.9038698236 -2.0838527953
C -7.7852378912 -6.0843997116 -1.5052042708
C -9.4057343386 -3.2279455076 -1.4139587298
O -4.8645639178 -4.2561870778 -5.0846942011
O -7.3869124049 -7.4274984032 -1.3380966462
N -9.3064964415 -3.2441085403 0.0331496798
O -8.6998109478 -1.0727696524 0.2149677389
C -8.9699713701 -2.1499738331 0.7473186293
C -8.9533403431 -2.3004836009 2.2489018826
H -7.8076854543 -3.5676599519 -5.2361740177
H -6.5582463149 -1.6467372961 -4.7201661665
H -8.6451495194 -1.591407684   -3.433802802
H -7.2467043322 -3.3706853195 -1.4122447409
H -8.0424208085 -6.2911041656 -3.5878604659
H -5.6081995248 -6.571497168   -3.4747883883
H -5.4437769489 -5.0308007981 -2.5854309113
H -6.4342177332 -5.4327892734 -5.4314350643
H -5.4792217697 -1.7542409949 -2.5201114842
H -7.7641164174 -0.850277924   -1.2653292279
H -8.8603824551 -6.0739140009 -1.3013942631
H -7.2952733012 -5.4334329256 -0.7659849933
H -9.9837633682 -2.3428555361 -1.6987188245
H -9.9847244553 -4.1000302434 -1.7197046202
H -4.585615635   -3.6025366301 -4.4216851312
H -6.430895739   -7.4639016887 -1.4678338124
H -9.4836596418 -4.1067061897 0.5214516352
H -7.9527509439 -2.0610368441 2.6163900772
H -9.6403726843 -1.5683271079 2.6794037333
H -9.232730619   -3.29791611   2.5955502564

calc_**2c** conf_18

C -7.2459899791   -2.4653046733 -4.5053771967
C -7.0338454379   -1.8665982356 -3.0973965848
C -7.5270658067   -2.9608957192 -2.1249149478
N -7.7871904212   -4.1659951757 -2.9550107395
C -7.1668636251   -3.9744664815 -4.2735478612

```
C  -7.2417156673   -5.4610212373  -2.5299326272
C  -5.756882357    -5.4346471022  -2.9243397953
C  -5.7418167222   -4.6208769374  -4.2194262475
O  -6.303142231    -1.9648443089  -5.4371107054
O  -5.6922317845   -1.4703972195  -2.9208880588
C  -7.4950820235   -5.898114065   -1.0998541386
C  -8.7890444917   -2.4748014687  -1.4056236683
O  -4.6707448931   -3.6880971818  -4.1642665154
O  -8.864442149    -6.1350424857  -0.8128530636
N  -9.2580324976   -3.35001236    -0.3654721496
O  -9.1847077056   -1.7732407104  1.2740301202
C  -9.4145720434   -2.9198663875  0.9174458396
C  -9.9211468716   -3.9708433009  1.8839837174
H  -7.7521478796   -4.5196105606  -5.0239897354
H  -8.2563170034   -2.2240311111  -4.8553017645
H  -7.6136707657   -0.9481573728  -2.9747374885
H  -6.7398582076   -3.1267036233  -1.3766203368
H  -7.7346597221   -6.2081455562  -3.1758805568
H  -5.3221711636   -6.4310402389  -3.0355342247
H  -5.1674355618   -4.896263262   -2.1730907199
H  -5.5999248357   -5.2666000861  -5.094851228
H  -5.8305274349   -1.2661618867  -4.9471921691
H  -5.1382830548   -2.2667471776  -3.0674273332
H  -7.1702737542   -5.1380748458  -0.3857922967
H  -6.9035602987   -6.8039208755  -0.9076516706
H  -8.5862237183   -1.516112796   -0.9212283345
H  -9.5725753758   -2.3198453264  -2.1602074006
H  -4.8008711458   -3.1202858775  -4.9460498153
H  -9.2208878766   -6.7175358244  -1.4960225694
H  -9.4444785848   -4.3209626379  -0.593386582
H  -9.3317548939   -3.9131657485  2.8011667732
H  -10.9566711545  -3.7377173832  2.1485586567
H  -9.8771908607   -4.9858527589  1.4827934189
```

calc_**2c** conf_19

```
C  -7.4768563454   -2.2378111632  -3.702420941
C  -6.758046429    -2.1371007271  -2.3318543598
C  -6.9302804079   -3.5257290136  -1.6789707413
N  -7.7277974125   -4.3288212534  -2.6374637383
C  -7.5485806948   -3.7408225541  -3.9717780532
C  -7.3212994553   -5.7328717716  -2.8397426614
C  -6.076405498    -5.669207652   -3.7392414759
C  -6.2880556549   -4.4146646713  -4.596445939
O  -6.8005937357   -1.5030934345  -4.7055452632
O  -5.410574159    -1.7562950696  -2.5002380597
C  -7.1687811784   -6.5358007271  -1.5620202419
C  -7.6280525463   -3.4516925831  -0.3213165924
O  -5.1172740729   -3.6064895506  -4.5361396901
O  -8.3865302306   -6.6928975426  -0.8712362574
N  -9.0049365666   -3.0581574211  -0.5003504487
O  -9.1422137514   -1.840430324   1.4057384163
C  -9.6690700412   -2.2734483284  0.3940428499
C  -11.1138201025  -1.9782988849  0.0489003861
H  -8.4148575548   -3.9999212985  -4.5914515614
H  -8.4990299894   -1.8537538116  -3.6030984978
H  -7.2059576094   -1.3546428884  -1.7157513067
H  -5.9235928324   -3.9379154776  -1.5212217565
H  -8.1402304247   -6.1976458699  -3.4048385632
```

H	-5.9454519744	-6.5758412388	-4.3342865565
H	-5.1644125495	-5.5299064697	-3.1483642112
H	-6.4834568542	-4.6702469412	-5.6444902413
H	-6.1424490516	-0.9759155905	-4.2145145071
H	-4.9925983827	-2.4773992983	-3.015483036
H	-6.3919085515	-6.090286311	-0.9209068206
H	-6.8273380924	-7.5430248081	-1.8169595671
H	-7.5599435608	-4.4135119318	0.203280104
H	-7.1439483501	-2.710705759	0.3194033738
H	-5.3589688804	-2.804428197	-5.0344004754
H	-8.8094510838	-5.8251579207	-0.8318031362
H	-9.459435003	-3.4056493026	-1.3329924373
H	-11.4192572769	-2.3476083175	-0.9333746339
H	-11.755792446	-2.429341469	0.8100257749
H	-11.2707512494	-0.8984644268	0.0913108667

calc_**2c** conf_20

C	-7.1685462742	-2.4095061601	-4.1921687604
C	-7.8955671769	-2.2084557727	-2.8383358362
C	-7.8163167015	-3.5682450473	-2.1111406772
N	-7.8440868985	-4.5016607324	-3.2570164414
C	-6.9124088501	-3.9192107155	-4.2444831957
C	-7.3050858068	-5.8673317779	-3.1109477016
C	-5.7705146834	-5.7596529875	-3.1542074982
C	-5.489479193	-4.4165222926	-3.8460861038
O	-5.9860262034	-1.6317017502	-4.2711410045
O	-7.2990540637	-1.132043362	-2.1721355185
C	-7.8725846656	-6.6504211599	-1.9498617367
C	-8.9934433927	-3.7633989266	-1.1340329239
O	-4.8350024072	-3.5914301493	-2.9102890547
O	-9.2788487339	-6.7875361509	-2.0417189589
N	-8.6999763007	-3.3498082063	0.2277628962
O	-9.0221182782	-1.1308590843	-0.0924003304
C	-8.7593919396	-2.0738476835	0.6574056802
C	-8.4940820182	-1.847122573	2.1265306926
H	-7.1464130441	-4.3367216108	-5.2315715734
H	-7.8286913637	-2.1140181169	-5.0151137861
H	-8.9538957868	-1.9958579084	-3.0389474265
H	-6.8677142082	-3.603511226	-1.5574693223
H	-7.6307706127	-6.3900053157	-4.0226566506
H	-5.3276250421	-6.6113529462	-3.6765527985
H	-5.3318779992	-5.7143818712	-2.1529660332
H	-4.8733164326	-4.5386877035	-4.748713188
H	-6.1269886422	-0.9195131281	-3.6175970818
H	-7.8939366416	-0.8928257743	-1.4308859397
H	-7.5923757086	-6.1712727881	-0.9989370127
H	-7.4331961616	-7.6537076208	-1.9422856718
H	-9.858681963	-3.1884781987	-1.478378057
H	-9.3026103696	-4.8073137132	-1.0869469695
H	-4.8882979917	-2.695137487	-3.2916431218
H	-9.5756055129	-6.1292643351	-2.6865245044
H	-8.4547549383	-4.0644868244	0.8936180711
H	-9.3416636282	-1.3058922148	2.5527823653
H	-8.3344165979	-2.7660607022	2.6950955459
H	-7.6146337674	-1.2067559827	2.2299596278

calc_**2c** conf_21

C	-7.4359554683	-2.3492301146	-3.9937547643
C	-7.5283450146	-2.2213448754	-2.4589429596
C	-7.9740357897	-3.6207221156	-1.9902448623
N	-7.8990689278	-4.5221371866	-3.1716713622
C	-7.1099216272	-3.8297385158	-4.2028383132
C	-7.1438561983	-5.7758244428	-2.9678631738
C	-5.6742108077	-5.3584304213	-2.9347127566
C	-5.6054199229	-4.2429059326	-3.9990672525
O	-6.6041234125	-1.4445252797	-4.6473927341
O	-6.2644142589	-1.8863045957	-1.913164436
C	-7.6958920882	-6.604288299	-1.8264398035
C	-9.3944688835	-3.6597621438	-1.4267847523
O	-4.7241737785	-3.1894576871	-3.6648453838
O	-9.0620200719	-6.9000897621	-2.0489075987
N	-9.6529806448	-2.7520632924	-0.3111946644
O	-7.5558201903	-2.3352083989	0.5087526785
C	-8.7789655276	-2.2145471914	0.5707188375
C	-9.4048303629	-1.4076637918	1.6883625158
H	-7.4370429411	-4.1622500436	-5.1932934911
H	-8.4472846582	-2.1791376943	-4.3799431368
H	-8.252136397	-1.4466247426	-2.1809886208
H	-7.2832486863	-3.9361714239	-1.2055117582
H	-7.3113129312	-6.3637705117	-3.8823047475
H	-4.984255861	-6.1812841696	-3.1371674969
H	-5.4085861601	-4.9402909946	-1.9565900345
H	-5.2189085912	-4.6398806492	-4.9409202776
H	-5.6936729678	-1.7294338054	-4.447098983
H	-6.3969324045	-1.9280177646	-0.9465014176
H	-7.5623793533	-6.0843863533	-0.8661497064
H	-7.1497039752	-7.5506639208	-1.7579481058
H	-10.0889693027	-3.3941956933	-2.2315959561
H	-9.6289941615	-4.6860421003	-1.1213113159
H	-5.0458956128	-2.791745415	-2.8262829022
H	-9.3786499972	-6.2171991666	-2.6594578604
H	-10.6288217671	-2.597586848	-0.1128105642
H	-8.9756929663	-0.4033543041	1.6737806378
H	-10.493271991	-1.331187548	1.6358771975
H	-9.1267362986	-1.8645328041	2.6412093255

calc_**2c** conf_22

C	-7.5713198783	-2.2681069679	-3.4441407678
C	-6.8176896331	-2.271478091	-2.0895335447
C	-6.9648715512	-3.7097163833	-1.549899528
N	-7.7403022802	-4.4480215468	-2.5665908206
C	-7.6250438998	-3.7437116741	-3.8443428367
C	-7.3206627286	-5.8154437236	-2.8905437303
C	-6.1267410455	-5.6619447725	-3.8542615331
C	-6.3858279968	-4.335911614	-4.5805890446
O	-6.9330884004	-1.4347120746	-4.3963139984
O	-5.4745240598	-1.868987316	-2.269377268
C	-7.0393168443	-6.7201576754	-1.7110106326
C	-7.6800530568	-3.7622855862	-0.1970828573
O	-5.2224596238	-3.5204369311	-4.4887363836
O	-8.2343048915	-6.9020212706	-0.9790067869
N	-9.0582354179	-3.3571307688	-0.3188149551
O	-8.8670632786	-1.4274573351	0.8642755814
C	-9.5492867373	-2.2080020582	0.2148780714
C	-11.0233675712	-1.9590804365	-0.0319274697

H	-8.5120908403	-3.9613787858	-4.452237071
H	-8.5963989187	-1.914967998	-3.2854195899
H	-7.2449466685	-1.5445222242	-1.3958947863
H	-5.9470868199	-4.1085073437	-1.4164300699
H	-8.1589379634	-6.2681445754	-3.4371478807
H	-6.0253332302	-6.5097837981	-4.5360740643
H	-5.1838866834	-5.5671259342	-3.3039422719
H	-6.6189318932	-4.4950155091	-5.6403754599
H	-6.2655174198	-0.9488770174	-3.8757249669
H	-5.0679209781	-2.5398094434	-2.8555477505
H	-6.2415427033	-6.2935495577	-1.0847172795
H	-6.663663928	-7.6759466548	-2.1054058339
H	-7.6455694409	-4.7793053046	0.2031343914
H	-7.2014339756	-3.0853373791	0.5133649423
H	-5.5027271469	-2.6721154204	-4.8801423452
H	-8.0524135677	-7.516462946	-0.2575716859
H	-9.6265282145	-3.9214334596	-0.9312662528
H	-11.1458806933	-0.958414246	-0.452014508
H	-11.4906088163	-2.6896634969	-0.6969016952
H	-11.5454212031	-1.9730326799	0.9283326829

calc_**2c** conf_23

C	-7.36793157	-2.6465208664	-4.6215955006
C	-7.4414976165	-1.949584423	-3.2415598021
C	-7.7125917851	-3.0805837295	-2.2192132579
N	-7.7672490138	-4.3387826773	-3.0195209596
C	-7.0414388203	-4.0956326137	-4.2784597895
C	-7.0531600878	-5.5215014709	-2.5006958334
C	-5.5625574924	-5.2370138455	-2.7032878334
C	-5.5374778351	-4.4407873263	-4.0144429386
O	-6.4245153687	-2.0242090959	-5.4746263165
O	-6.2710362762	-1.2066583031	-2.988193705
C	-7.5189265115	-5.9505693827	-1.1303607737
C	-9.0072661049	-2.7688256991	-1.4527972404
O	-4.6993063722	-3.3048627371	-3.8513833678
O	-8.9231693113	-6.1846202725	-1.1472208582
N	-9.2279285101	-3.5057411042	-0.234967535
O	-9.1661820373	-1.6742960197	1.1155539616
C	-9.3083612402	-2.8791518015	0.9733748743
C	-9.596917368	-3.7925358899	2.14664322
H	-7.4194939815	-4.7858349283	-5.0412197811
H	-8.3555409852	-2.6176873014	-5.0962812025
H	-8.2480170386	-1.2124189301	-3.2303524218
H	-6.8827021215	-3.0828988071	-1.4989518194
H	-7.33002171	-6.3370373838	-3.1864240439
H	-4.9581614647	-6.1460995707	-2.7417247854
H	-5.16168564	-4.6042722146	-1.9044540902
H	-5.1505618586	-5.0476925681	-4.8420529083
H	-6.1867509474	-1.2004537002	-5.0083563435
H	-5.5369285105	-1.8548170364	-2.961945917
H	-7.2788618944	-5.187159835	-0.3793293467
H	-7.0189101942	-6.8793339697	-0.8383888805
H	-8.9779427938	-1.7197145909	-1.1468818954
H	-9.8527966915	-2.9018721387	-2.1415339637
H	-4.813905368	-2.8035246385	-4.6795650057
H	-9.2438050999	-5.8002144002	-1.979154025
H	-9.3899529867	-4.5056476061	-0.2943536376
H	-8.838405841	-3.6261646383	2.9147799445

H -10.5615197324 -3.5143175258 2.5791713282
H -9.6185218185   -4.8529609576 1.8837724508

calc_**2c** conf_24

C -7.3845072046   -2.1450618701 -3.3301354947
C -6.703964265   -2.4141708172 -1.9641964348
C -7.1417849601   -3.8368028023 -1.5644736693
N -7.9971360224   -4.3284915881 -2.6742409447
C -7.6925809627   -3.5420585051 -3.8730121881
C -7.8160386685   -5.7127879048 -3.124306792
C -6.5615584265   -5.6925873154 -4.0182091418
C -6.5488126156   -4.2866640846 -4.6292854861
O -6.5628033711   -1.36277744   -4.1790710518
O -5.3030441633   -2.2599068887 -2.0729675259
C -7.8473332017   -6.7925576956 -2.0489671046
C -7.9214110063   -3.8588609119 -0.2503157479
O -5.2625319776   -3.7096109671 -4.4368181836
O -6.7433427472   -6.8106604445 -1.1684544615
N -9.1825529456   -3.1703042866 -0.3853063791
O -8.7907011398   -1.594463339   1.2043834443
C -9.5136436033   -2.0682314964 0.3406440075
C -10.8650324555 -1.4638562481 0.0158001752
H -8.5742170912   -3.5322050901 -4.5259793128
H -8.3326977053   -1.6210917107 -3.1643339225
H -7.0150742261   -1.6795772645 -1.2184607793
H -6.2431492665   -4.4493357034 -1.4295223883
H -8.682701259   -5.9265518726 -3.7679613538
H -6.5616556199   -6.4873779384 -4.7686326075
H -5.6501267646   -5.7971425077 -3.419248917
H -6.7658697015   -4.3116262263 -5.7040613264
H -5.8363588244   -1.0691301047 -3.5964348201
H -5.0045542836   -2.9466961803 -2.7035024438
H -7.9645031213   -7.7652022882 -2.548242386
H -8.7304307972   -6.6458089206 -1.4218040676
H -8.0827923983   -4.8936266664 0.0686385995
H -7.3587945062   -3.3522977379 0.536539324
H -5.3702128826   -2.7910014363 -4.7470893713
H -5.978125947   -7.1463354089 -1.650657303
H -9.7584533291   -3.4586953549 -1.1616058822
H -11.3827450427 -1.953437217   -0.8130315889
H -11.495599016   -1.5193147485 0.9066822558
H -10.7301584809 -0.405691017   -0.21935873

calc_**2c** conf_25

C -7.3054715723 -2.3213517297 -3.8385549614
C -7.2120284058 -2.311596067   -2.2916792505
C -7.5980931681 -3.7447594907 -1.8661960092
N -7.8517738753 -4.5007443376 -3.1148193376
C -7.1689429144 -3.7980786375 -4.2103757603
C -7.2860597146 -5.8591842811 -3.202021231
C -5.7853481413 -5.6645150605 -3.4486415671
C -5.7199526885 -4.3841096038 -4.2914864026
O -6.3258065296 -1.4830320501 -4.427598712
O -5.9225604454 -1.9153403313 -1.8719403004
C -7.6992358197 -6.7529877919 -2.0521456053
C -8.8446772289 -3.7728139697 -0.9723712688
O -4.7171009804 -3.5277256178 -3.758534355

O	-9.1013906268	-6.9169093831	-2.0145256302
N	-8.6568161027	-3.0919066507	0.2880620756
O	-9.7347702193	-1.1890853672	-0.3160014357
C	-9.1328696572	-1.8381077594	0.5272147738
C	-8.8557212551	-1.2891443629	1.9097535114
H	-7.6824504756	-4.0250487567	-5.1515048894
H	-8.300402865	-1.9787355722	-4.1435019203
H	-7.8969356181	-1.5764142146	-1.8665684211
H	-6.7473703092	-4.15060901	-1.2974540926
H	-7.7267428134	-6.3004168483	-4.1075511614
H	-5.3227748882	-6.524138768	-3.9391118219
H	-5.2451649566	-5.4888378678	-2.5116917434
H	-5.4717782086	-4.6065584056	-5.3364273341
H	-5.9539181767	-0.9860448355	-3.6749002805
H	-5.3005238988	-2.5751618932	-2.2439441103
H	-7.3220260231	-6.3500264194	-1.0991044112
H	-7.2529038191	-7.7444046694	-2.1783240341
H	-9.6660766851	-3.2711606779	-1.4888464479
H	-9.1472992707	-4.805684705	-0.7771306139
H	-4.8250625418	-2.6967494829	-4.2584654786
H	-9.4774423094	-6.1179863896	-2.4120175133
H	-8.148540319	-3.5630913776	1.0185449862
H	-8.1535139348	-0.4559024972	1.8220127
H	-9.7860359448	-0.8907877047	2.3191023011
H	-8.4434175969	-2.0248474122	2.6047457529

calc_**2c** conf_26

C	-7.201144057	-2.2422100028	-3.8116053501
C	-7.180345366	-2.3536517594	-2.2697143977
C	-7.7993626364	-3.7188743396	-1.9578992803
N	-7.9989668885	-4.3970428481	-3.2609090101
C	-7.2053028513	-3.7041048556	-4.2737260947
C	-7.5697212723	-5.7937127687	-3.3718299132
C	-6.0455434201	-5.7324517267	-3.5608390941
C	-5.8166035785	-4.4331899396	-4.3572175086
O	-6.1651421012	-1.4401882205	-4.321442973
O	-5.8283603237	-2.3361206221	-1.8008329353
C	-8.0803024855	-6.7515165053	-2.3017580127
C	-9.1414479529	-3.6183747308	-1.227257761
O	-4.7105577237	-3.679364039	-3.8869744631
O	-7.5612415601	-6.559628682	-0.9987394222
N	-9.0024259008	-3.1836867269	0.1439328852
O	-9.5814711949	-1.0196986301	-0.2215769542
C	-9.2316608458	-1.9050217477	0.5464254226
C	-9.026183122	-1.640844262	2.0234083227
H	-7.6970682596	-3.8010971501	-5.2488351087
H	-8.1454768224	-1.7792731623	-4.1122009529
H	-7.7308799031	-1.5361351337	-1.7950392868
H	-7.1047436952	-4.265850753	-1.310628947
H	-8.0093645199	-6.1643008694	-4.3104393313
H	-5.631980115	-6.615044655	-4.0566450514
H	-5.5380090583	-5.6303373796	-2.593646978
H	-5.5790672716	-4.649631309	-5.4012864645
H	-5.3898701734	-2.0298928304	-4.4009915414
H	-5.4603959177	-1.5092991115	-2.1551302273
H	-7.8894688279	-7.7781902868	-2.6458272152
H	-9.1635822602	-6.6392201434	-2.2119989231
H	-9.7970045373	-2.9072057507	-1.7337004903

H	-9.6311081604	-4.5952189117	-1.2398275996
H	-4.9218678523	-3.4046172581	-2.9675979725
H	-6.6312832584	-6.8174843829	-1.0096937485
H	-8.6626889604	-3.8546846294	0.8141800902
H	-9.9369783246	-1.1933795024	2.4273173765
H	-8.7790336245	-2.5328005884	2.6045840454
H	-8.2243451771	-0.9076537854	2.1409648665

calc_**2c** conf_27

C	-7.2008588926	-2.5030069594	-4.4603115548
C	-7.4070558746	-2.073860517	-2.9831367209
C	-7.827476514	-3.3621275892	-2.2474933869
N	-7.8889448576	-4.423055788	-3.2791413662
C	-7.0008813847	-4.01437498	-4.37468155
C	-7.336334076	-5.7368350046	-2.8937220572
C	-5.8122023073	-5.555854574	-2.8989040475
C	-5.5694477278	-4.524778127	-4.009282437
O	-6.128576156	-1.8018776043	-5.0629542399
O	-6.240422206	-1.4670677588	-2.4707102551
C	-7.9764604719	-6.3079320999	-1.6452472421
C	-9.1747668386	-3.2212670472	-1.5298283472
O	-4.7020492773	-3.5056088844	-3.521666832
O	-9.3588334791	-6.5341400658	-1.8578895288
N	-9.1280915978	-2.2640916326	-0.4458417354
O	-8.0225703457	-3.6648264599	0.9443399981
C	-8.5106192404	-2.5646844045	0.7297058796
C	-8.4547471327	-1.4470257521	1.7476666356
H	-7.3187419787	-4.5156709809	-5.2959263857
H	-8.1185190855	-2.3095430624	-5.0277732033
H	-8.1781480166	-1.3011068627	-2.9130411482
H	-7.0719743353	-3.5686084192	-1.4781228482
H	-7.6043004582	-6.4148763666	-3.7164643877
H	-5.2797947389	-6.4949223996	-3.0657784211
H	-5.4550189104	-5.1387706786	-1.9515690279
H	-5.105308885	-4.9849327017	-4.8899238202
H	-5.8979967928	-1.1136144656	-4.4102904501
H	-5.55370224	-2.1668154467	-2.4779374158
H	-7.8118412015	-5.6499166884	-0.7804239667
H	-7.5223688228	-7.2763072579	-1.4131122099
H	-9.9409552803	-2.9050659824	-2.244371423
H	-9.4815398236	-4.1847969984	-1.1170723057
H	-4.6689427722	-2.8565901565	-4.2474168286
H	-9.6488603293	-5.850478103	-2.4784195245
H	-9.4331899852	-1.3190915143	-0.6096094302
H	-8.6599150955	-1.8646545113	2.7344259233
H	-7.4416914647	-1.0348704488	1.7669885713
H	-9.1578514035	-0.6349517064	1.5459370901

calc_**2c** conf_28

C	-7.5915148903	-2.2067132225	-3.4149825542
C	-6.7852825103	-2.2475199764	-2.0971949388
C	-7.0690902395	-3.6235063734	-1.484979288
N	-7.8839386933	-4.3622661961	-2.4820828106
C	-7.7537357013	-3.6876604332	-3.7780962561
C	-7.4903990143	-5.7474099342	-2.7750917167
C	-6.2825418422	-5.6371966859	-3.7145512422
C	-6.5669597823	-4.371611561	-4.5434629629

O	-7.0018215686	-1.3937673194	-4.396320034
O	-5.3899321209	-2.1478484691	-2.3745416534
C	-7.291147552	-6.6290957842	-1.5574023984
C	-7.8279658786	-3.5314473222	-0.1602068858
O	-5.4221262862	-3.5506548006	-4.7276190434
O	-7.0441105689	-7.9321373297	-2.0434541025
N	-9.1403704687	-2.966502192	-0.3498708623
O	-8.8101498635	-1.1386724034	0.9568695652
C	-9.5289244959	-1.7887677087	0.212806846
C	-10.9328890943	-1.3390821405	-0.1337574007
H	-8.6697227618	-3.8486201131	-4.3579141411
H	-8.5796936441	-1.7892930812	-3.1993760611
H	-7.0710963004	-1.4352962251	-1.4199843907
H	-6.0972744219	-4.1015002266	-1.2979343221
H	-8.3245745658	-6.1901330281	-3.3361275715
H	-6.1352529812	-6.5310987447	-4.3221140794
H	-5.3622572138	-5.4842488675	-3.1371636736
H	-6.8863390916	-4.6244498989	-5.556764329
H	-6.3449477955	-1.9613607093	-4.8477979411
H	-5.3024551538	-1.333520497	-2.8980952422
H	-8.1961135126	-6.5953630345	-0.9325520419
H	-6.4476020291	-6.2675674736	-0.9502872485
H	-7.9148278704	-4.5274894675	0.2896530833
H	-7.2991569045	-2.8882308928	0.546334432
H	-5.1226107344	-3.2701210097	-3.837379719
H	-6.876728137	-8.507433224	-1.2879023579
H	-9.7207278607	-3.4209879055	-1.0383033048
H	-11.4389767307	-1.9829895694	-0.8573509359
H	-11.5255482403	-1.3064300292	0.7839322588
H	-10.8891934793	-0.3210061497	-0.5269346758

calc_**2c** conf_29

C	-7.1711550698	-2.3362184186	-4.3944905008
C	-6.8006562419	-1.8612083848	-2.9727408664
C	-7.3905680147	-2.9398968667	-2.040532805
N	-7.8044471361	-4.0659415269	-2.9116105386
C	-7.2559015735	-3.85856094	-4.2577108433
C	-7.3787331804	-5.4346979675	-2.5886591178
C	-5.9322452417	-5.5427336419	-3.0935436337
C	-5.9172128182	-4.6665120222	-4.3462448217
O	-6.237820566	-1.8828545157	-5.3601837931
O	-5.4093249799	-1.6631705218	-2.8602563276
C	-7.5644544171	-5.9096353724	-1.1642524298
C	-8.5864892032	-2.3587653649	-1.2825672868
O	-4.7403250918	-3.8678996548	-4.3332246212
O	-8.9490495416	-6.1321384996	-0.9308467215
N	-9.199778925	-3.2832874406	-0.3656614581
O	-9.1672033874	-1.8621335813	1.4116656297
C	-9.4502918357	-2.94848445	0.9279090426
C	-10.1126426384	-4.0305853554	1.7584811387
H	-7.9493875327	-4.2806872608	-4.9946768873
H	-8.16622081	-1.95928739	-4.6571289925
H	-7.2401143817	-0.8827763302	-2.7606783043
H	-6.6149550426	-3.2261932584	-1.317539646
H	-8.006929001	-6.0939786427	-3.2037238795
H	-5.620917828	-6.5726832152	-3.2850523984
H	-5.2328869085	-5.1131301716	-2.3663172149
H	-5.9173396104	-5.2747403963	-5.2589719214

```
H  -5.6496284274   -1.2849565212  -4.8628322637
H  -4.97591898     -2.5061993475  -3.1159723802
H  -7.1537295578   -5.186706674   -0.4470693904
H  -7.0055975389   -6.8480265465  -1.0505869231
H  -8.272094354    -1.4977003926  -0.687435318
H  -9.322785239    -2.0120389547  -2.0212848088
H  -4.8626847447   -3.2394094689  -5.0684537899
H  -9.0388314178   -6.5582745456  -0.0692765104
H  -9.4471265941   -4.207072393   -0.704694716
H  -9.4470752746   -4.3012943745  2.5825348419
H  -11.0230807852  -3.6213070019  2.2017979669
H  -10.3693961093  -4.926812589   1.1888324902
```

calc_**2c** conf_30

```
C  -7.3809918266   -2.0626296438  -3.4377893032
C  -6.5628873709   -2.1131313231  -2.1220634429
C  -6.8187727086   -3.520841033   -1.5362567103
N  -7.7709106746   -4.1673339757  -2.4551011196
C  -7.6476077319   -3.5310099205  -3.7672587235
C  -7.5687775561   -5.5851321373  -2.7482730618
C  -6.4247239581   -5.6370471639  -3.7842668602
C  -6.5381890807   -4.305691528   -4.5408587856
O  -6.684625261    -1.367357056   -4.4611167639
O  -5.1932918234   -1.8603082803  -2.3734616026
C  -7.3657704583   -6.4833135776  -1.5418758206
C  -7.322304903    -3.5171533419  -0.0695568137
O  -5.2741356265   -3.6516736043  -4.525272813
O  -8.4634163387   -6.4830943647  -0.6427332384
N  -8.7538161112   -3.5710927063  0.0834154073
O  -9.0906564943   -1.3892890084  -0.4464357626
C  -9.5390037381   -2.5053543014  -0.2110039051
C  -11.0289465545  -2.7764442775  -0.2106050544
H  -8.5914809601   -3.6579215936  -4.3126921315
H  -8.3347585006   -1.5640676802  -3.2412073629
H  -6.8938136298   -1.3335573092  -1.4375352597
H  -5.8366563809   -4.0214615216  -1.5317890937
H  -8.4896077581   -5.9237980443  -3.251591374
H  -6.4887173958   -6.5091271885  -4.4397468727
H  -5.4461359534   -5.6660628335  -3.2927551071
H  -6.841841131    -4.4597114749  -5.583763247
H  -5.95316832     -0.9256668888  -3.9897822927
H  -4.8871893717   -2.5965276179  -2.9399810473
H  -6.5000277489   -6.1626160019  -0.9574835641
H  -7.1525301058   -7.5025110818  -1.8937329357
H  -6.9202128924   -4.3793336657  0.4676617492
H  -6.9477105058   -2.6206060086  0.4306968622
H  -5.4661537645   -2.779223621   -4.9199516583
H  -9.2261037365   -6.8386168389  -1.1171659434
H  -9.1417015954   -4.5006776291  -0.0043476393
H  -11.5314052988  -1.9728025198  0.3305062703
H  -11.3928750177  -2.7550496637  -1.2419649218
H  -11.2960817162  -3.7357635731  0.2391399437
```

calc_**2c** conf_31

```
C  -7.4623705903   -2.0697959916  -3.3816244403
C  -6.617381178    -2.1942521192  -2.095276824
C  -6.8833055286   -3.6083230216  -1.5573032226
```

N	-7.8061685918	-4.2477798705	-2.5243310932
C	-7.6890918995	-3.5261719117	-3.7961776782
C	-7.5015095532	-5.6374202822	-2.9094767818
C	-6.2962511106	-5.5467685347	-3.8472530722
C	-6.5389473571	-4.2247812706	-4.6105799138
O	-6.8686819311	-1.242165661	-4.3521577918
O	-5.2256165451	-2.0939941635	-2.4122111543
C	-7.443256594	-6.5458754203	-1.6992617119
C	-7.3768492621	-3.6618907292	-0.0902730426
O	-5.3626631542	-3.4510659664	-4.7768580825
O	-8.6356125171	-6.3953261805	-0.9512367369
N	-8.8050405843	-3.5975541545	0.1273549031
O	-9.0549191796	-1.4325277698	-0.4805006075
C	-9.5409671959	-2.4734493983	-0.0594218779
C	-11.0114076884	-2.5853343681	0.2811772495
H	-8.6235245776	-3.6379162384	-4.3570014706
H	-8.4213622419	-1.6235378545	-3.1109513168
H	-6.8832190639	-1.4233014537	-1.3704675226
H	-5.8999221654	-4.1024585773	-1.5454815786
H	-8.3684768694	-5.9671798655	-3.5007054561
H	-6.2031805082	-6.4087505809	-4.5122565513
H	-5.3583287618	-5.4672021259	-3.2855268932
H	-6.8838330405	-4.4212972817	-5.6283020835
H	-6.2397590625	-1.8103111042	-4.8383246988
H	-5.1530707073	-1.2630718451	-2.9120851204
H	-6.5609655918	-6.3158013185	-1.0822848906
H	-7.3547505667	-7.591432234	-2.0117883855
H	-7.0513228634	-4.5997913119	0.3621416805
H	-6.8859704748	-2.8484149805	0.4550963862
H	-5.0568637077	-3.1807025321	-3.8835962159
H	-8.9952221064	-5.5386757464	-1.2396654376
H	-9.2439334364	-4.4127377932	0.5239430905
H	-11.5956052171	-2.3962813378	-0.6227313122
H	-11.3006003429	-3.555606958	0.6920710826
H	-11.2640482334	-1.8030520468	1.0003285728

calc_**2c** conf_32

C	-7.1541395484	-2.1317418374	-4.0763696303
C	-6.3604573686	-1.7811091159	-2.7733991931
C	-6.909816687	-2.7358227152	-1.7033645708
N	-7.1338897709	-3.9085193476	-2.5179300753
C	-7.6972048592	-3.5526248217	-3.8023245325
C	-7.5307168073	-5.2627113356	-2.207324096
C	-7.4938488846	-5.9131582312	-3.6262467498
C	-7.1853602651	-4.7552804612	-4.5981834772
O	-6.3337175672	-2.0028799143	-5.2178579909
O	-4.9984431894	-2.0747489339	-2.9805741545
C	-6.6085153611	-5.9550349914	-1.2244176892
C	-8.1403240766	-2.1588218122	-0.9973887453
O	-5.7821579679	-4.6108210463	-4.8040437276
O	-6.7990532604	-5.3869819323	0.0653268115
N	-8.6854881142	-3.113928993	-0.0616822483
O	-10.768759137	-2.2236970786	-0.0858226743
C	-9.9897816201	-3.0779077717	0.3124913069
C	-10.4143338374	-4.1714102591	1.2726496389
H	-8.802527378	-3.5483922025	-3.8080158804
H	-7.9844306913	-1.4336215085	-4.206705072
H	-6.4900163312	-0.7273331941	-2.4904128663

H	-6.1299374672	-2.9405847456	-0.9635842952
H	-8.5556495089	-5.3188041554	-1.8042891699
H	-8.4523585813	-6.3836758991	-3.8606071974
H	-6.7154321191	-6.6765562409	-3.7180736167
H	-7.6340403415	-4.8882931799	-5.58323868
H	-5.9523584804	-2.8896475321	-5.3822176849
H	-4.8329128519	-1.7816589626	-3.8944935298
H	-5.5696374292	-5.8318899938	-1.5597081232
H	-6.8437516172	-7.0284074515	-1.2069845202
H	-7.8559553759	-1.2275457496	-0.4872883164
H	-8.9310272423	-1.8940396783	-1.7075772898
H	-5.44618075	-4.3117109508	-3.9343676281
H	-6.1398085247	-5.7527683173	0.6668249835
H	-8.0829081806	-3.8634142766	0.2559483978
H	-9.6643427572	-4.9567817117	1.3959456949
H	-10.6245239641	-3.7262615363	2.2491282563
H	-11.3451920859	-4.6124121147	0.9101783358

calc_**2c** conf_33

C	-7.4618764893	-2.1573976316	-3.3468940611
C	-6.7068490579	-2.3092936793	-2.002853161
C	-7.0630026165	-3.724757727	-1.5021624839
N	-7.9203602384	-4.3299133482	-2.5470054856
C	-7.7065601589	-3.5985358308	-3.8004974616
C	-7.6572621947	-5.7263750109	-2.9156702235
C	-6.4376077381	-5.6778404867	-3.8559639214
C	-6.5489895635	-4.319438178	-4.5588436606
O	-6.7306367661	-1.3754666009	-4.2747041453
O	-5.3225864989	-2.0996046404	-2.1843589198
C	-7.5321147493	-6.7008195259	-1.7520227477
C	-7.8053334087	-3.6962656302	-0.1644380845
O	-5.2965438543	-3.6489377073	-4.4712820854
O	-7.4141899849	-8.0308822197	-2.2109572018
N	-9.0923224787	-3.0623654045	-0.3032856155
O	-8.6948019171	-1.3715301719	1.1599653294
C	-9.437616329	-1.9272871708	0.3651605047
C	-10.8276355621	-1.4025908667	0.0705993069
H	-8.6112163564	-3.6769142661	-4.4152014958
H	-8.4327745169	-1.6832010514	-3.1633295911
H	-7.0207711626	-1.5480396005	-1.2855141818
H	-6.1168289046	-4.2679400696	-1.3632965597
H	-8.5249161347	-6.0603187121	-3.5000509075
H	-6.4091656279	-6.515286777	-4.5569706198
H	-5.5000070432	-5.6841259857	-3.2861787023
H	-6.8118791914	-4.4331665385	-5.6172651662
H	-6.0007308498	-1.0001674656	-3.7471919296
H	-5.0229926303	-2.7880981278	-2.814243398
H	-8.4402957915	-6.669704534	-1.144653478
H	-6.68712389	-6.4313572321	-1.1013824925
H	-7.9311130701	-4.7144618236	0.2218590696
H	-7.2419386333	-3.1245014862	0.576159377
H	-5.4774332178	-2.7633840141	-4.8377484612
H	-6.537830697	-8.1316789214	-2.602327389
H	-9.6923526833	-3.4336745257	-1.0238731712
H	-10.757146648	-0.3429931888	-0.1837348348
H	-11.3407251484	-1.9341748173	-0.734803491
H	-11.4294681967	-1.4785090318	0.9799615407

calc_**2c** conf_34

```
C  -6.9800979585   -2.0270218101  -3.944401896
C  -6.2396115153   -1.7884313567  -2.5900160104
C  -6.9188754728   -2.7495068724  -1.599476938
N  -7.0145458077   -3.8905828596  -2.4916618782
C  -7.5683131966   -3.4452039842  -3.7576343269
C  -7.5015708954   -5.234414937   -2.2717689099
C  -7.4142461904   -5.8241959819  -3.70798076
C  -7.2031811268   -4.6264746998  -4.6609285118
O  -6.079429313    -1.8837456473  -5.0269819159
O  -4.8852154066   -2.1694705708  -2.7677737951
C  -6.6889208679   -6.0158271669  -1.263070739
C  -8.2229260338   -2.2075459862  -1.0141617298
O  -5.846301993    -4.5980072145  -5.0436615443
O  -6.9296252882   -5.4759918404  0.0319893724
N  -8.7915461927   -3.1642690745  -0.0929175203
O  -10.7823784544  -2.0979624967  0.0236602807
C  -10.0638112421  -3.0287105033  0.358802966
C  -10.5295284025  -4.1074777651  1.3157844031
H  -8.6723487794   -3.3752010245  -3.7178208726
H  -7.7867083472   -1.3021670893  -4.0857688757
H  -6.2344695385   -0.7435871905  -2.2749434405
H  -6.2260128105   -2.9691960209  -0.7812113724
H  -8.5531936108   -5.2392065989  -1.9378787629
H  -8.318744188    -6.3860334993  -3.9516584654
H  -6.558574669    -6.4963872164  -3.8197370195
H  -7.8565037836   -4.6881139288  -5.5411805297
H  -5.2052425966   -1.8112626499  -4.5939842456
H  -4.9322497754   -3.1396814802  -2.881256031
H  -5.6259989946   -5.9441792525  -1.5295217525
H  -6.9875908573   -7.072659298   -1.2991068223
H  -8.0205723525   -1.2554445383  -0.5042125783
H  -8.9614890307   -1.9871720753  -1.7924480413
H  -5.7266858784   -3.7219979764  -5.4524470349
H  -6.3599369877   -5.9245850951  0.6678735295
H  -8.2221990775   -3.9527286756  0.1910939401
H  -10.6737892361  -3.6663030619  2.3058265998
H  -11.5009256187  -4.4770405144  0.9807177772
H  -9.8326385099   -4.9452120463  1.3988634517
```

calc_**2c** conf_35

```
C  -7.3759240601   -2.0810828614  -3.4076500372
C  -6.5893505623   -2.1851707302  -2.0788653248
C  -6.8630108014   -3.6175928102  -1.5624825904
N  -7.7790486672   -4.2388599023  -2.5413913356
C  -7.6042579231   -3.5345489384  -3.8184454502
C  -7.5123654399   -5.6383875575  -2.9143628624
C  -6.2869914938   -5.5974708443  -3.8353936388
C  -6.4395103611   -4.2568640349  -4.5724404865
O  -6.6730842985   -1.318314842   -4.3771295605
O  -5.2144900407   -1.9227975057  -2.2809989783
C  -7.496274876    -6.5483498043  -1.7046483459
C  -7.363593603    -3.679138442   -0.0984280595
O  -5.2036331616   -3.5552164519  -4.5254282523
O  -8.6981415251   -6.3767764198  -0.9772083692
N  -8.7933564096   -3.6077658565  0.1141651024
O  -9.0492027633   -1.4398412136  -0.4898949867
```

C	-9.5295327444	-2.4827829337	-0.0677635117
C	-10.9977891803	-2.5953545736	0.2834811101
H	-8.5185428031	-3.6493986102	-4.412134999
H	-8.3448667352	-1.6143508514	-3.2071414777
H	-6.9396373759	-1.4370746557	-1.3701949905
H	-5.8811501695	-4.1158518935	-1.5483187444
H	-8.3797476056	-5.943628789	-3.5179613929
H	-6.2388059699	-6.4538912956	-4.5118602963
H	-5.3526955977	-5.5736452699	-3.2646874419
H	-6.7179059599	-4.4088653345	-5.622613526
H	-5.9618572191	-0.8859636969	-3.8688705826
H	-4.8951491785	-2.613343118	-2.8973596438
H	-6.619983808	-6.3378467633	-1.0724129276
H	-7.4238571802	-7.594951343	-2.0180610788
H	-7.0457242192	-4.6202084599	0.3531500351
H	-6.869146689	-2.8712496567	0.4524270604
H	-5.4219954222	-2.6773957539	-4.8936419444
H	-9.0328527558	-5.5089014506	-1.2610212141
H	-9.2331216269	-4.4203171561	0.5152560843
H	-11.5888876961	-2.3759055977	-0.6088257156
H	-11.2903771694	-3.5746454519	0.6698636482
H	-11.2381369076	-1.83224913	1.0272947254

calc_**2c** conf_36

C	-7.2108539764	-2.31735667	-4.3714986137
C	-6.719121814	-1.8840660861	-2.9715899347
C	-7.2685248328	-2.9563582546	-2.0054586046
N	-7.8943099172	-3.9960886462	-2.8554923028
C	-7.4209932338	-3.824221727	-4.2331736691
C	-7.6181405587	-5.4129819806	-2.5990123425
C	-6.2256988638	-5.6756811516	-3.2000087799
C	-6.1690482668	-4.7446079037	-4.4140439515
O	-6.2925667825	-1.9470788267	-5.3867635416
O	-5.314001351	-1.7477559057	-2.9565640671
C	-7.8509788646	-5.9401068229	-1.1936600362
C	-8.2585638769	-2.310276213	-1.0331696447
O	-4.9265697816	-4.0507929278	-4.4093576408
O	-6.9595936921	-5.4649032911	-0.1965125568
N	-8.690388908	-3.19210413	0.0214410084
O	-10.7671449995	-2.2935592273	0.2009898596
C	-9.92686524	-3.0962429241	0.5784496405
C	-10.1975974147	-4.0650146517	1.712853202
H	-8.1971108105	-4.180281382	-4.921614731
H	-8.1802510499	-1.8505222784	-4.5779206854
H	-7.0988399145	-0.891044498	-2.7180332818
H	-6.420022012	-3.3488231361	-1.4297394046
H	-8.3477819744	-5.9610357696	-3.2148175746
H	-6.0563376871	-6.7256363597	-3.4532279384
H	-5.4326980937	-5.3645239246	-2.5094495926
H	-6.2534418689	-5.3047495143	-5.3532181507
H	-5.6475719353	-1.3760382924	-4.9296345689
H	-4.9497658093	-2.6241195825	-3.2046172363
H	-7.8252794131	-7.037783246	-1.2314666504
H	-8.8468369045	-5.6392446457	-0.8663935755
H	-7.7787257969	-1.4177612944	-0.6053463492
H	-9.1516131118	-1.9782234154	-1.5684871216
H	-5.0178668793	-3.3966070191	-5.1271271862
H	-6.0791172847	-5.8186873865	-0.3717634559

H	-8.0094279551	-3.8367464132	0.3998771212
H	-11.1291330539	-4.596022755	1.5044608094
H	-9.3959548013	-4.7906273657	1.8729445786
H	-10.3492612694	-3.4963243813	2.6341469693

calc_**2c** conf_37

C	-7.2611510423	-2.4007147704	-4.256152297
C	-7.2781414032	-2.1372810516	-2.7276263942
C	-7.586022363	-3.5071014047	-2.0829893357
N	-7.8360563515	-4.4346717795	-3.2040631002
C	-7.0943489522	-3.9132146201	-4.3618246421
C	-7.2799577245	-5.7954519722	-3.0652668001
C	-5.7652493112	-5.646727364	-3.2809579678
C	-5.639962414	-4.4663251797	-4.2525707235
O	-6.2444018934	-1.6627292984	-4.9110267949
O	-6.0622599087	-1.5730203982	-2.28092456
C	-7.7221298923	-6.5078114528	-1.804018404
C	-8.7698305778	-3.4196928763	-1.1268059857
O	-4.7097459141	-3.522584272	-3.7257191301
O	-9.1177621299	-6.6981790972	-1.7775645105
N	-8.4467737092	-2.5029586781	-0.0536642003
O	-10.4498702057	-2.7541666634	0.9536968581
C	-9.3476715141	-2.2308146456	0.9287022025
C	-8.8926583798	-1.2466281344	1.9857874205
H	-7.5462826367	-4.3094663525	-5.2779156023
H	-8.2340844854	-2.1270660366	-4.679322161
H	-8.044215677	-1.4005134052	-2.4740535996
H	-6.695138248	-3.7962525255	-1.5013439237
H	-7.6970021342	-6.3651552354	-3.9066026164
H	-5.3074279851	-6.5626933039	-3.6610621205
H	-5.2499816295	-5.3843786213	-2.3505918158
H	-5.2872383093	-4.7899011225	-5.2387473496
H	-5.9672279003	-0.9912942551	-4.2620123076
H	-5.3815349581	-2.2561200873	-2.4575758179
H	-7.3713144973	-5.9607465481	-0.914373739
H	-7.262499079	-7.5001190597	-1.7680450568
H	-9.6541844511	-3.0678745051	-1.6724957194
H	-9.026564362	-4.3970512523	-0.7071729253
H	-4.7420319237	-2.7855525055	-4.3630372399
H	-9.5145709518	-5.9116767785	-2.17776821
H	-7.5565032427	-2.0291794015	-0.0717363717
H	-8.9192634718	-1.7414013013	2.9596674974
H	-7.8891551748	-0.8472680263	1.8185692181
H	-9.603785195	-0.4182160177	2.023608226

calc_**2c** conf_38

C	-7.2258213663	-2.3167433429	-4.3449165361
C	-7.4405085771	-1.9375880939	-2.8558954756
C	-7.9877650848	-3.2182464903	-2.1954582757
N	-8.0515404087	-4.2366585298	-3.2602985626
C	-7.1187755354	-3.8416364438	-4.3197316378
C	-7.598439464	-5.5884097782	-2.9032190109
C	-6.0615287267	-5.5190350397	-2.9035407971
C	-5.7312618808	-4.4607067057	-3.9610537919
O	-6.1014366004	-1.6540181168	-4.8934326343
O	-6.240627555	-1.4581024809	-2.2872563773
C	-8.2146701414	-6.158422364	-1.6398521507

C	-9.3757621471	-3.0242745429	-1.5754859129
O	-4.8097221243	-3.5196423096	-3.4109041199
O	-7.6829332168	-7.4622700145	-1.4883397063
N	-9.3604374629	-2.159654707	-0.4150964013
O	-8.6657774234	-3.7919605945	0.9916725321
C	-8.957182576	-2.6192628906	0.8003554859
C	-8.8909160241	-1.5877422503	1.9050904587
H	-7.4523751781	-4.2810448758	-5.2673402346
H	-8.1167428953	-2.0442454317	-4.9225208766
H	-8.1447495005	-1.1045683126	-2.7686209891
H	-7.2985030992	-3.4904031131	-1.3838087046
H	-7.9124165943	-6.2421897886	-3.728239654
H	-5.6016107291	-6.48846517	-3.0978024941
H	-5.6874353887	-5.1690429334	-1.9352669893
H	-5.2786122843	-4.9052223229	-4.8553421137
H	-5.8301658371	-1.02990395	-4.1936932871
H	-5.6083571811	-2.2078833754	-2.3318094509
H	-9.3089845343	-6.1885112094	-1.7536290058
H	-7.9869442593	-5.5302369059	-0.7691750238
H	-10.0587674484	-2.608574779	-2.3219769966
H	-9.7772742844	-3.9899341278	-1.2651365453
H	-4.7137598881	-2.8475147181	-4.1089635365
H	-7.9341624313	-7.7785635918	-0.6129116504
H	-9.4928549596	-1.1695094474	-0.5397634566
H	-9.3605847852	-0.6352197993	1.6475005883
H	-9.3733921826	-1.9971107298	2.7944356151
H	-7.842200224	-1.4054807231	2.1554277192

calc_**2c** conf_39

C	-6.5273232674	-2.382541197	-3.9497267023
C	-7.5761404072	-1.902439148	-2.9532343645
C	-7.8981900546	-3.1888726589	-2.1735782395
N	-7.9393241204	-4.2028635096	-3.2276192539
C	-7.0979960064	-3.7446845481	-4.3625767013
C	-7.5151851222	-5.5788080855	-2.9185506667
C	-6.034030086	-5.6292233053	-3.2974398796
C	-6.0554776041	-4.8608245318	-4.6126757917
O	-5.3280782715	-2.4539527304	-3.201459741
O	-7.1356185209	-0.8330588007	-2.1527850744
C	-7.8295178802	-6.067834899	-1.5168513428
C	-9.2131280932	-3.0769261386	-1.4052315357
O	-4.8158925386	-4.2910968216	-5.0280988401
O	-7.3539715802	-7.3986099169	-1.4467393527
N	-9.1170922139	-2.0646136373	-0.3777021918
O	-9.9504263502	-3.3709461145	1.2842635222
C	-9.4644634184	-2.3115752641	0.9126213798
C	-9.2270708917	-1.1658159271	1.8751124125
H	-7.7154824432	-3.6046478264	-5.2585390638
H	-6.4087277181	-1.7093852383	-4.8077524305
H	-8.4723991399	-1.5623204862	-3.4832281168
H	-7.0772481782	-3.3420737842	-1.4541715954
H	-8.0596647874	-6.249446276	-3.599593393
H	-5.649079345	-6.6480509542	-3.3833420319
H	-5.4380111431	-5.0787429523	-2.5606087383
H	-6.4222734512	-5.5191558181	-5.4115127705
H	-4.7014432243	-2.954860694	-3.7530799191
H	-6.2020992084	-1.0291563475	-1.9678482749
H	-8.9132782854	-6.0255548491	-1.3464091553

H -7.3426989676   -5.4336184477 -0.7630508753
H -10.0186223743 -2.8496592539 -2.1162936669
H -9.4794601514   -4.0048583162 -0.8979231697
H -4.1830085464   -5.0109363469 -5.1437818024
H -7.5801291147   -7.7439832624 -0.5751355218
H -8.6123953324   -1.2205681534 -0.6130018939
H -8.726239764   -0.3058499075 1.4237282846
H -10.1899573687 -0.8414126658 2.2780115435
H -8.6308550293   -1.5300311859 2.7148049549

calc_**2c** conf_40

C -7.349733894   -2.2651927463 -3.9184157945
C -7.1616530817 -2.2221266465 -2.38540561
C -7.7108963462 -3.5592188792 -1.8783430779
N -8.0045112209 -4.3748685853 -3.0769021314
C -7.3501037744 -3.7653938873 -4.2352207281
C -7.5334092363 -5.7634488105 -3.0923202298
C -6.04153011   -5.6742936778 -3.4519422121
C -5.9553265133 -4.4653627834 -4.4035639354
O -6.4026363815 -1.4888452232 -4.6080947237
O -5.7710635305 -2.1355116523 -2.0669326608
C -7.8550370269 -6.5763173434 -1.8456459187
C -8.9963700942 -3.3974308404 -1.0574516773
O -4.8304508832 -3.6381341509 -4.149115957
O -7.4794264063 -7.9285856531 -1.9975509547
N -8.7932251833 -2.7301184878 0.2086039977
O -9.5626708037 -0.7054517434 -0.4681015374
C -9.1093755458 -1.4206437427 0.4130733496
C -8.8634161073 -0.901439954   1.813566354
H -7.9438555503 -3.9725191179 -5.132813357
H -8.3366639385 -1.8580362956 -4.1565145124
H -7.6840074538 -1.3705520609 -1.9401293565
H -6.9338344647 -4.0060227996 -1.2400895677
H -8.0568391643 -6.2593458513 -3.9208564394
H -5.6431943515 -6.5884610964 -3.8998925319
H -5.4434069347 -5.4543791536 -2.5571852116
H -5.8314569881 -4.7876554508 -5.4396761101
H -5.6221516637 -2.0650970537 -4.7273740651
H -5.4642689834 -1.3454159316 -2.5429316123
H -8.935286975   -6.5811385759 -1.6795849839
H -7.3814549816 -6.1351342745 -0.9552720967
H -9.7182939604 -2.8064440438 -1.6241816716
H -9.4415378403 -4.3809222817 -0.8798604354
H -4.9348948331 -3.291563618   -3.2371794112
H -6.5155205174 -7.9634897529 -2.0319330512
H -8.396049321   -3.2545271423 0.9710439263
H -9.8032434517 -0.5104733858 2.2101657331
H -8.4706392477 -1.6515006701 2.5044968828
H -8.1615632393 -0.0659366362 1.7595313193

calc_**2c** conf_41

C -6.4019146377   -2.4279168373 -3.9246330969
C -7.3707830018   -1.9169050618 -2.8652997495
C -7.7887232369   -3.2118545855 -2.1445293394
N -7.9292561399   -4.1673891197 -3.244970153
C -7.1047203391   -3.7076676673 -4.3904361466
C -7.5967372671   -5.5838582296 -3.0192538223

C	-6.1494641137	-5.7297930047	-3.492426236
C	-6.1723188641	-4.8852105182	-4.7592154767
O	-5.1907653503	-2.6506489284	-3.2273897778
O	-6.80834341	-0.9415228786	-2.0222268515
C	-7.8533554733	-6.126826541	-1.6179062628
C	-9.0809088297	-3.0289607947	-1.3526048661
O	-4.9114595165	-4.3898948621	-5.2032438631
O	-7.4844176522	-7.4897457175	-1.5366999248
N	-8.8933507661	-2.051337277	-0.3044737327
O	-10.145960889	-3.1570190027	1.2308925972
C	-9.4340044356	-2.2089140173	0.9314595435
C	-9.0964341989	-1.1198700297	1.9294327586
H	-7.7468681634	-3.4586769376	-5.2447214153
H	-6.2508493636	-1.7182111749	-4.7472562222
H	-8.2470377399	-1.4651664716	-3.3426924619
H	-6.9739698066	-3.4689061169	-1.4486932563
H	-8.2342109597	-6.1734338445	-3.7008236145
H	-5.8514102764	-6.7687188331	-3.653289895
H	-5.472863322	-5.2723211837	-2.7618861793
H	-6.6295053313	-5.4615289901	-5.5749378586
H	-4.6238869094	-3.1541368257	-3.8375880585
H	-5.8909561246	-1.2302176263	-1.8836254005
H	-8.9042107417	-5.983393384	-1.3374989624
H	-7.2386146981	-5.6086560596	-0.8782802765
H	-9.8776060158	-2.7289445387	-2.0473542778
H	-9.4086815393	-3.950135844	-0.8681844981
H	-4.3456862109	-5.1481192981	-5.3958605814
H	-8.1405360744	-7.9997856181	-2.0278578906
H	-8.2655001999	-1.2823143002	-0.4974060465
H	-10.0262556787	-0.6684099511	2.2837347535
H	-8.6078669867	-1.5755481942	2.7941389019
H	-8.4515657357	-0.3350397342	1.5266076397

calc_**2c** conf_42

C	-6.7431511087	-2.2700889027	-3.7552381991
C	-7.7155552337	-2.0052957416	-2.5810010318
C	-7.9674865523	-3.3918187916	-1.9699500791
N	-7.9162103803	-4.2747839699	-3.1223243525
C	-7.0947155601	-3.6950610579	-4.1983048678
C	-7.6508554361	-5.7143230621	-3.0022552688
C	-6.7802645073	-6.0230753348	-4.2357301275
C	-5.9926077929	-4.7358901413	-4.4611021582
O	-5.4164562237	-2.1351109155	-3.2861112249
O	-7.1218123182	-1.0776168936	-1.7025071575
C	-6.9683904191	-6.1689410093	-1.7050966335
C	-9.3453022351	-3.5387160398	-1.3039600827
O	-4.9426937812	-4.7105206721	-3.5253598838
O	-6.84048413	-7.5770386845	-1.6722361124
N	-9.7831959068	-2.4780936623	-0.4021826998
O	-7.8443836495	-2.1270672188	0.7174987965
C	-9.0530109868	-1.9218514708	0.5859056417
C	-9.7885821045	-0.9902820469	1.5200725997
H	-7.6949765518	-3.626893467	-5.1187926765
H	-6.9015144668	-1.553933753	-4.5707537994
H	-8.6579131482	-1.5986978794	-2.9725454009
H	-7.1768513285	-3.5889040137	-1.2336977615
H	-8.6020087116	-6.2695253073	-3.0716433699
H	-7.4073596305	-6.2378843023	-5.1074364467

H -6.1241440264   -6.8777597844 -4.0600348689
H -5.6109856089   -4.6545804597 -5.4900410377
H -5.504444627   -1.5548170548 -2.5024974875
H -7.1926078042   -1.4268621672 -0.7895896455
H -7.5228265217   -5.8120044227 -0.8234200162
H -5.9561922687   -5.767516531   -1.6621110324
H -10.0770586039 -3.5831934381 -2.1140673778
H -9.3842465638   -4.4991282207 -0.7748533884
H -4.7583801458   -3.7704593293 -3.3448034462
H -7.7301844592   -7.951766971   -1.7060624621
H -10.7675835152 -2.2643571107 -0.4203300967
H -9.6375205328   -1.3392782114 2.5441365836
H -9.3406928468   0.0036585188   1.4468862438
H -10.8603503118 -0.9135204795 1.3235403283

calc_**2c** conf_43

C -7.2202292355 -2.2259283371 -3.7564117622
C -7.1241717724 -2.2907843261 -2.2116257614
C -7.7234569472 -3.6644254383 -1.8422717694
N -8.024357911   -4.3479025894 -3.1156167363
C -7.268919727   -3.6929990511 -4.1881393013
C -7.6433848472 -5.758102241   -3.2404766526
C -6.1363628772 -5.7547123129 -3.5461371778
C -5.9122903797 -4.4541594544 -4.3248533255
O -6.1448564947 -1.4910218044 -4.31611144
O -5.7874335374 -2.1126493135 -1.7901615231
C -8.060602245   -6.653884458   -2.0828976358
C -9.0094899875 -3.5396003848 -1.0163638738
O -4.8019452259 -3.762043974   -3.7620524115
O -7.670479328   -7.9938449303 -2.2984294848
N -8.8002006738 -2.9440706271 0.2835485317
O -9.6106525305 -0.8980678383 -0.2708323742
C -9.1205970233 -1.6487282571 0.5599889927
C -8.8230228281 -1.1910756147 1.9714997419
H -7.818273961   -3.8116562121 -5.1300110107
H -8.1650139649 -1.7498773741 -4.0410040953
H -7.6872084298 -1.4765615094 -1.7523888116
H -6.9651325532 -4.1936515106 -1.2439943747
H -8.1723813321 -6.1287638578 -4.1337069704
H -5.8086006555 -6.6465755374 -4.0823708346
H -5.5564358261 -5.7037895991 -2.6176477788
H -5.7014994457 -4.6473018958 -5.3837779442
H -5.7005794632 -1.0940688626 -3.5438116466
H -5.2687350322 -2.8316519373 -2.2102605112
H -9.1449008343 -6.5711780087 -1.9194212788
H -7.5597513493 -6.3524039624 -1.1586342302
H -9.7202371162 -2.9054266647 -1.5494212855
H -9.4716930924 -4.5238947383 -0.8930604236
H -4.7986740976 -2.9037168349 -4.2249323947
H -8.1671335402 -8.3250745174 -3.0573878506
H -8.3539261578 -3.4934938568 0.999983865
H -8.0501946944 -0.4190759939 1.9339940185
H -9.7228474488 -0.7317105813 2.3856836831
H -8.490327434   -1.9911255928 2.6375138381

calc_**2c** conf_44

C -7.4105200644   -2.272158779   -4.2110713398

```
C  -7.5969405271   -2.0264583443  -2.695966258
C  -8.0907706636   -3.3827112286  -2.1362154037
N  -8.0671470348   -4.3356180886  -3.2681934239
C  -7.1819574344   -3.7815253161  -4.297686964
C  -7.4644019342   -5.6446586406  -2.9761997879
C  -5.9472964955   -5.4015843126  -2.9541325901
C  -5.728895226    -4.2851553465  -3.9974408394
O  -6.4645376632   -1.4790375848  -4.8555795252
O  -6.364379664    -1.6581564095  -2.1066797396
C  -8.033966393    -6.3544080319  -1.7560527729
C  -9.4865514948   -3.3355815204  -1.490677766
O  -4.8137990479   -3.2877008435  -3.5812341153
O  -7.4185367001   -7.6062793214  -1.5388686826
N  -9.4584705175   -3.2636680087  -0.0357874051
O  -7.9888975202   -1.5431680376  0.1588233616
C  -8.7429941492   -2.3640325511  0.6766551365
C  -8.9158835697   -2.418935568   2.1777536957
H  -7.4879796506   -4.166207115   -5.2770613398
H  -8.3734750676   -2.0568468553  -4.6856959357
H  -8.3231941901   -1.2286107865  -2.5152497076
H  -7.3759747306   -3.6739515216  -1.3550959775
H  -7.6973611428   -6.275914308   -3.8491698632
H  -5.3667768836   -6.3037801981  -3.1555842808
H  -5.6308012385   -5.0365891881  -1.9697475877
H  -5.2920216522   -4.6887098505  -4.9139071787
H  -5.5982085476   -1.7867295623  -4.5327087395
H  -6.5906902375   -1.4841581473  -1.1747987338
H  -9.1248991613   -6.4573223161  -1.8659543322
H  -7.846051415    -5.7769716668  -0.8462246551
H  -10.0625151968  -2.4941683764  -1.8955977616
H  -10.0261027184  -4.246568391   -1.7528628353
H  -5.1853381953   -2.8287106287  -2.798214592
H  -7.6050198181   -8.161470015   -2.3067245718
H  -10.0797873253  -3.8732468579  0.4702516991
H  -7.934246757    -2.5499125338  2.6384342645
H  -9.3076621494   -1.4573231684  2.5181661195
H  -9.5799478229   -3.2149705801  2.5223004289
```

calc_**2c** conf_45

```
C  -7.1931761584  -2.2269001034  -3.7556701947
C  -7.1679707944  -2.3582491583  -2.2111812899
C  -7.7306803164  -3.7673233643  -1.9288030746
N  -7.9952667976  -4.3856124918  -3.2428884876
C  -7.2054041369  -3.6724720865  -4.2505222303
C  -7.6059194046  -5.7868862944  -3.4476198788
C  -6.0867614973  -5.7592896782  -3.6849481797
C  -5.8322369558  -4.4079341964  -4.3590426016
O  -6.0989610767  -1.46053802    -4.2309914694
O  -5.861950482   -2.1540915079  -1.7129635441
C  -8.0823731789  -6.8065979602  -2.4220672038
C  -9.0305839435  -3.711110212   -1.1169342448
O  -4.7685677245  -3.7396622297  -3.6866245086
O  -7.4226581314  -6.7619741093  -1.1698986785
N  -8.8611178253  -3.1304918733  0.1955020366
O  -9.8772099656  -1.1637372866  -0.3085370093
C  -9.2798905953  -1.8652369731  0.4929272216
C  -8.9391412849  -1.3809425682  1.885260701
H  -7.7108705962  -3.7580443449  -5.2201893116
```

H	-8.1279195023	-1.7452554459	-4.0626932035
H	-7.7811449338	-1.5834476837	-1.7480850929
H	-6.9663152774	-4.3127708485	-1.3562380466
H	-8.0962731693	-6.0847564479	-4.3869004408
H	-5.7373479287	-6.6075447094	-4.2789715427
H	-5.5561565493	-5.7830289352	-2.7284333844
H	-5.5569500733	-4.5259564179	-5.4140931579
H	-5.7039385063	-1.0824493656	-3.4232088071
H	-5.3061873056	-2.8544285838	-2.1168206907
H	-7.8785014001	-7.8007115194	-2.8317567515
H	-9.1707752154	-6.7239158126	-2.304861898
H	-9.7589991179	-3.092703461	-1.644217429
H	-9.4731045806	-4.7116253685	-1.0262527569
H	-4.7444842081	-2.8599228423	-4.1065842612
H	-7.9030694828	-6.1502695842	-0.602295916
H	-8.2872071438	-3.6106245973	0.8704861491
H	-9.8132011712	-0.8788983554	2.3030240855
H	-8.6216004323	-2.1768021414	2.5636684052
H	-8.1370831362	-0.6407934215	1.8144266875

calc_**2c** conf_46

C	-7.3818274985	-2.3993469581	-4.1475722005
C	-8.008644803	-2.2392941148	-2.7282103
C	-7.8901860901	-3.6190477458	-2.058401928
N	-7.8688735329	-4.5412094248	-3.2153101175
C	-7.0138625527	-3.8845707232	-4.2236810626
C	-7.2109738062	-5.8570693293	-3.0747955384
C	-5.6942384059	-5.6134285696	-3.1461890147
C	-5.5458226836	-4.2696834433	-3.8750551106
O	-6.2888824416	-1.5190779208	-4.3223037712
O	-7.3656513079	-1.1886664125	-2.043732473
C	-7.6874866931	-6.6718520648	-1.8940659724
C	-9.0917113979	-3.8969292233	-1.1457163627
O	-4.9235878419	-3.3693192532	-2.9863046403
O	-9.0815062204	-6.9028187211	-1.947189691
N	-9.4391306303	-2.811520206	-0.2339143789
O	-7.3569262727	-2.2744526516	0.4914486597
C	-8.5791418331	-2.1484787228	0.5700193306
C	-9.2008860107	-1.1916992793	1.5610383562
H	-7.2508413632	-4.3109542938	-5.2061745218
H	-8.1347757047	-2.1697605993	-4.9092710214
H	-9.0692761577	-1.978734371	-2.8318682704
H	-6.9590761039	-3.6514957976	-1.4821589646
H	-7.5111767626	-6.409524808	-3.9768873567
H	-5.1811883152	-6.4340583382	-3.6535864722
H	-5.2499466726	-5.5034905404	-2.1523360921
H	-4.9565619721	-4.3653985565	-4.7982261509
H	-6.3594078378	-0.9179564954	-3.5503288245
H	-7.0241980501	-1.5329413757	-1.1947126511
H	-7.4058125393	-6.1743309142	-0.9532806583
H	-7.1836473826	-7.6443132128	-1.9020935008
H	-9.962451129	-4.0432565333	-1.7918889784
H	-8.9507499753	-4.8183550252	-0.5750822155
H	-5.0107150636	-2.503291322	-3.4235545198
H	-9.4442613105	-6.2174580695	-2.5253618883
H	-10.4226658465	-2.6710338655	-0.0684632649
H	-8.8045910788	-1.4167333392	2.5534301705
H	-8.8870868809	-0.175966589	1.307477344

H	-10.2922298312	-1.227481189	1.5943040525

calc_**2c** conf_47

C	-7.3778266214	-2.315082544	-4.3241512547
C	-6.8526490612	-1.8033148251	-2.967686116
C	-7.4747382781	-2.7780064849	-1.9529317081
N	-7.9037822746	-3.9736671457	-2.7150229008
C	-7.4935680933	-3.8328011537	-4.1192709486
C	-7.4263887382	-5.3073651758	-2.3238918251
C	-6.0642009797	-5.4734705966	-3.0108670671
C	-6.1990865417	-4.6919862724	-4.3175455922
O	-6.5411136377	-1.9230702925	-5.399474154
O	-5.4462729121	-1.7259424971	-2.9733691375
C	-7.4084417461	-5.6188515942	-0.8313866033
C	-8.6874717662	-2.108088749	-1.2895942336
O	-5.0105773613	-3.9377373982	-4.5181691894
O	-7.0798020751	-6.9752013293	-0.6092065962
N	-9.5096801193	-3.0205002778	-0.5417539021
O	-9.2249175097	-2.0095329152	1.4713174333
C	-9.7218351363	-2.8992212863	0.7995748159
C	-10.6153894036	-3.9582083675	1.4106532573
H	-8.2724421865	-4.2516382786	-4.7674073705
H	-8.3840295996	-1.916954372	-4.4993152116
H	-7.1890737327	-0.7801847896	-2.7742720161
H	-6.7304386564	-3.0164826246	-1.1862867615
H	-8.1230949038	-6.0291895406	-2.7727869233
H	-5.7834003474	-6.5174425051	-3.1692825848
H	-5.2756853668	-4.9956018306	-2.4146931837
H	-6.3377221497	-5.3631264488	-5.1736868019
H	-5.8720395063	-1.3453339907	-4.9900304046
H	-5.0959207418	-2.587006028	-3.2906768525
H	-8.400530861	-5.4722966208	-0.4017556471
H	-6.7196647859	-4.9527467856	-0.2938237774
H	-8.3605222758	-1.3333784911	-0.5925241781
H	-9.2961755156	-1.6299953394	-2.0693595587
H	-5.2197099099	-3.3339059467	-5.2544573771
H	-6.1367698973	-7.0834118111	-0.7819882874
H	-9.9377999812	-3.7726589981	-1.0575580568
H	-10.0255137789	-4.5563233818	2.110227491
H	-11.3989345441	-3.4628171593	1.9877173786
H	-11.0777890037	-4.625456152	0.6787358457

calc_**2c** conf_48

C	-7.3863935362	-2.1642257645	-3.870037857
C	-6.7210684161	-2.0132187761	-2.4748371724
C	-6.8792720566	-3.3968078563	-1.7922026812
N	-7.7335021338	-4.1762060717	-2.7178172224
C	-7.4527901584	-3.6799500044	-4.0656915878
C	-7.5176027897	-5.6260823492	-2.8380281043
C	-6.3200780194	-5.7962303862	-3.8065244055
C	-6.2090577574	-4.4514082037	-4.5579675276
O	-6.7364294323	-1.4438156269	-4.8714741302
O	-5.3764556866	-1.6071324911	-2.5952116369
C	-7.3823027928	-6.374334919	-1.5228847338
C	-7.4903602382	-3.3099727648	-0.3904003271
O	-5.0127683055	-3.7544713782	-4.1992875062
O	-8.5579601415	-6.3658043181	-0.7527823231

N	-8.9218763945	-3.1372104189	-0.4957255081
O	-9.1537215686	-2.1676663312	1.5359080087
C	-9.6518305247	-2.5277351439	0.4826212226
C	-11.1243072286	-2.3479691798	0.1792652549
H	-8.2784733918	-3.9638753931	-4.7293621894
H	-8.422869749	-1.811272003	-3.8027136451
H	-7.2108833691	-1.2273951264	-1.895965734
H	-5.8714589348	-3.8241242113	-1.6921180116
H	-8.4183368524	-6.0172651664	-3.3277468565
H	-6.4719864048	-6.6442218601	-4.47968731
H	-5.3893306129	-5.9767245277	-3.2563590754
H	-6.2320366448	-4.5683052776	-5.6461824517
H	-5.8920399625	-1.1691222518	-4.4730913693
H	-4.9177025708	-2.3523786549	-3.027754341
H	-6.5149873566	-5.9940633725	-0.9592017112
H	-7.1741753852	-7.4251896268	-1.7453787764
H	-7.2534602777	-4.2024872335	0.2015649213
H	-7.062081335	-2.463601763	0.1543861545
H	-4.2583171304	-4.2703255111	-4.5091784808
H	-8.8125503785	-5.4456755839	-0.5901267223
H	-9.3159416969	-3.3441036105	-1.4054163302
H	-11.7055944875	-2.9252786099	0.9026861679
H	-11.3849998646	-1.2965633791	0.31898105
H	-11.4089964136	-2.6577848536	-0.8292570516

calc_**2c** conf_49

C	-7.3673156967	-2.6343971485	-4.6602840259
C	-7.4616292636	-1.9287336433	-3.2857366002
C	-7.6978520316	-3.0657257954	-2.2522778574
N	-7.7884382189	-4.3127620054	-3.0691177145
C	-7.00409359	-4.07091609	-4.2882280195
C	-7.1775293886	-5.557141235	-2.5753571226
C	-5.6592691804	-5.400251924	-2.7580142666
C	-5.5293709644	-4.4315238365	-3.9547291204
O	-6.5174285436	-1.9894742573	-5.5574649061
O	-6.3170297006	-1.1507457561	-3.0265120104
C	-7.6718078504	-5.9877203969	-1.2160953009
C	-8.9568237108	-2.7382077505	-1.4359442343
O	-4.7755030083	-3.2704292179	-3.6034716558
O	-9.0880050122	-6.1239306753	-1.2317473418
N	-9.1595031666	-3.4745067377	-0.2134873228
O	-8.8416432937	-1.6851228782	1.1571969646
C	-9.1055209359	-2.868085948	1.0059961305
C	-9.4081422834	-3.7739773635	2.1821936543
H	-7.328693903	-4.7757497008	-5.0625872377
H	-8.3699369686	-2.6737472353	-5.1019510946
H	-8.2918156761	-1.2185548766	-3.2805001641
H	-6.8389434295	-3.0801251609	-1.5657033013
H	-7.5132837062	-6.3300464343	-3.282995234
H	-5.1636909717	-6.3608364344	-2.922759813
H	-5.2053054653	-4.9350822887	-1.875912592
H	-5.0549383107	-4.9013008808	-4.8226402562
H	-5.9774902664	-1.3961455716	-5.0073348764
H	-5.5749691128	-1.7807985152	-2.9567159744
H	-7.3753490851	-5.2609513382	-0.4488669455
H	-7.2379927447	-6.9567947932	-0.9493505927
H	-8.8882608768	-1.6910354033	-1.1299685111
H	-9.8295265233	-2.8469188984	-2.0936883725

H -3.8786120782   -3.5492117869 -3.3807193079
H -9.38713802   -5.6866744186 -2.0450970033
H -9.4154740577   -4.4537694831 -0.2773287232
H -8.6013071943   -3.681847204   2.9125543311
H -10.3241347899 -3.4257431498 2.6668076113
H -9.5302309803   -4.8250137664 1.9088388073

calc_**2c** conf_50

C -7.1716362517   -2.4555349858 -4.4168736742
C -8.1137412384   -2.1566649323 -3.2144089853
C -7.9842425955   -3.3736560458 -2.2800327166
N -7.8273955505   -4.4550830553 -3.269985115
C -6.8366336804   -3.9486955369 -4.2311142467
C -7.2594091892   -5.7547956228 -2.8781116404
C -5.73442808   -5.5539797695 -2.7744949034
C -5.4400140886   -4.3315496369 -3.6600067778
O -6.0843815052   -1.5583941437 -4.4610242443
O -7.8242413138   -0.9281608052 -2.6089868148
C -7.9050056863   -6.3973844935 -1.6677430368
C -9.2149447586   -3.4823232431 -1.3757646242
O -4.9091652339   -3.2514304286 -2.8807930751
O -9.2719818708   -6.6697426131 -1.8911678481
N -9.2099449016   -2.4241561153 -0.3895652542
O -8.2877625796   -3.7426333809 1.2176336851
C -8.6423134715   -2.6374202194 0.8334994148
C -8.4896259664   -1.4036167272 1.6968569675
H -6.9422629773   -4.5019169837 -5.1716851617
H -7.7091828428   -2.3080543764 -5.3554760636
H -9.1487800535   -2.136818741   -3.5830634568
H -7.0976131765   -3.2409958839 -1.6422867113
H -7.4587244382   -6.4197053561 -3.7300215948
H -5.1870504876   -6.4476413576 -3.085587917
H -5.4419673154   -5.3216287719 -1.7447824029
H -4.7434765529   -4.5565911247 -4.4758894291
H -5.4035510552   -1.9150380662 -3.8572254514
H -7.0326179541   -0.6045398339 -3.0801818445
H -7.7534967882   -5.7753357821 -0.7734235449
H -7.4191488113   -7.3595269932 -1.4767797012
H -10.1243944214 -3.4090022909 -1.9844538258
H -9.2553797841   -4.4169354   -0.819130664
H -4.0283597353   -3.5001050303 -2.5729011855
H -9.6200904884   -5.9348420382 -2.4140624373
H -9.2165125737   -1.4762089353 -0.7399987303
H -9.0739208526   -0.5511278627 1.3429121359
H -8.7905360483   -1.6510906194 2.7161436272
H -7.434065681   -1.117672797   1.7249772484

calc_**2c** conf_51

C -7.2593902024   -2.3107148429 -4.3637692889
C -7.5595736886   -1.920136164   -2.8922302695
C -8.0163446495   -3.2367495931 -2.221470911
N -8.0269875913   -4.2489582045 -3.2959075938
C -7.0372828103   -3.8251399341 -4.285368844
C -7.6083650773   -5.6120038274 -2.9568598496
C -6.0611940472   -5.5820535876 -2.9268026897
C -5.6686337972   -4.3858416717 -3.819298838
O -6.2399385395   -1.5524673306 -4.9388690847

O	-6.4364795537	-1.3258441841	-2.2794463967
C	-8.252865175	-6.2217166344	-1.7181964555
C	-9.396345192	-3.1192039818	-1.5701070708
O	-4.9345381622	-3.4016543154	-3.0820822685
O	-7.8098737518	-7.5558938871	-1.5383540586
N	-9.3897556215	-2.2341713338	-0.4235642164
O	-8.4130382462	-3.7556359664	0.9396801414
C	-8.8312743579	-2.620204031	0.7552006554
C	-8.748119444	-1.5493308711	1.8208224995
H	-7.2616543496	-4.3072522448	-5.2443640089
H	-8.1686259578	-2.1639579665	-4.9576123862
H	-8.3398438683	-1.1545299195	-2.8530773911
H	-7.2958585145	-3.4728585149	-1.4250023175
H	-7.9097172088	-6.2418228138	-3.8039427108
H	-5.6290040147	-6.5278659549	-3.2625047676
H	-5.6976829657	-5.3893790863	-1.9090790847
H	-5.0657351423	-4.6841269495	-4.6834775966
H	-5.7505163654	-1.1800138436	-4.1859204124
H	-5.7574518334	-2.0272826988	-2.2443401936
H	-9.3355572161	-6.2760920204	-1.8626347842
H	-8.0672320556	-5.6140570789	-0.8229598325
H	-10.1207020091	-2.7483297414	-2.3005913071
H	-9.7412226465	-4.0978707094	-1.2336206563
H	-4.1312320741	-3.8190386954	-2.7471297019
H	-6.9693832392	-7.522491558	-1.0673872657
H	-9.5949418348	-1.2579808933	-0.5623640267
H	-9.4690987176	-0.7403023552	1.6783659451
H	-8.9100909713	-2.0118833512	2.7950004038
H	-7.7414491073	-1.1201432432	1.8172666342

calc_**2c** conf_52

C	-7.3218466809	-2.2075823081	-4.027475607
C	-6.9735601141	-1.9018473738	-2.5352375939
C	-6.9741003014	-3.2591042029	-1.8116866082
N	-7.8266696508	-4.1035528994	-2.6768233147
C	-7.5305297217	-3.7301145285	-4.0597906665
C	-7.660101483	-5.5602329688	-2.670457942
C	-6.4717974115	-5.8650591761	-3.6187825219
C	-6.3430037401	-4.6180188421	-4.5116137705
O	-6.3603387851	-1.6992566325	-4.9207323801
O	-5.7303171233	-1.2684286672	-2.4089057132
C	-7.5455054892	-6.2251081319	-1.3055521707
C	-7.5188285554	-3.1437597146	-0.3877104129
O	-5.1147407233	-3.9267523356	-4.2672094026
O	-7.5954033571	-7.632118163	-1.4300978528
N	-8.9451139437	-2.9334825592	-0.4208659209
O	-9.0636298166	-1.9649517318	1.6211571003
C	-9.6198399522	-2.3563218904	0.6065023512
C	-11.1165075565	-2.2215509195	0.4088327932
H	-8.3825072172	-4.0070196583	-4.6928037991
H	-8.2587269657	-1.7121986398	-4.2899453161
H	-7.7638359064	-1.2703882819	-2.1052279091
H	-5.9305211248	-3.6010116795	-1.7705656582
H	-8.567116907	-5.9735272516	-3.1306962059
H	-6.6356270712	-6.7806478943	-4.1930123647
H	-5.5388018527	-5.98113041	-3.0537401254
H	-6.4198171481	-4.8530924801	-5.5794327294
H	-5.6031109671	-2.3132894745	-4.864747976

H	-5.5652063342	-0.8671359216	-3.2794160098
H	-8.3951582072	-5.943962675	-0.6788623531
H	-6.62785059	-5.905828204	-0.7917182951
H	-7.2698501557	-4.0262328399	0.2130942679
H	-7.0471531485	-2.2908070528	0.10835275
H	-4.3863362045	-4.470449761	-4.5932645486
H	-6.7486998612	-7.9287310302	-1.7848712743
H	-9.4224683131	-3.2931149365	-1.235319571
H	-11.4746638326	-2.6142190663	-0.5462473897
H	-11.6272550484	-2.7429217679	1.2219154514
H	-11.3854587388	-1.1650479296	0.4819586898

Table S40. Sampled and DFT geometry optimized conformers related to calc_**2d**, with energy values (Hartree) and related % contribution on the final Boltzmann distribution for the three employed functional/basis set combinations.

Conformer	MPW1PW91/6-31g(d,p)		MPW1PW91/6-311+g(d,p)		B97-2/cc-pVTZ	
	**Energy (Hartree)**	**% contribution on the Boltzmann distribution**	**Energy (Hartree)**	**% contribution on the Boltzmann distribution**	**Energy (Hartree)**	**% contribution on the Boltzmann distribution**
calc_**2d** conf_1	-916.6602254	86.03%	-916.8959853	48.60%	-916.8428697	23.79%
calc_**2d** conf_2	-916.6579264	7.54%	-916.8953805	25.61%	-916.8432410	35.25%
calc_**2d** conf_3	-916.6567706	2.22%	-916.8949115	15.58%	-916.8424834	15.80%
calc_**2d** conf_4	-916.6567506	2.17%	-916.8935842	3.82%	-916.8421527	11.13%
calc_**2d** conf_5	-916.6563070	1.36%	-916.8938544	5.09%	-916.8420066	9.54%
calc_**2d** conf_6	-916.6551986	0.42%	-916.8892291	0.04%	-916.8386682	0.28%
calc_**2d** conf_7	-916.6539039	0.11%	-916.8919865	0.70%	-916.8405758	2.10%
calc_**2d** conf_8	-916.6529672	0.04%	-916.8897929	0.07%	-916.8385423	0.24%
calc_**2d** conf_9	-916.6528236	0.03%	-916.8896250	0.06%	-916.8385582	0.25%
calc_**2d** conf_10	-916.6522584	0.02%	-916.8861748	0.00%	-916.8354509	0.01%
calc_**2d** conf_11	-916.6522133	0.02%	-916.8876632	0.01%	-916.8369902	0.05%
calc_**2d** conf_12	-916.6521418	0.02%	-916.8896318	0.06%	-916.8385297	0.24%
calc_**2d** conf_13	-916.6520655	0.02%	-916.8860581	0.00%	-916.8354306	0.01%
calc_**2d** conf_14	-916.6514534	0.01%	-916.8909015	0.22%	-916.8396257	0.77%
calc_**2d** conf_15	-916.6510172	0.01%	-916.8873766	0.01%	-916.8361109	0.02%
calc_**2d** conf_16	-916.6509492	0.00%	-916.8897769	0.07%	-916.8384907	0.23%
calc_**2d** conf_17	-916.6505226	0.00%	-916.8869896	0.00%	-916.8357173	0.01%
calc_**2d** conf_18	-916.6504742	0.00%	-916.8892337	0.04%	-916.8381284	0.16%
calc_**2d** conf_19	-916.6494853	0.00%	-916.8866687	0.00%	-916.8355612	0.01%
calc_**2d** conf_20	-916.6492676	0.00%	-916.8875793	0.01%	-916.8364101	0.03%
calc_**2d** conf_21	-916.6491423	0.00%	-916.8830372	0.00%	-916.8330498	0.00%
calc_**2d** conf_22	-916.6488090	0.00%	-916.8882200	0.01%	-916.8370444	0.05%
calc_**2d** conf_23	-916.6486700	0.00%	-916.8842583	0.00%	-916.8340766	0.00%
calc_**2d** conf_24	-916.6482572	0.00%	-916.8852167	0.00%	-916.8344317	0.00%
calc_**2d** conf_25	-916.6480039	0.00%	-916.8832578	0.00%	-916.8323040	0.00%
calc_**2d** conf_26	-916.6474383	0.00%	-916.8837327	0.00%	-916.8334163	0.00%
calc_**2d** conf_27	-916.6473577	0.00%	-916.8855042	0.00%	-916.8348847	0.01%
calc_**2d** conf_28	-916.6468530	0.00%	-916.8860622	0.00%	-916.8350201	0.01%

calc_**2d** conf_29	-916.6468474	0.00%	-916.8846051	0.00%	-916.8337990	0.00%
calc_**2d** conf_30	-916.6465621	0.00%	-916.8853328	0.00%	-916.8343019	0.00%
calc_**2d** conf_31	-916.6464920	0.00%	-916.8859177	0.00%	-916.8352544	0.01%
calc_**2d** conf_32	-916.6461138	0.00%	-916.8845756	0.00%	-916.8332151	0.00%
calc_**2d** conf_33	-916.6458219	0.00%	-916.8842265	0.00%	-916.8334170	0.00%
calc_**2d** conf_34	-916.6457829	0.00%	-916.8852733	0.00%	-916.8346174	0.00%
calc_**2d** conf_35	-916.6454169	0.00%	-916.8854930	0.00%	-916.8350438	0.01%
calc_**2d** conf_36	-916.6444355	0.00%	-916.8845010	0.00%	-916.8338331	0.00%
calc_**2d** conf_37	-916.6438194	0.00%	-916.8820646	0.00%	-916.8314767	0.00%
calc_**2d** conf_38	-916.6437971	0.00%	-916.8827304	0.00%	-916.8319925	0.00%
calc_**2d** conf_39	-916.6435138	0.00%	-916.8823569	0.00%	-916.8321443	0.00%
calc_**2d** conf_40	-916.6433259	0.00%	-916.8847580	0.00%	-916.8338578	0.00%
calc_**2d** conf_41	-916.6428984	0.00%	-916.8838123	0.00%	-916.8334919	0.00%
calc_**2d** conf_42	-916.6426125	0.00%	-916.8795741	0.00%	-916.8299328	0.00%
calc_**2d** conf_43	-916.6425132	0.00%	-916.8798507	0.00%	-916.8302082	0.00%
calc_**2d** conf_44	-916.6420538	0.00%	-916.8792092	0.00%	-916.8282448	0.00%
calc_**2d** conf_45	-916.6412700	0.00%	-916.8800598	0.00%	-916.8291861	0.00%
calc_**2d** conf_46	-916.6390525	0.00%	-916.8774673	0.00%	-916.8267158	0.00%
calc_**2d** conf_47	-916.6385838	0.00%	-916.8775320	0.00%	-916.8267990	0.00%

Table S41. Cartesian coordinates of the optimized geometries for the conformers related to calc_**2d**. The related energies and % contribution on the final Boltzmann distribution for the three employed functional/basis set combinations are reported in Table S40.

calc_**2d** conf_1

C	-5.6312983065	-2.1941409735	0.1640601368
C	-6.7619517758	-1.2695529933	0.6331875336
C	-6.7760044328	-0.195787355	-0.4528002485
N	-5.3454024628	0.0985583804	-0.5334142966
C	-4.6629229912	-1.2199128964	-0.5403515664
C	-4.8799650274	0.8759594357	-1.6965388589
C	-3.8728947539	-0.0350052024	-2.4237719253
C	-4.29013101	-1.4449412867	-2.0188500836
O	-6.1547831214	-3.1819336565	-0.7134138549
O	-7.9519729961	-1.9895392527	0.773415285
C	-4.2832407027	2.2045869871	-1.1831078884
C	-7.6861661745	0.9942648803	-0.1805694176
O	-5.3908448933	-1.7912071424	-2.8245735802
O	-4.955161443	2.619032791	-0.0131301111
N	-7.5240966963	1.6370901066	1.1068271191
O	-8.8939029621	0.2069680879	2.2102375532
C	-8.190740216	1.2195294111	2.2044293299
C	-8.0411809477	2.0704028772	3.4426239248
H	-3.7308048229	-1.1500002323	0.0337275355
H	-5.1442002775	-2.7014070907	1.0044304951
H	-6.4600154738	-0.8019827845	1.5789977793
H	-7.1357574753	-0.6535185021	-1.3883333994
H	-5.7343477445	1.0860363779	-2.3498098631
H	-3.90018945	0.079618323	-3.5092325117
H	-2.851788203	0.1707380345	-2.0821326103
H	-3.4700128008	-2.1712206985	-2.1223507035
H	-7.0678032213	-3.3315604375	-0.4107199283
H	-8.5525746299	-1.4003633952	1.2735938175
H	-4.3778623279	2.9961644534	-1.9313344017
H	-3.2098579581	2.0707084934	-0.9751481673
H	-8.7191061251	0.6414126229	-0.2521689271
H	-7.5421945437	1.7564544287	-0.9498918133
H	-5.8349448756	-2.5226322154	-2.356734282
H	-5.0584939806	1.760983276	0.4512766685
H	-6.9611983864	2.4743013929	1.1357126453
H	-7.3397512842	2.8987089185	3.3219108945
H	-9.0218978503	2.4710481318	3.7114877364
H	-7.7125376555	1.4371387047	4.2694599846

calc_**2d** conf_2

C	-5.7843527184	-2.2654769481	0.1057620243
C	-6.8974402203	-1.3155122821	0.5507346533
C	-6.8085482602	-0.2092039355	-0.5003130981
N	-5.3771281828	0.0426019629	-0.466902641
C	-4.7218972104	-1.2865695214	-0.4339991491
C	-4.775160546	0.8385726483	-1.5380753308
C	-3.6560802139	-0.0482615362	-2.1230930751
C	-4.1503564509	-1.4643104319	-1.8519057904
O	-6.2814971643	-3.1499059043	-0.8915462784
O	-8.1269078608	-1.9793223476	0.6184226862
C	-4.2353921749	2.1578765027	-0.9980342411

C	-7.6880259844	0.996552408	-0.2132776722
O	-5.1361646242	-1.7400608249	-2.8177196944
O	-5.2391103902	3.015385925	-0.4682941058
N	-7.4663139038	1.6092890787	1.0741031554
O	-8.9431056952	0.2679178303	2.1525398795
C	-8.1169351973	1.1848000744	2.1762999499
C	-7.802180021	1.9140597959	3.4609143552
H	-3.8741030412	-1.2621972337	0.2644145734
H	-5.394215529	-2.8641790791	0.9366245624
H	-6.6160323466	-0.8883418874	1.5205387305
H	-7.1365031107	-0.6217510989	-1.47086748
H	-5.5209981118	1.0430499527	-2.3210482385
H	-3.4969569872	0.117933681	-3.1904939918
H	-2.7082504815	0.1302894685	-1.6002819986
H	-3.3382571352	-2.2064256131	-1.8770761793
H	-7.220965098	-3.2782747859	-0.6752028609
H	-8.7101627532	-1.3838487526	1.1296560554
H	-3.6581774219	2.6797008029	-1.7753131901
H	-3.5608784198	1.9454707202	-0.1646252726
H	-8.7328239923	0.6791643446	-0.2611362364
H	-7.5392381666	1.7598665902	-0.9808440032
H	-5.6892238796	-2.445835825	-2.4349550446
H	-5.7135224167	3.4056893864	-1.2127283929
H	-6.6877080106	2.2515588518	1.1317746189
H	-8.7191544491	2.3788281434	3.8318518925
H	-7.0343186084	2.682415792	3.3484856642
H	-7.4809132218	1.1884540481	4.2116111642

calc_**2d** conf_3

C	-5.8290489521	-2.0252499695	-0.0559874865
C	-6.93862182	-1.1686749759	0.539005382
C	-6.9391633649	0.0264634469	-0.4174228463
N	-5.5117545463	0.2946415651	-0.6051303451
C	-4.7661083903	-0.9710309728	-0.3638199159
C	-5.1458189459	0.8152224415	-1.9390926397
C	-4.5686425412	-0.3728050442	-2.7033706703
C	-3.8874619673	-1.2188210159	-1.617459235
O	-6.3026382609	-2.6297686009	-1.2522665948
O	-8.1465923119	-1.8718940577	0.5821712422
C	-4.1734050289	1.9851207661	-1.736789128
C	-7.7508409789	1.2360725822	0.0380530135
O	-3.6861831058	-2.5683801248	-1.9354972334
O	-4.6432176733	2.8043760478	-0.693436262
N	-7.5360626045	1.6515851273	1.4106060652
O	-9.0273322436	0.1826278367	2.27585804
C	-8.2458737536	1.1208267364	2.4327049379
C	-8.0435094323	1.7512900494	3.7896492166
H	-4.1340620733	-0.8671537312	0.5255439975
H	-5.465950695	-2.7932822936	0.6367552854
H	-6.6250735415	-0.8389992219	1.5387216954
H	-7.4003294287	-0.3211263927	-1.3558883747
H	-6.049494003	1.2123628047	-2.4143552297
H	-5.3755412374	-0.9620530434	-3.1502304703
H	-3.8765275811	-0.0833556417	-3.4998412244
H	-2.8871281863	-0.8195203842	-1.4241991901
H	-7.2019948101	-2.9386474278	-1.0564972491
H	-8.7428428253	-1.3261075832	1.1310939806
H	-4.1058575281	2.5912461503	-2.6448000459

H -3.1617557295 1.6046104152 -1.5180393463
H -8.8132329297 0.9992351401 -0.0703550344
H -7.5269918119 2.0894526454 -0.6077363475
H -4.5706359147 -2.9606689528 -2.0244701209
H -4.9277641909 2.1334221764 -0.0411580798
H -6.9256876116 2.43205762 1.5900133682
H -7.2564951119 2.5084607105 3.8097390428
H -8.9841331571 2.2102318955 4.1050984617
H -7.8072257115 0.9672332766 4.511829341

calc_**2d** conf_4

C -5.7682599365 -2.1544457518 -0.282312966
C -6.4344751139 -1.3426213985 0.8247892922
C -6.4173892825 0.0768554711 0.2284464386
N -5.1120891606 0.1445337797 -0.4387815684
C -4.6420756341 -1.2317749734 -0.733119338
C -5.0862506444 0.935834635 -1.6862516822
C -5.0777852105 -0.0855197957 -2.8208550319
C -4.2898402654 -1.2708342032 -2.2435479799
O -6.7154659654 -2.3400622227 -1.3249539957
O -7.7042009883 -1.8786464324 1.0612819683
C -3.8585397338 1.8521149889 -1.6586674687
C -6.5627603555 1.2401754323 1.2233556789
O -4.5104106536 -2.5066885939 -2.8653688181
O -3.7666766312 2.5002863035 -0.4133389106
N -7.9241346004 1.6942797281 1.4500318033
O -8.5345897841 0.0039340232 2.8232432697
C -8.79894792 1.0693358161 2.2631146785
C -10.1310041383 1.7489944716 2.4707679316
H -3.7555518274 -1.4623222099 -0.1307783582
H -5.3991781843 -3.1247317733 0.0703799384
H -5.8074302116 -1.3671757479 1.7288020711
H -7.2423994802 0.134007586 -0.499093349
H -5.976366489 1.5745353332 -1.709912082
H -6.0973715724 -0.4182168761 -3.0368842067
H -4.6370292657 0.2957370764 -3.7469702757
H -3.2176836686 -1.0827288444 -2.3562595064
H -7.5620110118 -2.4994563537 -0.8729773521
H -8.1142814961 -1.3408038806 1.7709436898
H -3.9410957494 2.6224427586 -2.4309199296
H -2.9464083732 1.2707236917 -1.8707034541
H -6.0073405628 2.0951053084 0.8306037912
H -6.1166990983 0.9700956923 2.1862519927
H -5.441942072 -2.7290885309 -2.7000723242
H -3.9048417402 1.7692880294 0.2164837675
H -8.2078495021 2.5585115898 1.0178779558
H -10.2480210788 2.6776354025 1.9076206559
H -10.9272371861 1.0563445121 2.1889018646
H -10.2493654116 1.9623459588 3.5358718097

calc_**2d** conf_5

C -5.577889399 -2.2199369409 -0.107679016
C -6.2537967973 -1.2839267045 0.9052247635
C -6.3776544508 0.0385203183 0.131999337
N -5.0673746771 0.1084753613 -0.5106180255
C -4.7257996643 -1.2674809646 -0.9603567721
C -4.9221348771 1.0230347913 -1.6656433523

C	-4.3545611823	0.1441640758	-2.7918686409
C	-4.920876169	-1.232050305	-2.4856193411
O	-6.5704566654	-2.8972624193	-0.8649670024
O	-7.449398305	-1.8782959529	1.3186458037
C	-3.9801868405	2.1702750568	-1.287598067
C	-6.6441351191	1.2738086445	1.0021858652
O	-6.2768257481	-1.2087755839	-2.8665724379
O	-2.8436497523	1.6699724129	-0.6311580245
N	-8.050209188	1.5577707044	1.2330696017
O	-8.3325409232	0.0984030721	2.9388707309
C	-8.7864577651	0.9754144531	2.2009962469
C	-10.2081574249	1.4601585998	2.3495052502
H	-3.6620450265	-1.4530472905	-0.7667783985
H	-4.9562349568	-2.9688765058	0.3960711622
H	-5.5712006014	-1.120251976	1.7515724522
H	-7.1877590317	-0.0595190131	-0.6095579751
H	-5.9035863592	1.4166141331	-1.9612643363
H	-4.6564633662	0.4907932185	-3.7825813649
H	-3.2612974429	0.1348047401	-2.7339761746
H	-4.3716431553	-2.0441305587	-2.9843411734
H	-7.3375210041	-2.9587069168	-0.266808124
H	-7.8305825847	-1.3151051577	2.0251183785
H	-4.5074478678	2.9080040557	-0.6593327198
H	-3.6417504875	2.6965030432	-2.1859317316
H	-6.2246955954	2.1571246524	0.5162576021
H	-6.1488175411	1.1683809452	1.9718359807
H	-6.6927031488	-1.9422583838	-2.3768031604
H	-3.2193516067	1.0283039322	-0.0014321517
H	-8.4982376012	2.2321100681	0.6340510936
H	-10.4805843452	2.2571992439	1.6540275791
H	-10.8849587301	0.614374945	2.2069505087
H	-10.3470145985	1.8174142056	3.3725056339

calc_**2d** conf_6

C	-5.9770038731	-2.5419160341	-0.2758372687
C	-7.305603866	-1.7923890231	-0.5061141412
C	-7.024202007	-0.3637777711	-0.0577246032
N	-5.5881808041	-0.1506694469	-0.3113670478
C	-4.8962826892	-1.4486368194	-0.4748494044
C	-5.2251781385	0.7619297016	-1.4107431194
C	-3.9532863126	0.1321437876	-1.9795400911
C	-4.2147750462	-1.3666110688	-1.8529428168
O	-5.8448136676	-3.6789761399	-1.0912853738
O	-7.6035839766	-1.7746058088	-1.8993796246
C	-5.0472497835	2.1683938463	-0.8267987821
C	-7.3713468233	-0.1148601158	1.4111327822
O	-5.0316191765	-1.8553945358	-2.9102647974
O	-4.3428129635	2.0949569033	0.3949795105
N	-7.1177513122	1.2527634277	1.7903039615
O	-9.2474539618	1.6804103264	2.4581676019
C	-8.1022945782	2.0629835403	2.2750959071
C	-7.6671636653	3.4784461382	2.589415632
H	-4.1138402856	-1.5595603069	0.2849483504
H	-5.9484913029	-2.894449476	0.7599532746
H	-8.138019261	-2.2519822135	0.0394732639
H	-7.6105335981	0.3326620504	-0.6662328167
H	-6.0083158699	0.7924255302	-2.1828822145
H	-3.7583348499	0.4229034913	-3.0137947678

```
H  -3.0923631661  0.4202927334   -1.3658763943
H  -3.2996829452  -1.9608239756  -1.8921892904
H  -5.4830900115  -3.3608770832  -1.9421415985
H  -7.636758234   -2.7098342045  -2.1597479701
H  -6.0356416761  2.6258659531   -0.6732764545
H  -4.4895008514  2.8075672221   -1.5188792288
H  -6.8017206136  -0.8010719704  2.0526073392
H  -8.434686101   -0.2897206707  1.5916221661
H  -5.9392443531  -1.51275175    -2.7763055283
H  -4.4953600563  1.1681796506   0.673066004
H  -6.2002335478  1.6420438511   1.6204585702
H  -7.7496749162  3.6397958272   3.6675244509
H  -6.6457715415  3.7054403045   2.2744079485
H  -8.3581341734  4.1717041291   2.1050165714
```

calc_**2d** conf_7

```
C  -5.9184358948   -2.516593991   -0.259362422
C  -6.5093533434   -1.70602221    0.9019404998
C  -6.2814635091   -0.2501921616  0.4776319795
N  -4.9828100933   -0.2844274928  -0.1673603963
C  -4.7205003474   -1.6529397755  -0.6789305964
C  -4.6962891764   0.6796263511   -1.2288987595
C  -3.7071066138   -0.0842437109  -2.1124019189
C  -4.3505567209   -1.4687851397  -2.1628273857
O  -6.9099167284   -2.6376479976  -1.2624353766
O  -7.8612278838   -2.0418004801  1.0647355587
C  -4.1785206577   2.0231580428   -0.7359458979
C  -6.3591313105   0.7217035209   1.6756309873
O  -5.4452853267   -1.3685409363  -3.0397285445
O  -5.2093950009   2.8817075856   -0.2505847803
N  -7.3487500331   1.7626557267   1.521725749
O  -9.0852663737   0.3409131385   1.7950212405
C  -8.6634915193   1.4786884923   1.5673050762
C  -9.6158109552   2.6280605675   1.3453421587
H  -3.832756306    -2.0593351058  -0.1725942537
H  -5.5991287972   -3.5183934102  0.0526471425
H  -5.9340517055   -1.9109120392  1.8177651784
H  -7.0779287709   0.0187646245   -0.2392882259
H  -5.6010830527   0.8692525536   -1.8366454168
H  -3.6105593164   0.3464597188   -3.1114864875
H  -2.7173631874   -0.1241783715  -1.6416952781
H  -3.6513764141   -2.2500814542  -2.4946876992
H  -7.7537962218   -2.5388496909  -0.7778175763
H  -8.3412168143   -1.2466225728  1.3897303736
H  -3.6314238407   2.5265163235   -1.5458468538
H  -3.4877632352   1.8726038411   0.0965791715
H  -5.390579538    1.2000054292   1.8126118496
H  -6.5946577849   0.1727920564   2.5931906503
H  -6.171504637    -1.8968219681  -2.6620600367
H  -5.7644840955   3.0943965112   -1.0121475672
H  -7.0180357368   2.6509176482   1.1767744968
H  -9.1138949148   3.5863762329   1.1944657448
H  -10.2432132206  2.4079696451   0.4781097747
H  -10.2768709219  2.7058204988   2.2115378414
```

calc_**2d** conf_8

```
C  -6.2945318769  -2.4218033696  -1.0605008557
```

C -6.9074687226 -1.9226700604 0.2638478562
C -6.380447981 -0.5018731704 0.3888591587
N -5.0044710193 -0.5975239854 -0.0742145291
C -4.9923846654 -1.5988883229 -1.1781995657
C -4.4096016683 0.6545456055 -0.5812678236
C -3.7769884728 0.2698282019 -1.9265758726
C -4.7429141036 -0.7779038137 -2.4570483553
O -7.2173667026 -2.1853876372 -2.1098302484
O -8.3147005386 -1.9769457858 0.2114588441
C -3.3921747718 1.2799205742 0.3584355702
C -6.5666072614 0.0325668053 1.8156441515
O -5.8789406944 -0.0781723674 -2.9018789066
O -3.9496832773 1.7485031629 1.5821148922
N -7.0667513209 1.3864510536 1.8522090293
O -9.0991800623 0.6734758125 1.172456509
C -8.3413234521 1.6043047869 1.4570505403
C -8.799128372 3.0393858188 1.392719789
H -4.1341666255 -2.2697171259 -1.0412717153
H -6.0746460436 -3.4949483906 -1.0298893099
H -6.5328753706 -2.5356393533 1.095243859
H -6.9779280533 0.1255905946 -0.2943850612
H -5.2050667724 1.3910222369 -0.763525382
H -3.6931328944 1.1180045641 -2.6093002345
H -2.7792643738 -0.1620306267 -1.7793541836
H -4.3068251147 -1.396914771 -3.2553143767
H -8.0772088933 -2.1087613401 -1.6461582538
H -8.6701693495 -1.1249603948 0.5435101566
H -2.9517171057 2.1541413855 -0.1327897232
H -2.583049597 0.5649455556 0.5546079758
H -5.6250891961 -0.019194361 2.3595500134
H -7.2887562986 -0.5907316833 2.3510864842
H -6.6042618266 -0.731299996 -2.8947536894
H -3.8722557451 1.0285558004 2.2176199526
H -6.390585761 2.1366844163 1.8738308664
H -9.0314099226 3.2915737377 0.3546992529
H -9.7230100263 3.1388643716 1.9663276418
H -8.0599160667 3.7460020713 1.7759855434

calc_**2d** conf_9

C -6.2712963749 -2.3002254859 -1.090847792
C -7.1496761756 -1.9365934586 0.1091257919
C -6.6978008432 -0.5132287744 0.4331526607
N -5.2750723995 -0.462663225 0.0914978998
C -4.9378057899 -1.6590405373 -0.7100214582
C -4.8328010131 0.7573166581 -0.6188078767
C -4.4532400674 0.3159054571 -2.0306276561
C -4.0681325241 -1.1650816161 -1.8895939913
O -6.8081412075 -1.6785293838 -2.2443930827
O -8.5160337996 -2.0012491715 -0.2485178363
C -3.6930699641 1.3961527453 0.1781071066
C -7.0063445699 -0.0942684685 1.8716650999
O -4.196548031 -1.9275054604 -3.0589272139
O -4.0342650567 1.4415127038 1.5487840273
N -7.1509747146 1.3365597093 2.0138753326
O -9.2352064519 1.234287887 1.1437200305
C -8.2963552214 1.9094412168 1.558039405
C -8.3583019074 3.4172476428 1.6153492006
H -4.3810227188 -2.3803826605 -0.0992122969

H	-6.1826598918	-3.3824585916	-1.2451034007
H	-6.9431402181	-2.619052822	0.946026584
H	-7.2696744129	0.1478134915	-0.2338147017
H	-5.6650363359	1.4699394836	-0.6305989644
H	-5.3232499044	0.3832559304	-2.688886068
H	-3.6467671069	0.9123640676	-2.4680819608
H	-3.0141693527	-1.2462484531	-1.6065351514
H	-7.7739746825	-1.7335861977	-2.1322560032
H	-8.9695805875	-1.2319296432	0.1306186164
H	-3.5153546007	2.42285291	-0.1528894917
H	-2.7597224868	0.8306849993	0.0297399796
H	-6.2186369672	-0.4481408329	2.5422464789
H	-7.948536303	-0.5447595394	2.1961590325
H	-5.1373119361	-1.8945650397	-3.2987768593
H	-4.3051711656	0.5206460328	1.7196008552
H	-6.3045134069	1.8835293461	2.1083868231
H	-9.2018736755	3.7086582937	2.2457431246
H	-7.4476556537	3.8753374572	2.007568256
H	-8.554882481	3.8040033287	0.6124855003

calc_**2d** conf_10

C	-6.0318715848	-2.5578783302	-0.2608322411
C	-7.3589589265	-1.817863771	-0.5340352615
C	-7.1205032273	-0.4272230275	0.0579031048
N	-5.6867917292	-0.1651762392	-0.1055892084
C	-4.9583119155	-1.4403923966	-0.3122295381
C	-5.3004095886	0.8061954218	-1.1488355456
C	-3.9752362027	0.2503063998	-1.6652478451
C	-4.1856179344	-1.2528905841	-1.6285639935
O	-5.8303991338	-3.613414479	-1.1881642488
O	-7.6277068212	-1.828353798	-1.9210379298
C	-5.194100448	2.1963312317	-0.519482282
C	-7.5540037325	-0.3654073774	1.5324739465
O	-4.9625816696	-1.6102716383	-2.7665013942
O	-4.4558346522	2.1237204819	0.6789510301
N	-7.1925050461	0.8672782029	2.1898589491
O	-8.7821681179	2.0901341718	1.1467817205
C	-7.8228179557	2.0439478961	1.9016561691
C	-7.2301887245	3.2595738106	2.5793190753
H	-4.2286027917	-1.5910667994	0.4931494242
H	-6.0470955762	-2.990947569	0.7457773775
H	-8.2100760762	-2.3245582105	-0.0695965236
H	-7.7030239466	0.3219190714	-0.4847091086
H	-6.0389064057	0.8364498376	-1.9629271329
H	-3.7292698085	0.5987100603	-2.6704692726
H	-3.1692090417	0.5368138936	-0.9820648203
H	-3.2464278521	-1.8176897271	-1.6322771578
H	-6.6382414387	-3.6095253589	-1.7340320986
H	-6.8408761661	-1.4585411509	-2.37367048
H	-6.2041495303	2.5945006974	-0.3416010985
H	-4.680986636	2.8856737795	-1.1972142895
H	-7.1076033166	-1.1873537795	2.1014144649
H	-8.6411479655	-0.4772036083	1.5805581097
H	-5.0882237562	-2.5704969821	-2.6679338478
H	-4.7362641904	1.2573867995	1.0335190317
H	-6.3400172586	0.8982990681	2.7242410495
H	-7.199315195	3.133198364	3.6659683513
H	-6.2054414748	3.4162191251	2.2258974088

H -7.8381141627 4.1295965141   2.3355461053

calc_**2d** conf_11

C	-5.7683826807	-1.657155312	0.5094066485
C	-7.1060694313	-0.9870048314	0.1410577704
C	-6.7013337097	0.4558461776	-0.1751138848
N	-5.349087722	0.3769300729	-0.7358370836
C	-4.7170644233	-0.9074792356	-0.3481158778
C	-5.1986967603	0.5972911926	-2.1857171723
C	-4.0597106008	-0.3470220802	-2.5659455547
C	-4.2864752159	-1.5555097809	-1.6750457594
O	-5.8046276156	-3.060384036	0.2756648956
O	-7.7026946196	-1.6573547461	-0.9517397714
C	-4.8871770792	2.0739940843	-2.4324601407
C	-6.6847950371	1.3732730182	1.0576716599
O	-5.3253690328	-2.336959051	-2.255562904
O	-3.8400102678	2.5010251103	-1.6005780356
N	-7.9766418951	1.5647671079	1.6763761744
O	-7.6653709388	-0.0049439955	3.2794844407
C	-8.3627877714	0.86663332	2.7802828844
C	-9.7127058131	1.2380012364	3.3537042874
H	-3.8172893573	-0.7160065781	0.2487961339
H	-5.5631036313	-1.490663329	1.571565409
H	-7.8208402373	-1.0512166779	0.9621894739
H	-7.3930698975	0.8691476939	-0.9183833722
H	-6.1143137084	0.3237698775	-2.7310904118
H	-4.0666480066	-0.6188552543	-3.6235627846
H	-3.1032129706	0.1274432159	-2.3257128939
H	-3.3878792782	-2.1708976411	-1.552553266
H	-6.7334233238	-3.2501760134	0.0566155423
H	-7.0262011569	-1.7239959666	-1.6580479236
H	-5.7999717925	2.6708100534	-2.2660725624
H	-4.580852824	2.2320500263	-3.4712742254
H	-6.2807045988	2.3450881262	0.7577738779
H	-6.0233219831	0.9602717171	1.822748691
H	-5.4572350289	-3.0538414801	-1.6078347122
H	-4.0376324425	2.0628411938	-0.7525248635
H	-8.5960254757	2.2633105811	1.299241285
H	-9.5721873426	1.5857622509	4.3800194535
H	-10.2392405263	2.0098841232	2.7870438196
H	-10.3348458036	0.3413258297	3.3975307526

calc_**2d** conf_12

C	-5.7990352927	-2.0470213663	-0.2931833045
C	-6.51906256	-1.2639294865	0.7981493045
C	-6.51169215	0.1683547679	0.2265407871
N	-5.2112042537	0.2696494813	-0.4402405976
C	-4.6785464938	-1.09396489	-0.6822686215
C	-5.1890153305	1.0346632414	-1.707077602
C	-5.0176511464	-0.0116663286	-2.8117620138
C	-4.2176993932	-1.1440372523	-2.1589595064
O	-6.6953340101	-2.2372690729	-1.378773373
O	-7.7873260761	-1.8232610211	0.9900990728
C	-4.0668136982	2.0755581361	-1.6271264172
C	-6.6740692228	1.2982979375	1.2562790255
O	-4.35346826	-2.4036496905	-2.7619862691
O	-2.8783103227	1.5182677314	-1.1199741131

```
N  -8.0471091328   1.676034083    1.5498273522
O  -8.504530907    -0.0598169173  2.9258682472
C  -8.8527124947   0.9926299843   2.3881429545
C  -10.2153864357  1.5883915593   2.6493260725
H  -3.8298314064   -1.2920307788  -0.0152302387
H  -5.4227700075   -3.0136835194  0.0624925599
H  -5.9228506731   -1.2919305426  1.722098877
H  -7.3393621621   0.2338790216   -0.4972183158
H  -6.1402004278   1.5696773707   -1.8250507951
H  -5.9982384501   -0.4042625189  -3.0984578653
H  -4.5289743666   0.3819557967   -3.7069055347
H  -3.1534709366   -0.9042398349  -2.2026018044
H  -7.5510726892   -2.4516294458  -0.971234473
H  -8.1816255159   -1.3642202199  1.7590696296
H  -4.4024161122   2.9164489106   -0.9969238729
H  -3.8382713131   2.4781323338   -2.6182932016
H  -6.1789393231   2.1923168018   0.870569004
H  -6.1761154546   1.0284704386   2.1927728408
H  -5.290014789    -2.6454713705  -2.672947914
H  -3.1580428188   1.0952852162   -0.2917403339
H  -8.4061821245   2.5148242241   1.1238569787
H  -10.3976500646  2.529227811    2.1250884687
H  -10.9785864753  0.8630662836   2.3576573482
H  -10.3204177093  1.7539531252   3.7241176444
```

calc_**2d** conf_13

```
C  -5.6439979524  -2.5800717405  -0.5602794989
C  -7.0476137636  -2.0315615209  -0.1714249164
C  -6.8367820144  -0.5414372622  0.1461219586
N  -5.4038968851  -0.2707490757  -0.0859348504
C  -4.8211601579  -1.3361781826  -0.9099360584
C  -5.0963045309  1.0041788213   -0.7512550404
C  -4.8123149571  0.6833533945   -2.2370765942
C  -4.8848649859  -0.8413876374  -2.3680651199
O  -5.7335058789  -3.5125225369  -1.62057079
O  -7.9754650402  -2.2588823974  -1.2111655729
C  -3.9032516593  1.6425960368   -0.0317365133
C  -7.2510722598  -0.1776521268  1.5714123935
O  -6.1073303331  -1.1952460479  -3.0109568648
O  -4.0853881757  1.5807624919   1.3668252138
N  -7.0681040396  1.2204969561   1.8751239301
O  -9.2677218966  1.6358468555   2.2764213333
C  -8.1117939784  2.0264656737   2.2205748511
C  -7.7349208328  3.4582276623   2.5396749287
H  -3.7656312498  -1.4724077073  -0.6431742687
H  -5.1929557826  -3.0689388005  0.3111785322
H  -7.4461875715  -2.5749522791  0.689215577
H  -7.4620264696  0.0428290336   -0.5438656317
H  -5.9568472703  1.668766556    -0.6346884812
H  -5.5458969087  1.1318023986   -2.9103391373
H  -3.8259947353  1.0528618108   -2.5360736967
H  -4.0504508644  -1.2403864608  -2.9558638211
H  -6.6903400598  -3.6890452135  -1.70191236
H  -7.6945407764  -1.6760245882  -1.9432718073
H  -3.8008839578  2.6953440349   -0.3075379214
H  -2.971011263   1.1312662461   -0.3206684629
H  -6.6774159799  -0.7960417787  2.2757144297
H  -8.3136847937  -0.3878450412  1.7163016347
```

H	-6.0473515848	-2.1642493093	-3.0817664738
H	-4.3083138953	0.6413077929	1.508094567
H	-6.1277339302	1.5990547399	1.8580767881
H	-8.0022190707	3.6669873134	3.5787027923
H	-6.6743275358	3.6777496431	2.3953017674
H	-8.3306969588	4.1256822455	1.9128231841

calc_**2d** conf_14

C	-6.2417416242	-2.4163489056	-0.5422873218
C	-7.5760802063	-1.734839331	-0.2368565563
C	-7.295111789	-0.2671922677	-0.6364739634
N	-5.8784809381	-0.0588782656	-0.3549313914
C	-5.2683163089	-1.3597281295	-0.0099137577
C	-5.0698159833	0.5630351949	-1.4038446808
C	-3.6632638059	0.1303365064	-1.0067348407
C	-3.8228822304	-1.3155596714	-0.5432374427
O	-6.2414158896	-2.5604786045	-1.9470292258
O	-8.6684057251	-2.3013194067	-0.9035379768
C	-5.2249089965	2.0828357311	-1.4632261006
C	-8.2227688197	0.7295625933	0.0626045463
O	-3.6726573516	-2.2583150742	-1.6099646175
O	-4.7962489917	2.7366723249	-0.3060805961
N	-8.1142342703	0.7237450825	1.5059434119
O	-6.7726001256	2.5502015517	1.6752146768
C	-7.3966823821	1.6354665495	2.2028061635
C	-7.4221299437	1.4831368851	3.7074419971
H	-5.2376894379	-1.4723779932	1.0833032945
H	-6.1311494589	-3.3872906595	-0.0402677916
H	-7.7834262598	-1.8051817851	0.8373509879
H	-7.4915512715	-0.1886704401	-1.7177406784
H	-5.3284099041	0.1514354127	-2.3981975806
H	-2.9334687368	0.2321761264	-1.8173274327
H	-3.3402757607	0.7649491589	-0.1763698433
H	-3.1076945419	-1.5793221703	0.2445058154
H	-5.3086169062	-2.6407269921	-2.2126700551
H	-8.3284801776	-2.5238357886	-1.7857181502
H	-6.2680540802	2.3318011765	-1.7158113767
H	-4.6105583629	2.4533945081	-2.2927671472
H	-9.2559050893	0.4920873112	-0.2126649982
H	-8.0099570467	1.7479969939	-0.2662173861
H	-2.8324650239	-2.0816367484	-2.0505958764
H	-5.4653921363	2.5864895085	0.3885504969
H	-8.629797894	0.0298471249	2.0216490247
H	-8.0478808759	0.6602837245	4.0617690943
H	-6.3993363242	1.3286436022	4.0594802967
H	-7.7781453294	2.4176051658	4.146846982

calc_**2d** conf_15

C	-5.5292574003	-2.1852861326	0.0560162221
C	-6.3275581143	-1.224666259	0.9783098443
C	-6.388631898	0.0858236889	0.188821423
N	-5.0841415139	0.1353033471	-0.4701400812
C	-4.6809600614	-1.2501753525	-0.8183748892
C	-4.980320502	0.9754453993	-1.6851932645
C	-4.3062388623	0.0661362764	-2.7219413172
C	-4.8129443022	-1.3176781771	-2.3493516338
O	-6.4356126961	-2.974918433	-0.6898489515

O	-7.5783876783	-1.8032936057	1.2686093432
C	-4.1625859119	2.2290913081	-1.3659318765
C	-6.5633196817	1.3523184606	1.0374047894
O	-6.1506349731	-1.3901957188	-2.7807333582
O	-2.9986303431	1.8859182715	-0.655696192
N	-7.5586809796	1.3188417463	2.1027383984
O	-9.2494279838	0.3312703137	0.9660498567
C	-8.8382382766	0.912353305	1.9727052171
C	-9.7479627027	1.1719757649	3.1495576013
H	-3.6198061348	-1.3835997376	-0.572080976
H	-4.8912670404	-2.853157301	0.6467010826
H	-5.7707587672	-1.0677914615	1.9115069847
H	-7.2025595835	0.0133440859	-0.5462395284
H	-5.9825003804	1.2547633383	-2.0359745273
H	-4.5790817855	0.3311636812	-3.745656879
H	-3.2181623849	0.1258135489	-2.6142250757
H	-4.2023258795	-2.1289288673	-2.772451349
H	-7.2469557639	-2.9656753255	-0.1402956084
H	-8.2804664834	-1.1643503913	1.0238212446
H	-4.7773954737	2.945868925	-0.7967575149
H	-3.8558315982	2.729044791	-2.290131563
H	-6.7756372694	2.1967332565	0.3703601229
H	-5.6018831501	1.5486841377	1.5183920286
H	-6.5434160479	-2.1228960758	-2.2712632125
H	-3.3189373721	1.2191466272	-0.0215598224
H	-7.3133503484	1.8049024175	2.9503808977
H	-9.2741450385	1.7177673628	3.9685286392
H	-10.6143467884	1.7368483493	2.7979084258
H	-10.1156388287	0.2140544353	3.5250354993

calc_**2d** conf_16

C	-5.822369136	-2.4696726217	-0.5847750635
C	-6.4488669734	-1.7106828566	0.6056345102
C	-6.3989528487	-0.2466773739	0.1495435967
N	-5.0588953882	-0.1742164344	-0.42111217
C	-4.8061768443	-1.4482722258	-1.1377597359
C	-4.7557660106	0.953603991	-1.3220944166
C	-4.1316633966	0.2943555452	-2.563148469
C	-4.8068526185	-1.0675694631	-2.6201651459
O	-6.8568562523	-2.8772131762	-1.4592749329
O	-7.729511688	-2.1897564381	0.9149229438
C	-3.7911917099	1.9159273263	-0.6266022778
C	-6.6278627345	0.7485515223	1.2777811557
O	-6.1631983887	-0.9214052844	-3.0558392365
O	-2.7038803041	1.2053598855	-0.0870467505
N	-7.959141643	0.589939261	1.8142968012
O	-8.3563271376	2.8133209425	2.0318318267
C	-8.7384360083	1.6563714128	2.1331835058
C	-10.1244455148	1.3145770058	2.6400612466
H	-3.7903310924	-1.7950747943	-0.9118525747
H	-5.3233591189	-3.3844664448	-0.2550117703
H	-5.8187623647	-1.8204399303	1.4959817968
H	-7.179335294	-0.0923409717	-0.6172204104
H	-5.6812051634	1.4784926092	-1.5957385498
H	-4.2830256422	0.8813134122	-3.4743235576
H	-3.0544542733	0.174372111	-2.4065637214
H	-4.2838090942	-1.8034547725	-3.2421433257
H	-6.9157161459	-2.2224099094	-2.1803275188

H	-8.0581257563	-2.5409125727	0.0652181551
H	-4.325470574	2.4807962546	0.1513769344
H	-3.3882810787	2.6397338338	-1.3418527718
H	-6.5451325881	1.7834286247	0.9351854424
H	-5.8611370164	0.5965186716	2.0510158919
H	-6.1659279407	-0.6847771137	-3.9917201757
H	-3.1299716617	0.4880427418	0.4128869383
H	-8.3315397634	-0.3498741673	1.8480091297
H	-10.8599415959	1.8587733378	2.0433933724
H	-10.3549123262	0.2468831805	2.6097565416
H	-10.2181669121	1.6668548812	3.6704927852

calc_**2d** conf_17

C	-6.0924638137	-2.154971053	-0.2477241113
C	-6.9269471053	-1.2566389283	0.6732732929
C	-6.4827886149	0.1741011958	0.2746216687
N	-5.1826467546	0.03340915	-0.3948430934
C	-4.7801080668	-1.3834457328	-0.3350062018
C	-5.1205129384	0.4716051039	-1.8011159162
C	-3.9083191988	-0.2912871296	-2.3269950813
C	-3.88590536	-1.6406325259	-1.5774899933
O	-6.7354126917	-2.1846000519	-1.5082352494
O	-8.2903079551	-1.522030552	0.442826279
C	-4.9625262224	1.9912736375	-1.829552436
C	-6.3085638884	1.1349215292	1.4571233754
O	-4.2740449164	-2.7525258873	-2.3438114862
O	-3.9375412875	2.399750133	-0.9560550328
N	-7.4024079655	1.2040923958	2.4198957099
O	-9.1756836014	1.0097496959	1.0124401677
C	-8.7227554494	1.2220236878	2.1389304388
C	-9.6459180568	1.5002349915	3.302460067
H	-4.2235242595	-1.5719854037	0.5930679611
H	-5.9662994939	-3.1702163427	0.1485073492
H	-6.6686127481	-1.4555265506	1.7244087457
H	-7.2184874525	0.5842782293	-0.4221445085
H	-6.0283209663	0.1817807939	-2.349879137
H	-3.943472693	-0.4548414238	-3.4061926678
H	-3.0058661406	0.2837736612	-2.0962574348
H	-2.866220362	-1.8690350215	-1.2582170472
H	-7.6866406795	-2.1203993986	-1.2975762025
H	-8.7782257869	-0.6789588663	0.5413852915
H	-5.9220292383	2.465088393	-1.5618309503
H	-4.7092998267	2.3267908554	-2.8405029605
H	-6.0924986955	2.1355018515	1.0633494021
H	-5.4247550978	0.813217339	2.015762203
H	-5.2329135994	-2.6872070171	-2.485800733
H	-3.9651489729	1.7284291273	-0.2493491502
H	-7.1377003417	1.4244227445	3.3669151614
H	-9.1345148248	1.6354401641	4.258067379
H	-10.2221678793	2.4010955564	3.0773811404
H	-10.3564470542	0.6753216493	3.3901637611

calc_**2d** conf_18

C	-5.8083007856	-2.3600850022	-0.2509769972
C	-6.4452268333	-1.4665821354	0.8246668733
C	-6.3423803066	-0.0583361286	0.2184453318
N	-4.9900377057	-0.0931745462	-0.3260080259

```
C  -4.7915092912   -1.4290003987  -0.9315026545
C  -4.6364377735   0.9287487294   -1.3239001344
C  -4.152149915    0.1404004445   -2.5584095247
C  -4.8907226139   -1.1855657393  -2.4485814786
O  -6.8144814206   -2.82392146    -1.1410971631
O  -7.7345454219   -1.9444766026  1.0799457325
C  -3.5651292684   1.8767168694   -0.7894651686
C  -6.5054404893   1.0897913001   1.2230250468
O  -6.206834349    -0.9513756465  -2.8876400014
O  -3.2239847278   2.8852487715   -1.7177168451
N  -7.8733625766   1.542536249    1.412588227
O  -8.483589123    -0.0694985398  2.8790415234
C  -8.7532665839   0.9541468502   2.2468244636
C  -10.1007239246  1.6210399612   2.383671032
H  -3.7732794017   -1.7793473524  -0.7161145844
H  -5.3159329254   -3.2312644111  0.1957550023
H  -5.8195258343   -1.4941927995  1.7278846458
H  -7.1064457429   0.0470312514   -0.5709719141
H  -5.5224126709   1.5171692253   -1.5983330176
H  -4.3840290545   0.6399668357   -3.5014882664
H  -3.0682365744   -0.030405175   -2.5067991653
H  -4.4072224742   -1.9896157624  -3.0236372389
H  -7.6270864947   -2.8424624593  -0.6024486773
H  -8.1011689742   -1.4177039626  1.8214455482
H  -2.688596362    1.2855921454   -0.4811003778
H  -3.9222773602   2.401121074    0.1009745027
H  -5.9407518526   1.9564811621   0.8736407981
H  -6.0954114737   0.8033469763   2.19581766
H  -6.7332281565   -1.6805829833  -2.5094086944
H  -2.8205670103   2.453915183    -2.4813583172
H  -8.1773855977   2.3486489726   0.8910221779
H  -10.8762512852  0.9143940296   2.0786862193
H  -10.2677605348  1.8498353552   3.4387457599
H  -10.2033071099  2.5384597191   1.7997777023
```

calc_**2d** conf_19

```
C  -6.1688490112  -2.5258952433  -0.9973415445
C  -7.2379841881  -2.0304222508  -0.0241756826
C  -6.9220877869  -0.5171182075  0.1003324569
N  -5.4699345061  -0.4009885561  -0.1619033292
C  -4.9494547711  -1.7452836458  -0.5013522242
C  -5.0554353749  0.521227296    -1.2272199764
C  -3.6602784595  0.0015481131   -1.5681407294
C  -3.7682210215  -1.5231591124  -1.4673392775
O  -6.6306216469  -2.1152815266  -2.2676585166
O  -8.5428325884  -2.3078223729  -0.4357569254
C  -5.0518362039  1.947073743    -0.6744698725
C  -7.3536839592  0.0115981339   1.4660563751
O  -4.0539139863  -2.1510140813  -2.7170514998
O  -4.4839891732  1.9530489429   0.622163509
N  -7.2408666527  1.4411757458   1.6256890438
O  -9.4592563355  1.8005420353   1.9819872807
C  -8.3207591049  2.2311609208   1.8828120254
C  -8.0081215729  3.7049509894   2.0466574382
H  -4.592458421   -2.2391288496  0.4116459638
H  -6.0218221439  -3.6127553833  -0.9546644234
H  -7.1042113988  -2.5141397561  0.9508751292
H  -7.4819675921  0.0136592487   -0.6797744136
```

```
H  -5.7181601305  0.45865167     -2.1029075051
H  -3.3142406024  0.3286990066   -2.553343346
H  -2.9501093448  0.3734043921   -0.8220761118
H  -2.8504301813  -1.9651620747  -1.0632016721
H  -5.877508905   -2.2120356223  -2.8737518313
H  -8.5493669538  -2.141083635   -1.3917342955
H  -6.0799622841  2.3317557397   -0.6429221106
H  -4.4664816778  2.6097636182   -1.3206286087
H  -6.7692503994  -0.4994825336  2.2435956441
H  -8.4081868064  -0.2333887898  1.6182626377
H  -3.3151809342  -1.9858785362  -3.3155672332
H  -4.5539147212  1.0120098992   0.8926351424
H  -6.3203786666  1.861462813    1.5682916964
H  -8.2827182802  4.010845607    3.0594733178
H  -6.9589191045  3.9558545338   1.8720730118
H  -8.6346051089  4.2746077287   1.3564304571
```

calc_**2d** conf_20

```
C  -5.9762194467   -2.2194552154  -0.7668002244
C  -6.6819883319   -1.6276166645  0.4512249744
C  -6.5117986478   -0.1161879859  0.2249364738
N  -5.1584219397   0.0021390286   -0.3190590926
C  -4.7498386797   -1.3094806043  -0.8793131956
C  -4.9648458005   1.0511684106   -1.3378679581
C  -4.8769379736   0.298829312    -2.6716352432
C  -4.2309665478   -1.034687818   -2.3023578258
O  -6.898340861    -2.0778590487  -1.8305945824
O  -8.020132063    -2.0412721257  0.5789327019
C  -3.7165650493   1.8613882687   -0.9826850955
C  -6.7069943294   0.7143274519   1.4859554284
O  -4.5830798318   -2.1260414702  -3.1516476564
O  -2.6300071698   1.0207427465   -0.6601721887
N  -8.0596162466   0.5702787555   1.9736474462
O  -8.2803236008   2.7341700836   2.6175474692
C  -8.756939272    1.6157699826   2.4901929828
C  -10.1784670389  1.3006006051   2.9095222717
H  -3.9490514381   -1.7495529495  -0.2713852188
H  -5.6977325825   -3.2715919737  -0.6309316052
H  -6.1627590491   -1.9276156292  1.368203193
H  -7.2691834717   0.1883629831   -0.5156714636
H  -5.8262966695   1.7290834425   -1.3318876945
H  -5.8864111965   0.0985911146   -3.0443733591
H  -4.3233626058   0.8480573794   -3.4394934441
H  -3.1426332224   -0.9282858318  -2.2748206808
H  -6.4028590788   -2.2965938763  -2.6389713645
H  -8.3584287703   -2.0803191048  -0.3311070873
H  -3.9535496759   2.5310072902   -0.1429214449
H  -3.4009311687   2.4825347647   -1.8261640822
H  -6.5408809394   1.7780222037   1.2958380102
H  -5.9701444626   0.4012321684   2.2389945998
H  -4.1879472448   -1.9712606116  -4.0184185195
H  -2.953763858    0.4965542826   0.0889822201
H  -8.507203656    -0.3254843703  1.8331414773
H  -10.2760869678  1.4809102969   3.9830004205
H  -10.8550622774  1.9921183966   2.4021433305
H  -10.4862288344  0.2744163121   2.6940160275
```

calc_**2d** conf_21

C	-5.9101259237	-2.4665492471	-0.2756871712
C	-7.3324875582	-1.9380036472	-0.5833534188
C	-7.2392114508	-0.4163720686	-0.357677574
N	-5.8176584155	-0.1106913777	-0.1253403631
C	-4.9981853174	-1.2606121675	-0.5243692607
C	-5.2682035764	1.0428032699	-0.8433843626
C	-4.7480724588	0.5014304373	-2.1923445378
C	-4.5291994048	-1.001961218	-1.9838932897
O	-5.5982213429	-3.6053607362	-1.0590726491
O	-7.7362528307	-2.3118189241	-1.8824798409
C	-4.1526494678	1.6829264657	-0.0184051462
C	-8.1133627085	0.0082003253	0.8270870786
O	-5.2886576991	-1.7118363975	-2.9611206457
O	-4.5964113604	2.1582827845	1.2208466698
N	-8.0998157881	1.4244762004	1.1021206123
O	-6.8089706946	1.2621022398	2.957398907
C	-7.4407822847	1.9544771541	2.1696760582
C	-7.4979007913	3.4576451436	2.2936771277
H	-4.1080256353	-1.314062004	0.1120367666
H	-5.8448715306	-2.7341617594	0.7849900062
H	-8.0681804243	-2.3964318056	0.0836116576
H	-7.6132917924	0.0785461228	-1.2675322812
H	-6.0567003796	1.7906785851	-0.9690551714
H	-5.4920816959	0.6241882779	-2.9845123149
H	-3.8387885914	1.0130640355	-2.5211613674
H	-3.475526164	-1.2818498843	-2.096665417
H	-6.4375537405	-3.8308915786	-1.5013241259
H	-7.0769205214	-1.9202426573	-2.4955178034
H	-3.7528301114	2.5436416012	-0.5648448303
H	-3.3223554291	0.9650415717	0.0962221092
H	-7.7520432631	-0.4803746912	1.7350356375
H	-9.1453470101	-0.3149085742	0.6430984315
H	-5.1593960962	-2.647984142	-2.722751473
H	-5.0651751604	1.4345209703	1.670185301
H	-8.6087738084	2.0431488919	0.4914595379
H	-6.5341843769	3.8473960411	1.9543488779
H	-8.2983336054	3.919065083	1.709107973
H	-7.61745159	3.7204776793	3.3455902923

calc_**2d** conf_22

C	-6.2090323806	-2.3729013744	-0.7122601086
C	-6.9617465513	-1.6340572537	0.395344118
C	-6.526249093	-0.1689883179	0.1715590406
N	-5.1260418209	-0.279605731	-0.239047723
C	-4.8419538268	-1.6858730312	-0.616879644
C	-4.6518448697	0.6052679014	-1.3073297598
C	-3.4534877292	-0.1698183227	-1.8499557315
C	-3.9046819972	-1.6276033546	-1.8371373966
O	-6.9541364851	-2.0925954654	-1.8771377826
O	-8.3527307269	-1.8261941942	0.3477701821
C	-4.2660219953	1.9668668852	-0.7293268379
C	-6.729711859	0.6912796458	1.4157762026
O	-4.6214422541	-1.9793705235	-3.0206610586
O	-3.4116157123	1.80768905	0.3745528214
N	-8.1355533145	0.7696271712	1.7405431214
O	-8.0984460349	2.9711382065	2.290095621
C	-8.7216772788	1.9317726636	2.1338197055

C	-10.2149230276	1.851755803	2.3774295015
H	-4.2993774943	-2.1765666618	0.2027917554
H	-6.1401818545	-3.4547007249	-0.5360319423
H	-6.6262602046	-1.9757570981	1.3810206152
H	-7.1412059894	0.2389259493	-0.6457996157
H	-5.4156257171	0.7355422833	-2.0925190178
H	-3.1404725031	0.1638048725	-2.8446204827
H	-2.6119726209	-0.0288934393	-1.1643210753
H	-3.0666304996	-2.3242038995	-1.7180917476
H	-6.3423589165	-2.1438997527	-2.6307711188
H	-8.5738249982	-1.796721983	-0.5982917587
H	-5.174159394	2.527100548	-0.4607518272
H	-3.7333594626	2.5568114693	-1.4820730815
H	-6.3761013336	1.7147653464	1.2740505719
H	-6.1523687236	0.2611698592	2.2472615041
H	-4.0551908522	-1.8150574185	-3.7843835455
H	-3.7871469408	1.0489591518	0.8549366091
H	-8.6988274472	-0.0494922542	1.5556312994
H	-10.7101407129	2.612586429	1.7697283528
H	-10.6485064228	0.8741907439	2.1525438663
H	-10.4129909548	2.0940468212	3.4245363673

calc_**2d** conf_23

C	-5.5629187627	-2.0753975513	0.0414990775
C	-6.8704985612	-1.2828870057	0.3223956183
C	-6.5388658965	0.1742307053	-0.0456719756
N	-5.1786300018	0.1710739246	-0.6045331558
C	-4.7713784193	-1.1976500146	-0.9362728146
C	-4.9624415658	0.9939353544	-1.801192542
C	-4.9735092143	0.0345256018	-3.0139422077
C	-5.0544634994	-1.3848420625	-2.442104673
O	-5.8441702982	-3.3688525169	-0.4646408039
O	-7.945825551	-1.8195539761	-0.4231591367
C	-3.6389504643	1.7428607912	-1.6455847302
C	-6.5913010052	1.1416467503	1.1415234278
O	-6.354552645	-1.9134079626	-2.6968132745
O	-3.6013195113	2.4328284837	-0.4243472569
N	-7.9147002813	1.3120145376	1.6950289334
O	-7.6174316468	-0.1277355234	3.4223717212
C	-8.331913209	0.6553906659	2.8134185712
C	-9.7500849399	0.9488813627	3.2517513478
H	-3.6914349724	-1.3085290871	-0.7784634084
H	-4.9924792341	-2.1706319972	0.9721496825
H	-7.1628261242	-1.3757137857	1.369302398
H	-7.2780594066	0.5008130562	-0.7939177292
H	-5.760681123	1.7413657741	-1.8571688522
H	-5.8321332441	0.1977828801	-3.6690421596
H	-4.0718507695	0.162403253	-3.6217034033
H	-4.3124500739	-2.0508164471	-2.897157148
H	-6.8045773918	-3.4740332095	-0.3285054261
H	-7.7169494032	-1.6686437111	-1.3622833728
H	-3.5223135612	2.4789068942	-2.4468368281
H	-2.802244426	1.0283255355	-1.7315857092
H	-6.1982000657	2.1145950655	0.8272291493
H	-5.9527697199	0.7647304633	1.9439570607
H	-6.3119342007	-2.802143967	-2.2988479425
H	-3.8784725202	1.7635053218	0.2241384485
H	-8.5632749942	1.917310258	1.2186285446

```
H  -9.7446459828   1.1736386619   4.3199887262
H  -10.2192147425  1.7760877233   2.7132309847
H  -10.3545325709  0.0489857535   3.1101608585
```

calc_**2d** conf_24

```
C  -5.9161705488  -2.6309320839  -1.0348949114
C  -6.8598739484  -2.1869187109  0.1096384641
C  -6.6906515814  -0.6620525708  0.160069946
N  -5.2343457932  -0.5369586957  -0.0257992292
C  -4.8498659528  -1.5148865492  -1.067593382
C  -4.6952880798  0.7779475333   -0.4221324964
C  -4.0249474578  0.577197197    -1.7979123273
C  -4.6802777831  -0.6831620377  -2.3459050827
O  -6.6501626012  -2.8206726807  -2.2276912769
O  -8.1657580226  -2.6326190431  -0.0805711147
C  -3.6999614074  1.2594148243   0.6363110449
C  -7.2326961504  -0.0970551059  1.4673010036
O  -5.9745281032  -0.3713236344  -2.8696186317
O  -4.2284462186  1.1211511863   1.9374851576
N  -7.1129093736  1.3334313524   1.6254955159
O  -9.2911887144  1.796112203    1.1698990877
C  -8.1726418572  2.174163886    1.4831610052
C  -7.8712408254  3.6362912827   1.7475850936
H  -3.8610492543  -1.9298771779  -0.8361525439
H  -5.4643587336  -3.6004952101  -0.8144045572
H  -6.4885149977  -2.5958505891  1.0586966586
H  -7.2450334677  -0.2041332476  -0.6736443702
H  -5.5215651456  1.492529347    -0.4934284793
H  -4.1553444325  1.4348448568   -2.4644718409
H  -2.9468211807  0.4125639088   -1.6828932421
H  -4.0780883097  -1.2002123956  -3.1024189284
H  -6.7061393532  -1.9557903035  -2.6758485634
H  -8.2271509066  -2.8013647244  -1.0384310867
H  -3.4556952269  2.3160075647   0.4943621131
H  -2.7648523929  0.6850379623   0.5459495501
H  -6.7343289602  -0.5940179097  2.3103603454
H  -8.2969137183  -0.3412417355  1.5120200447
H  -5.8662578759  0.1442403819   -3.6787851377
H  -4.5127618686  0.1899559743   1.9628211796
H  -6.2032482527  1.6876169751   1.8958088976
H  -6.8208618718  3.838034347    1.9722490921
H  -8.1716902249  4.2213311398   0.8751809933
H  -8.4833694067  3.9726924829   2.5882020091
```

calc_**2d** conf_25

```
C  -5.5780346943  -1.8133931026  0.4026930847
C  -7.0759386899  -1.4806999631  0.1328518883
C  -7.069056912   -0.1219523968  -0.5933815804
N  -5.6742702187  0.3323646102   -0.5748807082
C  -4.7940806619  -0.8354901699  -0.4773658015
C  -5.2458074432  1.0774711593   -1.762379434
C  -4.6342300895  0.0386024429   -2.7321644018
C  -4.4986622459  -1.2652859459  -1.9291293268
O  -5.2887747729  -3.1708273585  0.1199023283
O  -7.6784747201  -2.4999378415  -0.6425443502
C  -4.2559651208  2.1546120244   -1.2950785901
C  -8.028880309   0.9386178252   -0.034363244
```

O	-5.4456400136	-2.2153455156	-2.4121887459
O	-4.7400696649	2.7666375941	-0.1246809243
N	-7.5908989829	1.5948971689	1.1753235228
O	-8.5610518228	0.1311487258	2.6084999162
C	-7.9189714327	1.1548527201	2.4191159004
C	-7.4507623777	2.027473923	3.5638140112
H	-3.8486598673	-0.5414691879	-0.005476221
H	-5.3473408105	-1.6069026993	1.453784964
H	-7.6446821855	-1.4371301674	1.0622107812
H	-7.4074302114	-0.3197499684	-1.6240245849
H	-6.1179798716	1.5817598563	-2.190018261
H	-5.2748424201	-0.1511879473	-3.5963511062
H	-3.6649039416	0.3753746763	-3.1129732847
H	-3.4930359695	-1.693241114	-2.0156103447
H	-6.1665354376	-3.5938779682	0.0623416619
H	-7.2642700429	-2.4332683862	-1.5234564542
H	-4.1314204839	2.9244196203	-2.0620375264
H	-3.2658701887	1.6995504618	-1.1228862241
H	-8.9974708046	0.4698173841	0.1592780604
H	-8.1821790432	1.7163615809	-0.7890826205
H	-5.2904348625	-2.9874078853	-1.8373237267
H	-5.0776251547	1.9956820208	0.3740764872
H	-7.0440873138	2.4351014957	1.0738415038
H	-6.8036589693	2.852518513	3.2565467508
H	-8.3275054989	2.4369666973	4.0723039418
H	-6.9194967499	1.4039371174	4.2858126588

calc_**2d** conf_26

C	-5.4670990404	-2.3552447145	-0.3476703062
C	-6.6541608154	-1.6909972695	0.407888456
C	-6.3289311642	-0.1880031402	0.4211473206
N	-5.0404719028	-0.0372227007	-0.2757449615
C	-4.8248009096	-1.2070316963	-1.1326923203
C	-4.9664145476	1.1396811659	-1.154367374
C	-5.4565829211	0.6741710081	-2.5422671295
C	-5.430978266	-0.8610362417	-2.5145705697
O	-5.9067029232	-3.4320449117	-1.1529498876
O	-7.8785776012	-2.0040443165	-0.2229651183
C	-3.5230050715	1.6438418512	-1.156868998
C	-6.2269701177	0.4361968275	1.8146538267
O	-6.76364718	-1.3469370148	-2.6771674648
O	-3.0616529005	1.8123433771	0.1568422303
N	-7.5068419041	0.5702382974	2.4720827319
O	-8.0722864516	2.4081848239	1.2833010816
C	-8.3676597573	1.5653121275	2.1173213106
C	-9.7073586999	1.5605115189	2.8200668343
H	-3.749211783	-1.3727461096	-1.2626832568
H	-4.7369185372	-2.7280810719	0.3801539145
H	-6.7435935385	-2.1005785381	1.4181871623
H	-7.1360256121	0.3361668576	-0.1090424453
H	-5.59928096	1.9254179685	-0.7309431901
H	-6.4831965182	0.9935909134	-2.7374235128
H	-4.8334276776	1.0772626697	-3.3464035111
H	-4.8119772014	-1.2744709368	-3.3189628058
H	-6.8412359952	-3.5524040637	-0.8968122718
H	-7.837275873	-1.5660055604	-1.0980240542
H	-3.4652676722	2.6126808438	-1.662123815
H	-2.8846852832	0.9425436874	-1.7232715804

H	-5.7884046268	1.4328668953	1.71851444
H	-5.5618347471	-0.1604300129	2.4465163213
H	-6.6609112647	-2.3143557691	-2.6394000442
H	-3.3960661317	1.0221566215	0.6161361304
H	-7.823829996	-0.1571112893	3.0912720904
H	-9.8998366208	2.5620671573	3.2095496591
H	-10.4891442086	1.3386446201	2.0888023185
H	-9.7747335787	0.8388661257	3.6379187885

calc_**2d** conf_27

C	-5.946048462	-2.4270695024	-0.7774102027
C	-6.4896020628	-1.7497059987	0.5026016114
C	-6.3456816641	-0.2506971487	0.2096475535
N	-5.0177111382	-0.2240740314	-0.4077111776
C	-4.952992104	-1.3780895826	-1.3276847797
C	-4.6312810713	0.9835766316	-1.1603468315
C	-4.4237847907	0.5310649903	-2.6220811666
C	-5.1868147257	-0.7831527885	-2.7237099984
O	-7.0208267445	-2.7780621041	-1.6269150958
O	-7.7896947104	-2.1753115744	0.8079994336
C	-3.3592487061	1.5791923681	-0.5599470606
C	-6.4635620145	0.6106922692	1.4600755473
O	-6.585895677	-0.5268215338	-2.8880738913
O	-3.4909895618	1.8065748407	0.817014022
N	-7.8043121137	0.52018972	1.9881739727
O	-8.0638378527	2.7527575683	2.3181313734
C	-8.5171013421	1.6187964259	2.3544972443
C	-9.9275278685	1.3392026868	2.8332579921
H	-3.9361377575	-1.7894145045	-1.3337294785
H	-5.4393178883	-3.3651491899	-0.5407359194
H	-5.8393390302	-1.9934027524	1.3514597224
H	-7.1362248738	0.0537529252	-0.496549826
H	-5.4364786047	1.7232790985	-1.0880785902
H	-4.773405586	1.2688461574	-3.3502562391
H	-3.3595985385	0.3549604653	-2.8208657491
H	-4.8269396491	-1.4418768736	-3.5230305435
H	-7.1956717322	-2.0127656064	-2.2076900639
H	-8.1393491545	-2.5002522052	-0.0436948712
H	-3.1322055483	2.5426334618	-1.0268730641
H	-2.5160999737	0.8999318932	-0.7734653976
H	-6.2617525057	1.665745377	1.2610278041
H	-5.7272801787	0.2703308089	2.2038105511
H	-6.730361389	-0.1489038801	-3.7648222174
H	-3.7724142313	0.9524278164	1.1796579341
H	-8.2492843325	-0.3871639837	1.9541603815
H	-10.0088639025	1.6433031925	3.8799579352
H	-10.6211426649	1.9592868676	2.2609630131
H	-10.2252198485	0.2913676956	2.7462360727

calc_**2d** conf_28

C	-5.8290899899	-2.4473049411	-0.5654778865
C	-6.449089333	-1.6916801757	0.6232998443
C	-6.417606393	-0.2291248607	0.1599292783
N	-5.0875052059	-0.154878729	-0.4305522837
C	-4.8473106599	-1.4234795848	-1.1601909889
C	-4.8105760657	0.9726771055	-1.3479771349
C	-4.240430644	0.3103102609	-2.6119596301

```
C  -4.9352145681   -1.0407971079  -2.6480280439
O  -6.9103741143   -2.8111298627  -1.4426150581
O  -7.7064359607   -2.1887796973  0.9987244477
C  -3.8212067552   1.9303544298   -0.6824996757
C  -6.6194883067   0.7647567138   1.2956367604
O  -6.2536976457   -0.8046239919  -3.087935375
O  -2.7292175645   1.2154885159   -0.1554708737
N  -7.9345792336   0.6173822202   1.8732309782
O  -8.3254683755   2.8442340188   2.0678068743
C  -8.7018364303   1.6900252576   2.2049784886
C  -10.0677632213  1.3571836359   2.7693443588
H  -3.8198448621   -1.7611027414  -0.9707238852
H  -5.3228898041   -3.3610703969  -0.2450003846
H  -5.7887406899   -1.7903411353  1.4914880648
H  -7.2096459564   -0.0746817089  -0.5940751666
H  -5.7435030908   1.4978131656   -1.5889256624
H  -4.446983423    0.8876537652   -3.5154985918
H  -3.1579764925   0.1866261946   -2.5049729188
H  -4.4194689132   -1.7724322876  -3.288801558
H  -6.8009287583   -3.7328389772  -1.700767764
H  -8.1466031862   -2.4187422365  0.1639835584
H  -4.3308380605   2.511652812    0.100813415
H  -3.4266616963   2.640836673    -1.4153285058
H  -6.5423050717   1.79707593     0.9456638292
H  -5.8303440168   0.613166764    2.0461642732
H  -6.7776641373   -1.5723309754  -2.8112113985
H  -3.1547993187   0.4976680779   0.3440442631
H  -8.2976679141   -0.3210489519  1.9643187114
H  -10.2898484665  0.2873288962   2.7894540692
H  -10.1325589216  1.7509884696   3.7867238696
H  -10.8268367526  1.8701654558   2.1744077018
```

calc_**2d** conf_29

```
C  -5.7092967461  -2.364273284   0.0974676663
C  -6.9974271933  -1.5874053243  0.4601523402
C  -6.9591771908  -0.3967064428  -0.505940788
N  -5.5501817011  -0.0144382337  -0.4582319425
C  -4.7284093534  -1.234995659   -0.2691715585
C  -4.9906501182  0.8036318265   -1.5399222551
C  -3.6018207263  0.1898519669   -1.7672944089
C  -3.8427175296  -1.2953740059  -1.5159401737
O  -6.0246294703  -3.2888568537  -0.9257691095
O  -8.1364029181  -2.3806379288  0.3442671344
C  -4.9503586139  2.2703341698   -1.0801191307
C  -7.9288051495  0.7329585369   -0.1654704849
O  -4.5960587352  -1.847434057   -2.5989937106
O  -4.5917764165  2.3262121769   0.2833367049
N  -7.5778327632  1.4653842526   1.0272199755
O  -9.5794902581  1.1723684152   2.0687193867
C  -8.463975876   1.6647699565   2.0466466055
C  -7.9544645868  2.5424834056   3.1721066022
H  -4.0485059592  -1.0860235421  0.5800253065
H  -5.3398220146  -2.9451637544  0.9476039218
H  -6.941228036   -1.2114096353  1.487503999
H  -7.2329939878  -0.7711769581  -1.5089156582
H  -5.5921855369  0.7101973078   -2.454513199
H  -3.2096514887  0.3935767349   -2.7680395956
H  -2.8917396023  0.5890891315   -1.0342563823
```

H -2.9284626486 -1.876459856 -1.3495793961
H -5.6587527392 -2.9545543533 -1.7636700931
H -7.9183855628 -3.0061046619 -0.3698439703
H -5.9315164388 2.7393041552 -1.2436870796
H -4.2166637924 2.8389080338 -1.6616674466
H -8.9177517356 0.300415783 0.0058433367
H -8.0131580159 1.419946041 -1.0182490652
H -4.0119210792 -1.9666645233 -3.3578786225
H -4.8591587982 1.4367723268 0.6041218523
H -6.7183175424 1.9961439154 1.0230109473
H -6.9343261664 2.9055925394 3.0247015896
H -8.6255251067 3.3980184988 3.2803554283
H -8.0004584022 1.9757198989 4.1050712737

calc_**2d** conf_30

C -6.0653929182 -1.989323005 -0.0264388785
C -6.9726287785 -0.9710961214 0.6687852872
C -6.6579697913 0.3419950455 -0.0966832186
N -5.291573431 0.1889092553 -0.6155303732
C -4.8010318258 -1.1550235505 -0.2462355377
C -5.0981743305 0.3680471757 -2.0576031939
C -3.7720538542 -0.3522292793 -2.2865329511
C -3.8164084361 -1.570668788 -1.3585994478
O -6.7513462959 -2.3084924624 -1.222071305
O -8.327186271 -1.3348672132 0.6314285752
C -5.0443814379 1.8618195468 -2.372355913
C -6.7792413922 1.5851737898 0.7885714375
O -4.298935407 -2.7515217197 -2.003008561
O -4.1349693444 2.5148470333 -1.5239230233
N -8.0624841744 1.6771024981 1.4470353989
O -7.2897716971 1.0217374049 3.4828552556
C -8.2282991899 1.2793020973 2.7446857014
C -9.6690068409 1.1646869591 3.1899057803
H -4.272594265 -1.1007753099 0.7143375629
H -5.8831031683 -2.8851027491 0.5809784874
H -6.6956820856 -0.8627579967 1.7217491348
H -7.368709043 0.4113562263 -0.9331921242
H -5.9040808089 -0.109171252 -2.638069577
H -3.6145931941 -0.6350643815 -3.3320247705
H -2.958383937 0.3160350132 -1.9883536869
H -2.8293106744 -1.7919446536 -0.9372871933
H -6.106444014 -2.7587683925 -1.793371722
H -8.4423307886 -1.7709856127 -0.2289518655
H -6.0570037841 2.2875814147 -2.2787060193
H -4.7211748138 2.0233757365 -3.4057814385
H -6.5933767738 2.491850314 0.20130516
H -6.0297535172 1.5411540375 1.5816202779
H -3.6923789878 -2.9732755352 -2.7200167429
H -4.2436914249 2.0560742559 -0.6707454099
H -8.8855214469 1.6908780057 0.8641296099
H -9.7128738568 1.2514717802 4.2751469688
H -10.3118438482 1.9231159449 2.7343716069
H -10.0452941511 0.177554488 2.9025767083

calc_**2d** conf_31

C -6.6496554516 -2.0651289004 -1.1387503033
C -7.4045908078 -1.4484756313 0.0344853538

```
C  -6.5290939206  -0.2197915711  0.3852711161
N  -5.1646859251  -0.6203762902  0.0349970174
C  -5.2069117581  -1.9220916808  -0.6677677651
C  -4.4119225881  0.3211096624   -0.794926763
C  -3.3460311234  -0.5733378348  -1.4177114577
C  -4.073599488   -1.8968855616  -1.7277611331
O  -6.8971230505  -1.248075193   -2.2718592023
O  -8.7035475222  -1.1372938534  -0.3835733643
C  -3.8458370317  1.4669071958   0.0348441378
C  -6.6482433012  0.2425131935   1.8562908858
O  -4.4970040811  -2.0104303141  -3.0614500808
O  -3.1238416235  2.3989567559   -0.7395736733
N  -7.3997111569  1.4704779538   2.0460715019
O  -9.4235117629  0.5481407919   1.6377299627
C  -8.742544923   1.5302029383   1.936263709
C  -9.3853278432  2.8669647993   2.2159559061
H  -5.0222181371  -2.7301436576  0.0535086878
H  -6.939759007   -3.1041625436  -1.3376673946
H  -7.4124661054  -2.1534060884  0.8808654277
H  -6.8576350868  0.6091479609   -0.2596727077
H  -5.0448740737  0.7509144814   -1.5909384014
H  -2.9028483834  -0.1661272063  -2.3293119587
H  -2.5490847549  -0.7526504131  -0.6838078142
H  -3.3955143888  -2.7401534961  -1.5709379424
H  -7.8193236995  -0.9505061785  -2.1734669776
H  -9.1055469096  -0.5707668938  0.3090296332
H  -3.2378107737  1.0439525528   0.8507132961
H  -4.6585823752  2.040351837    0.4933866908
H  -5.6442574452  0.4098417838   2.2480346488
H  -7.1124099381  -0.5385741298  2.4677367992
H  -5.3416926445  -1.5391667008  -3.1516780303
H  -2.366416398   1.9354025462   -1.1183352855
H  -6.8955452293  2.3136299587   2.2669201887
H  -8.6730285202  3.6554025351   2.469090807
H  -9.9598239986  3.1720178523   1.3383491145
H  -10.091978772  2.7476093397   3.0406453709
```

calc_**2d** conf_32

```
C  -5.9930970126   -2.3858075241  -0.4821548234
C  -6.7149433199   -1.7055423443  0.6775784644
C  -6.3274588926   -0.2210942723  0.4966745102
N  -5.0310326472   -0.2230510195  -0.1822211477
C  -4.6685099202   -1.6260024036  -0.4791761666
C  -4.9337077945   0.5378041604   -1.4366712599
C  -3.7144381142   -0.0981554509  -2.0927689082
C  -3.832181599    -1.6027694715  -1.7850325355
O  -6.732437513    -2.1113678676  -1.658903729
O  -8.0975520079   -1.9340276071  0.5788947554
C  -4.7798645138   2.0373696545   -1.2221342789
C  -6.3231371025   0.5483739545   1.8381599181
O  -4.3667771904   -2.3631683022  -2.8393074767
O  -5.980243588    2.7094056944   -0.8971398889
N  -7.1620460828   1.7394559595   1.8112054225
O  -9.0584111643   0.5238005023   1.6006829529
C  -8.5169641718   1.6143329131   1.7563521034
C  -9.3190622936   2.8870170817   1.8544071466
H  -4.0679738451   -2.0306968162  0.347559487
H  -5.8700111788   -3.4667569799  -0.3409332732
```

H	-6.3191410644	-2.087696799	1.6319188479
H	-7.0862117055	0.2281692545	-0.1578659456
H	-5.8256788545	0.378477809	-2.0658222421
H	-3.6641878341	0.0720703303	-3.1702663364
H	-2.8041824464	0.3079935026	-1.6345373847
H	-2.8422597272	-2.0298002425	-1.6051229534
H	-7.66424508	-2.1023873242	-1.3728698923
H	-8.5529034294	-1.1649684535	0.9750776001
H	-4.4376627077	2.4951064855	-2.1550624483
H	-3.998800943	2.2033784856	-0.4631093334
H	-5.3013536833	0.8619256357	2.0558338688
H	-6.6636309932	-0.099713551	2.6541856388
H	-5.3290360834	-2.23208181	-2.8452425519
H	-6.3391232294	2.3290783573	-0.0834055048
H	-6.7795320926	2.6102183044	2.1474325933
H	-8.7224929367	3.7623590422	2.1199375303
H	-9.7983633804	3.0717473281	0.8894346526
H	-10.1113438566	2.7480037838	2.5924125884

calc_**2d** conf_33

C	-6.1419698968	-2.4843683935	-0.8050300283
C	-7.5610627393	-2.0075434327	-0.4752176282
C	-7.4277872035	-0.4600611009	-0.3527622728
N	-6.0063226519	-0.1770653516	-0.2182986302
C	-5.2752536335	-1.4368713901	-0.1037933321
C	-5.345363169	0.7445466807	-1.1476094752
C	-4.1564738909	-0.0383140533	-1.7257860533
C	-3.9048109849	-1.1750901096	-0.7340187706
O	-6.031892793	-2.4335303258	-2.2164229396
O	-8.5170982958	-2.4335434497	-1.4067079913
C	-4.9160419847	2.0130068236	-0.405157509
C	-8.2339700343	0.0279504711	0.8561266354
O	-3.440756347	-2.3824857636	-1.3372708365
O	-4.1932804312	1.7206262293	0.761432806
N	-8.1852509785	1.4552955415	1.0851012259
O	-6.5127285612	1.3758179513	2.6174969975
C	-7.3306132821	2.0239453219	1.9737672572
C	-7.4509487013	3.5229288476	2.1412515525
H	-5.1646123396	-1.7303738455	0.9495450845
H	-5.9494565141	-3.5007621537	-0.4413830294
H	-7.85850031	-2.4205556668	0.4963676602
H	-7.8258891134	-0.0017201826	-1.2680787282
H	-6.0357046302	1.0330214026	-1.9537798175
H	-4.4345887835	-0.475023625	-2.6880844522
H	-3.2731476778	0.5904983856	-1.8707217327
H	-3.2034331802	-0.8497095138	0.0410826988
H	-5.1105951311	-2.6764997462	-2.4075585965
H	-8.0401989999	-2.4773825035	-2.2516727635
H	-5.8097270343	2.6171557378	-0.1853850836
H	-4.2669647787	2.6201490556	-1.0465360434
H	-7.835186367	-0.4430111395	1.7581251746
H	-9.2804958965	-0.2768425983	0.7419994154
H	-2.5448540429	-2.2268797109	-1.6606452832
H	-4.8317575818	1.3145488566	1.3750607781
H	-8.8266419956	2.0481476313	0.5839472854
H	-8.1700074105	3.992390451	1.4649751375
H	-7.7430390166	3.7364369561	3.1725947255
H	-6.4655736174	3.9671677128	1.984046563

calc_**2d** conf_34

C	-6.0426694448	-2.308324148	-0.413987464
C	-6.7525319323	-1.599886451	0.7353940151
C	-6.3780440999	-0.1161160794	0.4949865744
N	-5.0592195483	-0.1586637394	-0.1395739029
C	-4.7117317126	-1.5661016645	-0.440070402
C	-4.904146007	0.6142984565	-1.3714453074
C	-3.7028572888	-0.0492811699	-2.0303903975
C	-3.888232374	-1.5532081234	-1.7560271835
O	-6.7924531908	-2.0477679104	-1.5905768711
O	-8.1234871089	-1.8721150548	0.6528863808
C	-4.7177563731	2.0987149644	-1.0899597749
C	-6.3891552028	0.7526721346	1.7748055278
O	-4.4633376906	-2.2620949925	-2.8251227738
O	-4.4853318093	2.8496266973	-2.2616189971
N	-7.4879309666	1.6986546983	1.8551825434
O	-9.0812283584	0.1398247184	2.2363970521
C	-8.7591597484	1.3207147407	2.1006225221
C	-9.7823716311	2.4242236915	2.2195075818
H	-4.1010370341	-1.9710818033	0.3785557097
H	-5.9342819565	-3.3879113567	-0.2529366662
H	-6.3325650876	-1.9398442873	1.6952271367
H	-7.121383639	0.2971018144	-0.2035212462
H	-5.7941833763	0.4920896785	-2.0214817067
H	-3.6242408871	0.1472954408	-3.100300801
H	-2.7875412293	0.3118672618	-1.5471380992
H	-2.9161944545	-2.0263428765	-1.5930114327
H	-7.7223972045	-2.047327898	-1.3003318555
H	-8.5834310314	-1.2397132705	1.2448447369
H	-3.837857221	2.2347857224	-0.4545929949
H	-5.5893258581	2.4910130843	-0.5377339897
H	-5.461726038	1.3252516857	1.8087349217
H	-6.4175558951	0.119796189	2.6683622686
H	-5.4255610817	-2.1381305317	-2.786732137
H	-5.2290407068	2.6961959366	-2.8584840575
H	-7.2886989455	2.6785603235	1.7378998157
H	-9.3715605596	3.4255610472	2.0720016236
H	-10.5748182203	2.2506601762	1.4879763193
H	-10.2389550855	2.3700028949	3.2106533307

calc_**2d** conf_35

C	-6.0490635234	-2.5035971429	-0.8684991064
C	-6.5454842935	-1.8802201993	0.4547269652
C	-6.3629038728	-0.376065721	0.2192786056
N	-5.0498919995	-0.3476142272	-0.4082739138
C	-4.9648094192	-1.5040854164	-1.326025574
C	-4.6659566686	0.8683024298	-1.1334893273
C	-4.1989126252	0.377614649	-2.5163580885
C	-5.0128416897	-0.8915038217	-2.729267961
O	-7.1547807896	-2.6831417992	-1.7333662999
O	-7.8553700778	-2.2764122111	0.7615828745
C	-3.5778435079	1.6420536116	-0.3942754891
C	-6.4373558471	0.449317999	1.495702524
O	-6.3700243027	-0.5506556932	-3.0344569653
O	-3.1256082694	2.7609289241	-1.1307493541
N	-7.7615214295	0.3643798629	2.0668603667

O	-7.966031239	2.5931656711	2.4493732241
C	-8.4445309918	1.467677445	2.4696239001
C	-9.8531103062	1.2103737627	2.9642338197
H	-3.978916471	-1.9772402118	-1.2302170644
H	-5.627447327	-3.4986446655	-0.705510125
H	-5.8930501616	-2.1851373436	1.2811049844
H	-7.1600220081	-0.0276016874	-0.4641554329
H	-5.5467646771	1.5181882472	-1.2591496086
H	-4.3489059223	1.1202666718	-3.3040397235
H	-3.1295868214	0.13873893	-2.4950060427
H	-4.6062464702	-1.5611211854	-3.4964262036
H	-7.1943895763	-1.9125165689	-2.3307129431
H	-8.2493430517	-2.4698309099	-0.1104799523
H	-2.7105753949	0.9897870866	-0.2567907228
H	-3.9296554558	1.9293541437	0.6058060334
H	-6.245364841	1.5089446606	1.3076235417
H	-5.6720731825	0.0930689792	2.19885016
H	-6.4007664261	-0.1606629915	-3.9169569234
H	-3.8176784961	3.433082101	-1.0972288988
H	-8.2284375808	-0.5306858631	2.0039698662
H	-10.1609507214	0.1640887607	2.8970724507
H	-9.9237535267	1.5341677264	4.0055705334
H	-10.5460310352	1.8262359969	2.3860558709

calc_**2d** conf_36

C	-6.0873007394	-2.2393437744	-0.4301355422
C	-6.8031149682	-1.4909392464	0.6879494615
C	-6.3842449203	-0.0216068199	0.4251245755
N	-5.0493263578	-0.1125726972	-0.1718280527
C	-4.7360753743	-1.5353504823	-0.4360907567
C	-4.8488262928	0.6300220483	-1.4187014662
C	-3.6661221911	-0.0886637084	-2.054870286
C	-3.8830811415	-1.578581561	-1.7320341267
O	-6.7968442354	-1.9817460655	-1.6324543377
O	-8.1771210779	-1.7380960257	0.5847432587
C	-4.5925942526	2.1023275788	-1.1514257379
C	-6.3947922247	0.8752441544	1.6827964424
O	-4.4413690546	-2.3237268373	-2.7849327684
O	-4.3952372218	2.7396630963	-2.3955629828
N	-7.574534253	1.7118210637	1.8258526726
O	-8.9653574903	0.0526414351	2.4807450561
C	-8.7723459144	1.2433269268	2.2307694847
C	-9.8789253952	2.2596655897	2.3809970409
H	-4.1585734138	-1.942018205	0.4055095692
H	-6.0138996772	-3.3180412303	-0.2451510337
H	-6.4101084304	-1.8190465879	1.6626408504
H	-7.0980431364	0.3980402291	-0.2999227559
H	-5.7315375472	0.5490025613	-2.0763100983
H	-3.5891320931	0.0752922584	-3.1300540437
H	-2.7389542931	0.2627422803	-1.5862583782
H	-2.9224411812	-2.0592380414	-1.5285503955
H	-7.7359685235	-1.9674670368	-1.3790661319
H	-8.6162403041	-1.1990809781	1.2750322253
H	-3.7124195506	2.1882696092	-0.4957384838
H	-5.4514217151	2.5428833612	-0.6165502972
H	-5.5307559299	1.5398098517	1.642301678
H	-6.2956802519	0.2674690853	2.5879427262
H	-5.3975121952	-2.1553758617	-2.7971560928

H -4.148027069 3.6562888268 -2.2284009091
H -7.4944932442 2.6901260808 1.6015565198
H -9.5740685724 3.2838665264 2.1543790497
H -10.7062200879 1.9794327961 1.724680428
H -10.2512896786 2.2179597995 3.4071736382

calc_**2d** conf_37

C -6.2606958986 -2.1180172632 -0.347343491
C -7.067594402 -1.2308180028 0.6063024784
C -6.4415380678 0.1735502605 0.4033857353
N -5.1164299513 -0.0392222048 -0.1850750419
C -4.8730223921 -1.491030328 -0.2530814057
C -4.8877799137 0.5191689578 -1.5212813873
C -3.7184086559 -0.3115939824 -2.0388538994
C -3.9100507569 -1.7244533703 -1.4472490105
O -6.7986101278 -1.9444994585 -1.6458818582
O -8.4296964014 -1.3116239735 0.2538257096
C -4.5922795153 2.0111760003 -1.4502587622
C -6.2705813943 0.9722682556 1.7031813297
O -4.3512725837 -2.6960728815 -2.3624008816
O -4.2914277339 2.563662458 -2.7142685925
N -7.4119572503 1.002149814 2.6122574116
O -9.0833820245 1.2268304331 1.0930174006
C -8.700757195 1.1998210035 2.2646837638
C -9.6808187224 1.3656185722 3.4029548522
H -4.4195102965 -1.8363511734 0.6866842376
H -6.2744957534 -3.1768455351 -0.0598994486
H -6.919578151 -1.5667497587 1.6437080122
H -7.0768492434 0.7373431165 -0.2864873261
H -5.7694869109 0.3609915407 -2.168972254
H -3.6644283347 -0.3504798086 -3.1277349466
H -2.7808828999 0.1225994392 -1.6741400477
H -2.9524200861 -2.1042757136 -1.0831358048
H -7.751240046 -1.7931059268 -1.4957057862
H -8.8302214381 -0.4339049777 0.4164180484
H -3.7089858389 2.1686866029 -0.824821203
H -5.4322930703 2.5440291281 -0.9749177125
H -5.972797716 1.996143022 1.4480480681
H -5.4404185947 0.5269561349 2.2573243559
H -5.2834914337 -2.5067266675 -2.5591553614
H -5.0662407628 2.4403469919 -3.2772920247
H -7.1950801066 1.0258934782 3.5959073872
H -10.4429627498 0.5868147249 3.3248298084
H -9.2263020695 1.3220228383 4.395269666
H -10.1870115108 2.3266982536 3.2851579808

calc_**2d** conf_38

C -6.1321059088 -2.5583991595 -1.0243446451
C -7.3522645926 -2.0785397613 -0.2330274138
C -7.0694836792 -0.5647090439 -0.0209337768
N -5.6152569417 -0.3946957532 -0.1623945333
C -5.0196712378 -1.7253839891 -0.3795896047
C -5.1342539001 0.4724960669 -1.23624833
C -3.6767042829 0.0358806751 -1.353784672
C -3.7210103046 -1.4857792457 -1.1761908196
O -6.4099282237 -2.1945666889 -2.3628133869
O -8.5663130549 -2.3573564897 -0.8688452135

C	-5.3116341873	1.9525792251	-0.947639901
C	-7.5510731578	-0.0897229404	1.3472526885
O	-3.7882713945	-2.1971458805	-2.4142058889
O	-4.646423762	2.2933977192	0.2605254312
N	-7.1730593185	1.2769371776	1.6056031852
O	-9.2227525842	1.9684282746	2.3118453904
C	-8.0456593914	2.2027359504	2.0801901948
C	-7.4537490345	3.5799739002	2.3138862466
H	-4.783229209	-2.1867155142	0.5890075384
H	-5.9643067761	-3.6395075595	-0.9330684236
H	-7.3816495134	-2.5840773025	0.7393890019
H	-7.5931573135	-0.0024537004	-0.8046708349
H	-5.662006114	0.2710861319	-2.1848927241
H	-3.2149896706	0.3283654022	-2.3025728499
H	-3.1095165152	0.4908930138	-0.5373911908
H	-2.8505679768	-1.8503265425	-0.6193528661
H	-5.5705857568	-2.2875460818	-2.8439789273
H	-8.3911748133	-2.2214260571	-1.8138858555
H	-6.3821841114	2.187972105	-0.8767777554
H	-4.8992022079	2.51801539	-1.7949897116
H	-7.1269512761	-0.7475949575	2.1191866603
H	-8.6393705813	-0.1514214006	1.4170702889
H	-2.9830504382	-2.0110251447	-2.9123927228
H	-4.597481045	3.2556109401	0.3088272069
H	-6.2016575009	1.5238478576	1.4467061244
H	-6.4144713756	3.6674175548	1.988141775
H	-8.0638614091	4.3185675402	1.7888795138
H	-7.5099714391	3.8121882883	3.3804808015

calc_**2d** conf_39

C	-6.1715936243	-2.4790635999	-1.0653475296
C	-7.4265365183	-1.9164285263	-0.3983594332
C	-7.0613850794	-0.4237629769	-0.157070357
N	-5.5918928968	-0.3516081109	-0.1562042502
C	-5.0773136378	-1.7243905956	-0.3075018472
C	-4.942201478	0.4772872748	-1.1679040801
C	-3.5243656338	-0.0941035817	-1.1971783019
C	-3.6945075785	-1.5972894354	-0.9863405399
O	-6.2749407064	-2.0967421723	-2.4238700092
O	-8.5894875454	-2.1102499692	-1.1517098274
C	-4.929073953	1.9617665659	-0.8356238229
C	-7.6945743345	0.0654338086	1.139583642
O	-3.6134863919	-2.2530991175	-2.2595213761
O	-6.1909908278	2.6009850147	-0.9714566409
N	-7.4975183225	1.4783185126	1.3507543857
O	-7.8040835521	1.3685582876	3.5939350984
C	-7.5515412515	2.0221618491	2.5906107189
C	-7.2844424493	3.5140175608	2.6519749545
H	-4.9749018165	-2.1899414544	0.6830288382
H	-6.0961185779	-3.5716453487	-0.9630429443
H	-7.5878825053	-2.4149242638	0.5644607831
H	-7.4598835905	0.1548604681	-1.0006633994
H	-5.412978464	0.3453640816	-2.1610522428
H	-2.9883407632	0.1123558079	-2.126012226
H	-2.9482258646	0.3257887928	-0.3658805204
H	-2.9019940955	-1.9979898602	-0.3469385234
H	-5.3765398235	-2.1725824872	-2.7927618525
H	-8.3187585929	-1.9729433123	-2.0734537052

```
H  -4.1846373964  2.4697933718   -1.4653499875
H  -4.6417967727  2.0898138337   0.2104384523
H  -7.2675147341  -0.472080051   1.9923668562
H  -8.7656531041  -0.1798059141  1.1109887304
H  -3.4652038276  -3.1941726293  -2.0998506579
H  -6.4842501473  2.500299073    -1.8863501517
H  -7.2338197321  2.0453820498   0.5530103464
H  -6.9853254257  3.9448967959   1.6932083602
H  -8.187760024   4.0205721381   3.0022264706
H  -6.5024789609  3.7001681201   3.3918565898
```

calc_**2d** conf_40

```
C  -6.2305595872   -2.3930119586  -0.7951928462
C  -6.9784188983   -1.6964611378  0.3427911625
C  -6.514845576    -0.2288830483  0.1970558521
N  -5.1230976604   -0.3369663688  -0.2290365892
C  -4.855601012    -1.7290557444  -0.6610617901
C  -4.6516720516   0.5674682908   -1.2708882147
C  -3.4382257301   -0.1805887518  -1.8153555391
C  -3.9075298096   -1.6325803906  -1.8706030523
O  -6.9630257927   -2.047043667   -1.9507749347
O  -8.3734587454   -1.8582211897  0.2729361164
C  -4.3061164273   1.9452507747   -0.7349472747
C  -6.6872266952   0.5598419386   1.4913723948
O  -4.6156828888   -1.9220390422  -3.0773326556
O  -3.7407587985   2.6790765799   -1.8040923827
N  -8.0866905782   0.6285540578   1.8489861843
O  -7.9755843351   2.7320099684   2.6898165441
C  -8.631302991    1.7377959117   2.411445097
C  -10.1207057339  1.6640540464   2.6802095318
H  -4.3260768508   -2.2614182199  0.1420004344
H  -6.1792977954   -3.4830921833  -0.6702901326
H  -6.6598029998   -2.0946501782  1.3127468357
H  -7.1324644434   0.2367070032   -0.5883162219
H  -5.3995519328   0.6873960783   -2.0749364352
H  -3.0938542022   0.2010551938   -2.7803162115
H  -2.6083533722   -0.0906246483  -1.105594425
H  -3.0787595978   -2.3441112125  -1.7779049199
H  -6.336604684    -2.0437769949  -2.6951246607
H  -8.5814726944   -1.7776457557  -0.6733473136
H  -3.6044910918   1.8289409669   0.1047090312
H  -5.2101057196   2.438470384    -0.3518221596
H  -6.3264854635   1.5861895165   1.3977683208
H  -6.0934765081   0.0827818906   2.2835675733
H  -4.1056964009   -1.5804236051  -3.8219554587
H  -3.5886147849   3.5807485507   -1.4983842109
H  -8.6709508239   -0.152816938   1.5824963575
H  -10.5839813166  0.7304856315   2.3516119391
H  -10.290021589   1.7849270971   3.7530610387
H  -10.6094344175  2.5026571541   2.1787030152
```

calc_**2d** conf_41

```
C  -6.3659501088   -2.3802829183  -0.8753400789
C  -7.1058044039   -1.8137675258  0.3392509818
C  -6.5392564379   -0.3812791714  0.4304703248
N  -5.143328687    -0.5211133601  0.0310479972
C  -4.9537537019   -1.8471456494  -0.6024941612
```

C	-4.5991918277	0.4957633734	-0.8629538417
C	-3.4132373749	-0.2331464024	-1.485261158
C	-3.9556697192	-1.6357320177	-1.7569774769
O	-7.0281095226	-1.8051334942	-1.9815351739
O	-8.5038164636	-1.8684998641	0.2250630968
C	-4.2068108544	1.7787837629	-0.1392033036
C	-6.7202964983	0.2352203349	1.8181880316
O	-4.6330440961	-1.7172119731	-3.0123588151
O	-3.5603207242	2.686842644	-1.0128969179
N	-8.1013596328	0.5911768852	2.0603021813
O	-7.7617420734	2.8092168177	1.6882848573
C	-8.5259137056	1.8753330219	1.8892695025
C	-10.0216559385	2.0832981667	1.9986306251
H	-4.4929504098	-2.5324093916	0.123160833
H	-6.3950551993	-3.4774566271	-0.9220136027
H	-6.8386247368	-2.3778576645	1.24064767
H	-7.0888057715	0.2421205396	-0.2931791287
H	-5.3254348015	0.7501917185	-1.6597964348
H	-3.022920885	0.2600160618	-2.3795780535
H	-2.598695922	-0.2892757782	-0.7543670319
H	-3.168631172	-2.3987233556	-1.7389699927
H	-6.3827376041	-1.7606949894	-2.7074458263
H	-8.6837722919	-1.6262029247	-0.6984490575
H	-3.4909326417	1.5340736271	0.6513153428
H	-5.0824065693	2.2424307325	0.3332725307
H	-6.1423919218	1.1536324662	1.9163502639
H	-6.3722280708	-0.4630078484	2.5881035938
H	-4.0445746367	-1.380961333	-3.6992416753
H	-4.2446215495	3.1315075827	-1.5273667835
H	-8.782229262	-0.1502948456	1.9778916801
H	-10.3846262353	2.5329337381	1.071155395
H	-10.5812365508	1.1657726078	2.1953914478
H	-10.2168619975	2.7968830539	2.8026321585

calc_**2d** conf_42

C	-5.7661892523	-2.4928316375	-0.5006309051
C	-6.9484409956	-1.7615954592	0.1956337856
C	-6.4248822054	-0.3438777335	0.4812927101
N	-5.0519846397	-0.2961853168	-0.0411083842
C	-4.8801121914	-1.3604763599	-1.0311666777
C	-4.7151062057	0.9575476219	-0.7187774659
C	-5.0755258829	0.7586992101	-2.2097216773
C	-5.2407732458	-0.7530975858	-2.4102534608
O	-6.2264503471	-3.3872049889	-1.4961366192
O	-8.0990154	-1.7901906829	-0.6229612066
C	-3.2334448645	1.2792502867	-0.5145331618
C	-6.4129987964	0.0494884599	1.9609029828
O	-6.5881749424	-1.0242399837	-2.8035347709
O	-2.8293067607	2.4401479415	-1.2091812408
N	-7.7269671771	0.3453623113	2.4854164457
O	-7.7187944508	2.4541921789	1.6659175168
C	-8.3072831231	1.5552010995	2.2495773567
C	-9.7236019409	1.7162154114	2.7566296306
H	-3.8284284012	-1.6694762761	-1.0587608733
H	-5.1960939444	-3.0549103507	0.2482199104
H	-7.2387211972	-2.284930127	1.1115283411
H	-7.0853536329	0.3659764898	-0.0382584788
H	-5.3027659088	1.7677229761	-0.2693955146

```
H  -6.0236171021   1.2387106749   -2.4663439268
H  -4.306700536    1.1914098544   -2.8538281555
H  -4.5723606029   -1.1399974641  -3.1877799127
H  -7.1954300145   -3.3946823609  -1.3785958981
H  -7.8630396046   -1.2653947904  -1.417034599
H  -2.6123610688   0.4688604676   -0.908569276
H  -3.0289276547   1.3516886939   0.562743593
H  -5.8043897625   0.9486709285   2.0761908607
H  -5.952649737    -0.7419294225  2.55984309
H  -6.6100484415   -1.9931805729  -2.8939826219
H  -3.308041435    3.1888118829   -0.8308168415
H  -8.2855523912   -0.3987850202  2.8698932405
H  -10.4024246215  1.7682892284   1.9009757431
H  -10.0498137923  0.9073365775   3.4150901406
H  -9.7972277294   2.665403838    3.2905163209
```

calc_**2d** conf_43

```
C  -6.2612228921  -2.4872152827  -0.9576251495
C  -7.6191601618  -1.9235863284  -0.521663556
C  -7.339070302   -0.4119886439  -0.2862231215
N  -5.8848590321  -0.2658422977  -0.1790919237
C  -5.2957735296  -1.6123616714  -0.1651112307
C  -5.2170259692  0.5162660925   -1.2322191291
C  -3.7850330186  -0.0192368446  -1.2185148761
C  -3.8515500064  -1.4748779863  -0.7081241608
O  -6.1097629239  -2.2584059239  -2.3665603929
O  -8.6596711754  -2.1821133807  -1.4327534298
C  -5.3021615816  2.0004012567   -0.8894216089
C  -8.096257706   0.0733189532   0.9529017161
O  -3.5077411193  -2.4584890648  -1.6541389404
O  -4.7766255194  2.2679900906   0.384882306
N  -8.0307885625  1.4959317899   1.1825419774
O  -6.565188272   1.361362788    2.9063397877
C  -7.2581354683  2.0434452673   2.1655040002
C  -7.2779194304  3.5518075732   2.2436579391
H  -5.2924592223  -2.0010667327  0.8616607813
H  -6.1435958877  -3.5527257577  -0.737318914
H  -7.9248182693  -2.3974279061  0.4173088384
H  -7.6990356489  0.1476862317   -1.1624611913
H  -5.6848143752  0.3476374573   -2.2165864897
H  -3.316265837   0.0146098579   -2.204697818
H  -3.1876850208  0.5919616451   -0.5366231726
H  -3.1390743654  -1.6143548313  0.107362188
H  -6.5825511766  -2.970499293   -2.8176543671
H  -8.4051436171  -1.7374181847  -2.2538181198
H  -6.3513701532  2.3316413116   -0.9645883826
H  -4.7249676677  2.5839763546   -1.61400793
H  -7.6713665287  -0.3983990653  1.8422026905
H  -9.1453544277  -0.2346263621  0.8645174785
H  -4.2111077782  -2.441877027   -2.3231642555
H  -5.1050267036  1.5542574385   0.9608860898
H  -8.5863210477  2.1073132839   0.6063902109
H  -7.2355302497  3.8509075736   3.2915685013
H  -6.374546682   3.9170515914   1.7467173145
H  -8.1530186705  4.0049460271   1.7699263404
```

calc_**2d** conf_44

C	-6.30723933	-2.3558560508	-0.8909111236
C	-7.6360985904	-1.5228979765	-0.8383191228
C	-7.2819377599	-0.2714890761	-0.0010299378
N	-5.8299390047	-0.1935521339	0.0588487908
C	-5.358571211	-1.5789708745	0.0528930973
C	-5.1895845551	0.4583232154	-1.1021033458
C	-3.772754468	-0.1163365903	-1.0747161689
C	-3.8730450656	-1.4922212816	-0.3650372567
O	-5.8243521011	-2.3966340667	-2.2169283492
O	-8.0676100625	-1.2151618455	-2.16008108
C	-5.2481227893	1.9778022442	-1.0343168548
C	-7.9051771586	-0.3655664779	1.4033275239
O	-3.4075941756	-2.591356985	-1.0995142987
O	-6.5373147534	2.496959681	-1.2831417461
N	-7.3523317675	0.5634346458	2.3666414851
O	-7.8933463616	2.4255953408	1.2045954848
C	-7.3367390836	1.8987433892	2.1673755543
C	-6.6084069359	2.7200224178	3.2056569241
H	-5.4590198154	-2.0121098781	1.0569375511
H	-6.4684377672	-3.3812773052	-0.5370238326
H	-8.4407233239	-2.108532295	-0.3817632223
H	-7.6722473481	0.6320280003	-0.4695954429
H	-5.6763126692	0.1599859612	-2.0446244376
H	-3.3583419102	-0.2373614454	-2.0779306559
H	-3.1026696845	0.5494807753	-0.5193706053
H	-3.2574703196	-1.4762446211	0.5378013186
H	-6.5638041458	-2.0709016392	-2.7672617964
H	-7.9131614973	-0.2740691127	-2.3181410943
H	-4.5842595393	2.3812196611	-1.8081857489
H	-4.8564214884	2.3048463063	-0.0603673593
H	-7.7687172563	-1.3668164744	1.8217938485
H	-8.9834143071	-0.1973322707	1.3140691674
H	-4.0073008787	-2.6811396537	-1.8587090168
H	-6.9982890909	2.6089692218	-0.4324946058
H	-6.7440081665	0.1904549764	3.0766361922
H	-6.3028484412	2.1481950403	4.0848029543
H	-5.7209036816	3.1645906296	2.7472670195
H	-7.258483495	3.5391765478	3.5189201904

calc_**2d** conf_45

C	-6.1122945284	-2.3623711316	-0.4481618525
C	-7.4594993204	-1.6423387851	-0.7279505487
C	-7.2776750161	-0.2691404437	-0.0784196306
N	-5.8594615041	0.0622028818	-0.2567155818
C	-5.119917153	-1.1999220964	-0.1050959344
C	-5.4827346387	0.699193547	-1.539971592
C	-4.4686781913	-0.2426832195	-2.197173639
C	-3.9008948647	-1.0138472627	-1.0108358514
O	-5.801454786	-3.1482870109	-1.5795092147
O	-7.6271830441	-1.4634704269	-2.1129462195
C	-4.9027622377	2.085930237	-1.2574816089
C	-7.6409293233	-0.2725746024	1.407295839
O	-3.3113192637	-2.2744256072	-1.3114858675
O	-4.4791917755	2.7551163996	-2.4259868919
N	-7.2107752682	0.9557778113	2.0212413885
O	-8.9963435744	1.1694711652	3.4013188363
C	-7.9317890703	1.5948816884	2.9786751099
C	-7.3243027738	2.8839560324	3.4938469723

H	-4.798754889	-1.3245128065	0.9343958141
H	-6.2104197789	-3.014315128	0.4287365584
H	-8.3074766571	-2.1991675223	-0.3035270291
H	-7.8909722547	0.4794250908	-0.5873695916
H	-6.36488183	0.8040961135	-2.1806066269
H	-4.9821606931	-0.945253766	-2.8589284766
H	-3.7173474649	0.3064466888	-2.7716193554
H	-3.1648321401	-0.3980313798	-0.4776372439
H	-4.8299062355	-3.1550338288	-1.6669750516
H	-7.1311724876	-2.2006735609	-2.5136217751
H	-4.0156901653	1.9933124144	-0.6233495704
H	-5.6425756111	2.6747054967	-0.6941256039
H	-7.1903349462	-1.1385423007	1.9150932293
H	-8.7216790141	-0.3558617788	1.5411484297
H	-2.6097102724	-2.1330570891	-1.9596296979
H	-5.2615031265	2.932025063	-2.9638130858
H	-6.3322759287	1.3237248069	1.6858201822
H	-7.1406797236	2.7820167953	4.5662800195
H	-6.3904934323	3.1627878562	2.9990433407
H	-8.0509270151	3.6894396589	3.3650418213

calc_**2d** conf_46

C	-6.3115887871	-2.2585162277	-0.3125425798
C	-7.5914965994	-1.6370961682	-0.9388834552
C	-7.6133869123	-0.2154617683	-0.3636833091
N	-6.2163719327	0.1782867088	-0.2482312564
C	-5.4841471122	-1.0212290977	0.1753036804
C	-5.5782100876	0.7560430573	-1.4480713823
C	-4.3847015905	-0.152080269	-1.765683711
C	-4.0920267634	-0.8186749773	-0.4248438762
O	-5.7102179959	-3.0783067041	-1.295492135
O	-7.4536064385	-1.5577843103	-2.3364429244
C	-5.1596709025	2.195561874	-1.1460789044
C	-8.3206009806	-0.1767000481	1.0007640015
O	-3.3966354458	-2.0607376118	-0.4944403058
O	-4.5072095534	2.8228103735	-2.231111158
N	-8.1809818885	1.0839100189	1.6848902475
O	-6.548489599	0.3588875092	3.0916711075
C	-7.2068535144	1.2741524524	2.6219928404
C	-7.006605301	2.7104063029	3.0563661372
H	-5.4331646761	-1.0473214013	1.2673950943
H	-6.5780387303	-2.8740523149	0.5553788058
H	-8.4890924804	-2.2159624734	-0.674015049
H	-8.1291330143	0.4592893042	-1.0553828952
H	-6.2794245597	0.7569112424	-2.291221798
H	-4.6810409357	-0.92433264	-2.4809725974
H	-3.5416351635	0.4103503125	-2.1773689862
H	-3.5321970939	-0.1354833651	0.2264038286
H	-4.7457749343	-3.0287462119	-1.1544847156
H	-6.8202671854	-2.2670413407	-2.5540785806
H	-4.4479770252	2.2060122088	-0.3154212018
H	-6.0442583836	2.7628059217	-0.8181838674
H	-7.9051318181	-0.929213374	1.6751208634
H	-9.3825518572	-0.4069192317	0.8617410557
H	-2.5569082811	-1.9114094375	-0.9467060852
H	-5.1345697385	2.8655572066	-2.9640626144
H	-8.5788882679	1.9017202583	1.2499787511
H	-7.8794953831	3.3434574686	2.8754575583

H	-6.7622322534	2.7253971294	4.118946608
H	-6.1554168133	3.1295096237	2.510992809

calc_**2d** conf_47

C	-6.1101356391	-1.6428247685	0.2096249742
C	-7.3173104531	-0.7051504784	-0.0476903158
C	-6.7062139613	0.6975767841	0.0102605229
N	-5.3581091023	0.5608750205	-0.5379545662
C	-4.8599922742	-0.7452956394	-0.083322348
C	-5.2148014791	0.6581832386	-2.0063177808
C	-4.6219398562	-0.6795084813	-2.4613606542
C	-3.9155029297	-1.1792194189	-1.2064827406
O	-6.2706601147	-2.7845085081	-0.6108207618
O	-7.8161827763	-0.9092813895	-1.3513118837
C	-4.3132941489	1.8475834126	-2.3413280691
C	-6.6104385178	1.2797440023	1.4273867704
O	-3.6766240185	-2.5819187	-1.1427469357
O	-4.0884727172	1.9965043625	-3.728887692
N	-7.8900564608	1.4811720401	2.0675493111
O	-7.7679345208	-0.3762629076	3.3620787565
C	-8.3762277199	0.6222814776	3.0061820659
C	-9.7304620958	0.9791256196	3.5806954288
H	-4.2989273599	-0.6307554881	0.8504030641
H	-6.0983829338	-1.946323859	1.2622807765
H	-8.1053840686	-0.8626645439	0.6974406114
H	-7.2933580486	1.378897612	-0.6144179444
H	-6.1943462426	0.8108116532	-2.473886573
H	-5.4241100164	-1.3785318656	-2.7127331488
H	-3.9629410516	-0.5608408	-3.3262393255
H	-2.9604551463	-0.6532984268	-1.0772289229
H	-5.3772472028	-3.0822830329	-0.8629154589
H	-7.5818750627	-1.8338787627	-1.5526564158
H	-3.3278103578	1.7062979127	-1.8881477702
H	-4.7418897251	2.7577348174	-1.8960558859
H	-6.0705289143	2.2297236009	1.3726287576
H	-6.0432638879	0.613267287	2.080770269
H	-3.1674569134	-2.8353572462	-1.9228717811
H	-4.9390544396	2.1891193298	-4.1435630308
H	-8.4522846431	2.2690954137	1.7900649054
H	-10.1168846831	1.9438636722	3.2423520737
H	-10.4446727415	0.1976098687	3.3091019574
H	-9.6557677752	0.9864371912	4.6701197599
